# A Potent and Selective Quinolone-Based PTPN22 Inhibitor with Improved Immunotherapeutic Activity

**DOI:** 10.1021/acs.jmedchem.5c03467

**Published:** 2026-07-02

**Authors:** Jianping Lin, Brenson A. Jassim, Yunpeng Bai, Zihan Qu, Frederick Nguele Meke, Jiajun Dong, Li Wu, Benjamin Babalola, Jingmei Yu, Haoran Zhang, Zhong-Yin Zhang

**Affiliations:** † Borch Department of Medicinal Chemistry and Molecular Pharmacology, 311308 Purdue University, West Lafayette, Indiana 47907, United States; ‡ The James Tarpo Jr. and Margaret Tarpo Department of Chemistry, Purdue University, West Lafayette, Indiana 47907, United States; § Institute for Drug Discovery, Purdue University, West Lafayette, Indiana 47907, United States; ∥ Institute for Cancer Research, Purdue University, West Lafayette, Indiana 47907, United States

## Abstract

Protein tyrosine phosphatase nonreceptor type 22 (PTPN22) is a cytosolic enzyme expressed primarily in hematopoietic cells that negatively regulates T cell signaling and antitumor immune response. Genetic and pharmacological studies have demonstrated that PTPN22 is a systemic immunotherapy target that can enhance T cell activation and function when abrogated, resulting in reduced tumor burden. Building on our previously reported PTPN22 inhibitor, **L-1**, we present the design, synthesis, and biological evaluation of a novel series of quinolone-3-carboxylic acid-based inhibitors. In addition to gaining new insights into the structure–activity relationship of this scaffold, compound **L-32** displays improved potency, selectivity, and cellular efficacy compared to **L-1**. Notably, **L-32** exhibits a more favorable pharmacokinetic profile and drug properties, including oral bioavailability. Finally, **L-32** is superior to **L-1** in reducing MC38 tumor growth *in vivo* by promoting robust antitumor immunity, thus offering a promising lead molecule for the development of novel anticancer agents targeting PTPN22.

## Graphical abstract

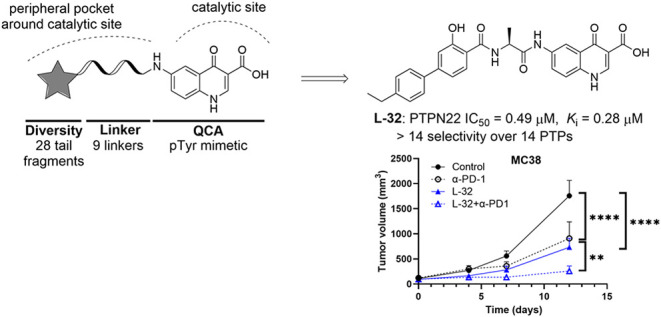

## Introduction

Beginning in the 1980s with the administration of interleukin 2 (IL-2) to contemporary use of adoptive cell transfer (ACT) and immune checkpoint inhibition, immunotherapy, namely leveraging the body’s immune system against cancer, has become mainstream in modern oncology.^1^ Despite their success in the treatment of aggressive cancers such as melanoma and lymphoma, these biological therapeutics are lamentably only efficacious in a small subgroup of patients and cancer types.^2,3^ Further aggravating this impasse, toxic immune-related adverse events (irAEs) are frequently encountered in the clinic,^4,5^ thereby limiting the broad application of these therapies. Consequently, there is intense interest in developing additional strategies that can expand the limited options for immunotherapy and enhance safety and efficacy, either alone or in combination with current approaches.

Among possible therapeutic options, small molecules offer certain potential advantages over current biologic immunotherapies, such as T cell therapies and antibodies, in terms of both toxicity and efficacy. Unlike biologics, small molecules can traverse cell membranes, granting them access to intracellular targets, and can better penetrate the tumor milieu, which provides enhanced efficacy.^6–8^ Notably, the systemic concentration of small molecules can be finely controlled by halting administration and subsequent elimination, potentially circumventing some of the toxicity associated with current biologics.^6–8^ Moreover, small-molecule immunotherapies allow for easier administration and lower development and manufacturing costs. As a result, they can be made accessible to a broader range of patients and may lead to better patient compliance.^7^

Recent genetic and pharmacological studies have identified several protein tyrosine phosphatases (PTPs) as promising intracellular immunotherapy targets.^9,10^ PTPs, along with the complementary protein tyrosine kinases (PTKs), maintain proper levels of cellular protein tyrosine phosphorylation, which regulate numerous cell signaling cascades.^11^ Indeed, cellular processes such as growth, survival, differentiation, metabolism, and the immune response are beholden to this post-translational modification.^12^ Hence, anomalous protein tyrosine phosphorylation is a hallmark of many human pathologies, such as cancer, diabetes, obesity, autoimmune disorders, developmental abnormalities, and neurological diseases.^11,12^ While many small-molecule PTK inhibitors have achieved FDA approval for clinical uses,^13^ PTP drug discovery and development have historically been shrouded by innate difficulties in obtaining potent, selective, and bioavailable inhibitors. These challenges can be attributed to the conserved, electropositive active sites across the PTP family, which impede the development of more selective and drug-like chemical matter.^14^ Nevertheless, PTPs remain highly valued drug targets, especially as their roles in normal and disease states become better defined.

Protein tyrosine phosphatase nonreceptor type 22 (PTPN22) is a cytosolic phosphatase whose expression is exclusive to immune cells.^15^ PTPN22 acts downstream of the T cell receptor (TCR), where it attenuates T cell signaling by dephosphorylating the immunoreceptor tyrosine-based activation motifs (ITAMs) of the TCR CD3 and ζ chains, as well as VAV-1, and the protein tyrosine kinases LCK (Y394) and Zap70 (Y493).^15,16^ PTPN22 also downregulates signaling in other immune cells, such as macrophages,^17^ dendritic cells,^18,19^ neutrophils,^20^ and B cells^21,22^ and promotes the development and function of immunosuppressive regulatory T cells (Tregs).^23,24^ PTPN22 is comprised of a PTP catalytic domain at the N-terminus, followed by an interdomain region and four C-terminal poly proline motifs (P1–P4).^25^ Consistent with the negative regulatory roles of PTPN22 in immunity, a missense C1858T single-nucleotide polymorphism in PTPN22, which converts an Arg at position 620 into a Trp (R620W) within the first Pro-rich region in the C-terminus that disrupts PTPN22 binding to CSK,^26,27^ has been identified as a common risk factor for multiple autoimmune disorders, including type 1 diabetes and systemic lupus erythematosus.^15,28^ Interestingly, the PTPN22 R620W variant is associated with reduced cancer risk and significantly improved responses to checkpoint inhibitor immunotherapy.^29,30^ Moreover, mice with the murine ortholog variant PTPN22 R619W phenocopy those of the *Ptpn22* knockout mice.^19,31,32^ In direct interrogation, genetic abrogation of *Ptpn22* in C57Bl/6J mice led to robust antitumor immunity against the immunogenic MC38 tumor model, increased immune infiltration within tumors, and enhanced response to anti-PD-1 therapy.^29,30^ Similar results have also been observed with the PTPN22 R619W mice,^33^ and the catalytically inactive PTPN22 C227S (active site Cys227 to Ser mutant) mice.^29^ The latter highlights that the loss of PTPN22 catalytic activity, rather than its adaptor function, is crucial in driving its antitumor response, supporting therapeutic strategies that inhibit PTPN22 phosphatase activity. Significantly, *Ptpn22*-deficient mice do not spontaneously develop autoimmune diseases, potentially reducing concerns about immune-related adverse events associated with PTPN22 inhibition.^29,32^ Collectively, these studies demonstrate that PTPN22 is a well-established negative regulator of immune receptor signaling, and strongly suggest that targeting PTPN22 with small-molecule inhibitors represents an attractive immunotherapeutic strategy to unleash both innate and adaptive immunity against tumor cells.

Given the extensive genetic and biochemical evidence implicating PTPN22 as a promising target for immunotherapy, there is growing interest in developing PTPN22 small-molecule inhibitors as a new immunotherapeutic approach for cancer treatment. Unfortunately, most existing PTPN22 inhibitors lack the necessary potency, selectivity, and/or *in vivo* efficacy for clinical translation (Table 1).^34,35^ As part of an ongoing effort to pharmacologically validate PTPN22 as a translatable immunotherapy target, we previously reported inhibitor **L-1**, a competitive small-molecule PTPN22 inhibitor, through a fragment-linking campaign.^30^ This quinolone-based inhibitor exhibited moderate potency (IC_50_ = 1.4 ± 0.2 μM; *K*_i_ = 0.50 ± 0.03 μM) and selectivity (>7- to 10-fold) across a large panel of PTPs. Intraperitoneal administration of **L-1** at 10 mg/kg in mice resulted in an average AUC of 4.55 μM·h, a *C*_max_ of 1.11 μM, and a *t*_1/2_ of 2.03 h. Notably, **L-1** was efficacious in mice, reducing tumor growth across multiple cancer types, including MC38 and CT26, and demonstrated synergy when combined with anti-PD-1 therapy. Critically, **L-1** treatment did not result in significant differences between the vehicle-treated and the **L-1**–treated *Ptpn22*-deficient mice harboring MC38 tumors, suggesting the observed therapeutic effect was PTPN22-dependent. More recently, a different PTPN22 inhibitor was also reported to exhibit *in vivo* antitumor efficacy against MC38, further substantiating PTPN22 as an immunotherapy target.^36^

**Table 1:**
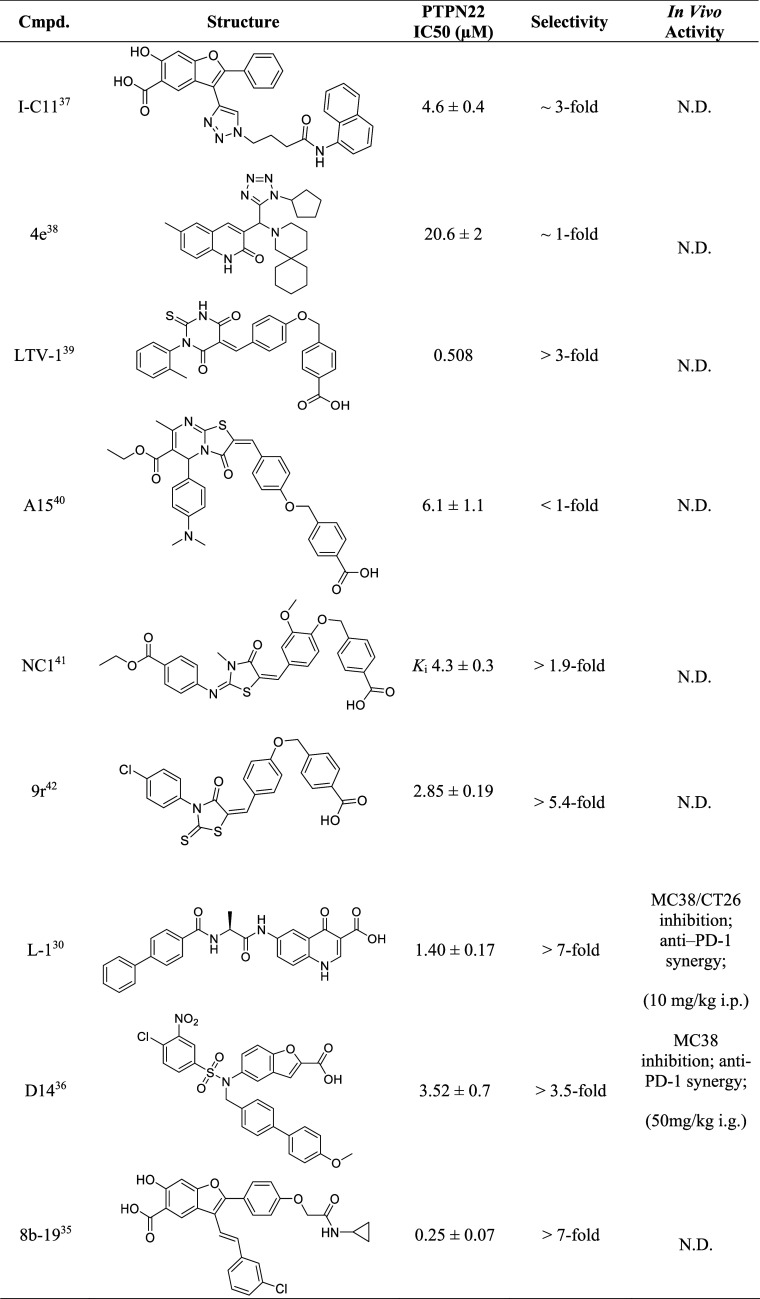
Structure, IC_50_, Selectivity, and *In Vivo* Activity of Reported Small Molecule PTPN22 Inhibitors^30,35–42^ ^a^

a N.D. = Not Determined.

In pursuit of our ultimate goal of developing a PTPN22 inhibitor for clinical application, we endeavored to optimize several properties associated with our previously reported **L-1**, including potency, selectivity, cellular activity, pharmacokinetics, and *in vivo* efficacy. Significantly, we also substantially expanded the structure–activity relationship (SAR) of this scaffold, which will undoubtedly aid in the future development of PTPN22 inhibitors. Herein, we report our ligand-based design, SAR, and synthesis of PTPN22 inhibitors with enhanced potency and isozyme selectivity. Notably, compound **L-32** exhibits improved cellular activity, pharmacokinetics properties, and *in vivo* antitumor efficacy as a monotherapy compared to the lead compound **L-1**. Finally, the leads generated in this study could also be utilized to further probe the therapeutic value of targeting PTPN22 for cancer immunotherapy.

## Results and Discussion

### Fragment-Based Library Approach for Acquisition of PTPN22 Inhibitors

Despite its known roles in cancer immunity, PTPN22 remains underexplored as a therapeutic target due to the scarcity of high-quality small-molecule inhibitors. While PTPs represent attractive drug targets, it has historically been challenging to develop active site-directed, potent, selective, and pharmacologically efficacious PTP inhibitors.^14^ Although success in harnessing allosteric regulatory mechanisms for PTP inhibitor development has been documented in a few cases,^44–46^ the discovery of allosteric inhibitors has been mostly serendipitous. Consequently, a general approach for targeting the PTP active site is highly desirable for the acquisition of orthosteric PTP inhibitors as chemical probes/drugs to advance our understanding of PTPs in disease biology and therapeutic development. Recognizing the importance of pTyr as well as residues flanking it for PTP substrate recognition,^47–50^ we advanced a novel paradigm for the design of potent and selective PTP inhibitors,^51–53^ namely by tethering appropriately functionalized fragments to a nonhydrolyzable pTyr mimetic in order to engage both the active site and unique peripheral binding pockets. We found that natural product-like benzofuran- and indole-based salicylic acid analogs can engage the PTP active site in a similar fashion to that of pTyr, but additional diversity elements attached to the benzofuran/indole core interact with unique secondary pockets in the vicinity of the active site and thereby confer inhibitor selectivity for a number of PTPs.^37,43,54–58^ With this approach, we have developed several potent and selective PTP inhibitors with excellent *in vivo* efficacy.^14^

We initiated our latest effort in PTPN22 inhibitor development with the discovery of a quinolone-3-carboxylic acid (**Core 1**, Table 1) as a PTPN22 active site-directed, nonhydrolyzable pTyr mimetic from an in-house drug-like small molecule collection.^30^ **Core 1** inhibited PTPN22 with an IC_50_ of 432 ± 45 μM. Using a fragment-based focused library approach, we then identified compound **L-1**, which is comprised of **Core 1** linked to a biphenyl carboxylic group via an l-alanine linker. **L-1** was shown to inhibit PTPN22 with an IC_50_ of 1.4 ± 0.2 μM. In our previous study leading to the discovery of **L-1**, a range of linear (e.g., malonic acid, glycine) and more rigid alkyl linkers (e.g., lysine, 1,4-cyclohexanedicarboxylic acid, and oxalic acid) were evaluated but generally showed inferior activity. In contrast, the l-alanine linker provided the most favorable activity profile and was therefore selected as the optimal linker for further optimization. Given that amino acids are synthetically accessible and offer a diverse range of physicochemical properties, they provide an efficient platform for linker optimization and SAR exploration. Accordingly, we expanded our investigation to include a series of amino acid linkers bearing neutral, acidic, and basic side chains to further optimize ligand binding affinity. The structures and IC_50_ values of these compounds are summarized in Table 2. These quinolone-based **L-1** analogues were synthesized according to Schemes 1 and 2.

**Scheme 1:**
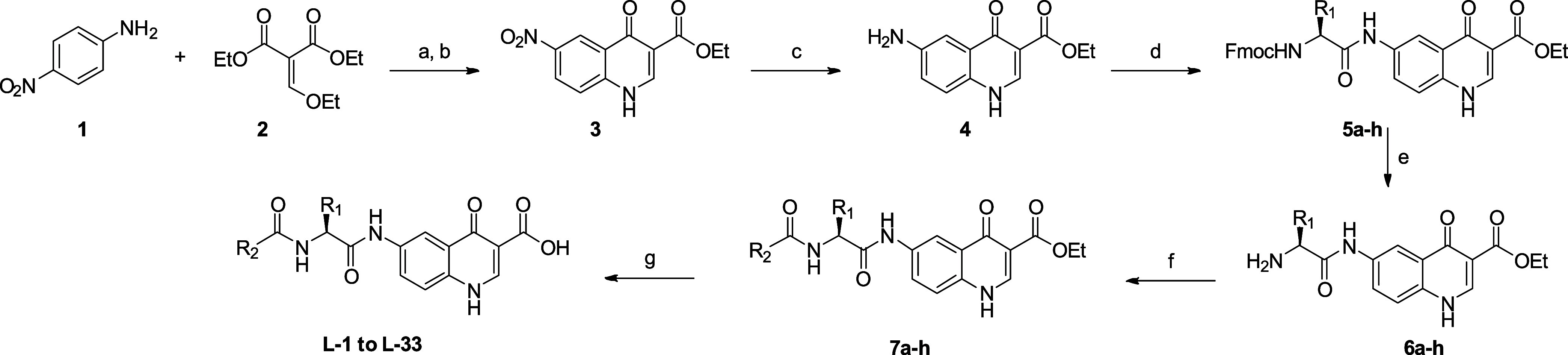
Synthesis of Quinolone-Based Analogues **L-1** to **L-33** ^a^

a Reagents and conditions: (a) 135 °C, 2 h, 75%; (b) PhOPh, reflux, 1 h, 95%; (c) Pd/C, H_2_, DMF, 100 °C, 12 h, 85%; (d) Corresponding Fmoc-amino acid, HOBt, HBTU, DIPEA, DMF, r.t., overnight, 55–71%; (e) Piperidine, DMF, r.t., 1 h, 70–80%; (f) Corresponding carboxylic acid, HOBt, HBTU, DIPEA, DMF, r.t., overnight, 65–85%; (g) KOH, MeOH/H_2_O, 60 °C, 16 h, 75–93%.

**Scheme 2:**
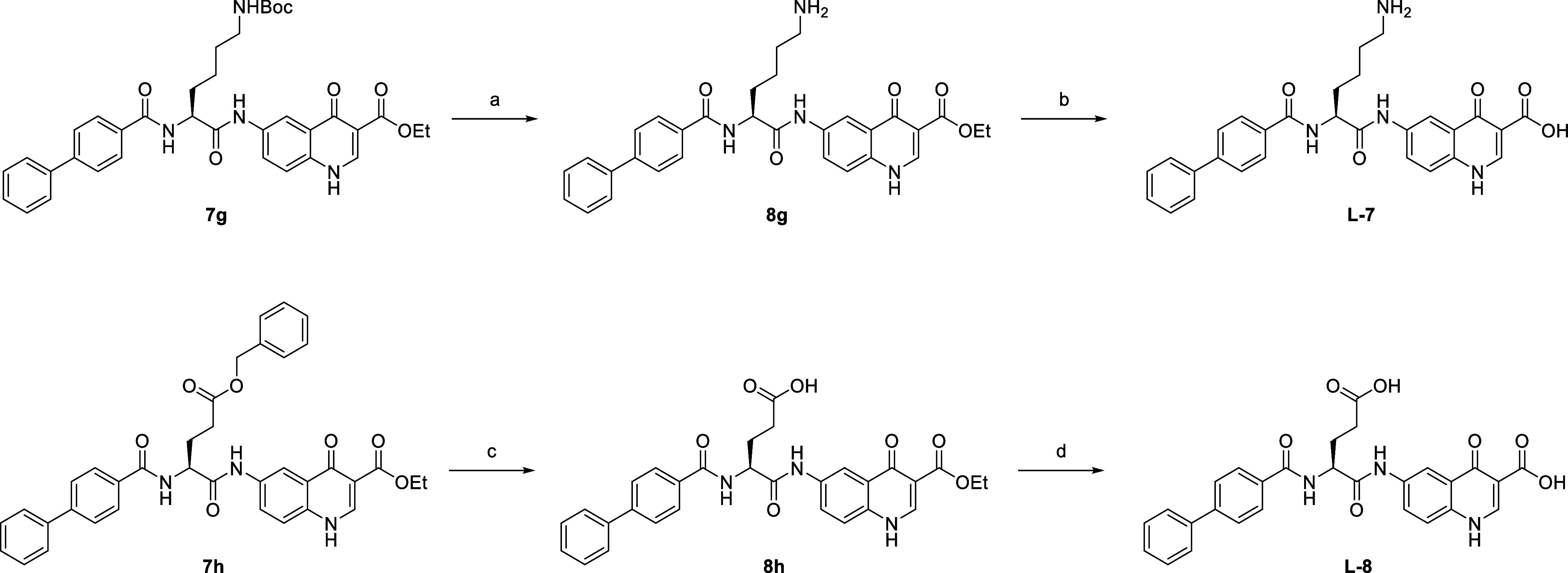
Synthesis of Quinolone-Based Analogues **L-7** and **L-8** ^a^

a Reagents and conditions: (a) CF_3_COOH, DMF, r.t., 3 h, 80%; (b) KOH, MeOH/H_2_O, 60 °C, 16 h, 76%; (c) Pd/C, H_2_, DMF, 100 °C, 12 h, 85%; (d) KOH, MeOH/H_2_O, 60 °C, 16 h, 81%.

**Table 2:**
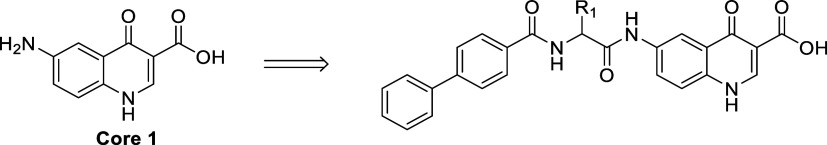

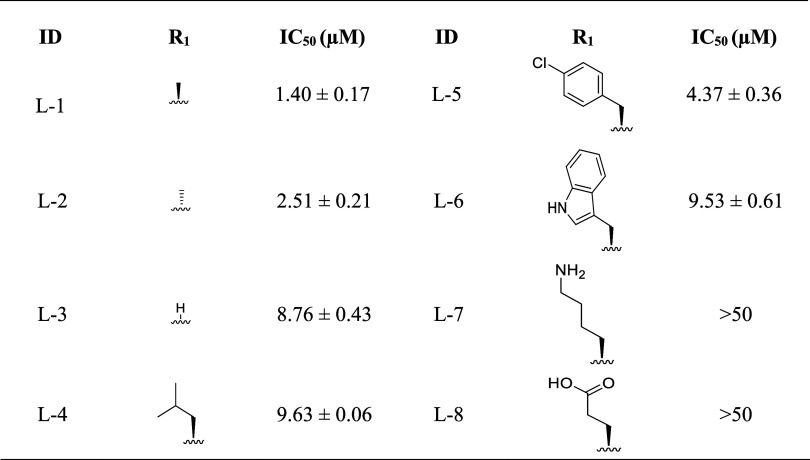
IC_50_ Values (μM) of Quinolone-Based Analogues for PTPN22^a^

a IC_50_ values were determined from three independent measurements.

Starting with *p*-nitroaniline **1** and diethyl ethoxymethylenemalonate **2**, we obtained the key intermediate ethyl 6-nitro-4-oxo-1,4-dihydroquinoline-3-carboxylate **3**. This intermediate was then hydrogenated using 5% palladium on carbon (Pd/C) and hydrogen (H_2_), yielding compound **4** in good yield. To obtain Fmoc-protected intermediates **5a**–**5h**, we conducted a condensation reaction between compound **4** and various Fmoc-protected amino acids. This reaction took place in DMF with the presence of HOBt, HBTU, and DIPEA. The Fmoc group was subsequently removed using a 20% piperidine solution in DMF at room temperature, yielding compounds **6a**–**6h**. Compounds **7a**–**7h** were obtained by coupling compounds **6a**–**6h** with corresponding carboxylic acids under similar reaction conditions as the previous condensation. Finally, we performed a hydrolysis using an aqueous solution of potassium hydroxide (aq. KOH) in a mixture of methanol and water as solvent. This process provided moderate to high yields of **L-1** analogs as free acids after aqueous workup with HCl (Scheme 1).

To obtain the target molecules **L-7** and **L-8**, we had to perform additional steps because they each had other reactive functional groups on their linkers. For compound **L-7**, we treated the Boc-protected intermediate **7g** with trifluoroacetic acid in DMF for 3 h, resulting in the formation of **8g**. In the case of compound **L-8**, the intermediate **7h** was treated with hydrogen and Pd/C to remove the benzyl protection group, producing compound 8h in good yield. Both intermediates **8g** and **8h** were then separately subjected to the aforementioned hydrolysis process to yield the corresponding final products, **L-7** and **L-8** (Scheme 2).

### l-Alanine Linker is Optimal for PTPN22 Inhibition

To establish structure–activity relationships (SAR) for the linker region, we systematically evaluated a series of amino acid-derived linkers (Table 2). Rather than observing isolated changes in potency, several clear trends emerged. First, l-alanine provided the optimal balance of size, flexibility, and neutrality, affording the most potent compound **L-1**. Increasing steric bulk or introducing bulky aromatic substituents (e.g., leucine/**L-4**, tryptophan/**L-6**) led to substantial losses in activity, suggesting limited tolerance for steric expansion in this region. Conversely, increasing linker flexibility (e.g., glycine/**L-3**) was also detrimental, indicating that a certain degree of conformational constraint is required for productive binding. Notably, introduction of charged residues (e.g., lysine/**L-7**, glutamate/**L-8**) completely abolished activity, highlighting the importance of maintaining a neutral linker environment. Additionally, the reduced potency of d-alanine/**L-2** relative to l-alanine/**L-1** underscores the stereochemical requirement for optimal binding. Collectively, these results identify l-alanine as an optimal linker that balances conformational control and physicochemical compatibility with the binding site.

### Design and Synthesis of Novel Biaryl **L-1** Analogs

**L-1** possesses moderate potency (IC_50_ = 1.4 ± 0.17 μM) and selectivity (>7–10-fold selectivity) for PTPN22, along with a half-life (*t*_1/2_) of 2.03 h, an AUC of 4.55 μM·hr, and a *C*_max_ of 1.11 μM in mice. Notably, **L-1** was the first small-molecule PTPN22 inhibitor to exhibit robust *in vivo* efficacy against multiple tumor types in mice and to phenocopy the antitumor effects observed in genotypic *Ptpn22* knockout mice.^30^ Notwithstanding these promising attributes, **L-1**’s potency, selectivity, and *in vivo* efficacy could be further optimized to enable the translation of PTPN22 as a novel target for cancer immunotherapy.

With the linker optimized, we next focused on the biphenyl tail to further improve potency and physicochemical properties (Table 3).^59–61^ SAR analysis revealed that both the orientation and electronic properties of the distal aromatic ring are critical for activity. Positional isomerization of the distal phenyl ring (ortho/**L-9** or meta/**L-10**) led to marked reductions in potency, indicating a strong preference for para-substitution and a defined spatial arrangement. Similarly, replacing the phenyl ring with smaller or alternative aromatic systems (e.g., thiophene/**L-11**, furan/**L-12**, naphthalene/**L-13**) was generally unfavorable, suggesting that both size and planarity are important for optimal interactions.

**Table 3:**
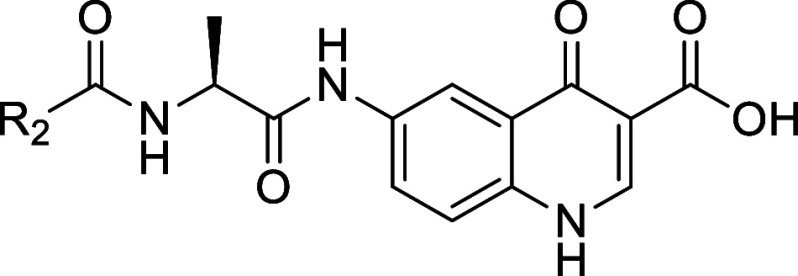

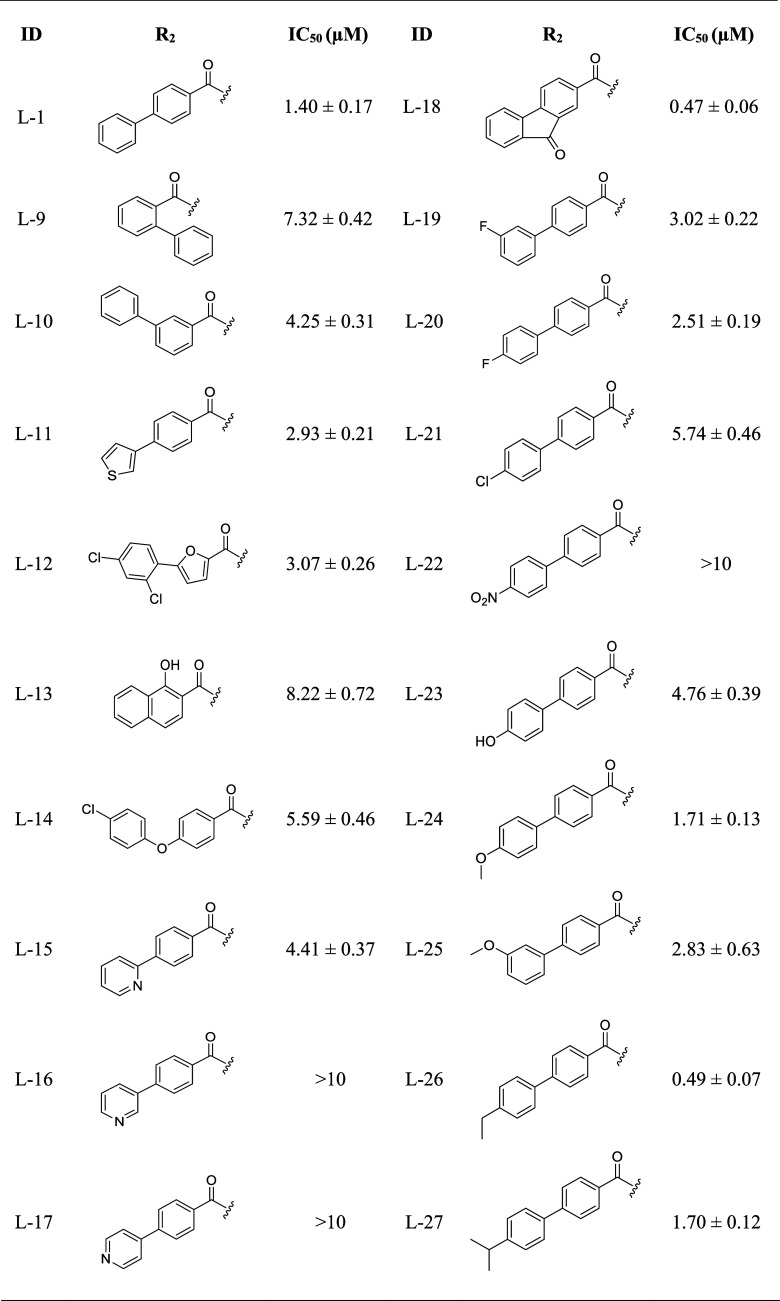
IC_50_ Values (μM) of Quinolone-Based Analogues for PTPN22^a^

a IC_50_ values were determined from three independent measurements.

Electronic effects also played a key role: electron-withdrawing substitutions or heteroaromatic replacements (e.g., pyridine) diminished activity, indicating that maintaining sufficient electron density in the distal ring is important for binding. In contrast, hydrophobic substituents at the para-position were better tolerated, with small alkyl groups (e.g., ethyl/**L-26**) improving potency, likely through enhanced hydrophobic interactions. Furthermore, rigidification of the scaffold (e.g., fluorenone/**L-18**) also improved activity, suggesting that conformational restriction can favor the bioactive binding mode. Together, these findings define key design principles for this scaffold, including the importance of linker-controlled geometry, para-oriented aromatic interactions, and balanced electronic and hydrophobic properties.

Out of our newly designed **L-1** derivatives, **L-18** and **L-26** were the only two compounds with improved potency relative to **L-1**. To facilitate the selection of our next lead and advance our optimization campaign, we next assessed their specificity for PTPN22 against the related PTPs SHP-2 and PTP1B. Of the two second-generation inhibitors, **L-26** possessed the best overall selectivity profile and was markedly more selective than **L-1** (Table 4). For example, **L-18** and **L-26** were 14.7 and 13.3-fold more selective for PTPN22 against SHP-2, while **L-1** displayed 7.9-fold selectivity against SHP-2. Moreover, although **L-1** and **L-18** were both roughly 18-fold selective for PTPN22 over PTP1B, **L-26** was 27.5-fold more selective. Since **L-18** was not readily accessible for additional synthetic modifications on the fluorene ring system, we thus advanced **L-26** to the next round of modification.

**Table 4:** Selectivity of 2nd-Generation Compounds for PTPN22 against SHP-2 and PTP1B^a^

IC_50_ (μM)			
ID	PTPN22	SHP-2	PTP1B
L-1	1.40 ± 0.17	11.07 ± 0.69 (7.9-fold)	>25 (17.9-fold)
L-18	0.47 ± 0.06	6.91 ± 0.59 (14.7-fold)	7.68 ± 0.44 (17.5-fold)
L-26	0.54 ± 0.07	7.19 ± 0.58 (13.3-fold)	14.83 ± 1.48 (27.5-fold)

a IC_50_ values were determined from three independent measurements.

Having explored the **L-1** distal ring, we next performed SAR studies on the penultimate phenyl ring. Fluorine is a frequently employed isostere of hydrogen (present in ∼25% of all FDA-approved drugs^62^ that can contextually improve metabolic stability and enhance cell permeability and ligand binding affinity.^63^ While introducing a fluorine at the *ortho*-position of the penultimate phenyl ring markedly reduced binding affinity (**L-28**), substitution at the *meta-*position (**L-29**) resulted in a 2.9-fold increase in potency (Table 4). Substitution of the same position with chlorine (**L-30**) and methyl (**L-31**) resulted in decreased potency compared to **L-29** (Table 5). Interestingly, the incorporation of a hydroxyl group *meta* to the penultimate ring (**L-32**) resulted in similar potency as **L-29**. It is well documented that aromatic fluorine atoms *ortho* to amides can form an intramolecular 6-membered ring with the amide −NH,^64^ while aromatic hydroxyls can interact with adjacent amide carbonyls via a 6-membered intramolecular hydrogen bond.^65,66^ In the case of both **L-29** and **L-32**’s slightly improved potency, it is possible that the intramolecular 6-membered rings may facilitate formation of the bioactive conformation by keeping the amide and phenyl ring coplanar. Likewise, the larger chlorine atom of **L-30** and the methyl group of **L-31** are unable to form these intramolecular pseudorings. They are likely to result in a different conformation compared to **L-29** and **L-32**. Notably, shuffling this −OH group *ortho-* to *meta-*position (**L-33**) led to an almost 17-fold reduction in biochemical potency compared to **L-32**. Alternatively, it is also possible that a lone pair on the **L-32** hydroxyl or the δ^–^ fluorine of **L-29** electrostatically interacts with a nearby residue or water, thus improving binding affinity.

**Table 5:**
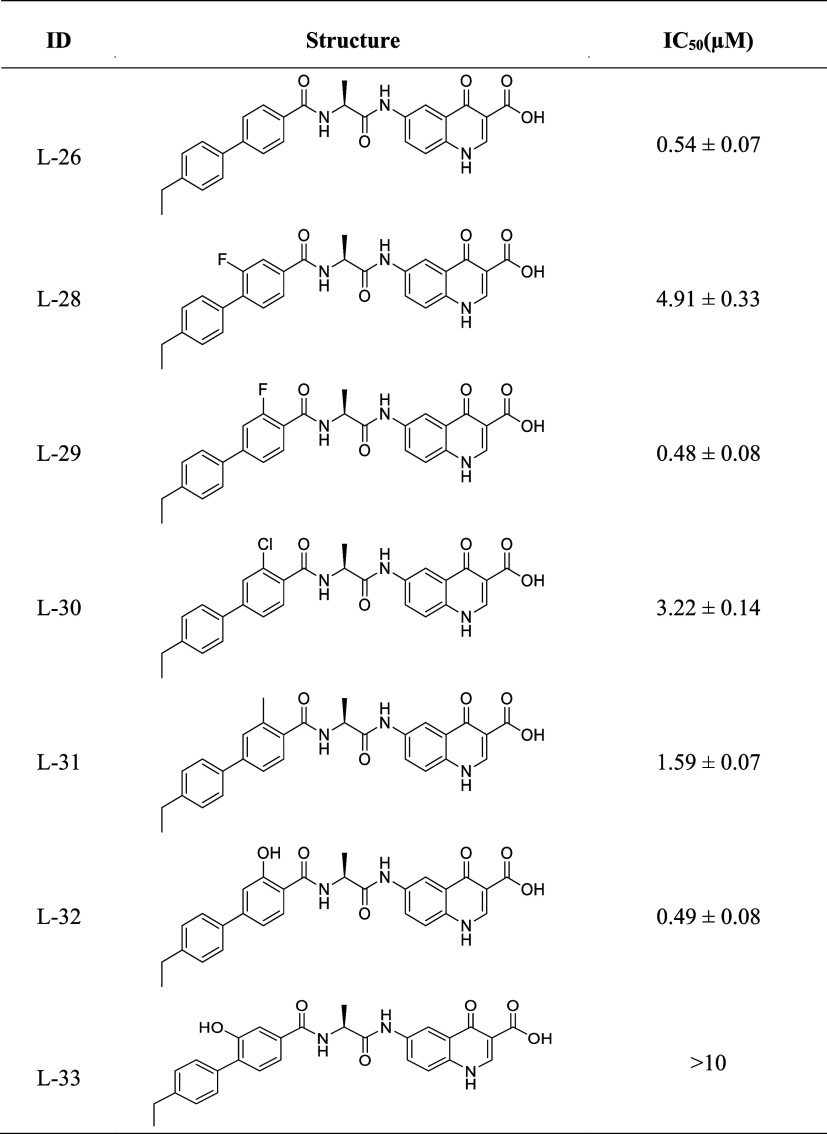
Structures and IC_50_ Values (μM) of **L-26** Analogs for PTPN22^a^

a IC_50_ values were determined from three independent measurements.

### 2nd-Generation **L-1** Analogs **L-26**, **L-29** and **L-32** Possess Improved Potency and Greater Selectivity for PTPN22

Given the paramount importance of PTP inhibitor selectivity, we next evaluated the selectivity of our more potent second-generation hits. When tested against a panel of PTPs, including nonreceptor, receptor, dual-specific, and low molecular weight PTPs, **L-26**, **L-29**, and **L-32** were generally more selective than **L-1** (Table 6). For instance, all three new derivatives exhibit greater than 13-fold selectivity for PTPN22 over SHP-2, while **L-1** possesses only 7-fold selectivity. Interestingly, **L-1** shows 17.9-fold selectivity for PTPN22 over TCPTP, whereas **L-26** and **L-32** exhibit 34.5-fold and 51.0-fold selectivity, respectively. Additionally, **L-1** is 7.9-fold selective for PTPN22 over LMW-PTP, while **L-29** and **L-32** are 21.4 and 19.9-fold selective, respectively. Notably, many of the PTPs tested were not inhibited at concentrations of up to 25 μM. Overall, **L-32** appears to have the most favorable selectivity profile among these derivatives.

**Table 6:** IC_50_ Values and Selectivity of Top Compounds against an Expanded PTP Panel^a^

Compound	L-1 IC_50_(μM)	L-26 IC_50_ (μM)	L-29 IC_50_ (μM)	L-32 IC_50_ (μM)
PTPN22	1.40 ± 0.17	0.54 ± 0.07	0.48 ± 0.08	0.49 ± 0.08
SHP-2	11.07 ± 0.69	7.19 ± 0.58	7.10 ± 0.62	7.11 ± 0.51
PTP1B	>25	14.83 ± 1.48	20.89 ± 1.04	23.43 ± 2.03
PTP-MEG2	>20	>20	>20	>20
TCPTP	>25	18.62 ± 1.67	5.66 ± 0.46	>25
STEP	>25	>25	>25	>25
PTP–PEST	>25	>25	>25	>25
PTPα	>25	>25	>25	>25
PTPε	>25	>25	>25	>25
PTPγ	>25	>25	>25	>25
PPK	>25	>25	>25	>25
Laforin	>25	>25	>25	>25
CDC14A	>25	>25	>25	>25
CD45	>25	>25	>25	>25
LMW-PTP	11.08 ± 4.24	3.96 ± 0.15	11.54 ± 0.89	9.76 ± 0.90

a IC_50_ values were determined from three independent measurements.

To assess the potential for nonspecific aggregation by compound **L-32**, we conducted additional experiments to measure its potency in the presence of the detergent Triton X-100. Triton X-100 is known to diminish the apparent inhibitory potency of nonspecific aggregators.^67,68^ The presence of Triton X-100 in the assay buffer had no impact on the PTPN22 inhibition mediated by compound **L-32** (Supporting Information, Figure S1). The IC_50_ value remained at 0.53 ± 0.03 μM, indicating that compound **L-32** does not inhibit PTPN22 activity through nonspecific aggregation.

To further evaluate the selectivity of **L-32** against non-PTP targets and exclude potential off-target liabilities, we assessed its ability to inhibit kinases such as SRC and CSK. We selected these two kinases because in proximal TCR signaling, PTPN22 constitutively binds to CSK and cooperatively suppresses T-cell activation by jointly modulating SRC-family kinase activity, most notably LCK and FYN, which share high catalytic domain homology with the prototypical SRC kinase. To ensure that the downstream phenotypic effects of **L-32** were mediated specifically by PTPN22 inhibition rather than the disruption of these closely associated enzymatic regulators, we profiled **L-32** against SRC and CSK. **L-32** exhibited no detectable inhibition against these kinases at concentrations up to 10 μM, further supporting its target specificity (Figure S2A). Moreover, given that the quinolone-3-carboxylic acid scaffold contains dual carbonyl groups prone to metal chelation—a known liability for off-target interactions with metal-dependent enzymes—we evaluated the inhibitory potential of **L-32** against metalloenzymes implicated in tumor immunity, such as IDO1 and Arginase. Notably, **L-32** exhibited no inhibitory activity against these enzymes (Figure S2B and C). Collectively, the lack of activity against these diverse non-PTP enzyme classes underscores the robust selectivity profile of L-32 for PTPN22 over potential off-targets.

### **L-32** is a Competitive Inhibitor with Multiple Putative Interactions with PTPN22

Using steady-state kinetics, we determined the mode of PTPN22 inhibition by **L-32**, which, as expected, behaved as a competitive PTPN22 inhibitor with a *K*_i_ of 0.28 ± 0.09 μM (Figure 1A). To gain additional insights into the observed SAR, the molecular basis of inhibition, and selectivity, we performed molecular docking of **L-32** and the PTPN22 active site using Glide63 with an existing crystal structure (PDB 4J51). Our selected docking pose (Figure 1B) reveals several key interactions that generally agree with our observed SAR. Expectedly, the quinolone core, which mimics phosphotyrosine, forms a network of electrostatic interactions with the PTPN22 active site. The carboxylate functionality makes hydrogen bonds with the −NH amide backbone of R233 and the Q278 side chain, and forms ionic bonds with the guanidinium group of R233. Further strengthening ligand affinity, the quinoline core makes a cation−π interaction with K138, while the amide carbonyl adjacent to the quinoline captures a hydrogen bond with the K136 side chain. As shown in the sequence alignment of the tested nonreceptor PTPs in Figure 1C, K136 is only shared by PTPN22 and SHP-2, and may significantly contribute to the observed isozyme selectivity of the **L-1** scaffold. Moreover, the penultimate phenyl ring of the scaffold participates in π–π stacking with the active site Y60.

**Figure 1:**
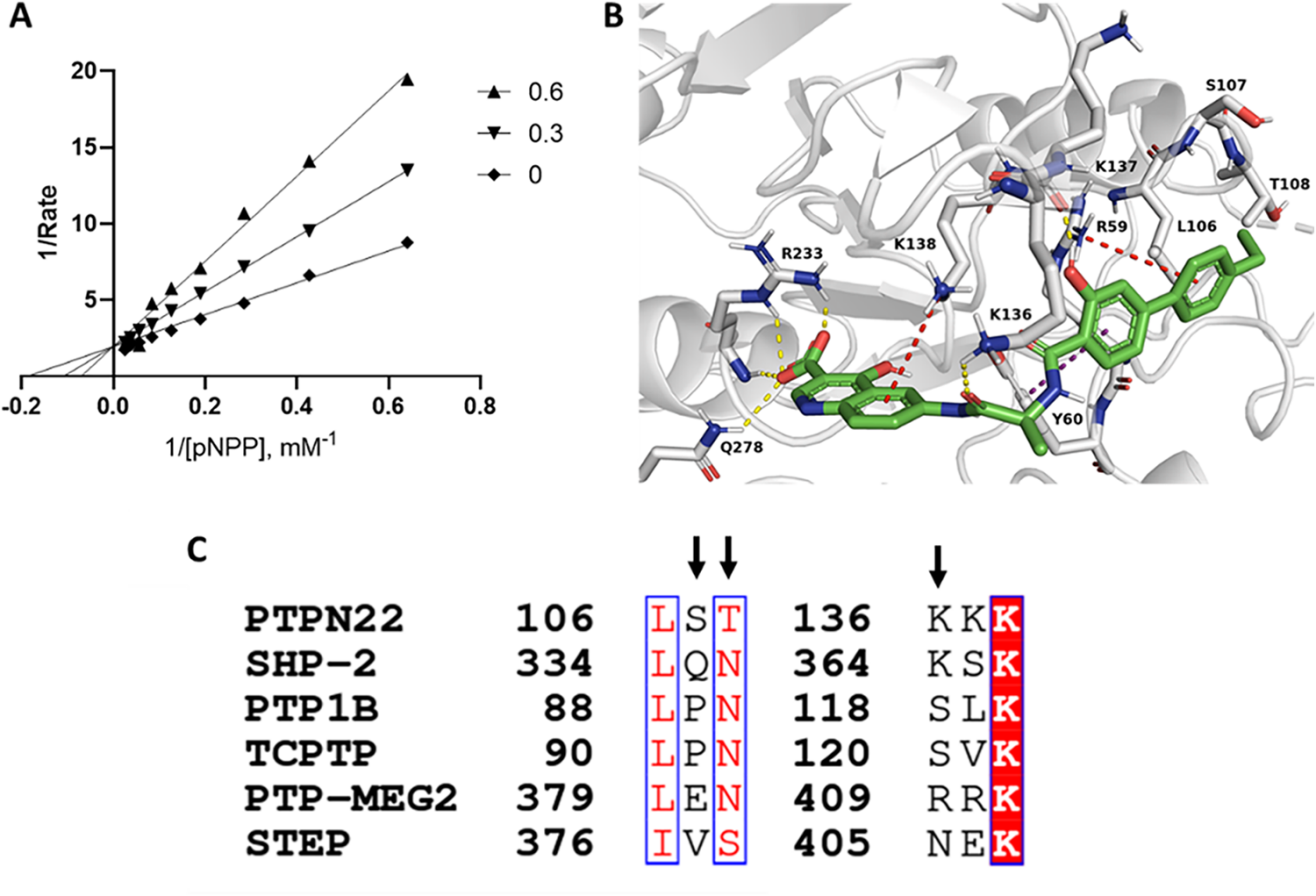
**L-32** competitively inhibits PTPN22 and makes putative interactions with multiple residues that are unique to PTPN22. (A) Lineweaver–Burk plot showing the effect of compound **L-32** on PTPN22-catalyzed hydrolysis of *p*NPP, suggesting competitive inhibition. (B) Docking pose of **L-32** (green stick) bound to the PTPN22 active site, colored by gray element (PDB 4J51). Yellow dashes represent hydrogen bonds and ionic bonds. Cation−π interactions are shown with red dashes, and purple dashes depict π–π interactions. (C) Sequence alignment of tested nonreceptor PTPs showing binding site residues that are unique to PTPN22, marked by a black arrow. Uniprot accession codes used for alignment are as follows: PTPN22 - Q9Y2R2; SHP-2 - Q06124; PTP1B - P18031; TCPTP - P17706; PTP-MEG2 - P43378; and STEP - P54829. Key: red box with white text = identical residues; white box with black text = different residues; white box with red text = similar residues; text outlined with blue box = similar residues across group. The figure was prepared using ESPript 3.0.^69^

Notably, the distal phenyl ring is predicted to form a cation−π interaction with R59, in agreement with our observations that electron-poor pyridine rings and electron-withdrawing substituents such as nitro on the distal ring reduce binding affinity. Moreover, this prediction is in line with the favored directionality (para-substitution) of the distal phenyl ring. Notably, the 4-ethyl group forms van der Waals interactions with the aliphatic regions of the L106 and T108 side chains, thus explaining why the addition of ethyl increases potency. Interestingly, T108 and the nearby S107 are unique to PTPN22 (Figure 1C) and may contribute to the increased selectivity upon the addition of this ethyl group. Lastly, the hydroxyl group of the penultimate ring is predicted to form a hydrogen bond with the K137 backbone. However, this hydrogen bond is likely weak as it lies on the highly solvent-accessible protein surface; hence, this hydrogen bond is likely not a major contributor to binding affinity, which is consistent with the marginally improved potency between **L-32** (hydroxy present) and **L-26** (no hydroxy present). To validate this structural model, we designed and synthesized three additional analogues based on **L-1** and **L-32** to further corroborate the key predicted interactions with PTPN22. The IC_50_ values of these three analogues are summarized in Table 7. Removal of the 3-carboxylic acid group, which serves as the critical phosphotyrosine mimetic, resulted in compound **L-34**, which showed no detectable inhibition of PTPN22 at concentrations up to 100 μM. This result underscores the essential role of the carboxylate moiety in establishing key electrostatic and hydrogen-bonding interactions within the active site. Modification of the amide linkage in **L-1** by replacing the carbonyl group with a methylene group (**L-35**) led to a substantial loss of potency (IC_50_ = 18.4 ± 1.27 μM), corresponding to an approximately 30-fold decrease. This finding highlights the importance of the amide carbonyl in forming a hydrogen bond with K136, as predicted by the docking model. Furthermore, replacement of the distal phenyl ring with a cyclohexyl group (**L-36**) resulted in a dramatic reduction in potency (IC_50_ = 47.3 ± 10.0 μM; ∼80-fold decrease), consistent with disruption of the predicted cation−π interaction with R59 and loss of favorable aromatic interactions. Collectively, these results provide strong experimental support for the key interactions proposed in the docking model (Figure 1B) and further validate the structural basis for the observed SAR.

**Table 7:**
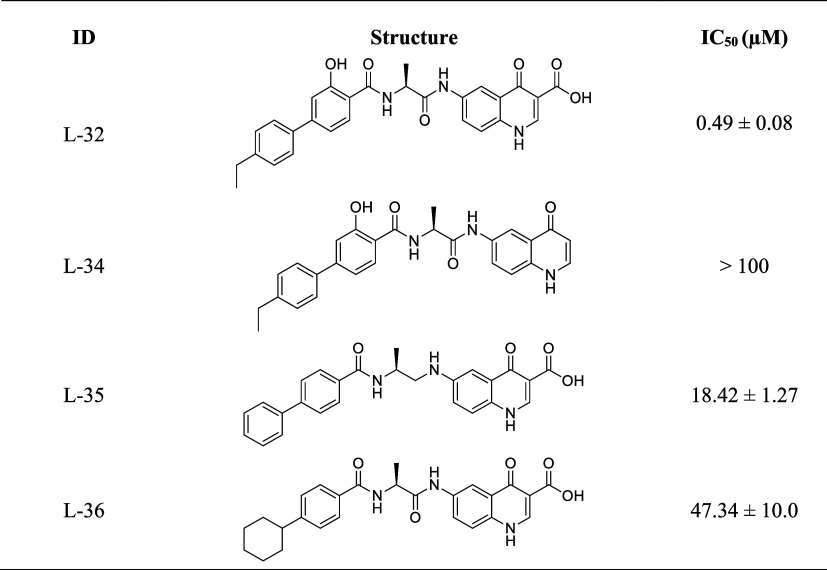
IC_50_ Values (μM) of **L-32** and Its Analogues for PTPN22^a^

a IC_50_ values were determined from three independent measurements.

To further validate the proposed binding mode, we also performed site-directed mutagenesis on residues predicted to interact with **L-32** (Figure 1B). Molecular docking suggested that residues K136, K138, R59, and Y60 form key interactions with **L-32** in the PTPN22 active site. Accordingly, we generated several mutants, including K136S, K136R, K138A, R59A, and Y60A. All wild-type and mutant PTPN22 proteins were expressed in *Escherichia coli* and purified to homogeneity.

As shown in Table 8, the kinetic parameters (*k*_cat_ and *K*_m_) of K136S, K136R, and K138A were comparable to those of wild-type PTPN22, indicating that these mutations do not significantly perturb the overall enzyme structure or catalytic function. In contrast, consistent with the critical role of the pTyr binding loop in PTP catalysis,^43^ the R59A and Y60A mutants exhibited no detectable phosphatase activity.

**Table 8:** Kinetic Parameters of the Wild-Type and Mutant PTPN22 with *p*NPP as a Substrate^a^

Enzyme	*K*_m_ (in mM)	*k*_cat_ (s^‑1^)
Wild-type	5.62 ± 0.32	1.17 ± 0.02
K136S	6.46 ± 0.21	1.72 ± 0.03
K136R	4.4 ± 0.15	1.75 ± 0.02
K138A	7.3 ± 0.43	2.54 ± 0.05
R59A	NA	NA
Y60A	NA	NA

a Kinetics measured at pH = 7.0, in 50.0 mM 3,3-dimethylglutarate, 1 mM EDTA, 0.15 M NaCl buffer at room temperature. NA = showed no measurable phosphatase activity.

We next evaluated the effects of K136 and K138 mutations on PTPN22 inhibition. As summarized in Table 9, both **L-1** and **L-32** exhibited reduced inhibitory potency against the K136S, K136R, and K138A mutants relative to the wild-type enzyme. For the K136S mutant, the shorter side chain is unable to maintain the hydrogen-bonding interaction observed in the wild-type enzyme, resulting in a ∼4-fold and ∼6-fold decrease in potency for **L-32** and **L-1**, respectively. In contrast, the K136R mutant retains a side chain of lightly longer length and functionality, leading to a more modest 2–3-fold reduction in potency for both compounds. For the K138A mutant, disruption of the predicted cation−π interaction results in a greater than 4-fold loss in potency. Notably, **L-32** consistently retained higher potency than **L-1** across all mutants. These results support the involvement of K136 and K138 in ligand binding and are consistent with the proposed binding mode from the molecular docking studies.

**Table 9:** Comparison of IC_50_ Data for **L-1** and **L-32** against Wild-Type and Mutant PTPN22^a^

Entry	Wild-type IC_50_ (μM)	K136S IC_50_ (μM)	K136R IC_50_ (μM)	K138A IC_50_ (μM)
**L-1**	1.4 ± 0.20	9.2 ± 0.81	4.17 ± 0.3	6.18 ± 0.5
**L-32**	0.49 ± 0.08	1.95 ± 0.09	1.11 ± 0.04	2.16 ± 0.19

a IC_50_ values were determined from three independent measurements.

### **L-32** is the Most Efficacious PTPN22 Inhibitor in T Cells

Since compounds **L-26**, **L-29**, and **L-32** acquired improved potency and selectivity compared to **L-1**, we next evaluated their cellular efficacy on potentiating TCR signaling in Jurkat T cells. PTPN22 downregulates T cell signaling, in part, by directly dephosphorylating the activating tyrosine residue (Y394) in LCK. ERK1/2 lies downstream of LCK and is also phosphorylated during T cell signaling; thus, LCK (Y394) and ERK1/2 (T202 and Y204) phosphorylation serve as a readout of T cell activation. Treatment of Jurkat T cells with **L-26**, **L-29**, and **L-32** increased T cell activation compared to **L-1**, with **L-32** showing the best cellular efficacy among the tested compounds, as illustrated by the pLCK and pERK1/2 levels (Figure 2A). We also included our negative control compound, **L-34**, which lacks the key carboxylic acid pharmacophore required for PTPN22 inhibition (Table 7). In line with our previous findings, **L-34** did not show significant effects on LCK and ERK1/2 phosphorylation compared to the DMSO control (Figure 2A and B). Further studies show that **L-32** increased LCK and ERK1/2 phosphorylation levels in a dose-dependent manner (Figure 2B). We next evaluated the effect of **L-32** on T cell activation and function. We found that **L-32** treatment enhanced α-CD28-mediated IL-2 expression (Figure 2C) and secretion (Figure 2D) from Jurkat T cells. As expected, negative control compound **L-34** failed to alter IL-2 level (Figure 2C and D). Finally, we observed that **L-32** treatment increased mouse splenic T cell proliferation (Figure 2E). Altogether, these results confirmed that inhibition of PTPN22 by **L-32** efficiently enhanced T cell signaling and activation.

**Figure 2:**
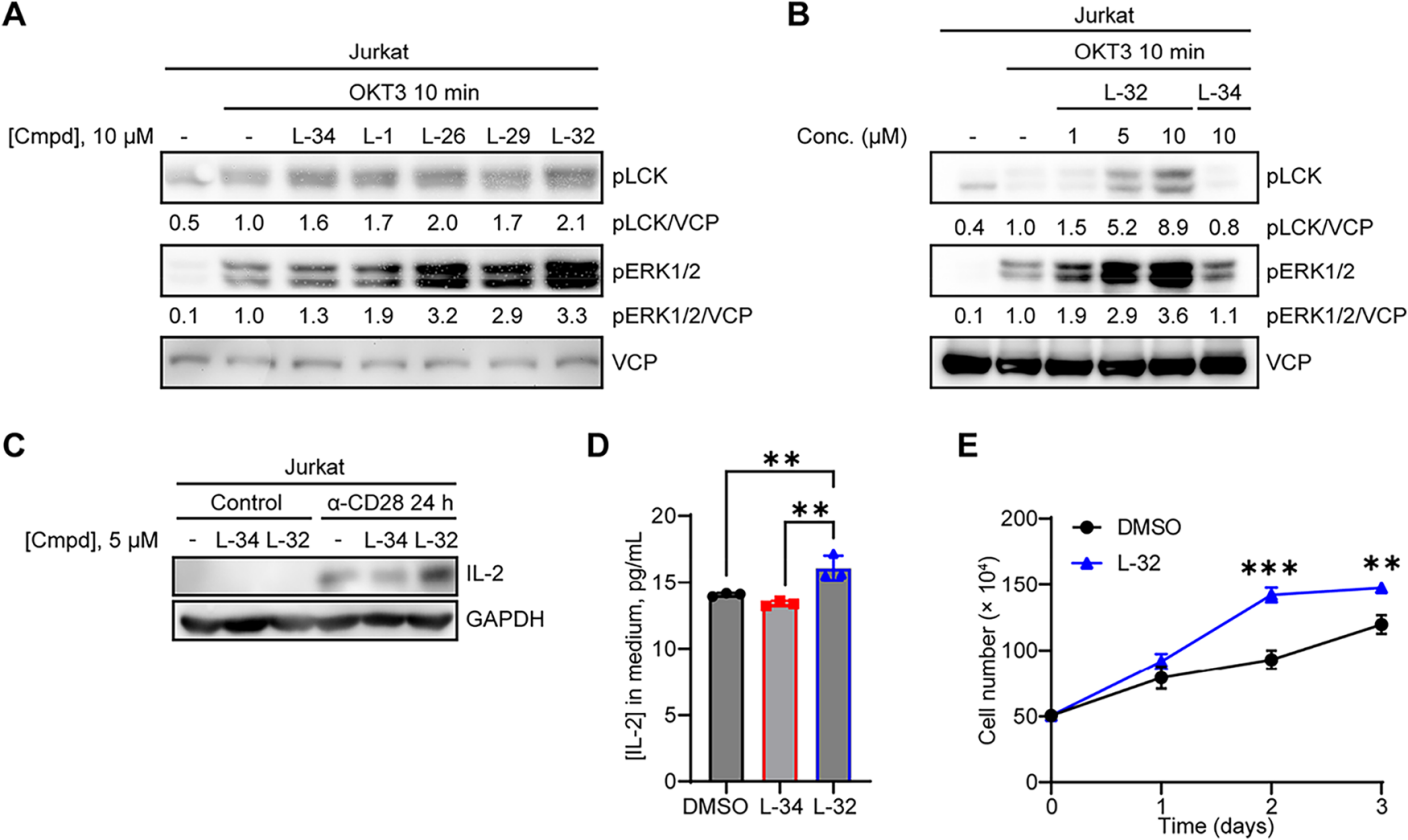
Cellular activity of selected compounds in Jurkat T cells at 10 μM concentration. (A) Effects of **L-1**, **L-26**, **L-29**, and **L-32** on anti-CD3-induced phosphorylation of LCK pY394 and ERK1/2 pT202 and pY204. **L-34** is included as a structurally related negative control. (B) Dose-dependent increase of pLCK and pERK1/2 with **L-32** treatment. **L-34** is included as a structurally related negative control. VCP level was used as a loading control for both experiments. (C, D) Effects of **L-32** and **L-34** on anti-CD28-induced IL-2 expression in Jurkat cells by Western blot (C) and IL-2 secretion in medium by ELISA (D). (E) Effects of **L-32** on cell proliferation of mouse splenic T cells.

### **L-32** Possesses Improved Pharmacokinetic Properties in Mice

Building on the improved potency, selectivity, and cellular efficacy of **L-32** compared with **L-1** and other top analogs, we investigated its *in vivo* PK profile. To this end, **L-32** was administered to three C57BL/6 mice via intraperitoneal (IP) injection at a dose of 10 mg/kg with a formulation of 85% PBS, 10% DMSO, and 5% Cremaphor, which was the same as what was previously used for **L-1**.^30^ **L-32** showed a notable increase in peak plasma concentration (*C*_max_ = 2.31 μM), which was significantly higher than that of **L-1** (*C*_max_ = 1.11 μM). Additionally, **L-32** demonstrated an extended half-life (*t*_1/2_ = 4.73 h), compared to the half-life of 2.03 h observed for **L-1**. Moreover, the plasma exposure (AUC) of **L-32** (15.37 μM·h) is more than 3.0-fold greater than that of **L-1** (4.76 μM·h) (Figure 3). Importantly, pharmacokinetic evaluation following both intravenous (IV) and oral (PO) administration revealed that L-32 possesses favorable oral bioavailability (*F* = 50.4 ± 8.4%) achieving a peak plasma concentration (*C*_max_) of 0.29 ± 0.02 μM at 1 h postdose (Figure S3A). Notably, the oral PK profile exhibited a prolonged terminal phase compared to IV administration, suggesting that while the initial absorption may be limited, the overall systemic exposure is sustained, likely due to slow-release absorption kinetics consistent with flip-flop behavior^70^ (Figure S3A).

**Figure 3:**
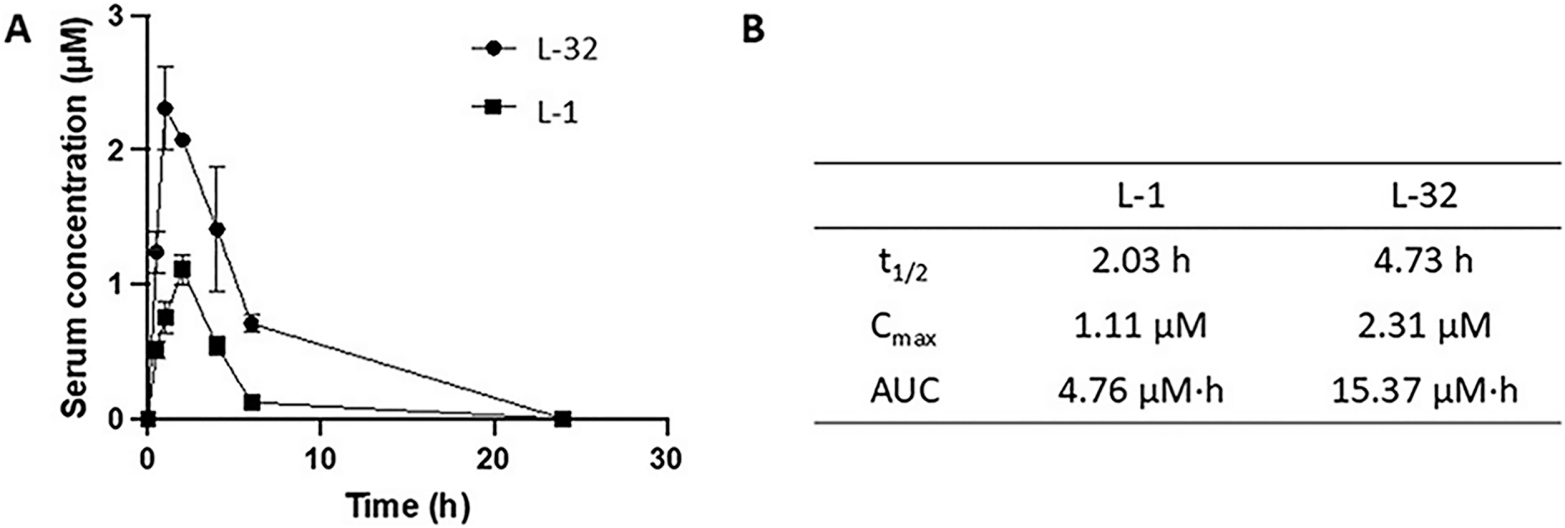
Pharmacokinetic profile of compounds **L-1** and **L-32**. (A) *In vivo* pharmacokinetic data based on mass spectrometric quantification at 0, 0.5, 1, 2, 4, 6, and 24-h time points after a single 10 mg/kg IP dose for three mice. (B) Table of key pharmacokinetic data (10 mg/kg, IP) for **L-32** displayed next to our previously reported **L-1** values for comparison.

We then conducted a more comprehensive preclinical ADME characterization of compound **L-32**, including key physicochemical and ADME parameters, to further evaluate its drug-like properties. **L-32** exhibited a modest aqueous solubility of 35 μM in pH 7.4 PBS buffer and measurable membrane permeability in a PAMPA assay (*P*_e_ = 2.6 × 10^–6^ cm/s). Furthermore, **L-32** exhibited moderate metabolic stability in mouse liver microsomes (*t*_1/2_ = 11.74 min), with an intrinsic clearance (Cl_int_) of 118.06 μL/min/mg (Figure S3B). However, human liver microsome stability studies were not conducted in the present work, and additional evaluation in human metabolic systems will be important in future studies to further assess the translational potential of **L-32**. Collectively, these results show encouraging drug-like properties of **L-32**.

Overall, the PK improvement and favorable drug-like properties of **L-32** establish this new inhibitor as a promising lead compound for further optimization and biological evaluation of its antitumor efficacy as a small-molecule PTPN22 inhibitor.

### **L-32** is Superior at Blocking Syngeneic MC38 Tumor Growth by Activating the Host Antitumor Immunity

Leveraging our new lead molecule’s improved potency, selectivity, cellular efficacy, and pharmacokinetic profile, we next determined **L-32**’s cytotoxicity. To this end, HEK293 cells were treated with **L-1** and **L-32** for 24 h, and their cell viability was measured. As shown in Figure S4, cell viability was not observably affected at compound concentrations up to 30 μM for both **L-1** and **L-32**, indicating that these compounds are nontoxic in cell culture.

It is well-documented that genetic deletion or abrogation of PTPN22 catalytic activity suppresses tumor growth in mice.^29,30^ Given **L-32**’s improved potency, selectivity, cellular efficacy, and *in vivo* pharmacokinetic properties relative to **L-1**, we further investigated the *in vivo* efficacy of **L-32** in an MC38 syngeneic mouse tumor model. Previously, we demonstrated that **L-1** treatment, administered subcutaneously via implanted osmotic pumps or intraperitoneally (IP) twice daily, can suppress the growth of MC38 or CT26 tumors *in vivo*.^30^ To compare the *in vivo* efficacy of **L-32** with **L-1** under the same experimental conditions, we first assessed the antitumor activity with IP injection twice daily at 10 mg/kg with either vehicle control, **L-1**, or **L-32** for 2 weeks (Figure 4A). Consistent with previous results, **L-1** treatment resulted in a ∼38% reduction in tumor volume, while **L-32** further extended this to a ∼83% reduction, indicating a better *in vivo* efficacy of **L-32** compared to **L-1** (Figure 4B, C, and D). We further assessed the compounds’ anticancer activity at lower frequency by IP injection once daily for 2 weeks (Figure S5A). Under this condition, although **L-1** treatment only showed a slight reduction in tumor growth (∼18%) without reaching statistical significance, **L-32** administration readily led to considerably repressed MC38 tumor growth (∼53%) compared to the control group (Figure S5B–D), again indicating that **L-32** is more efficacious than **L-1** in inhibiting MC38 tumor growth *in vivo*. In both cases, no significant reduction in body weight was recorded. (Figures 4E and S5E). Moreover, no signs of toxicity were observed even when animals were dosed at a higher concentration of 100 mg/kg, as indicated by the absence of adverse effects on body weight (Figure S5F). These observations are consistent with **L-32**’s improved biochemical, cellular, and pharmacological properties.

**Figure 4:**
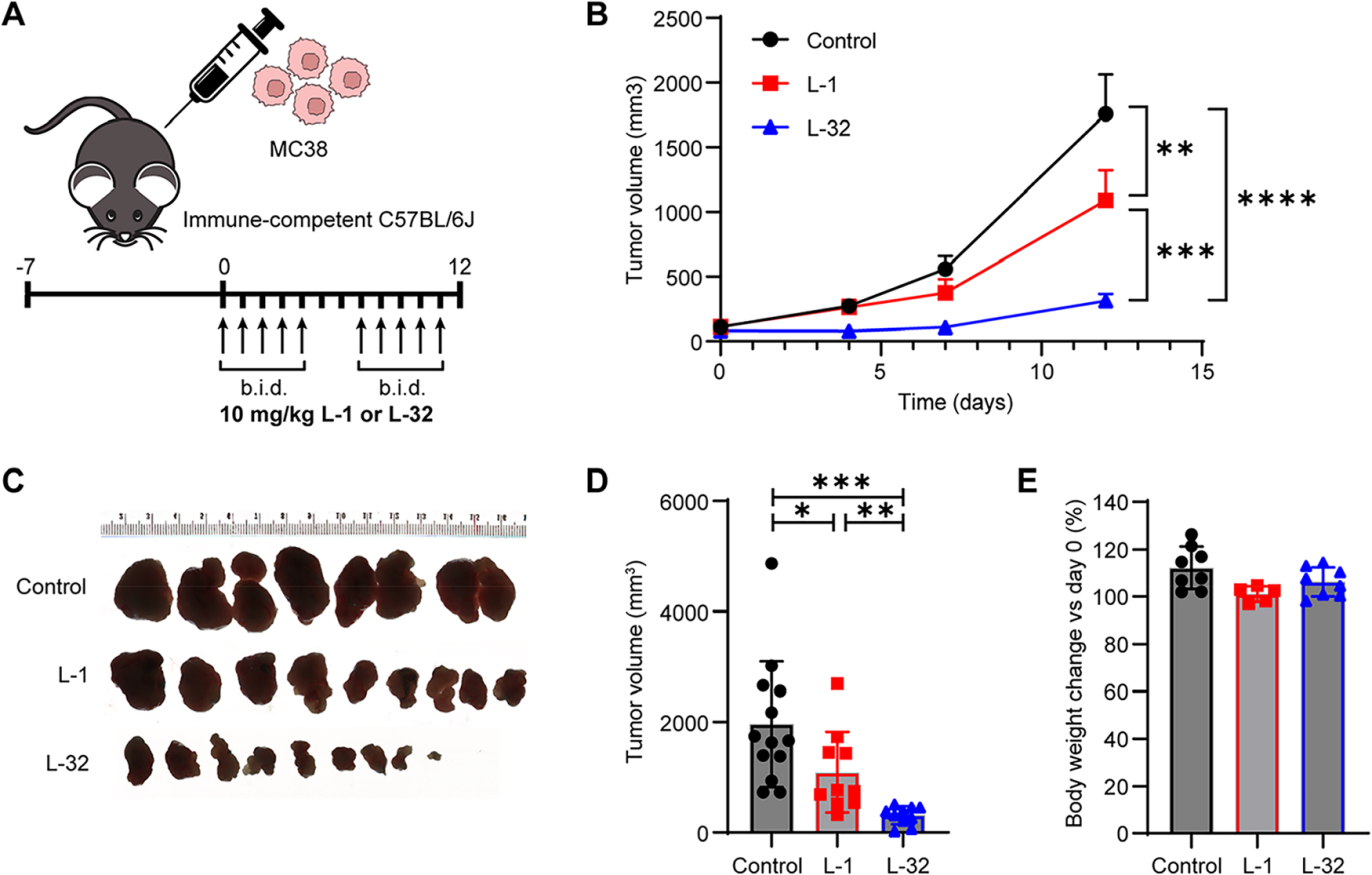
(A) Schematic of the **L-1**/**L-32** treatment of the MC38 tumor model in immune-competent C57BL/6J mice. Mice were injected with 10^6^ MC38 cells at both sides to induce tumor growth, and divided into three groups: Control group (*n* = 8), **L-1** treatment group (*n* = 5), **L-32** treatment group (*n* = 5). Starting day 7 of injection, **L-1**/**L-32** is administered intraperitoneally twice daily at 10 mg/kg for five consecutive days per week for 2 weeks. (B, D) Changes in tumor volume for each treatment group and control. (C) Images of representative dissected tumors. (E) Body weight change compared to day 0 upon treatment of control, **L-1** and **L-32**. Statistical analyses were performed with Graphpad Prism software 11.0.0 through the student *t* test or the one-way ANOVA test using Turkey posthoc comparison. *p < 0.05, **p < 0.01, ***p < 0.001, and ****p < 0.0001 were considered significant. Figures are plotted as average ± standard error of the mean (SEM) for (B) or average ± standard deviation (SD) for (D) and (E).

Most importantly, immunohistochemical analyses revealed a marked increase in CD3+ T-cell infiltration in MC38 tumors (Figure S6), accompanied by a reduction in tumor growth following **L-32** treatment. To validate that **L-32** enhances antitumor immunity through T cell activation, we repeated the *in vivo* MC38 syngeneic tumor experiment and performed systemic immune cells profiling in the tumor microenvironment by flow cytometry (Figure 5). We first confirmed that 10 mg/kg **L-32** effectively reduced MC38 tumor growth (Figure 5A–C) and no systemic toxicity was observed, as evidenced by the stable body weight maintained throughout **L-32** treatment (Figure 5D). CCK8 cytotoxicity assays showed that **L-32** does not impair the growth of MC38 tumor cells or murine splenic T cells at concentrations up to 10 μM (Figure 5E), confirming that the observed antitumor effects were not due to direct cellular toxicity, but rather through modulated immune responses. Furthermore, **L-32** showed no inhibitory activity against the tumor-associated metalloenzymes IDO1 and Arginase (Figure S2B and C), providing additional evidence for its targeted mode of action.

**Figure 5:**
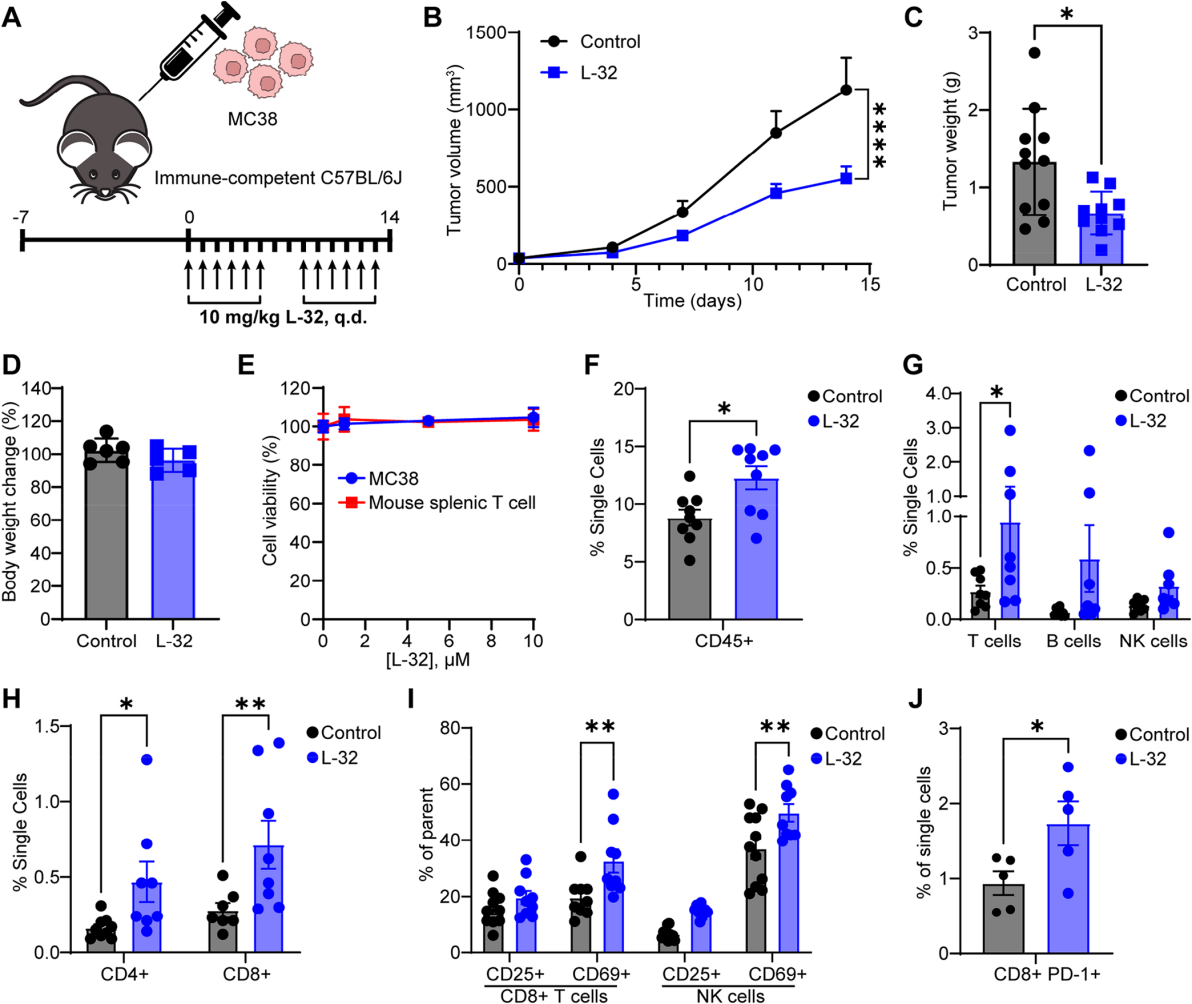
Immune profiling of tumors treated with **L-32**. (A) Schematic of the **L-32** treatment of the MC38 tumor model in immune-competent C57BL/6J mice. Mice were injected with 10^6^ MC38 cells at both sides to induce tumor growth, and divided into two groups: Control group (*n* = 6) and **L-32** treatment group (*n* = 5). Seven days after injection, **L-32** is administered at 10 mg/kg for two cycles of daily intraperitoneal injections for six consecutive days, separated by a two-day treatment-free interval. (B, C) Changes in tumor volume and final tumor weight for control and **L-32** treatment groups. *p < 0.05 and ****p < 0.0001. (D) Body weight change compared to day 0 upon treatment of control and **L-32**. (E) CCK8 cytotoxicity assays for mouse MC38 and splenic T cells upon 24 h treatment of L-32 at 1, 5, and 10 μM. (F–J) Tumor samples were analyzed by flow cytometry to show CD45+ cells (F), T cells, B cells and NK cells (G), CD4+ and CD8+ T cells (H), CD25+ and CD69+ CD8+ T cells and NK cells (I) and PD-1+ CD8+ T cells (J) in the tumor microenvironment. Statistical analyses were performed with Graphpad Prism software 11.0.0 through the student *t* test or the one-way ANOVA test using Turkey posthoc comparison. *p < 0.05, **p < 0.01, and ****p < 0.0001 were considered significant. Figures are plotted as average ± standard error of the mean (SEM) for (B) and (F–J) or average ± standard deviation (SD) for (C) and (D).

Compared to the control group, **L-32** treatment increased CD45+ immune cell infiltration into the tumor microenvironment (Figure 5F). T cells were the primary infiltrating immune cells, though B cells and NK cells also showed an upward trend (Figure 5G). Both CD4+ helper T cells and CD8+ cytotoxic T cells were increased upon **L-32** treatment (Figure 5H). Most importantly, **L-32** treatment stimulated the activation of T cells and NK cells, characterized by a significant increase in CD69 expression and a rising trend in CD25-positive populations (Figure 5I). Furthermore, consistent with previous finding,^30^ **L-32** treatment also led to higher expression of PD-1 in CD8+ T cells, reflecting a nuanced phenotypic shift that typically occurs downstream of initial T cell activation. To further determine whether the antitumor activity of **L-32** is dependent on a functional adaptive immune system, we evaluated its effects on MC38 tumor growth in immune-deficient NRG mice (Figure 6A). As expected, **L-32** failed to inhibit MC38 tumor progression in NRG mice (Figure 6B–D). Notably, **L-32** administration again produced no detectable toxicity in these mice (Figure 6E). This lack of efficacy demonstrates that a functional immune system is indispensable for the antitumor activity of **L-32**, supporting the mechanism where **L-32**-mediated PTPN22 inhibition remodels the tumor microenvironment by promoting cytotoxic CD8+ T cell infiltration and activation.

**Figure 6:**
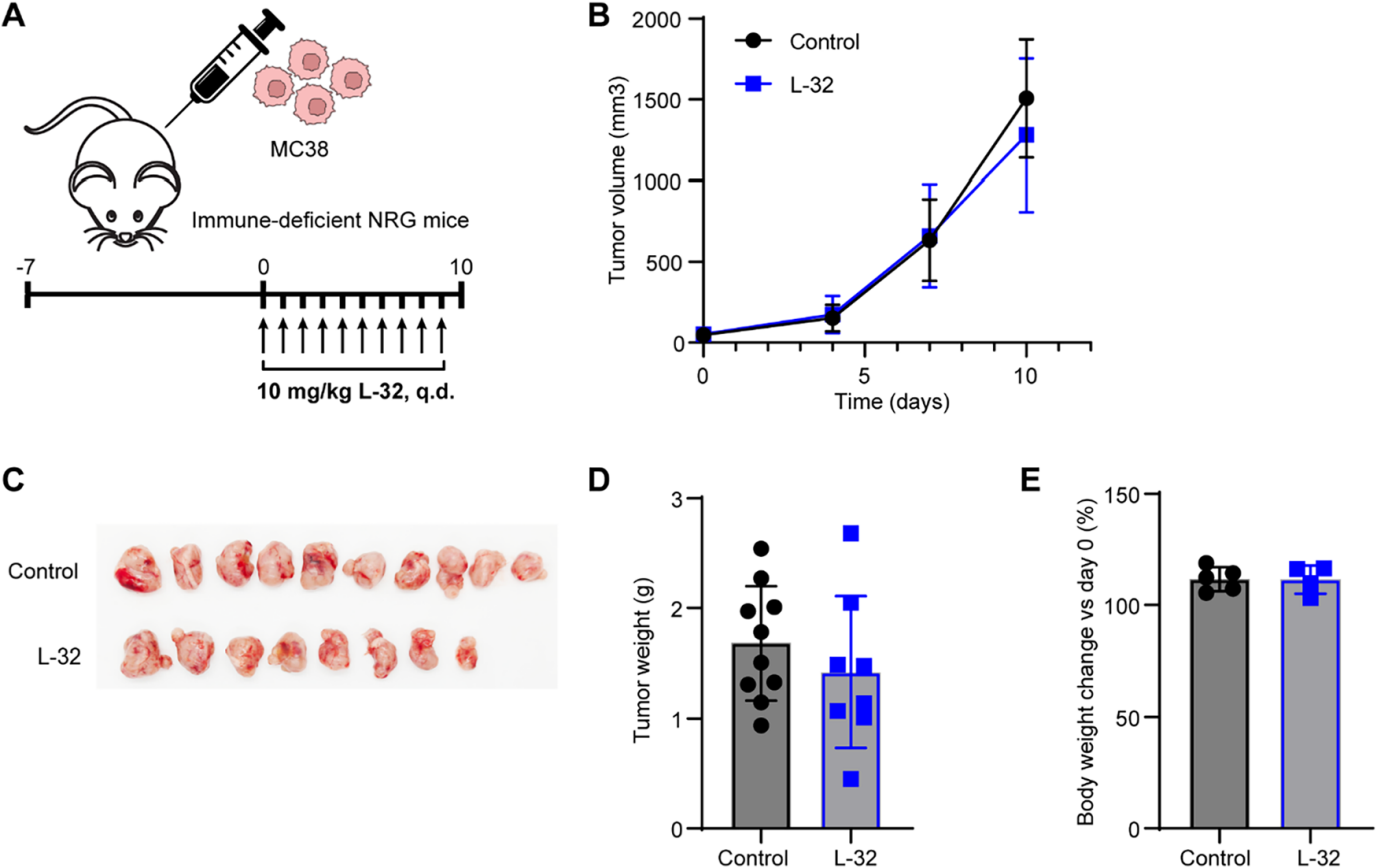
(A) Schematic of the **L-32** treatment of the MC38 tumor model in immune-deficient NRG mice. Mice were injected with 10^6^ MC38 cells at both sides to induce tumor growth, and divided into two groups: Control group (*n* = 5) and **L-32** treatment group (*n* = 4). Starting day 7 of injection, **L-32** is administered intraperitoneally once daily at 10 mg/kg for ten consecutive days. (**B**) Changes in tumor volume for control and **L-32** treatment groups. (C) Images of representative dissected tumors. (D) Weight of tumors in control and **L-32** treatment groups. (E) Body weight change compared to day 0 upon treatment of control and **L-32**. Statistical analyses were performed with Graphpad Prism software 11.0.0 through the student *t* test or the one-way ANOVA test using Turkey posthoc comparison. Figures are plotted as average ± standard deviation (SD).

Our previous findings suggested that PTPN22 abrogation or inhibition by **L-1** can synergize with anti-PD-1 therapy.^30^ Indeed, we also observed that **L-32** treatment led to higher expression of PD-1 in CD8+ T cells (Figure 5I), suggesting checkpoint inhibition by anti-PD-1 antibody would further enhance the antitumor immunity elicited by **L-32**. To further evaluate whether **L-32** can synergize with anti-PD-1 therapy, we treated the MC38 tumor-bearing mice with both anti-PD-1 antibodies and **L-32**. The results showed that the combination arm was superior to all monotherapy arms in the MC38 model (Figure 7A and B) with no obvious toxicity (Figure 7C). Together, these data demonstrate that **L-32** is a more effective PTPN22 inhibitor than **L-1** and a promising lead for further development. Targeting PTPN22 with small molecule inhibitors represents a novel and effective immunotherapy strategy for cancer treatment.

**Figure 7:**
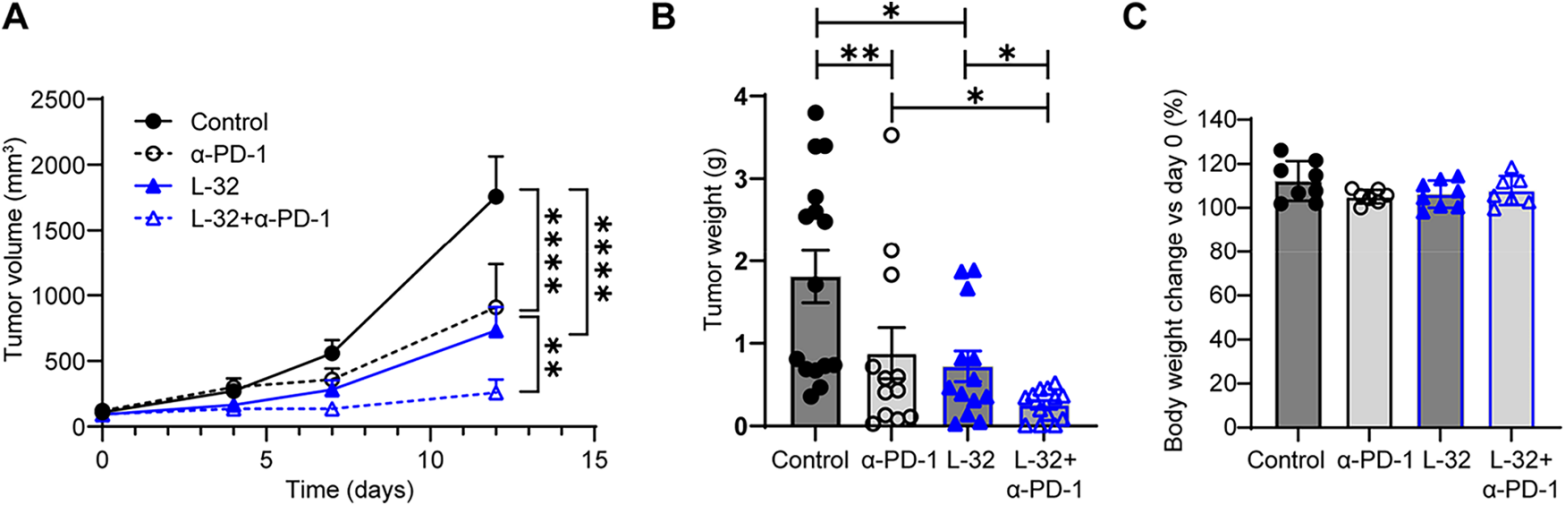
Targeting PTPN22 with **L-32** synergizes anti-PD-1 therapy. (A, B) Mice were injected with 10^6^ MC38 cells at both sides to induce tumor growth, and divided into four groups: Control group (*n* = 8), α-PD-1 treatment group (*n* = 7), **L-32** treatment group (*n* = 8) and **L-32**+α-PD-1 treatment group (*n* = 7). Mice were intraperitoneally injected twice daily with saline or 10 mg/kg **L-32** for five consecutive days per week with or without PD-1 antibody at a dose of 10 mg/kg twice weekly for 2 weeks. Changes in tumor volume (A) and final tumor weight (B) for each treatment group and control. *p < 0.05, **p < 0.01, and ****p < 0.0001. (C) Body weight change compared to day 0 upon treatment of control, α-PD-1, **L-32** or **L-32**+α-PD-1. Statistical analyses were performed with Graphpad Prism software 11.0.0 through the student *t* test or the one-way ANOVA test using Turkey posthoc comparison. *p < 0.05, **p < 0.01, and ****p < 0.0001 were considered significant. Figures are plotted as average ± standard error of the mean (SEM) for (A) and (B) or average ± standard deviation (SD) for (**C**).

## Conclusions

While wielding the body’s immune system via immunotherapy has reached the front line of cancer treatment, the broad application of these therapeutics is hampered by a lack of clinical efficacy in major cancer types and toxicity associated with immune-related adverse events. These shortcomings have spurred the search for novel therapeutic targets for new immunomodulatory agents. PTPN22 is an intracellular negative regulator of immune cells and has emerged as an attractive target for small-molecule immunotherapies, which are particularly desirable due to their advantages in both safety and efficacy. Despite its well-established role in immunotherapy, PTPN22 remains an unexplored therapeutic target due to the lack of high-quality small-molecule inhibitors for clinical translation. To this end, we initiated a lead optimization campaign for our previously reported PTPN22 inhibitor, **L-1**, a moderately potent and selective PTPN22 inhibitor with demonstrated *in vivo* efficacy. Through systematic modification of the linker as well as the distal and penultimate rings of the biphenyl tail, we identified three new lead compounds with improved potency and selectivity. Molecular docking studies and sequence alignment of related nonreceptor PTPs suggest that a key hydrogen bond between the linker and K136 may be a major contributor to the isozyme selectivity, which is supported by site directed mutagenesis and SAR analysis. At the same time, the distal ring’s ethyl group makes hydrophobic contacts with additional residues unique to PTPN22. Of the new leads, **L-32** inhibits PTPN22 with an IC_50_ of 0.49 μM and shows greater than 14-fold selectivity against a large panel of PTPs. In addition, **L-32** exhibits highly efficacious cellular activity and can specifically block PTPN22-dependent signaling within cells. Moreover, **L-32** shows markedly improved drug properties including oral bioavailability. Importantly, **L-32** demonstrates excellent monotherapy efficacy in a syngeneic MC38 tumor model and synergizes with anti-PD-1 to inhibit tumor growth *in vivo* by promoting robust antitumor immunity through enhanced CD8+ cytotoxic T cell and NK cell function. Collectively, our results provide further support for the notion that PTPN22 is a druggable systemic target and that specific PTPN22 inhibitors can be used as effective immunotherapeutic agents. Therefore, **L-32** can not only serve as a valuable tool for advancing the exploration of PTPN22 as a potential therapeutic target for immunotherapy, but also as a promising lead molecule for further development of novel anticancer agents targeting PTPN22.

## Experimental Section

### General Synthetic Procedures and Reagents

Unless otherwise specified, all reagents were purchased from commercial suppliers and used directly without further purification. Analytical thin-layer chromatography (TLC) was performed on 0.25 mm silica gel 60-F_254_. Column chromatography was performed using KP-SIL silica gel (Biotage, USA), and flash column chromatography was performed on Biotage prepacked columns using the automated flash chromatography system Biotage Isolera One. The ^1^H and ^13^C NMR spectra were recorded on a Bruker AVANCE 500 MHz instrument. Chemical shifts for Proton magnetic resonance spectra (^1^H NMR) were quoted in parts per million (ppm) referenced to the appropriate solvent peak or 0.0 ppm for tetramethylsilane (TMS). The following abbreviations were used to describe peak splitting patterns when appropriate: br = broad, s = singlet, d = doublet, t = triplet, q = quartet, m = multiplet, dd = doublet of doublet. Coupling constants, J, were reported in hertz (Hz). Chemical shifts for ^13^C NMR were reported in ppm, referenced to the center line at 39.52 ppm of DMSO-D_6_. HPLC purification was performed on a Waters Delta 600 system equipped with a Sunfire Prep C18 OBD column (30 mm × 150 mm, 5 μm) using a methanol–water (both containing 0.1% TFA) mobile phase (gradient: 50–100% methanol, flow rate 10 mL/min). Low-resolution mass spectra and purity data were obtained using an Agilent Technologies 6470 series, triple quadrupole LC/MS. The purity of all final tested compounds was determined to be greater than 95% (UV, λ = 254 nm). Enantiomer excess (*ee*) values were determined by high-performance liquid chromatography (HPLC) analysis on an Agilent chromatograph using a Daicel Chiralpak AD-H column. High-resolution mass spectra (HRMS) were recorded on an Agilent Mass spectrometer using ESI-TOF (electrospray ionization-time-of-flight).

#### Ethyl 6-Amino-4-oxo-1,4-dihydroquinoline-3-carboxylate (**4**)

To a solution of 6-nitro-4-oxo-1,4-dihydroquinoline-3-carboxylate (compound **3**, 2.0 g, 7.63 mmol) in dimethylformamide (DMF, 40 mL), 10% Pd/C (0.2 g) was added. Hydrogenation was carried out at 1 atm and 100 °C. After stirring for 12 h, the catalyst and solvent were removed, yielding a solid residue. This residue was then washed with ethyl acetate (40 mL) to afford ethyl 6-amino-4-oxo-1,4-dihydroquinoline-3-carboxylate (1.5 g, 85% yield). ^1^H NMR (500 MHz, DMSO-*d*_6_) δ 12.03 (s, 1H), 8.32 (s, 1H), 7.33 (d, *J* = 8.7 Hz, 1H), 7.26 (d, *J* = 2.6 Hz, 1H), 6.99 (dd, *J* = 8.7, 2.6 Hz, 1H), 5.45 (s, 2H), 4.18 (q, *J* = 7.1 Hz, 2H), 1.26 (t, *J* = 7.1 Hz, 3H). Mass spectra (ESI) *m*/*z*: 233.2 (M + H)^+^.

### General Procedures for the Preparation of Compounds **5a**–**5h**

The specified Fmoc-l-amino acid (6.0 mmol, 1.0 equiv), HOBt (7.8 mmol, 1.3 equiv), and HBTU (7.8 mmol, 1.3 equiv) were dissolved in dry dimethylformamide (DMF, 40 mL) and stirred at room temperature for 15 min. Then, ethyl 6-amino-4-oxo-1,4-dihydroquinoline-3-carboxylate **4** (5.4 mmol, 0.9 equiv) and *N*,*N*-diisopropylethylamine (18.0 mmol, 3.0 equiv) were added, with the mixture stirred overnight at room temperature. Following this, DMF was evaporated, and ethyl acetate and water were added. The precipitate was filtered, yielding the corresponding Fmoc-protected intermediates (**5a**–**5h**) as a light-brown solid with a yield of 65–85%. These intermediates were used directly for the next step without further purification.

#### Ethyl (*S*)-6-(2-((((9*H*-Fluoren-9-yl)­methoxy)­carbonyl)­amino)­propanamido)-4-oxo-1,4-dihydroquinoline-3-carboxylate (**5a**)

Brown powder (2.20 g, 70% yield). ^1^H NMR (500 MHz, DMSO-*d*_6_) δ 12.28 (d, *J* = 6.4 Hz, 1H), 10.27 (s, 1H), 8.47 (d, *J* = 6.5 Hz, 1H), 8.39 (d, *J* = 2.1 Hz, 1H), 7.95 (dd, *J* = 8.9, 2.3 Hz, 1H), 7.88 (d, *J* = 7.6 Hz, 2H), 7.75–7.68 (m, 3H), 7.57 (d, *J* = 8.9 Hz, 1H), 7.44–7.37 (m, 2H), 7.35–7.28 (m, 2H), 4.28–4.26 (m, 2H), 4.21–4.17 (m, 4H), 1.32 (d, *J* = 7.1 Hz, 3H), 1.26 (t, *J* = 7.1 Hz, 3H). Mass spectra (ESI) *m*/*z*: 526.2 (M + H)^+^.

#### Ethyl (*R*)-6-(2-((((9*H*-Fluoren-9-yl)­methoxy)­carbonyl)­amino)­propanamido)-4-oxo-1,4-dihydroquinoline-3-carboxylate (**5b**)

Brown powder (2.41 g, 76% yield). Mass spectra (ESI) *m*/*z*: 526.2 (M + H)^+^.

#### Ethyl 6-(2-((((9*H*-Fluoren-9-yl)­methoxy)­carbonyl)­amino)­acetamido)-4-oxo-1,4-dihydroquinoline-3-carboxylate (**5c**)

Brown powder (1.98 g, 65% yield). Mass spectra (ESI) *m*/*z*: 512.2 (M + H)^+^.

#### Ethyl (*S*)-6-(2-((((9*H*-Fluoren-9-yl)­methoxy)­carbonyl)­amino)-4-methylpentanamido)-4-oxo-1,4-dihydroquinoline-3-carboxylate (**5d**)

Brown powder (2.35 g, 69% yield). Mass spectra (ESI) *m*/*z*: 568.2 (M + H)^+^.

#### Ethyl (*S*)-6-(2-((((9*H*-Fluoren-9-yl)­methoxy)­carbonyl)­amino)-3-(4-chlorophenyl)­propanamido)-4-oxo-1,4-dihydroquinoline-3-carboxylate (**5e**)

Brown powder (2.68 g, 70% yield). Mass spectra (ESI) *m*/*z*: 636.2 (M + H)^+^.

#### Ethyl (*S*)-6-(2-((((9*H*-Fluoren-9-yl)­methoxy)­carbonyl)­amino)-3-(1*H*-indol-3-yl)­propanamido)-4-oxo-1,4-dihydroquinoline-3-carboxylate (**5f**)

Brown powder (2.81 g, 73% yield). Mass spectra (ESI) *m*/*z*: 640.3 (M + H)^+^.

#### Ethyl (*S*)-6-(2-((((9*H*-Fluoren-9-yl)­methoxy)­carbonyl)­amino)-6-((*tert*-butoxycarbonyl)­amino)­hexanamido)-4-oxo-1,4-dihydroquinoline-3-carboxylate (**5g**)

Brown powder (3.47 g, 85% yield). Mass spectra (ESI) *m*/*z*: 683.3 (M + H)^+^.

#### Ethyl (*S*)-6-(2-((((9*H*-fluoren-9-yl)­methoxy)­carbonyl)­amino)-5-(benzyloxy)-5-oxopentanamido)-4-oxo-1,4-dihydroquinoline-3-carboxylate (**5h**)

Brown powder (3.03 g, 75% yield). Mass spectra (ESI) *m*/*z*: 674.3 (M + H)^+^.

### General Procedures for the Preparation of Compounds **6a**–**6h**

The Fmoc-protected intermediate **5** (4.0 mmol, 1.0 equiv) was dissolved in DMF (30 mL), and piperidine (7.5 mL) was added. This mixture was stirred at room temperature for 1 h. After concentrating the mixture in vacuo, a brown solid was obtained. This solid was washed with ethyl acetate, yielding the target compounds **6a**–**6h** with yields ranging from 66 to 82%.

#### Ethyl (*S*)-6-(2-Aminopropanamido)-4-oxo-1,4-dihydroquinoline-3-carboxylate (**6a**)

Brown powder (0.84 g, 69% yield). ^1^H NMR (500 MHz, DMSO-*d*_6_) δ 8.49 (s, 1H), 8.43 (d, *J* = 2.4 Hz, 1H), 7.98 (dd, *J* = 8.9, 2.4 Hz, 1H), 7.58 (d, *J* = 8.9 Hz, 1H), 4.21 (q, *J* = 7.1 Hz, 2H), 3.52–3.47 (m, 1H), 1.27 (t, *J* = 7.1 Hz, 3H), 1.25 (d, *J* = 6.9 Hz, 3H). Mass spectra (ESI) *m*/*z*: 304.2 (M + H)^+^.

#### Ethyl (*R*)-6-(2-Aminopropanamido)-4-oxo-1,4-dihydroquinoline-3-carboxylate (**6b**)

Brown powder (0.81 g, 67% yield). Mass spectra (ESI) *m*/*z*: 304.2 (M + H)^+^.

#### Ethyl 6-(2-Aminoacetamido)-4-oxo-1,4-dihydroquinoline-3-carboxylate (**6c**)

Brown powder (0.76 g, 66% yield). Mass spectra (ESI) *m*/*z*: 290.2 (M + H)^+^.

#### Ethyl (*S*)-6-(2-Amino-4-methylpentanamido)-4-oxo-1,4-dihydroquinoline-3-carboxylate (**6d**)

Brown powder (1.02 g, 74% yield). Mass spectra (ESI) *m*/*z*: 346.2 (M + H)^+^.

#### Ethyl (*S*)-6-(2-Amino-3-(4-chlorophenyl)­propanamido)-4-oxo-1,4-dihydroquinoline-3-carboxylate (**6e**)

Brown powder (1.35 g, 82% yield). Mass spectra (ESI) *m*/*z*: 414.2 (M + H)^+^.

#### Ethyl (*S*)-6-(2-Amino-3-(1*H*-indol-3-yl)­propanamido)-4-oxo-1,4-dihydroquinoline-3-carboxylate (**6f**)

Brown powder (1.24 g, 74% yield). Mass spectra (ESI) *m*/*z*: 419.3 (M + H)^+^.

#### Ethyl (*S*)-6-(2-Amino-6-((*tert*-butoxycarbonyl)­amino)­hexanamido)-4-oxo-1,4-dihydroquinoline-3-carboxylate (**6g**)

Brown powder (1.36 g, 74% yield). Mass spectra (ESI) *m*/*z*: 461.3 (M + H)^+^.

#### Ethyl (*S*)-6-(2-Amino-5-(benzyloxy)-5-oxopentanamido)-4-oxo-1,4-dihydroquinoline-3-carboxylate (**6h**)

Brown powder (1.29 g, 72% yield). Mass spectra (ESI) *m*/*z*: 452.3 (M + H)^+^.

### General Procedures for the Preparation of Compounds **7a**–**7h**

Biphenyl-4-carboxylic acid (2.0 mmol, 1.0 equiv), HOBt (2.6 mmol, 1.3 equiv), and HBTU (2.6 mmol, 1.3 equiv) were dissolved in dry DMF (20 mL) and stirred at room temperature for 15 min. Then, compound **6** (1.8 mmol, 0.9 equiv) and *N*,*N*-diisopropylethylamine (6.0 mmol, 3.0 equiv) were added. The mixture was stirred overnight at room temperature. After removing DMF with a rotary evaporator and adding ethyl acetate and water, the precipitate formed was filtered and washed with ethyl acetate, yielding intermediates **7a**–**7h** with a yield of 71–81%.

#### Ethyl (*S*)-6-(2-([1,1′-Biphenyl]-4-carboxamido)­propanamido)-4-oxo-1,4-dihydroquinoline-3-carboxylate (**7a**)

Brown powder (0.71 g, 73% yield). ^1^H NMR (500 MHz, DMSO-*d*_6_) δ 12.30 (d, *J* = 6.7 Hz, 1H), 10.36 (s, 1H), 8.74 (d, *J* = 7.0 Hz, 1H), 8.48 (d, *J* = 6.7 Hz, 1H), 8.41 (d, *J* = 2.3 Hz, 1H), 8.03 (d, *J* = 8.4 Hz, 2H), 8.00 (dd, *J* = 8.9, 2.4 Hz, 1H), 7.79 (d, *J* = 8.4 Hz, 2H), 7.75 (d, *J* = 7.2 Hz, 2H), 7.59 (d, *J* = 8.9 Hz, 1H), 7.50 (t, *J* = 7.6 Hz, 2H), 7.41 (t, *J* = 7.4 Hz, 1H), 4.67–4.61 (m, 1H), 4.21 (q, *J* = 7.1 Hz, 2H), 1.48 (d, *J* = 7.2 Hz, 3H), 1.28 (t, *J* = 7.1 Hz, 3H).Mass spectra (ESI) *m*/*z*: 484.2 (M + H)^+^.

#### Ethyl (*R*)-6-(2-([1,1′-Biphenyl]-4-carboxamido)­propanamido)-4-oxo-1,4-dihydroquinoline-3-carboxylate (**7b**)

Off-white powder (0.69 g, 71% yield). ^1^H NMR (500 MHz, DMSO-*d*_6_) δ 12.30 (d, *J* = 6.7 Hz, 1H), 10.36 (s, 1H), 8.74 (d, *J* = 6.9 Hz, 1H), 8.48 (d, *J* = 6.7 Hz, 1H), 8.41 (d, *J* = 2.4 Hz, 1H), 8.03 (d, *J* = 8.4 Hz, 2H), 8.00 (dd, *J* = 8.9, 2.4 Hz, 1H), 7.80 (d, *J* = 8.4 Hz, 2H), 7.75 (d, *J* = 7.2 Hz, 2H), 7.59 (d, *J* = 8.9 Hz, 1H), 7.50 (t, *J* = 7.6 Hz, 2H), 7.41 (t, *J* = 7.3 Hz, 1H), 4.64 (p, *J* = 6.9 Hz, 1H), 4.21 (q, *J* = 7.1 Hz, 2H), 1.48 (d, *J* = 7.2 Hz, 3H), 1.28 (t, *J* = 7.1 Hz, 3H). Mass spectra (ESI) *m*/*z*: 484.2 (M + H)^+^.

#### Ethyl 6-(2-([1,1′-Biphenyl]-4-carboxamido)­acetamido)-4-oxo-1,4-dihydroquinoline-3-carboxylate (**7c**)

Off-white powder (0.74 g, 78% yield). Mass spectra (ESI) *m*/*z*: 470.2 (M + H)^+^.

#### Ethyl (*S*)-6-(2-([1,1′-Biphenyl]-4-carboxamido)-4-methylpentanamido)-4-oxo-1,4-dihydroquinoline-3-carboxylate (**7d**)

Off-white powder (0.84 g, 79% yield). Mass spectra (ESI) *m*/*z*: 526.3 (M + H)^+^.

#### Ethyl (*S*)-6-(2-([1,1′-Biphenyl]-4-carboxamido)-3-(4-chlorophenyl)­propanamido)-4-oxo-1,4-dihydroquinoline-3-carboxylate (**7e**)

Off-white powder (0.97 g, 81% yield). Mass spectra (ESI) *m*/*z*: 594.2 (M + H)^+^.

#### Ethyl (*S*)-6-(2-([1,1′-Biphenyl]-4-carboxamido)-3-(1*H*-indol-3-yl)­propanamido)-4-oxo-1,4-dihydroquinoline-3-carboxylate (**7f**)

Brown powder (0.86 g, 71% yield). Mass spectra (ESI) *m*/*z*: 599.3 (M + H)^+^.

#### Ethyl (*S*)-6-(2-([1,1′-Biphenyl]-4-carboxamido)-6-((*tert*-butoxycarbonyl)­amino)­hexanamido)-4-oxo-1,4-dihydroquinoline-3-carboxylate (**7g**)

Off-white powder (0.94 g, 73% yield). Mass spectra (ESI) *m*/*z*: 641.3 (M + H)^+^.

#### Ethyl (*S*)-6-(2-([1,1′-Biphenyl]-4-carboxamido)-5-(benzyloxy)-5-oxopentanamido)-4-oxo-1,4-dihydroquinoline-3-carboxylate (**7h**)

Off-white powder (0.91 g, 71% yield). Mass spectra (ESI) *m*/*z*: 632.3 (M + H)^+^.

#### Ethyl (*S*)-6-(2-([1,1′-Biphenyl]-4-carboxamido)-6-aminohexanamido)-4-oxo-1,4-dihydroquinoline-3-carboxylate (**8g**)

The Boc-protected intermediate **7g** (200 mg, 0.31 mmol) was dissolved in DMF (10 mL), and then trifluoroacetic acid (2.0 mL) was added. This mixture was stirred at room temperature for 6 h. After concentrating the mixture in vacuo, a brown solid was obtained. This solid was washed with ethyl acetate, yielding the target compound as a brown powder (125 mg, 74% yield). Mass spectra (ESI) *m*/*z*: 541.3 (M + H)^+^.

#### (*S*)-4-([1,1′-Biphenyl]-4-carboxamido)-5-((3-(ethoxycarbonyl)-4-oxo-1,4-dihydroquinolin-6-yl)­amino)-5-oxopentanoic Acid (**8h**)

To a solution of compound **7h** (200 mg, 0.32 mmol) in dimethylformamide (DMF, 20 mL), 10% Pd/C (0.2 g) was added. Hydrogenation was carried out at a pressure of 1 atm and a temperature of 60 °C. After stirring for 12 h, removal of the catalyst and solvent gave a solid residue, which was then washed with ethyl acetate (5 mL) to afford the desired product (105 mg, 61% yield). Off-white powder; Mass spectra (ESI) *m*/*z*: 540.3 (M – H)^−^.

### General Procedures for the Preparation of Compounds **L-9-m** to **L-33-m**

The corresponding carboxylic acid (2.0 mmol, 1.0 equiv), HOBt (2.6 mmol, 1.3 equiv), and HBTU (2.6 mmol, 1.3 equiv) were dissolved in dry DMF (20 mL) and stirred at room temperature for 15 min. Then, compound **6a** (1.8 mmol, 0.9 equiv) and *N*,*N*-diisopropylethylamine (6.0 mmol, 3.0 equiv) were added. The mixture was stirred overnight at room temperature. After removing DMF with a rotary evaporator and adding ethyl acetate and water, the precipitate was filtered and washed with ethyl acetate, yielding intermediates with yields of 71 to 90%.

#### (*S*)-Ethyl 6-(2-([1,1′-Biphenyl]-2-ylcarboxamido)­propanamido)-4-oxo-1,4-dihydroquinoline-3-carboxylate (**L-9-M**)

Off-white powder (720 mg, 74% yield). ^1^H NMR (500 MHz, DMSO-*d*_6_) δ 12.30 (d, *J* = 6.6 Hz, 1H), 10.23 (s, 1H), 8.50 (dd, *J* = 9.0, 7.1 Hz, 2H), 8.42 (d, *J* = 2.3 Hz, 1H), 7.94 (dd, *J* = 8.9, 2.4 Hz, 1H), 7.59 (d, *J* = 8.9 Hz, 1H), 7.54–7.49 (m, 2H), 7.46–7.39 (m, 4H), 7.33 (t, *J* = 7.5 Hz, 2H), 7.27 (d, *J* = 7.3 Hz, 1H), 4.44 (p, *J* = 7.2 Hz, 1H), 4.21 (q, *J* = 7.1 Hz, 2H), 1.28 (t, *J* = 7.1 Hz, 3H), 1.24 (d, *J* = 7.1 Hz, 3H). Mass spectra (ESI) *m*/*z*: 484.2 (M + H)^+^.

#### (*S*)-Ethyl 6-(2-([1,1′-Biphenyl]-3-ylcarboxamido)­propanamido)-4-oxo-1,4-dihydroquinoline-3-carboxylate (**L-10-M**)

Off-white powder (690 mg, 71% yield). ^1^H NMR (500 MHz, DMSO-*d*_6_) δ 12.30 (d, *J* = 6.4 Hz, 1H), 10.37 (s, 1H), 8.86 (d, *J* = 6.9 Hz, 1H), 8.49 (d, *J* = 6.5 Hz, 1H), 8.42 (d, *J* = 2.4 Hz, 1H), 8.23 (d, *J* = 1.5 Hz, 1H), 8.00 (dd, *J* = 8.9, 2.4 Hz, 1H), 7.91 (d, *J* = 7.8 Hz, 1H), 7.85 (d, *J* = 7.8 Hz, 1H), 7.80–7.74 (m, 2H), 7.63–7.55 (m, 2H), 7.51 (t, *J* = 7.7 Hz, 2H), 7.41 (t, *J* = 7.4 Hz, 1H), 4.66 (p, *J* = 7.1 Hz, 1H), 4.21 (q, *J* = 7.1 Hz, 2H), 1.49 (d, *J* = 7.2 Hz, 3H), 1.28 (t, *J* = 7.1 Hz, 3H). Mass spectra (ESI) *m*/*z*: 484.2 (M + H)^+^.

#### (*S*)-Ethyl 4-Oxo-6-(2-(4-(thiophen-3-yl)­benzamido)­propanamido)-1,4-dihydroquinoline-3-carboxylate (**L-11-M**)

Off-white powder (721 mg, 73% yield). ^1^H NMR (500 MHz, DMSO-*d*_6_) δ 12.30 (d, *J* = 6.1 Hz, 1H), 10.35 (s, 1H), 8.72 (d, *J* = 6.6 Hz, 1H), 8.48 (d, *J* = 6.6 Hz, 1H), 8.41 (s, 1H), 7.99 (t, *J* = 8.7 Hz, 3H), 7.77 (d, *J* = 6.9 Hz, 2H), 7.66 (s, 1H), 7.61 (dd, *J* = 23.6, 6.6 Hz, 2H), 7.23–7.12 (m, 1H), 4.68–4.58 (m, 1H), 4.21 (dd, *J* = 13.6, 6.5 Hz, 2H), 1.47 (d, *J* = 6.4 Hz, 3H), 1.28 (t, *J* = 7.0 Hz, 3H). Mass spectra (ESI) *m*/*z*: 490.2 (M + H)^+^.

#### (*S*)-Ethyl 6-(2-(5-(2,4-Dichlorophenyl)­furan-2-carboxamido)­propanamido)-4-oxo-1,4-dihydroquinoline-3-carboxylate (**L-12-M**)

Off-white powder (831 mg, 76% yield). Mass spectra (ESI) *m*/*z*: 542.2 (M + H)^+^.

#### (*S*)-Ethyl 6-(2-(1-Hydroxy-2-naphthamido)­propanamido)-4-oxo-1,4-dihydroquinoline-3-carboxylate (**L-13-M**)

Off-white powder (752 mg, 79% yield). ^1^H NMR (500 MHz, DMSO-*d*_6_) δ 12.31 (d, *J* = 6.6 Hz, 1H), 10.44 (s, 1H), 9.13 (d, *J* = 6.7 Hz, 1H), 8.49 (d, *J* = 6.7 Hz, 1H), 8.41 (d, *J* = 2.2 Hz, 1H), 8.26 (d, *J* = 8.3 Hz, 1H), 8.09 (d, *J* = 8.9 Hz, 1H), 8.01 (dd, *J* = 8.9, 2.4 Hz, 1H), 7.89 (d, *J* = 8.2 Hz, 1H), 7.65 (t, *J* = 7.5 Hz, 1H), 7.60 (d, *J* = 8.9 Hz, 1H), 7.55 (t, *J* = 7.6 Hz, 1H), 7.42 (d, *J* = 8.9 Hz, 1H), 4.72 (p, *J* = 7.1 Hz, 1H), 4.21 (q, *J* = 7.1 Hz, 2H), 1.54 (d, *J* = 7.2 Hz, 3H), 1.27 (t, *J* = 7.1 Hz, 3H). Mass spectra (ESI) *m*/*z*: 474.3 (M + H)^+^.

#### (*S*)-Ethyl 6-(2-(4-(4-Chlorophenoxy)­benzamido)­propanamido)-4-oxo-1,4-dihydroquinoline-3-carboxylate (**L-14-M**)

Off-white powder (821 mg, 77% yield). Mass spectra (ESI) *m*/*z*: 534.2 (M + H)^+^.

#### (*S*)-Ethyl 4-Oxo-6-(2-(4-(pyridin-2-yl)­benzamido)­propanamido)-1,4-dihydroquinoline-3-carboxylate (**L-15-M**)

Off-white powder (736 mg, 76% yield). Mass spectra (ESI) *m*/*z*: 485.2 (M + H)^+^.

#### (*S*)-Ethyl 4-Oxo-6-(2-(4-(pyridin-3-yl)­benzamido)­propanamido)-1,4-dihydroquinoline-3-carboxylate (**L-16-M**)

Off-white powder (705 mg, 72% yield). Mass spectra (ESI) *m*/*z*: 485.2 (M + H)^+^.

#### (*S*)-Ethyl 4-Oxo-6-(2-(4-(pyridin-4-yl)­benzamido)­propanamido)-1,4-dihydroquinoline-3-carboxylate (**L-17-M**)

Off-white powder (746 mg, 77% yield). Mass spectra (ESI) *m*/*z*: 485.2 (M + H)^+^.

#### (*S*)-Ethyl 4-Oxo-6-(2-(9-oxo-9*H*-fluorene-2-carboxamido)­propanamido)-1,4-dihydroquinoline-3-carboxylate (**L-18-M**)

Off-white powder (913 mg, 90% yield). ^1^H NMR (500 MHz, DMSO-*d*_6_) δ 12.30 (s, 1H), 10.35 (s, 1H), 8.93 (s, 1H), 8.45 (d, *J* = 42.6 Hz, 2H), 8.21 (d, *J* = 21.9 Hz, 2H), 8.09–7.84 (m, 3H), 7.67 (s, 2H), 7.59 (s, 1H), 7.45 (s, 1H), 4.63 (p, *J* = 7.1 Hz, 1H), 4.21 (q, *J* = 7.1 Hz, 2H), 1.48 (d, *J* = 7.2 Hz, 3H), 1.27 (t, *J* = 7.1 Hz, 3H). Mass spectra (ESI) *m*/*z*: 510.3 (M + H)^+^.

#### (*S*)-Ethyl 6-(2-(3′-Fluoro-[1,1′-biphenyl]-4-ylcarboxamido)­propanamido)-4-oxo-1,4-dihydroquinoline-3-carboxylate (**L-19-M**)

Off-white powder (762 mg, 76% yield). ^1^H NMR (500 MHz, DMSO-*d*_6_) δ 12.30 (d, *J* = 6.5 Hz, 1H), 10.36 (s, 1H), 8.74 (d, *J* = 6.9 Hz, 1H), 8.48 (d, *J* = 6.7 Hz, 1H), 8.41 (d, *J* = 2.3 Hz, 1H), 8.08–7.96 (m, 3H), 7.84–7.69 (m, 4H), 7.59 (d, *J* = 8.9 Hz, 1H), 7.33 (t, *J* = 8.8 Hz, 2H), 4.64 (p, *J* = 7.1 Hz, 1H), 4.21 (q, *J* = 7.1 Hz, 2H), 1.47 (d, *J* = 7.2 Hz, 3H), 1.28 (t, *J* = 7.1 Hz, 3H). Mass spectra (ESI) *m*/*z*: 502.3 (M + H)^+^.

#### (*S*)-Ethyl 6-(2-(4′-Fluoro-[1,1′-biphenyl]-4-ylcarboxamido)­propanamido)-4-oxo-1,4-dihydroquinoline-3-carboxylate (**L-20-M**)

Off-white powder (735 mg, 73% yield). ^1^H NMR (500 MHz, DMSO-*d*_6_) δ 12.30 (d, *J* = 6.7 Hz, 1H), 10.36 (s, 1H), 8.77 (d, *J* = 6.9 Hz, 1H), 8.48 (d, *J* = 6.7 Hz, 1H), 8.41 (d, *J* = 2.3 Hz, 1H), 8.04 (d, *J* = 8.4 Hz, 2H), 8.00 (dd, *J* = 8.9, 2.4 Hz, 1H), 7.84 (d, *J* = 8.4 Hz, 2H), 7.65–7.51 (m, 4H), 7.29–7.21 (m, 1H), 4.64 (p, *J* = 7.1 Hz, 1H), 4.21 (q, *J* = 7.1 Hz, 2H), 1.48 (d, *J* = 7.2 Hz, 3H), 1.28 (t, *J* = 7.1 Hz, 3H). Mass spectra (ESI) *m*/*z*: 502.3 (M + H)^+^.

#### (*S*)-Ethyl 6-(2-(4′-Chloro-[1,1′-biphenyl]-4-ylcarboxamido)­propanamido)-4-oxo-1,4-dihydroquinoline-3-carboxylate (**L-21-M**)

Off-white powder (753 mg, 73% yield). ^1^H NMR (500 MHz, DMSO-*d*_6_) δ 12.30 (d, *J* = 6.7 Hz, 1H), 10.36 (s, 1H), 8.76 (d, *J* = 6.9 Hz, 1H), 8.48 (d, *J* = 6.7 Hz, 2H), 8.41 (d, *J* = 2.4 Hz, 1H), 8.04 (d, *J* = 8.4 Hz, 2H), 8.00 (dd, *J* = 8.9, 2.4 Hz, 1H), 7.83–7.77 (m, 3H), 7.61–7.53 (m, 3H), 4.64 (dt, *J* = 14.2, 7.1 Hz, 1H), 4.21 (q, *J* = 7.1 Hz, 2H), 1.47 (d, *J* = 7.2 Hz, 3H), 1.28 (t, *J* = 7.1 Hz, 3H). Mass spectra (ESI) *m*/*z*: 518.3 (M + H)^+^.

#### (*S*)-Ethyl 6-(2-(4′-Nitro-[1,1′-biphenyl]-4-ylcarboxamido)­propanamido)-4-oxo-1,4-dihydroquinoline-3-carboxylate (**L-22-M**)

Off-white powder (761 mg, 72% yield). ^1^H NMR (500 MHz, DMSO-*d*_6_) δ 12.34 (s, 1H), 10.37 (s, 1H), 8.83 (d, *J* = 6.9 Hz, 1H), 8.49 (s, 1H), 8.42 (d, *J* = 2.3 Hz, 1H), 8.33 (d, *J* = 8.8 Hz, 2H), 8.09 (d, *J* = 8.4 Hz, 2H), 8.05 (d, *J* = 8.8 Hz, 2H), 8.00 (dd, *J* = 9.0, 2.3 Hz, 1H), 7.93 (d, *J* = 8.3 Hz, 2H), 7.60 (d, *J* = 8.9 Hz, 1H), 4.65 (p, *J* = 7.0 Hz, 1H), 4.21 (q, *J* = 7.1 Hz, 2H), 1.49 (d, *J* = 7.2 Hz, 3H), 1.28 (t, *J* = 7.1 Hz, 3H). Mass spectra (ESI) *m*/*z*: 529.3 (M + H)^+^.

#### (*S*)-Ethyl 6-(2-(4′-Hydroxy-[1,1′-biphenyl]-4-ylcarboxamido)­propanamido)-4-oxo-1,4-dihydroquinoline-3-carboxylate (**L-23-M**)

Off-white powder (742 mg, 74% yield). ^1^H NMR (500 MHz, DMSO-*d*_6_) δ 12.32 (s, 1H), 10.35 (s, 1H), 9.66 (s, 1H), 8.68 (d, *J* = 6.8 Hz, 1H), 8.48 (s, 1H), 8.41 (d, *J* = 1.8 Hz, 1H), 7.99 (dd, *J* = 16.4, 5.2 Hz, 3H), 7.70 (d, *J* = 8.2 Hz, 2H), 7.59 (dd, *J* = 8.7, 3.0 Hz, 3H), 6.87 (d, *J* = 8.5 Hz, 2H), 4.71–4.56 (m, 1H), 4.21 (q, *J* = 7.0 Hz, 2H), 1.47 (d, *J* = 7.1 Hz, 3H), 1.28 (t, *J* = 7.0 Hz, 4H). Mass spectra (ESI) *m*/*z*: 500.3 (M + H)^+^.

#### (*S*)-Ethyl 6-(2-(4′-Methoxy-[1,1′-biphenyl]-4-carboxamido)­propanamido)-4-oxo-1,4-dihydroquinoline-3-carboxylate (**L-24-M**)

Off-white powder (762 mg, 74% yield). Mass spectra (ESI) *m*/*z*: 514.3 (M + H)^+^.

#### (*S*)-Ethyl 6-(2-(3′-Methoxy-[1,1′-biphenyl]-4-carboxamido)­propanamido)-4-oxo-1,4-dihydroquinoline-3-carboxylate (**L-25-M**)

Off-white powder (751 mg, 73% yield). Mass spectra (ESI) *m*/*z*: 514.3 (M + H)^+^.

#### (*S*)-Ethyl 6-(2-(4′-Ethyl-[1,1′-biphenyl]-4-ylcarboxamido)­propanamido)-4-oxo-1,4-dihydroquinoline-3-carboxylate (**L-26-M**)

Off-white powder (723 mg, 71% yield). ^1^H NMR (500 MHz, DMSO-*d*_6_) δ 12.30 (d, *J* = 6.6 Hz, 1H), 10.35 (s, 1H), 8.72 (d, *J* = 6.9 Hz, 1H), 8.48 (d, *J* = 6.7 Hz, 1H), 8.41 (d, *J* = 2.1 Hz, 1H), 8.00 (dd, *J* = 10.0, 5.3 Hz, 3H), 7.77 (d, *J* = 8.4 Hz, 2H), 7.67 (d, *J* = 8.1 Hz, 2H), 7.59 (d, *J* = 8.9 Hz, 1H), 7.33 (d, *J* = 8.1 Hz, 2H), 4.70–4.57 (m, 1H), 4.21 (q, *J* = 7.1 Hz, 2H), 1.47 (d, *J* = 7.2 Hz, 3H), 1.28 (t, *J* = 7.1 Hz, 3H), 1.22 (t, *J* = 7.6 Hz, 3H). Mass spectra (ESI) *m*/*z*: 512.3 (M + H)^+^.

#### (*S*)-Ethyl 6-(2-(4′-Isopropyl-[1,1′-biphenyl]-4-carboxamido)­propanamido)-4-oxo-1,4-dihydroquinoline-3-carboxylate (**L-27-M**)

Off-white powder (769 mg, 73% yield). Mass spectra (ESI) *m*/*z*: 526.3 (M + H)^+^.

#### (*S*)-Ethyl 6-(2-(4′-Ethyl-2-fluoro-[1,1′-biphenyl]-4-carboxamido)­propanamido)-4-oxo-1,4-dihydroquinoline-3-carboxylate (**L-28-M**)

Off-white powder (758 mg, 72% yield). Mass spectra (ESI) *m*/*z*: 530.3 (M + H)^+^.

#### (*S*)-Ethyl 6-(2-(4′-Ethyl-3-fluoro-[1,1′-biphenyl]-4-carboxamido)­propanamido)-4-oxo-1,4-dihydroquinoline-3-carboxylate (**L-29-M**)

Off-white powder (801 mg, 76% yield). Mass spectra (ESI) *m*/*z*: 530.3 (M + H)^+^.

#### (*S*)-Ethyl 6-(2-(3-Chloro-4′-ethyl-[1,1′-biphenyl]-4-carboxamido)­propanamido)-4-oxo-1,4-dihydroquinoline-3-carboxylate (**L-30-M**)

Off-white powder (814 mg, 75% yield). Mass spectra (ESI) *m*/*z*: 545.2 (M + H)^+^.

#### (*S*)-Ethyl 6-(2-(4′-Ethyl-3-methyl-[1,1′-biphenyl]-4-carboxamido)­propanamido)-4-oxo-1,4-dihydroquinoline-3-carboxylate (**L-31-M**)

Off-white powder (803 mg, 76% yield). Mass spectra (ESI) *m*/*z*: 526.3 (M + H)^+^.

#### (*S*)-Ethyl 6-(2-(4′-Ethyl-3-hydroxy-[1,1′-biphenyl]-4-carboxamido)­propanamido)-4-oxo-1,4-dihydroquinoline-3-carboxylate (**L-32-M**)

Off-white powder (783 mg, 74% yield). Mass spectra (ESI) *m*/*z*: 528.3 (M + H)^+^.

#### (*S*)-Ethyl 6-(2-(4′-Ethyl-2-hydroxy-[1,1′-biphenyl]-4-carboxamido)­propanamido)-4-oxo-1,4-dihydroquinoline-3-carboxylate (**L-33-M**)

Off-white powder (766 mg, 73% yield). Mass spectra (ESI) *m*/*z*: 528.3 (M + H)^+^.

### General Procedures for the Preparation of Compounds **L-2** to **L-33**

To a solution of compound **7a**–**f**, **8g**, **8h**, or **L-X-m** (1.0 mmol, 1.0 equiv) in a mixture of methanol (20 mL) and water (20 mL), KOH (10.0 mmol, 10.0 equiv) was added. The mixture was stirred at 60 °C for 16 h. It was then cooled to 0 °C and carefully acidified with 1 N HCl to reach a pH of around 1–2. The resulting precipitate was filtered and purified using HPLC, yielding the final product as an off-white solid with a yield of 68 to 90%.

#### (*S*)-6-(2-([1,1′-Biphenyl]-4-ylcarboxamido)­propanamido)-4-oxo-1,4-dihydroquinoline-3-carboxylic acid (**L-1**)

Brown powder (422 mg, 90% yield, purity > 95%, *ee* > 99%). ^1^H NMR (500 MHz, DMSO) δ 10.53 (s, 1H), 8.83 (d, *J* = 6.8 Hz, 1H), 8.78 (d, *J* = 6.8 Hz, 1H), 8.65 (d, *J* = 2.4 Hz, 1H), 8.10 (dd, *J* = 9.1, 2.4 Hz, 1H), 8.04 (d, *J* = 8.5 Hz, 2H), 7.84–7.77 (m, 3H), 7.76–7.70 (m, 2H), 7.50 (t, *J* = 7.6 Hz, 2H), 7.45–7.39 (m, 1H), 4.68–4.61 (m, 1H), 1.49 (d, *J* = 7.2 Hz, 3H). ^13^C NMR (126 MHz, DMSO) δ 177.99 (s), 172.03 (s), 166.55 (s), 166.08 (s), 144.05 (s), 142.89 (s), 139.15 (s), 137.34 (s), 135.40 (s), 132.67 (s), 129.04 (s), 128.30 (s), 128.08 (s), 126.88 (s), 126.41 (s), 126.19 (s), 124.95 (s), 120.41 (s), 113.21 (s), 107.07 (s), 50.13 (s), 17.61 (s). Mass spectra (ESI): 454.3 *m*/*z* [M – H]^−^. HRMS (ESI-TOF): *m*/*z* [M – H]^−^ calcd. For C_26_H_20_N_3_O_5_: 454.1403, found: 454.1413.

#### (*R*)-6-(2-([1,1′-Biphenyl]-4-ylcarboxamido)­propanamido)-4-oxo-1,4-dihydroquinoline-3-carboxylic acid (**L-2**)

Off-white powder (401 mg, 88% yield, purity > 95%, *ee* > 99%). ^1^H NMR (500 MHz, DMSO-*d*_6_) δ 10.53 (s, 1H), 8.82 (s, 1H), 8.78 (d, *J* = 6.8 Hz, 1H), 8.66 (d, *J* = 2.4 Hz, 1H), 8.11 (dd, *J* = 9.0, 2.4 Hz, 1H), 8.04 (d, *J* = 8.4 Hz, 2H), 7.86–7.68 (m, 5H), 7.49 (t, *J* = 7.6 Hz, 2H), 7.41 (t, *J* = 7.3 Hz, 1H), 4.66 (p, *J* = 7.0 Hz, 1H), 1.50 (d, *J* = 7.2 Hz, 3H). ^13^C NMR (126 MHz, DMSO-*d*_6_) δ 177.94 (s), 171.98 (s), 166.50 (s), 166.04 (s), 144.00 (s), 142.84 (s), 139.11 (s), 137.29 (s), 135.36 (s), 132.64 (s), 128.99 (s), 128.26 (s), 128.03 (s), 126.83 (s), 126.37 (s), 126.14 (s), 124.91 (s), 120.36 (s), 113.17 (s), 107.04 (s), 50.10 (s), 17.58 (s). Mass spectra (ESI) *m*/*z*: 454.3 (M – H)^−^. HRMS (ESI-TOF): *m*/*z* [M – H]^−^ calcd. for C_26_H_20_N_3_O_5_: 454.1403, found: 454.1414.

#### 6-(2-([1,1′-Biphenyl]-4-ylcarboxamido)­acetamido)-4-oxo-1,4-dihydroquinoline-3-carboxylic Acid (**L-3**)

Off-white powder (340 mg, 77% yield, purity > 95%). ^1^H NMR (500 MHz, DMSO-*d*_6_) δ 10.53 (s, 1H), 8.97 (t, *J* = 5.7 Hz, 1H), 8.82 (s, 1H), 8.62 (d, *J* = 2.3 Hz, 1H), 8.09 (dd, *J* = 9.0, 2.3 Hz, 1H), 8.02 (d, *J* = 8.3 Hz, 2H), 7.84–7.77 (m, 3H), 7.75 (d, *J* = 7.4 Hz, 2H), 7.50 (t, *J* = 7.6 Hz, 2H), 7.41 (t, *J* = 7.3 Hz, 1H), 4.15 (d, *J* = 5.7 Hz, 2H). ^13^C NMR (126 MHz, DMSO-*d*_6_) δ 178.42 (s), 168.85 (s), 167.00 (s), 166.85 (s), 144.50 (s), 143.38 (s), 139.59 (s), 137.63 (s), 135.86 (s), 133.16 (s), 129.50 (s), 128.52 (s), 127.34 (s), 127.01 (s), 126.54 (s), 125.44 (s), 120.92 (s), 113.55 (s), 107.54 (s), 43.93 (s). Mass spectra (ESI) *m*/*z*: 440.2 (M – H)^−^. HRMS (ESI-TOF): *m*/*z* [M – H]^−^ calcd. for C_25_H_18_N_3_O_5_: 440.1246, found: 440.1240.

#### (*S*)-6-(2-([1,1′-Biphenyl]-4-ylcarboxamido)-4-methylpentanamido)-4-oxo-1,4-dihydroquinoline-3-carboxylic Acid (**L-4**)

Off-white powder (421 mg, 83% yield, purity > 95%). ^1^H NMR (500 MHz, DMSO-*d*_6_) δ 10.59 (s, 1H), 8.83 (s, 1H), 8.73 (d, *J* = 7.6 Hz, 1H), 8.67 (d, *J* = 2.3 Hz, 1H), 8.12 (dd, *J* = 9.0, 2.3 Hz, 1H), 8.04 (d, *J* = 8.3 Hz, 2H), 7.87–7.75 (m, 3H), 7.73 (d, *J* = 7.8 Hz, 2H), 7.49 (t, *J* = 7.6 Hz, 2H), 7.40 (t, *J* = 7.3 Hz, 1H), 4.72 (ddd, *J* = 10.7, 7.6, 4.9 Hz, 1H), 1.92–1.85 (m, 1H), 1.83–1.74 (m, 1H), 1.70–1.60 (m, 1H), 0.96 (dd, *J* = 11.7, 6.5 Hz, 6H). ^13^C NMR (126 MHz, DMSO-*d*_6_) δ 177.93 (s), 171.94 (s), 166.53 (s), 166.30 (s), 144.05 (s), 142.88 (s), 139.14 (s), 137.23 (s), 135.46 (s), 132.71 (s), 128.99 (s), 128.29 (s), 128.02 (s), 126.84 (s), 126.39 (s), 126.17 (s), 124.91 (s), 120.42 (s), 113.26 (s), 107.05 (s), 52.94 (s), 24.59 (s), 23.04 (s), 21.42 (s). Mass spectra (ESI) *m*/*z*: 496.3 (M – H)^−^. HRMS (ESI-TOF): *m*/*z* [M – H]^−^ calcd. for C_29_H_26_N_3_O_5_: 496.1872, found: 496.1878.

#### (*S*)-6-(2-([1,1′-Biphenyl]-4-ylcarboxamido)-3-(4-chlorophenyl)­propanamido)-4-oxo-1,4-dihydroquinoline-3-carboxylic Acid (**L-5**)

Off-white powder (412 mg, 73% yield, purity > 95%). ^1^H NMR (500 MHz, DMSO-*d*_6_) δ 10.70 (s, 1H), 8.91 (d, *J* = 7.8 Hz, 1H), 8.84 (s, 1H), 8.66 (s, 1H), 8.09 (d, *J* = 8.2 Hz, 1H), 7.96 (d, *J* = 8.0 Hz, 2H), 7.88–7.67 (m, 5H), 7.54–7.33 (m, 7H), 4.90 (s, 1H), 3.25–3.10 (m, 2H). ^13^C NMR (126 MHz, DMSO-*d*_6_) δ 177.93 (s), 170.70 (s), 166.48 (s), 166.26 (s), 144.09 (s), 142.94 (s), 139.07 (s), 137.11 (s), 137.01 (s), 135.50 (s), 132.49 (s), 131.07 (s), 128.99 (s), 128.16 (s), 128.08 (s), 126.83 (s), 126.42 (s), 126.17 (s), 124.92 (s), 120.47 (s), 113.30 (s), 107.09 (s), 55.84 (s), 36.29 (s). Mass spectra (ESI) *m*/*z*: 564.2 (M – H)^−^. HRMS (ESI-TOF): *m*/*z* [M – H]^−^ calcd. for C_32_H_23_ClN_3_O_5_: 564.1326, found: 564.1331.

#### (*S*)-6-(2-([1,1′-Biphenyl]-4-ylcarboxamido)-3-(1*H*-indol-3-yl)­propanamido)-4-oxo-1,4-dihydroquinoline-3-carboxylic Acid (**L-6**)

Off-white powder (421 mg, 74% yield, purity > 95%). ^1^H NMR (500 MHz, DMSO-*d*_6_) δ 10.83 (s, 1H), 10.71 (s, 1H), 8.84 (s, 1H), 8.81 (d, *J* = 7.5 Hz, 1H), 8.69 (d, *J* = 1.7 Hz, 1H), 8.13 (dd, *J* = 9.0, 1.7 Hz, 1H), 7.98 (d, *J* = 8.2 Hz, 2H), 7.85–7.71 (m, 6H), 7.49 (t, *J* = 7.5 Hz, 2H), 7.40 (t, *J* = 7.2 Hz, 1H), 7.33 (d, *J* = 8.3 Hz, 2H), 7.06 (t, *J* = 7.4 Hz, 1H), 7.00 (t, *J* = 7.4 Hz, 1H), 4.95 (dd, *J* = 14.0, 8.0 Hz, 1H), 3.33–3.21 (m, 2H). ^13^C NMR (126 MHz, DMSO-*d*_6_) δ 177.96 (s), 171.38 (s), 166.51 (s), 166.12 (s), 144.05 (s), 142.86 (s), 139.08 (s), 137.20 (s), 136.03 (s), 135.46 (s), 132.62 (s), 128.99 (s), 128.20 (s), 128.04 (s), 127.20 (s), 126.83 (s), 126.37 (s), 126.31 (s), 124.90 (s), 123.87 (s), 123.87 (s), 120.92 (s), 120.36 (s), 118.61 (s), 118.22 (s), 113.41 (s), 111.31 (s), 110.09 (s), 107.06 (s), 55.30 (s), 27.42 (s). Mass spectra (ESI) *m*/*z*: 569.3 (M – H)^−^. HRMS (ESI-TOF): *m*/*z* [M – H]^−^ calcd. for C_34_H_25_N_4_O_5_: 569.1825, found: 569.1831.

#### (*S*)-6-(2-([1,1′-Biphenyl]-4-ylcarboxamido)-6-aminohexanamido)-4-oxo-1,4-dihydroquinoline-3-carboxylic Acid (**L-7**)

*Off-white powder* (398 mg, 78% yield, purity > 95%). ^1^H NMR (500 MHz, DMSO-*d*_6_) δ 10.66 (s, 1H), 8.81 (d, *J* = 7.5 Hz, 1H), 8.77 (s, 1H), 8.63 (d, *J* = 2.4 Hz, 1H), 8.08 (d, *J* = 8.4 Hz, 2H), 8.03 (dd, *J* = 9.1, 2.4 Hz, 1H), 7.81–7.71 (m, 5H), 7.48 (t, *J* = 7.7 Hz, 2H), 7.39 (t, *J* = 7.4 Hz, 1H), 4.67 (dd, *J* = 14.8, 7.4 Hz, 1H), 2.90–2.76 (m, 2H), 2.00–1.88 (m, 2H), 1.72–1.61 (m, 2H), 1.60–1.52 (m, 1H), 1.52–1.44 (m, 1H). ^13^C NMR (126 MHz, DMSO-*d*_6_) δ 176.02 (s), 171.70 (s), 169.05 (s), 166.84 (s), 147.39 (s), 143.36 (s), 141.23 (s), 139.61 (s), 136.63 (s), 133.24 (s), 129.49 (s), 128.82 (s), 128.52 (s), 127.37 (s), 127.32 (s), 127.06 (s), 126.86 (s), 125.16 (s), 113.36 (s), 107.52 (s), 55.02 (s), 39.01 (s), 31.36 (s), 27.10 (s), 23.32 (s). Mass spectra (ESI) *m*/*z*: 511.3 (M – H)^−^. HRMS (ESI-TOF): *m*/*z* [M – H]^−^ calcd. for C_29_H_27_N_4_O_5_: 511.1981, found: 511.1987.

#### (*S*)-6-(2-([1,1′-Biphenyl]-4-ylcarboxamido)-4-carboxybutanamido)-4-oxo-1,4-dihydroquinoline-3-carboxylic Acid (**L-8**)

Off-white powder (351 mg, 68% yield, purity > 95%). ^1^H NMR (500 MHz, DMSO-*d*_6_) δ 10.38 (s, 1H), 8.78 (s, 1H), 8.70 (d, *J* = 7.7 Hz, 1H), 8.60 (d, *J* = 2.2 Hz, 1H), 8.00 (d, *J* = 8.3 Hz, 3H), 7.80–7.70 (m, 5H), 7.49 (t, *J* = 7.6 Hz, 2H), 7.40 (t, *J* = 7.3 Hz, 1H), 4.52–4.45 (m, 1H), 2.57 (t, *J* = 7.4 Hz, 2H), 2.32–2.23 (m, 1H), 2.15–2.09 (m, 1H). ^13^C NMR (126 MHz, DMSO-*d*_6_) δ 177.51 (s), 173.49 (s), 170.97 (s), 166.90 (s), 166.06 (s), 144.39 (s), 142.81 (s), 139.09 (s), 137.19 (s), 136.19 (s), 132.70 (s), 128.98 (s), 128.09 (s), 128.03 (s), 126.80 (s), 126.38 (s), 125.69 (s), 124.89 (s), 120.95 (s), 112.73 (s), 106.96 (s), 52.32 (s), 33.09 (s), 26.22 (s). Mass spectra (ESI) *m*/*z*: 512.3 (M – H)^−^. HRMS (ESI-TOF): *m*/*z* [M – H]^−^ calcd. for C_28_H_22_N_3_O_7_: 512.1458, found: 512.1455.

#### (*S*)-6-(2-([1,1′-Biphenyl]-2-ylcarboxamido)­propanamido)-4-oxo-1,4-dihydroquinoline-3-carboxylic Acid (**L-9**)

Off-white powder (398 mg, 87% yield, purity > 95%). ^1^H NMR (500 MHz, DMSO-*d*_6_) δ 10.42 (s, 1H), 8.83 (s, 1H), 8.65 (d, *J* = 2.2 Hz, 1H), 8.57 (d, *J* = 7.0 Hz, 1H), 8.03 (dd, *J* = 9.0, 2.3 Hz, 1H), 7.81 (d, *J* = 9.0 Hz, 1H), 7.53 (t, *J* = 7.3 Hz, 2H), 7.48–7.40 (m, 4H), 7.33 (t, *J* = 7.5 Hz, 2H), 7.26 (t, *J* = 7.3 Hz, 1H), 4.45 (p, *J* = 7.0 Hz, 1H), 1.26 (d, *J* = 7.1 Hz, 3H). ^13^C NMR (126 MHz, DMSO-*d*_6_) δ 177.94 (s), 171.47 (s), 169.01 (s), 166.48 (s), 144.01 (s), 140.09 (s), 139.43 (s), 137.17 (s), 136.31 (s), 135.36 (s), 129.79 (s), 129.59 (s), 128.41 (s), 128.03 (s), 127.04 (s), 126.92 (s), 126.04 (s), 124.90 (s), 120.37 (s), 113.11 (s), 107.04 (s), 49.58 (s), 17.33 (s). Mass spectra (ESI) *m*/*z*: 454.2 (M – H)^−^. HRMS (ESI-TOF): *m*/*z* [M – H]^−^ calcd. for C_26_H_20_N_3_O_5_: 454.1403, found: 454.1410.

#### (*S*)-6-(2-([1,1′-Biphenyl]-3-ylcarboxamido)­propanamido)-4-oxo-1,4-dihydroquinoline-3-carboxylic Acid (**L-10**)

Off-white powder (374 mg, 82% yield, purity > 95%). ^1^H NMR (500 MHz, DMSO-*d*_6_) δ 10.54 (s, 1H), 8.89 (d, *J* = 6.8 Hz, 1H), 8.82 (d, *J* = 6.4 Hz, 1H), 8.66 (d, *J* = 2.4 Hz, 1H), 8.23 (t, *J* = 1.5 Hz, 1H), 8.10 (dd, *J* = 9.0, 2.4 Hz, 1H), 7.91 (d, *J* = 7.9 Hz, 1H), 7.87–7.84 (m, 1H), 7.81 (d, *J* = 9.0 Hz, 1H), 7.77 (dd, *J* = 8.2, 1.1 Hz, 2H), 7.58 (t, *J* = 7.7 Hz, 1H), 7.51 (dd, *J* = 10.6, 4.8 Hz, 2H), 7.41 (t, *J* = 7.4 Hz, 1H), 4.67 (p, *J* = 7.1 Hz, 1H), 1.50 (d, *J* = 7.2 Hz, 3H). ^13^C NMR (126 MHz, DMSO-*d*_6_) δ 177.95 (s), 171.98 (s), 166.49 (s), 166.26 (s), 144.02 (s), 140.10 (s), 139.54 (s), 137.28 (s), 135.37 (s), 134.46 (s), 129.56 (s), 128.95 (s), 127.75 (s), 126.86 (s), 126.79 (s), 126.16 (s), 125.70 (s), 124.91 (s), 120.37 (s), 113.18 (s), 107.04 (s), 50.12 (s), 17.57 (s). Mass spectra (ESI) *m*/*z*: 454.2 (M – H)^−^. HRMS (ESI-TOF): *m*/*z* [M – H]^−^ calcd. for C_26_H_20_N_3_O_5_: 454.1403, found: 454.1411.

#### (*S*)-4-Oxo-6-(2-(4-(thiophen-3-yl)­benzamido)­propanamido)-1,4-dihydroquinoline-3-carboxylic Acid (**L-11**)

Off-white powder (388 mg, 84% yield, purity > 95%). ^1^H NMR (500 MHz, DMSO-*d*_6_) δ 10.58 (s, 1H), 8.81 (d, *J* = 6.7 Hz, 1H), 8.78 (d, *J* = 6.8 Hz, 1H), 8.66 (d, *J* = 2.1 Hz, 1H), 8.11 (dd, *J* = 9.0, 2.3 Hz, 1H), 7.99 (d, *J* = 8.3 Hz, 2H), 7.83 (d, *J* = 9.0 Hz, 1H), 7.78 (d, *J* = 8.3 Hz, 2H), 7.66 (d, *J* = 3.2 Hz, 1H), 7.63 (d, *J* = 5.0 Hz, 1H), 7.18 (dd, *J* = 4.8, 3.8 Hz, 1H), 4.65 (p, *J* = 7.0 Hz, 1H), 1.49 (d, *J* = 7.2 Hz, 3H). ^13^C NMR (126 MHz, DMSO-*d*_6_) δ 177.92 (s), 171.96 (s), 166.48 (s), 165.74 (s), 143.89 (s), 142.25 (s), 137.29 (s), 136.38 (s), 135.35 (s), 132.46 (s), 128.68 (s), 128.46 (s), 126.76 (s), 126.12 (s), 124.89 (s), 120.34 (s), 113.13 (s), 107.00 (s), 50.10 (s), 17.58 (s). Mass spectra (ESI) *m*/*z*: 460.2 (M – H)^−^. HRMS (ESI-TOF): *m*/*z* [M – H]^−^ calcd. for C_24_H_18_N_3_O_5_S: 460.0967, found: 460.0972.

#### (*S*)-6-(2-(5-(2,4-Dichlorophenyl)­furan-2-carboxamido)­propanamido)-4-oxo-1,4-dihydroquinoline-3-carboxylic Acid (**L-12**)

Off-white powder (376 mg, 73% yield, purity > 95%). ^1^H NMR (500 MHz, DMSO-*d*_6_) δ 10.56 (s, 1H), 8.87 (d, *J* = 7.2 Hz, 1H), 8.83 (s, 1H), 8.65 (d, *J* = 2.3 Hz, 1H), 8.24 (d, *J* = 8.6 Hz, 1H), 8.10 (dd, *J* = 9.0, 2.3 Hz, 1H), 7.82 (d, *J* = 9.0 Hz, 1H), 7.77 (d, *J* = 2.0 Hz, 1H), 7.61 (dd, *J* = 8.6, 2.1 Hz, 1H), 7.33 (dd, *J* = 6.9, 3.7 Hz, 2H), 4.67 (p, *J* = 7.1 Hz, 1H), 1.51 (d, *J* = 7.1 Hz, 3H). ^13^C NMR (126 MHz, DMSO-*d*_6_) δ 177.93 (s), 171.54 (s), 166.48 (s), 157.35 (s), 149.78 (s), 147.00 (s), 144.03 (s), 137.16 (s), 135.42 (s), 133.45 (s), 130.40 (s), 130.20 (s), 129.92 (s), 127.80 (s), 126.49 (s), 126.18 (s), 124.90 (s), 120.38 (s), 115.97 (s), 113.29 (s), 113.07 (s), 107.05 (s), 49.38 (s), 17.64 (s). Mass spectra (ESI) *m*/*z*: 512.2 (M – H)^−^. HRMS (ESI-TOF): *m*/*z* [M – H]^−^ calcd. for C_24_H_16_Cl_2_N_3_O_6_: 512.0416, found: 512.0421.

#### (*S*)-6-(2-(1-Hydroxy-2-naphthamido)­propanamido)-4-oxo-1,4-dihydroquinoline-3-carboxylic Acid (**L-13**)

Off-white powder (312 mg, 70% yield, purity > 95%). ^1^H NMR (500 MHz, DMSO-*d*_6_) δ 10.63 (s, 1H), 9.16 (d, *J* = 6.5 Hz, 1H), 8.82 (s, 1H), 8.65 (d, *J* = 2.3 Hz, 1H), 8.26 (d, *J* = 8.3 Hz, 1H), 8.15–8.05 (m, 2H), 7.89 (d, *J* = 8.2 Hz, 1H), 7.82 (d, *J* = 9.0 Hz, 1H), 7.64 (t, *J* = 7.1 Hz, 1H), 7.55 (t, *J* = 7.6 Hz, 1H), 7.42 (d, *J* = 8.9 Hz, 1H), 4.73 (p, *J* = 7.0 Hz, 1H), 1.56 (d, *J* = 7.2 Hz, 3H). ^13^C NMR (126 MHz, DMSO-*d*_6_) δ 177.92 (s), 171.38 (s), 170.56 (s), 166.49 (s), 159.62 (s), 144.04 (s), 137.16 (s), 135.88 (s), 135.46 (s), 128.91 (s), 127.43 (s), 126.17 (s), 125.74 (s), 124.91 (s), 124.57 (s), 123.21 (s), 122.99 (s), 120.42 (s), 117.54 (s), 113.27 (s), 107.05 (s), 106.83 (s), 49.96 (s), 17.38 (s). Mass spectra (ESI) *m*/*z*: 444.2 (M – H)^−^. HRMS (ESI-TOF): *m*/*z* [M – H]^−^ calcd. for C_24_H_18_N_3_O_6_: 444.1196, found: 444.1191.

#### (*S*)-6-(2-(4-(4-Chlorophenoxy)­benzamido)­propanamido)-4-oxo-1,4-dihydroquinoline-3-carboxylic Acid (**L-14**)

Off-white powder (369 mg, 73% yield, purity > 95%). ^1^H NMR (500 MHz, DMSO-*d*_6_) δ 10.51 (s, 1H), 8.82 (s, 1H), 8.69 (d, *J* = 6.5 Hz, 1H), 8.64 (s, 1H), 8.09 (d, *J* = 8.1 Hz, 1H), 7.98 (d, *J* = 8.4 Hz, 2H), 7.81 (d, *J* = 8.9 Hz, 1H), 7.48 (d, *J* = 8.5 Hz, 2H), 7.10 (dd, *J* = 12.4, 8.7 Hz, 4H), 4.73 (p, *J* = 7.0 Hz, 1H), 1.47 (d, *J* = 7.0 Hz, 3H). ^13^C NMR (126 MHz, DMSO-*d*_6_) δ 177.92 (s), 171.98 (s), 166.49 (s), 165.57 (s), 159.05 (s), 154.66 (s), 144.00 (s), 137.27 (s), 135.36 (s), 130.04 (s), 129.86 (s), 128.97 (s), 127.97 (s), 126.12 (s), 124.90 (s), 121.05 (s), 120.36 (s), 117.59 (s), 113.15 (s), 107.03 (s), 50.07 (s), 17.56 (s). Mass spectra (ESI) *m*/*z*: 504.2 (M – H)^−^. HRMS (ESI-TOF): *m*/*z* [M – H]^−^ calcd. for C_26_H_19_ClN_3_O_6_: 504.0962, found: 504.0968.

#### (*S*)-4-Oxo-6-(2-(4-(pyridin-2-yl)­benzamido)­propanamido)-1,4-dihydroquinoline-3-carboxylic Acid (**L-15**)

Off-white powder (361 mg, 79% yield, purity > 95%). ^1^H NMR (500 MHz, DMSO-*d*_6_) δ 10.54 (s, 1H), 8.82 (dd, *J* = 6.5, 1.4 Hz, 2H), 8.75–8.68 (m, 1H), 8.65 (d, *J* = 2.3 Hz, 1H), 8.21 (d, *J* = 8.5 Hz, 2H), 8.11 (dd, *J* = 9.0, 2.4 Hz, 1H), 8.09–8.01 (m, 3H), 7.93 (td, *J* = 7.8, 1.8 Hz, 1H), 7.81 (d, *J* = 9.0 Hz, 1H), 7.47–7.37 (m, 1H), 4.66 (p, *J* = 7.1 Hz, 1H), 1.50 (d, *J* = 7.2 Hz, 3H). ^13^C NMR (126 MHz, DMSO-*d*_6_) δ 177.95 (s), 171.95 (s), 166.49 (s), 165.98 (s), 154.94 (s), 149.58 (s), 144.02 (s), 141.11 (s), 137.45 (s), 137.29 (s), 135.37 (s), 134.08 (s), 128.07 (s), 126.23 (s), 126.15 (s), 124.91 (s), 123.17 (s), 120.77 (s), 120.37 (s), 113.17 (s), 107.03 (s), 50.10 (s), 17.56 (s). Mass spectra (ESI) *m*/*z*: 455.3 (M – H)^−^. HRMS (ESI-TOF): *m*/*z* [M – H]^−^ calcd. for C_25_H_19_N_4_O_5_: 455.1355, found: 455.1351.

#### (*S*)-4-Oxo-6-(2-(4-(pyridin-3-yl)­benzamido)­propanamido)-1,4-dihydroquinoline-3-carboxylic Acid (**L-16**)

Off-white powder (342 mg, 75% yield, purity > 95%). ^1^H NMR (500 MHz, DMSO-*d*_6_) δ 10.59 (s, 1H), 9.10 (s, 1H), 8.87 (d, *J* = 6.8 Hz, 1H), 8.81 (d, *J* = 6.2 Hz, 1H), 8.72 (d, *J* = 4.3 Hz, 1H), 8.67 (d, *J* = 2.4 Hz, 1H), 8.44 (d, *J* = 8.0 Hz, 1H), 8.10 (dd, *J* = 8.8, 2.1 Hz, 3H), 7.93 (d, *J* = 8.5 Hz, 2H), 7.83 (d, *J* = 9.0 Hz, 1H), 7.74 (dd, *J* = 7.9, 5.1 Hz, 1H), 4.67 (p, *J* = 7.1 Hz, 1H), 1.50 (d, *J* = 7.2 Hz, 3H). ^13^C NMR (126 MHz, DMSO-*d*_6_) δ 178.45 (s), 172.44 (s), 167.01 (s), 166.28 (s), 146.89 (s), 145.83 (s), 144.45 (s), 139.13 (s), 137.80 (s), 136.14 (s), 135.89 (s), 134.26 (s), 128.96 (s), 127.35 (s), 126.64 (s), 125.52 (s), 125.42 (s), 120.88 (s), 113.66 (s), 107.54 (s), 50.66 (s), 18.09 (s). Mass spectra (ESI) *m*/*z*: 455.3 (M – H)^−^. HRMS (ESI-TOF): *m*/*z* [M – H]^−^ calcd. for C_25_H_19_N_4_O_5_: 455.1355, found: 455.1360.

#### (*S*)-4-Oxo-6-(2-(4-(pyridin-4-yl)­benzamido)­propanamido)-1,4-dihydroquinoline-3-carboxylic Acid (**L-17**)

Off-white powder (332 mg, 73% yield, purity > 95%). ^1^H NMR (500 MHz, DMSO-*d*_6_) δ 10.61 (s, 1H), 8.93 (d, *J* = 6.8 Hz, 1H), 8.84 (s, 2H), 8.81 (d, *J* = 3.9 Hz, 1H), 8.67 (d, *J* = 2.4 Hz, 1H), 8.25–8.00 (m, 7H), 7.83 (d, *J* = 9.0 Hz, 1H), 4.67 (p, *J* = 7.1 Hz, 1H), 1.51 (d, *J* = 7.2 Hz, 3H). ^13^C NMR (126 MHz, DMSO-*d*_6_) δ 177.98 (s), 171.91 (s), 166.54 (s), 165.63 (s), 150.29 (s), 146.40 (s), 143.97 (s), 138.31 (s), 137.32 (s), 135.43 (s), 135.32 (s), 128.60 (s), 127.36 (s), 126.17 (s), 124.95 (s), 122.79 (s), 120.41 (s), 113.20 (s), 107.07 (s), 50.23 (s), 17.61 (s). Mass spectra (ESI) *m*/*z*: 455.2 (M – H)^−^. HRMS (ESI-TOF): *m*/*z* [M – H]^−^ calcd. for C_25_H_19_N_4_O_5_: 455.1355, found: 455.1362.

#### (*S*)-4-Oxo-6-(2-(9-oxo-9*H*-fluorene-2-carboxamido)­propanamido)-1,4-dihydroquinoline-3-carboxylic Acid (**L-18**)

Off-white powder (356 mg, 74% yield, purity > 95%). ^1^H NMR (500 MHz, DMSO-*d*_6_) δ 10.51 (s, 1H), 8.94 (d, *J* = 6.7 Hz, 1H), 8.80 (s, 1H), 8.62 (d, *J* = 2.3 Hz, 1H), 8.22 (s, 1H), 8.17 (dd, *J* = 7.8, 1.4 Hz, 1H), 8.09 (dd, *J* = 9.0, 2.4 Hz, 1H), 7.91 (d, *J* = 7.8 Hz, 1H), 7.87 (d, *J* = 7.6 Hz, 1H), 7.79 (d, *J* = 9.0 Hz, 1H), 7.65 (t, *J* = 7.0 Hz, 2H), 7.43 (t, *J* = 7.5 Hz, 1H), 4.62 (p, *J* = 7.0 Hz, 1H), 1.48 (d, *J* = 7.2 Hz, 3H). ^13^C NMR (126 MHz, DMSO-*d*_6_) δ 192.49 (s), 177.94 (s), 171.83 (s), 166.50 (s), 165.11 (s), 146.41 (s), 144.02 (s), 143.11 (s), 137.25 (s), 135.54 (s), 135.39 (s), 134.97 (s), 134.64 (s), 133.83 (s), 133.19 (s), 130.13 (s), 126.17 (s), 124.90 (s), 124.10 (s), 122.88 (s), 121.89 (s), 121.04 (s), 120.36 (s), 113.23 (s), 107.03 (s), 50.24 (s), 17.47 (s). Mass spectra (ESI) *m*/*z*: 480.2 (M – H)^−^. HRMS (ESI-TOF): *m*/*z* [M – H]^−^ calcd. for C_27_H_18_N_3_O_6_: 480.1196, found: 480.1190.

#### (*S*)-6-(2-(4′-Fluoro-[1,1′-biphenyl]-4-ylcarboxamido)­propanamido)-4-oxo-1,4-dihydroquinoline-3-carboxylic Acid (**L-19**)

Off-white powder (381 mg, 80% yield, purity > 95%). ^1^H NMR (500 MHz, DMSO-*d*_6_) δ 10.53 (s, 1H), 8.82 (d, *J* = 5.7 Hz, 1H), 8.78 (d, *J* = 6.8 Hz, 1H), 8.65 (d, *J* = 2.3 Hz, 1H), 8.10 (dd, *J* = 9.0, 2.4 Hz, 1H), 8.03 (d, *J* = 8.4 Hz, 2H), 7.87–7.73 (m, 5H), 7.32 (t, *J* = 8.8 Hz, 2H), 4.66 (p, *J* = 7.1 Hz, 1H), 1.49 (d, *J* = 7.2 Hz, 3H). ^13^C NMR (126 MHz, DMSO-*d*_6_) δ 177.99 (s), 172.02 (s), 166.54 (s), 166.02 (s), 162.23 (d, *J* = 245.3 Hz), 144.04 (s), 141.81 (s), 137.33 (s), 135.63 (s), 135.40 (s), 132.63 (s), 128.95 (d, *J* = 8.2 Hz), 128.31 (s), 126.35 (s), 126.18 (s), 124.95 (s), 120.41 (s), 115.85 (d, *J* = 21.5 Hz), 113.20 (s), 107.08 (s), 50.13 (s), 17.61 (s). Mass spectra (ESI) *m*/*z*: 472.2 (M – H)^−^. HRMS (ESI-TOF): *m*/*z* [M – H]^−^ calcd. for C_26_H_19_FN_3_O_5_: 472.1309, found: 472.1313.

#### (*S*)-6-(2-(4′-Fluoro-[1,1′-biphenyl]-4-ylcarboxamido)­propanamido)-4-oxo-1,4-dihydroquinoline-3-carboxylic Acid (**L-20**)

Off-white powder (366 mg, 77% yield, purity > 95%). ^1^H NMR (500 MHz, DMSO-D_6_) δ 10.54 (s, 1H), 8.83 (s, 1H), 8.82 (d, *J* = 6.9 Hz, 1H), 8.66 (d, *J* = 2.3 Hz, 1H), 8.11 (dd, *J* = 9.0, 2.4 Hz, 1H), 8.05 (d, *J* = 8.4 Hz, 2H), 7.85 (d, *J* = 8.4 Hz, 2H), 7.82 (d, *J* = 9.0 Hz, 1H), 7.66–7.59 (m, 2H), 7.58–7.51 (m, 1H), 7.29–7.21 (m, 1H), 4.66 (p, *J* = 7.1 Hz, 1H), 1.50 (d, *J* = 7.2 Hz, 3H). ^13^C NMR (126 MHz, DMSO-D_6_) δ 177.93 (s), 171.94 (s), 166.48 (s), 165.89 (s), 162.67 (d, *J* = 243.5 Hz), 144.00 (s), 141.52 (d, *J* = 7.8 Hz), 141.33 (s), 137.28 (s), 135.35 (s), 133.17 (s), 130.93 (d, *J* = 8.4 Hz), 128.27 (s), 126.54 (s), 126.13 (s), 124.90 (s), 122.91 (s), 120.36 (s), 114.74 (d, *J* = 21.0 Hz), 113.66 (s), 113.48 (s), 113.16 (s), 107.03 (s), 50.10 (s), 17.56 (s). Mass spectra (ESI) *m*/*z*: 472.2 (M – H)^−^. HRMS (ESI-TOF): *m*/*z* [M – H]^−^ calcd. for C_26_H_19_FN_3_O_5_: 472.1309, found: 472.1311.

#### (*S*)-6-(2-(4′-Chloro-[1,1′-biphenyl]-4-ylcarboxamido)­propanamido)-4-oxo-1,4-dihydroquinoline-3-carboxylic Acid (**L-21**)

Off-white powder (403 mg, 82% yield, purity > 95%). ^1^H NMR (500 MHz, DMSO-D_6_) δ 10.54 (s, 1H), 8.83 (s, 1H), 8.81 (d, *J* = 6.8 Hz, 1H), 8.66 (d, *J* = 2.4 Hz, 1H), 8.11 (dd, *J* = 9.0, 2.4 Hz, 1H), 8.05 (d, *J* = 8.4 Hz, 2H), 7.83–7.77 (m, 6H), 7.56–7.54 (m, 2H), 4.67 (p, *J* = 7.1 Hz, 1H), 1.50 (d, *J* = 7.2 Hz, 3H). ^13^C NMR (126 MHz, DMSO-D_6_) δ 177.95 (s), 171.97 (s), 166.52 (s), 165.95 (s), 144.01 (s), 141.47 (s), 137.91 (s), 137.30 (s), 135.38 (s), 132.95 (s), 128.95 (s), 128.63 (s), 128.33 (s), 128.16 (s), 126.36 (s), 126.15 (s), 124.92 (s), 120.38 (s), 113.18 (s), 107.05 (s), 50.12 (s), 17.58 (s). Mass spectra (ESI) *m*/*z*: 488.2 (M – H)^−^. HRMS (ESI-TOF): *m*/*z* [M – H]^−^ calcd. for C_26_H_19_ClN_3_O_5_: 488.1013, found: 488.1020.

#### (*S*)-6-(2-(4′-Nitro-[1,1′-biphenyl]-4-ylcarboxamido)­propanamido)-4-oxo-1,4-dihydroquinoline-3-carboxylic Acid (**L-22**)

Off-white powder (386 mg, 77% yield, purity > 95%). ^1^H NMR (500 MHz, DMSO-D_6_) δ 10.59 (s, 1H), 8.88 (d, *J* = 6.8 Hz, 1H), 8.80 (d, *J* = 6.7 Hz, 1H), 8.66 (d, *J* = 2.3 Hz, 1H), 8.33–8.28 (m, 2H), 8.06 (d, *J* = 8.4 Hz, 2H), 8.02–7.98 (m, 2H), 7.89 (d, *J* = 8.4 Hz, 2H), 7.84 (dd, *J* = 11.4, 2.7 Hz, 1H), 7.81 (s, 1H), 4.72–4.63 (m, 1H), 1.51 (d, *J* = 7.2 Hz, 3H). ^13^C NMR (126 MHz, DMSO-D_6_) δ 177.93 (s), 171.92 (s), 166.51 (s), 165.76 (s), 146.97 (s), 145.51 (s), 143.89 (s), 140.38 (s), 137.30 (s), 135.37 (s), 134.03 (s), 128.62 (s), 128.46 (s), 127.67 (s), 127.30 (s), 126.12 (s), 124.91 (s), 120.34 (s), 113.17 (s), 107.03 (s), 50.18 (s), 17.59 (s). Mass spectra (ESI) *m*/*z*: 499.2 (M – H)^−^. HRMS (ESI-TOF): *m*/*z* [M – H]^−^ calcd. for C_26_H_19_N_4_O_7_: 499.1254, found: 499.1249.

#### (*S*)-6-(2-(4′-Hydroxy-[1,1′-biphenyl]-4-ylcarboxamido)­propanamido)-4-oxo-1,4-dihydroquinoline-3-carboxylic Acid (**L-23**)

Off-white powder (329 mg, 70% yield, purity > 95%). ^1^H NMR (500 MHz, DMSO-D_6_) δ 10.52 (s, 1H), 9.66 (s, 1H), 8.82 (d, *J* = 5.6 Hz, 1H), 8.72 (d, *J* = 6.8 Hz, 1H), 8.65 (d, *J* = 2.3 Hz, 1H), 8.10 (dd, *J* = 9.0, 2.4 Hz, 1H), 7.98 (d, *J* = 8.4 Hz, 2H), 7.81 (d, *J* = 9.0 Hz, 1H), 7.70 (d, *J* = 8.4 Hz, 2H), 7.58 (d, *J* = 8.6 Hz, 2H), 6.87 (d, *J* = 8.6 Hz, 2H), 4.65 (p, *J* = 7.1 Hz, 1H), 1.49 (d, *J* = 7.2 Hz, 3H). ^13^C NMR (126 MHz, DMSO-D_6_) δ 177.96 (s), 172.06 (s), 166.52 (s), 166.12 (s), 157.68 (s), 144.00 (s), 142.91 (s), 137.32 (s), 135.37 (s), 131.53 (s), 129.89 (s), 129.75 (s), 128.20 (s), 128.10 (s), 127.99 (s), 126.15 (s), 125.81 (s), 125.45 (s), 124.92 (s), 120.37 (s), 115.81 (s), 113.17 (s), 107.04 (s), 50.08 (s), 17.60 (s). Mass spectra (ESI) *m*/*z*: 470.2 (M – H)^−^. HRMS (ESI-TOF): *m*/*z* [M – H]^−^ calcd. for C_26_H_20_N_3_O_6_: 470.1352, found: 470.1359.

#### (*S*)-6-(2-(4′-Methoxy-[1,1′-biphenyl]-4-ylcarboxamido)­propanamido)-4-oxo-1,4-dihydroquinoline-3-carboxylic Acid (**L-24**)

Off-white powder (395 mg, 81% yield, purity > 95%). ^1^H NMR (500 MHz, DMSO-D_6_) δ 10.53 (s, 1H), 8.82 (d, *J* = 4.9 Hz, 1H), 8.74 (d, *J* = 6.7 Hz, 1H), 8.65 (d, *J* = 1.9 Hz, 1H), 8.10 (dd, *J* = 8.9, 1.7 Hz, 1H), 8.00 (d, *J* = 8.2 Hz, 2H), 7.81 (d, *J* = 9.0 Hz, 1H), 7.72 (dd, *J* = 22.0, 8.4 Hz, 4H), 7.05 (d, *J* = 8.6 Hz, 2H), 4.65 (p, *J* = 6.9 Hz, 1H), 3.81 (s, 3H), 1.49 (d, *J* = 7.1 Hz, 3H). ^13^C NMR (126 MHz, DMSO-D_6_) δ 177.94 (s), 172.02 (s), 166.50 (s), 166.06 (s), 159.35 (s), 143.99 (s), 142.49 (s), 137.30 (s), 135.35 (s), 131.86 (s), 131.35 (s), 128.22 (s), 127.98 (s), 126.13 (s), 125.70 (s), 124.91 (s), 120.36 (s), 114.42 (s), 113.15 (s), 107.03 (s), 55.19 (s), 50.07 (s), 17.58 (s). Mass spectra (ESI) *m*/*z*: 484.2 (M – H)^−^. HRMS (ESI-TOF): *m*/*z* [M – H]^−^ calcd. for C_27_H_22_N_3_O_6_: 484.1509, found: 484.1513.

#### (*S*)-6-(2-(3′-Methoxy-[1,1′-biphenyl]-4-ylcarboxamido)­propanamido)-4-oxo-1,4-dihydroquinoline-3-carboxylic Acid (**L-25**)

Off-white powder (374 mg, 77% yield, purity > 95%). ^1^H NMR (500 MHz, DMSO-D_6_) δ 10.53 (s, 1H), 8.83 (s, 1H), 8.78 (d, *J* = 6.8 Hz, 1H), 8.65 (d, *J* = 2.4 Hz, 1H), 8.10 (dd, *J* = 9.0, 2.4 Hz, 1H), 8.03 (d, *J* = 8.4 Hz, 2H), 7.81 (dd, *J* = 8.6, 6.7 Hz, 3H), 7.41 (t, *J* = 7.9 Hz, 1H), 7.35–7.23 (m, 2H), 6.98 (dd, *J* = 8.2, 2.0 Hz, 1H), 4.65 (p, *J* = 7.1 Hz, 1H), 3.84 (s, 3H), 1.49 (d, *J* = 7.2 Hz, 3H). ^13^C NMR (126 MHz, DMSO-D_6_) δ 177.94 (s), 171.98 (s), 166.49 (s), 166.01 (s), 159.77 (s), 144.01 (s), 142.71 (s), 140.60 (s), 137.29 (s), 135.36 (s), 132.75 (s), 130.06 (s), 128.18 (s), 126.48 (s), 126.13 (s), 124.91 (s), 120.37 (s), 119.16 (s), 113.65 (s), 113.16 (s), 112.29 (s), 107.03 (s), 55.16 (s), 50.09 (s), 17.56 (s). Mass spectra (ESI) *m*/*z*: 484.2 (M – H)^−^. HRMS (ESI-TOF): *m*/*z* [M – H]^−^ calcd. for C_27_H_22_N_3_O_6_: 484.1509, found: 484.1515.

#### (*S*)-6-(2-(4′-Ethyl-[1,1′-biphenyl]-4-ylcarboxamido)­propanamido)-4-oxo-1,4-dihydroquinoline-3-carboxylic Acid (**L-26**)

Off-white powder (381 mg, 79% yield, purity > 95%). ^1^H NMR (500 MHz, DMSO-D_6_) δ 10.52 (s, 1H), 8.82 (d, *J* = 6.4 Hz, 1H), 8.75 (d, *J* = 6.8 Hz, 1H), 8.64 (d, *J* = 2.3 Hz, 1H), 8.09 (dd, *J* = 9.1, 2.4 Hz, 1H), 8.01 (d, *J* = 8.4 Hz, 2H), 7.80 (d, *J* = 9.0 Hz, 1H), 7.76 (d, *J* = 8.4 Hz, 2H), 7.65 (d, *J* = 8.2 Hz, 2H), 7.32 (d, *J* = 8.2 Hz, 2H), 4.64 (p, *J* = 7.0 Hz, 1H), 2.65 (q, *J* = 7.6 Hz, 2H), 1.48 (d, *J* = 7.2 Hz, 3H), 1.20 (t, *J* = 7.6 Hz, 3H). ^13^C NMR (126 MHz, DMSO-D_6_) δ 177.99 (s), 172.05 (s), 166.54 (s), 166.10 (s), 144.07 (s), 143.82 (s), 142.85 (s), 137.34 (s), 136.54 (s), 135.40 (s), 132.37 (s), 128.46 (s), 128.27 (s), 126.81 (s), 126.14 (s), 124.95 (s), 120.41 (s), 113.18 (s), 107.06 (s), 50.13 (s), 27.82 (s), 17.61 (s), 15.58 (s). Mass spectra (ESI) *m*/*z*: 482.3 (M – H)^−^. HRMS (ESI-TOF): *m*/*z* [M – H]^−^ calcd. for C_28_H_24_N_3_O_5_: 482.1716, found: 482.1713.

#### (*S*)-6-(2-(4′-Isopropyl-[1,1′-biphenyl]-4-ylcarboxamido)­propanamido)-4-oxo-1,4-dihydroquinoline-3-carboxylic Acid (**L-27**)

Off-white powder (392 mg, 79% yield, purity > 95%). Mass spectra (ESI) *m*/*z*: 496.3 (M – H)^−^. HRMS (ESI-TOF): *m*/*z* [M – H]^−^ calcd. for C_29_H_26_N_3_O_5_: 496.1872, found: 496.1879.

#### (*S*)-6-(2-(4′-Ethyl-2-fluoro-[1,1′-biphenyl]-4-ylcarboxamido)­propanamido)-4-oxo-1,4-dihydroquinoline-3-carboxylic Acid (**L-28**)

Off-white powder (372 mg, 74% yield, purity > 95%). ^1^H NMR (500 MHz, DMSO-D_6_) δ 10.53 (s, 1H), 8.86 (d, *J* = 6.7 Hz, 1H), 8.83 (s, 1H), 8.65 (d, *J* = 2.3 Hz, 1H), 8.10 (dd, *J* = 9.0, 2.4 Hz, 1H), 7.88 (s, 1H), 7.86 (d, *J* = 1.8 Hz, 1H), 7.81 (d, *J* = 9.0 Hz, 1H), 7.65 (t, *J* = 8.0 Hz, 1H), 7.53 (d, *J* = 6.9 Hz, 2H), 7.35 (d, *J* = 8.2 Hz, 2H), 4.64 (p, *J* = 7.1 Hz, 1H), 2.67 (q, *J* = 7.6 Hz, 2H), 1.49 (d, *J* = 7.2 Hz, 3H), 1.22 (t, *J* = 7.6 Hz, 3H). ^13^C NMR (126 MHz, DMSO-D_6_) δ 177.92 (s), 171.76 (s), 166.47 (s), 164.76 (s), 158.67 (d, *J* = 246.3 Hz), 144.11 (s), 144.02 (s), 137.23 (s), 135.38 (s), 134.57 (d, *J* = 7.0 Hz), 131.59 (s), 130.96 (d, *J* = 13.5 Hz), 130.49 (s), 128.75 (s), 128.09 (s), 126.15 (s), 124.89 (s), 124.04 (s), 120.37 (s), 115.23 (d, *J* = 24.6 Hz), 113.20 (s), 107.03 (s), 50.18 (s), 27.86 (s), 17.51 (s), 15.48 (s). Mass spectra (ESI) *m*/*z*: 500.3 (M – H)^−^. HRMS (ESI-TOF): *m*/*z* [M – H]^−^ calcd. for C_28_H_23_FN_3_O_5_: 500.1622, found: 500.1629.

#### (*S*)-6-(2-(4′-Ethyl-3-fluoro-[1,1′-biphenyl]-4-ylcarboxamido)­propanamido)-4-oxo-1,4-dihydroquinoline-3-carboxylic Acid (**L-29**)

Off-white powder (385 mg, 77% yield, purity > 95%). ^1^H NMR (500 MHz, DMSO-D_6_) δ 10.56 (s, 1H), 8.83 (s, 1H), 8.66 (d, *J* = 2.4 Hz, 1H), 8.56 (dd, *J* = 6.8, 3.1 Hz, 1H), 8.09 (dd, *J* = 9.0, 2.4 Hz, 1H), 7.82 (d, *J* = 9.0 Hz, 1H), 7.76 (t, *J* = 8.0 Hz, 1H), 7.69 (d, *J* = 8.2 Hz, 2H), 7.65–7.59 (m, 2H), 7.34 (d, *J* = 8.2 Hz, 2H), 4.66 (p, *J* = 7.0 Hz, 1H), 2.66 (q, *J* = 7.6 Hz, 2H), 1.47 (d, *J* = 7.1 Hz, 3H), 1.21 (t, *J* = 7.6 Hz, 3H). ^13^C NMR (126 MHz, DMSO-D_6_) δ 177.93 (s), 171.43 (s), 166.47 (s), 163.30 (s), 159.89 (d, *J* = 249.0 Hz), 144.54 (d, *J* = 8.6 Hz), 144.42 (s), 144.06 (s), 137.14 (s), 135.43 (s), 135.19 (s), 130.87 (s), 128.47 (s), 126.81 (s), 126.10 (s), 124.91 (s), 122.12 (s), 121.48 (d, *J* = 14.2 Hz), 120.43 (s), 113.71 (d, *J* = 23.7 Hz), 113.17 (s), 107.06 (s), 49.87 (s), 27.79 (s), 17.87 (s), 15.48 (s). Mass spectra (ESI) *m*/*z*: 500.3 (M – H)^−^. HRMS (ESI-TOF): *m*/*z* [M – H]^−^ calcd. for C_28_H_23_FN_3_O_5_: 500.1622, found: 500.1631.

#### (*S*)-6-(2-(3-Chloro-4′-ethyl-[1,1′-biphenyl]-4-ylcarboxamido)­propanamido)-4-oxo-1,4-dihydroquinoline-3-carboxylic Acid (**L-30**)

Off-white powder (397 mg, 77% yield, purity > 95%). ^1^H NMR (500 MHz, DMSO-D_6_) δ 10.54 (s, 1H), 8.84 (d, *J* = 6.9 Hz, 2H), 8.68 (d, *J* = 2.4 Hz, 1H), 8.09 (dd, *J* = 9.0, 2.4 Hz, 1H), 7.83 (d, *J* = 9.0 Hz, 1H), 7.77 (d, *J* = 1.6 Hz, 1H), 7.70 (dd, *J* = 8.0, 1.7 Hz, 1H), 7.65 (d, *J* = 8.2 Hz, 2H), 7.59 (d, *J* = 8.0 Hz, 1H), 7.33 (d, *J* = 8.2 Hz, 2H), 4.63 (p, *J* = 7.1 Hz, 1H), 2.66 (q, *J* = 7.6 Hz, 2H), 1.44 (d, *J* = 7.1 Hz, 3H), 1.21 (t, *J* = 7.6 Hz, 3H). ^13^C NMR (126 MHz, DMSO-D_6_) δ 177.93 (s), 171.39 (s), 166.48 (s), 166.08 (s), 144.18 (s), 144.04 (s), 142.68 (s), 137.21 (s), 135.40 (s), 135.30 (s), 134.53 (s), 130.79 (s), 129.78 (s), 128.49 (s), 127.17 (s), 126.78 (s), 126.08 (s), 124.92 (s), 124.85 (s), 120.44 (s), 113.10 (s), 107.05 (s), 49.74 (s), 27.78 (s), 17.59 (s), 15.50 (s). Mass spectra (ESI) *m*/*z*: 516.2 (M – H)^−^. HRMS (ESI-TOF): *m*/*z* [M – H]^−^ calcd. for C_28_H_23_ClN_3_O_5_: 516.1326, found: 516.1341.

#### (*S*)-6-(2-(4′-Ethyl-3-methyl-[1,1′-biphenyl]-4-ylcarboxamido)­propanamido)-4-oxo-1,4-dihydroquinoline-3-carboxylic Acid (**L-31**)

Off-white powder (373 mg, 75% yield, purity > 95%). ^1^H NMR (500 MHz, DMSO-D_6_) δ 10.53 (s, 1H), 8.83 (s, 1H), 8.68 (d, *J* = 2.2 Hz, 1H), 8.61 (d, *J* = 6.7 Hz, 1H), 8.10 (dd, *J* = 9.0, 2.2 Hz, 1H), 7.83 (d, *J* = 9.0 Hz, 1H), 7.61 (d, *J* = 8.0 Hz, 2H), 7.56–7.46 (m, 3H), 7.31 (d, *J* = 8.0 Hz, 2H), 4.68–4.55 (m, 1H), 2.65 (q, *J* = 7.5 Hz, 2H), 2.44 (s, 3H), 1.44 (d, *J* = 7.1 Hz, 3H), 1.21 (t, *J* = 7.6 Hz, 3H). ^13^C NMR (126 MHz, DMSO-D_6_) δ 177.93 (s), 171.87 (s), 168.97 (s), 166.50 (s), 144.00 (s), 143.40 (s), 141.02 (s), 137.32 (s), 136.84 (s), 136.21 (s), 135.36 (s), 135.09 (s), 128.38 (s), 128.33 (s), 128.08 (s), 126.61 (s), 126.07 (s), 124.93 (s), 123.35 (s), 120.42 (s), 113.04 (s), 107.04 (s), 49.76 (s), 27.77 (s), 19.58 (s), 17.52 (s), 15.56 (s). Mass spectra (ESI) *m*/*z*: 496.3 (M – H)^−^. HRMS (ESI-TOF): *m*/*z* [M – H]^−^ calcd. for C_29_H_26_N_3_O_5_: 496.1872, found: 496.1879.

#### (*S*)-6-(2-(4′-Ethyl-3-hydroxy-[1,1′-biphenyl]-4-ylcarboxamido)­propanamido)-4-oxo-1,4-dihydroquinoline-3-carboxylic Acid (**L-32**)

Off-white powder (368 mg, 74% yield, purity > 95%). ^1^H NMR (500 MHz, DMSO-D_6_) δ 12.27 (s, 1H), 10.60 (s, 1H), 9.08 (d, *J* = 6.7 Hz, 1H), 8.83 (s, 1H), 8.65 (d, *J* = 2.4 Hz, 1H), 8.14–8.04 (m, 2H), 7.82 (d, *J* = 9.0 Hz, 1H), 7.62 (d, *J* = 8.2 Hz, 2H), 7.31 (d, *J* = 8.2 Hz, 2H), 7.24 (dd, *J* = 8.3, 1.7 Hz, 1H), 7.18 (d, *J* = 1.7 Hz, 1H), 4.71 (p, *J* = 7.0 Hz, 1H), 2.65 (q, *J* = 7.6 Hz, 2H), 1.51 (d, *J* = 7.1 Hz, 3H), 1.20 (t, *J* = 7.6 Hz, 3H). ^13^C NMR (126 MHz, DMSO-D_6_) δ 177.93 (s), 171.43 (s), 167.81 (s), 166.47 (s), 159.64 (s), 145.24 (s), 144.07 (s), 137.09 (s), 136.16 (s), 135.46 (s), 129.44 (s), 128.37 (s), 126.70 (s), 126.15 (s), 124.91 (s), 120.42 (s), 117.00 (s), 114.53 (s), 114.28 (s), 113.28 (s), 107.06 (s), 49.69 (s), 27.79 (s), 17.95 (s), 15.48 (s). Mass spectra (ESI) *m*/*z*: 498.4 (M – H)^−^. HRMS (ESI-TOF): *m*/*z* [M – H]^−^ calcd. for C_28_H_24_N_3_O_6_: 498.1665, found: 498.1671.

#### (*S*)-6-(2-(4′-Ethyl-2-hydroxy-[1,1′-biphenyl]-4-ylcarboxamido)­propanamido)-4-oxo-1,4-dihydroquinoline-3-carboxylic Acid (**L-33**)

Off-white powder (353 mg, 71% yield, purity > 95%). ^1^H NMR (500 MHz, DMSO-D_6_) δ 10.51 (s, 1H), 9.77 (s, 1H), 8.83 (s, 1H), 8.65 (d, *J* = 2.2 Hz, 1H), 8.63 (d, *J* = 6.9 Hz, 1H), 8.10 (dd, *J* = 9.0, 2.2 Hz, 1H), 7.82 (d, *J* = 9.0 Hz, 1H), 7.53–7.44 (m, 4H), 7.34 (d, *J* = 7.8 Hz, 1H), 7.26 (d, *J* = 8.1 Hz, 2H), 4.62 (p, *J* = 7.0 Hz, 1H), 2.64 (q, *J* = 7.6 Hz, 2H), 1.47 (d, *J* = 7.1 Hz, 3H), 1.21 (t, *J* = 7.6 Hz, 3H). ^13^C NMR (126 MHz, DMSO-D_6_) δ 177.90 (s), 171.97 (s), 166.51 (s), 166.25 (s), 154.08 (s), 144.04 (s), 142.53 (s), 137.27 (s), 135.42 (s), 135.05 (s), 133.88 (s), 130.57 (s), 129.82 (s), 128.97 (s), 127.37 (s), 126.09 (s), 124.91 (s), 120.42 (s), 118.40 (s), 115.47 (s), 113.12 (s), 107.02 (s), 49.99 (s), 27.88 (s), 17.60 (s), 15.63 (s). Mass spectra (ESI) *m*/*z*: 498.3 (M – H)^−^. HRMS (ESI-TOF): *m*/*z* [M – H]^−^ calcd. for C_28_H_24_N_3_O_6_: 498.1665, found: 498.1671.

### Synthesis of Compound **L-34**

Starting from 6-aminoquinolin-4­(1*H*)-one (**9**) and Fmoc-l-Ala-OH, the condensation reaction gave the Fmoc-protected intermediate **10** in the presence of HOBt, HBTU, and DIPEA in DMF. Deprotection of the Fmoc group was carried out by using a 20% piperidine-containing DMF solution at room temperature. Final compound **L-34** was obtained by condensing compound **11** with 4′-ethyl-3-hydroxy-[1,1′-biphenyl]-4-carboxylic acid in similar reaction conditions as the previous condensation reaction (Scheme S1).

#### (*S*)-(9*H*-Fluoren-9-yl)­methyl­(1-oxo-1-((4-oxo-1,4-dihydroquinolin-6-yl)­amino)­propan-2-yl)­carbamate (**10**)

Fmoc-l-Ala-OH (0.7 g, 2.25 mmol, 1.0 equiv), HOBt (0.39 g, 2.92 mmol, 1.3 equiv), and HBTU (1.11 g, 2.92 mmol, 1.3 equiv) were dissolved in dry dimethylformamide (DMF, 15 mL). The mixture was stirred at room temperature for 15 min. 6-Aminoquinolin-4­(1*H*)-one (0.32 g, 2.00 mmol, 0.89 equiv) and *N*,*N*-diisopropylethylamine (1.2 mL, 6.75 mmol, 3.0 equiv) were then added, and the resulting mixture was stirred at room temperature overnight. DMF was removed by rotary evaporator, and ethyl acetate and water were then added. The formed precipitate was collected by filtration and purified by column chromatography, eluting with a 10:1 v/v mixture of dichloromethane and methanol, to give the Fmoc-protected intermediate (**10**) as a light brown solid (0.89 g, 87% yield). ^1^H NMR (500 MHz, DMSO-D_6_) δ 8.32 (d, *J* = 21.3 Hz, 1H), 7.91–7.85 (m, 3H), 7.84– 7.77 (m, 3H), 7.76–7.70 (m, 1H), 7.50 (dd, *J* = 8.7, 2.6 Hz, 1H), 7.40 (t, *J* = 7.4 Hz, 2H), 7.33 (t, *J* = 7.4 Hz, 2H), 6.26 (s, 2H), 5.98–5.94 (m, 1H), 3.42 (q, *J* = 6.9 Hz, 1H), 1.21 (d, *J* = 6.9 Hz, 3H). Mass spectra (ESI) *m*/*z*: 454.3 (M + H)^+^.

#### (*S*)-2-Amino-*N*-(4-oxo-1,4-dihydroquinolin-6-yl)­propanamide (**11**)

The Fmoc-protected intermediate **10** (0.8 g, 1.76 mmol) was dissolved in DMF (16 mL). Piperidine (4 mL) was added, and the reaction mixture was stirred at room temperature for 1 h. Concentration in vacuo gave a brown solid, which was washed with ethyl acetate to afford the title compound (0.31 g, 76% yield). ^1^H NMR (500 MHz, DMSO-D_6_) δ 8.34 (d, *J* = 2.4 Hz, 1H), 7.89 (dd, *J* = 8.9, 2.5 Hz, 1H), 7.82 (d, *J* = 7.3 Hz, 1H), 7.48 (d, *J* = 8.9 Hz, 1H), 5.96 (d, *J* = 7.3 Hz, 1H), 3.42 (q, *J* = 6.9 Hz, 1H), 1.21 (d, *J* = 6.9 Hz, 3H). Mass spectra (ESI) *m*/*z*: 232.2 (M + H)^+^.

#### (*S*)-4′-Ethyl-3-hydroxy-*N*-(1-oxo-1-((4-oxo-1,4-dihydroquinolin-6-yl)­amino)­propan-2-yl)-[1,1′-biphenyl]-4-carboxamide (**L-34**)

4′-Ethyl-3-hydroxy-[1,1′-biphenyl]-4-carboxylic acid (100 mg, 0.41 mmol), HOBt (67 mg, 0.50 mmol), and HBTU (188 mg, 0.50 mmol) were dissolved in dry dimethylformamide (DMF, 5 mL). The mixture was stirred at room temperature for 15 min. (*S*)-2-amino-*N*-(4-oxo-1,4-dihydroquinolin-6-yl)­propanamide (95 mg, 0.41 mmol) and DIPEA (0.30 mL, 1.65 mmol) were then added, and the resulting mixture was stirred at room temperature overnight. DMF was removed by rotary evaporator, and ethyl acetate and water were then added. The formed precipitate was collected by filtration and washed with ethyl acetate to give the desired product L-34 (138 mg, 73% yield, purity > 95%). Mass spectra (ESI) *m*/*z*: 456.2 (M + H)^+^. HRMS (ESI-TOF): *m*/*z* [M + H]^+^ calcd. for C_27_H_26_N_3_O_4_: 456.1923, found: 456.1929.

### Synthesis of Compound **L-35**

Starting from 6-aminoquinolin-4­(1*H*)-one (**4**) and *tert*-butyl (*S*)-(1-oxopropan-2-yl)­carbamate, the reductive amination gave the Boc-protected intermediate **5i** in the presence of sodium cyanoborohydride in DMF. The Boc group was deprotected using a 20% trifluoroacetic acid solution in DMF at room temperature. Compound **7i** was obtained by coupling compounds **6i** with Biphenyl-4-carboxylic acid in DMF in the presence of HOBt, HBTU, and DIPEA. Finally, we performed a hydrolysis using an aqueous solution of potassium hydroxide (aq. KOH) in a mixture of methanol and water as solvent. This process yielded L-34 as the free acid after aqueous workup with HCl (Scheme S2).

#### (*S*)-Ethyl 6-((2-((*tert*-Butoxycarbonyl)­amino)­propyl)­amino)-4-oxo-1,4-dihydroquinoline-3-carboxylate (**5i**)

To a solution of compound **4** (200 mg, 0.86 mmol, 1.0 equiv) in dimethylformamide (DMF, 20 mL), was added *tert*-butyl (*S*)-(1-oxopropan-2-yl)­carbamate (150 mg, 0.86 mmol, 1.0 equiv), and the mixture was stirred at room temperature for 1 h. Then, sodium cyanoborohydride (109 mg, 1.72 mmol, 2.0 equiv) was slowly added to the mixture, which was stirred at room temperature. After stirring for 12 h, the solvent was removed, yielding a solid residue, which was then washed with methanol (20 mL) to afford the desired product (271 mg, 81% yield). Brown powder; Mass spectra (ESI) *m*/*z*: 390.5 (M + H)^+^.

#### (*S*)-Ethyl 6-((2-Aminopropyl)­amino)-4-oxo-1,4-dihydroquinoline-3-carboxylate (**6i**)

To a solution of compound **5i** (250 mg, 0.64 mmol) in dimethylformamide (DMF, 10 mL), was added trifluoroacetic acid (2.0 mL). This mixture was stirred at room temperature for 6 h. After concentrating the mixture in vacuo, a brown solid was obtained. This solid was washed with ethyl acetate, yielding the target compound as a brown powder (159 mg, 86% yield). Mass spectra (ESI) *m*/*z*: 290.2 (M + H)^+^.

#### (*S*)-Ethyl 6-((2-([1,1′-biphenyl]-4-carboxamido)­propyl)­amino)-4-oxo-1,4-dihydroquinoline-3-carboxylate (**7i**)

Biphenyl-4-carboxylic acid (100 mg, 0.50 mmol, 1.0 equiv), HOBt (75 mg, 0.56 mmol, 1.12 equiv), and HBTU (210 mg, 0.56 mmol, 1.12 equiv) were dissolved in dry DMF (10 mL) and stirred at room temperature for 15 min. Then, compound **6i** (146 mg, 0.50 mmol, 1.0 equiv) and *N*,*N*-diisopropylethylamine (0.27 mL, 1.51 mmol, 3.0 equiv) were added. The mixture was stirred overnight at room temperature. After removing DMF with a rotary evaporator and adding ethyl acetate and water, the precipitate formed was filtered and washed with ethyl acetate, yielding intermediates **7i** as a brown powder (185 mg, 78% yield). Mass spectra (ESI) *m*/*z*: 470.3 (M + H)^+^.

#### (*S*)-6-((2-([1,1′-Biphenyl]-4-ylcarboxamido)­propyl)­amino)-4-oxo-1,4-dihydroquinoline-3-carboxylic Acid (**L-35**)

To a solution of compound **7i** (100 mg, 0.21 mmol, 1.0 equiv) in a mixture of methanol (10 mL) and water (10 mL), KOH (5.0 mmol, 23.8 equiv) was added. The mixture was stirred at 60 °C for 16 h. It was then cooled to 0 °C and carefully acidified with 1 N HCl to reach a pH of around 1–2. The resulting precipitate was filtered and purified using HPLC, yielding the final product as an off-white solid. (71 mg, 75% yield, purity > 95%). ^1^H NMR (500 MHz, DMSO-D_6_) δ 8.61 (d, *J* = 6.8 Hz, 1H), 8.39 (d, *J* = 8.0 Hz, 1H), 7.97 (d, *J* = 8.4 Hz, 2H), 7.75 (d, *J* = 8.4 Hz, 2H), 7.73–7.67 (m, 2H), 7.60 (d, *J* = 9.0 Hz, 1H), 7.48 (t, *J* = 7.6 Hz, 2H), 7.40 (t, *J* = 7.4 Hz, 1H), 7.34 (dd, *J* = 9.0, 2.6 Hz, 1H), 7.28 (d, *J* = 2.5 Hz, 1H), 4.37–4.28 (m, 1H), 3.37 (dd, *J* = 13.1, 6.8 Hz, 1H), 3.23 (dd, *J* = 13.1, 6.5 Hz, 1H), 1.27 (d, *J* = 6.7 Hz, 3H). ^13^C NMR (126 MHz, DMSO-D_6_) δ 177.16 (s), 167.14 (s), 165.67 (s), 147.47 (s), 142.59 (s), 140.85 (s), 139.17 (s), 133.42 (s), 130.96 (s), 128.98 (s), 128.00 (s), 126.81 (s), 126.32 (s), 126.11 (s), 122.17 (s), 120.51 (s), 106.15 (s), 100.90 (s), 47.83 (s), 44.46 (s), 18.37 (s). Mass spectra (ESI) *m*/*z*: 440.3 (M – H)^−^. HRMS (ESI-TOF): *m*/*z* [M – H]^−^ calcd. for C_26_H_22_N_3_O_4_: 440.1610, found: 440.1617.

### Synthesis of Compound **L-36**

Compound **L-19** was synthesized following the same process previously used for the **L-1** analogues.

#### (*S*)-Ethyl 6-(2-(4-Cyclohexylbenzamido)­propanamido)-4-oxo-1,4-dihydroquinoline-3-carboxylate (**L-36-M**)

Off-white powder (764 mg, 78% yield). Mass spectra (ESI) *m*/*z*: 490.3 (M + H)^+^.

#### (*S*)-6-(2-(4-Cyclohexylbenzamido)­propanamido)-4-oxo-1,4-dihydroquinoline-3-carboxylic Acid (**L-36**)

Off-white powder (367 mg, 80% yield, purity > 95%). ^1^H NMR (500 MHz, DMSO-D_6_) δ 10.48 (s, 1H), 8.82 (s, 1H), 8.64 (d, *J* = 2.3 Hz, 1H), 8.60 (d, *J* = 6.8 Hz, 1H), 8.09 (dd, *J* = 9.0, 2.4 Hz, 1H), 7.85 (d, *J* = 8.2 Hz, 2H), 7.80 (d, *J* = 9.0 Hz, 1H), 7.32 (d, *J* = 8.2 Hz, 2H), 4.61 (p, *J* = 7.1 Hz, 1H), 2.57 (dd, *J* = 15.7, 7.1 Hz, 1H), 1.85–1.78 (m, 4H), 1.46 (d, *J* = 7.2 Hz, 3H), 1.41–1.23 (m, 6H). ^13^C NMR (126 MHz, DMSO-D_6_) δ 177.92 (s), 172.01 (s), 166.48 (s), 166.31 (s), 151.08 (s), 144.00 (s), 137.28 (s), 135.34 (s), 131.51 (s), 127.64 (s), 126.42 (s), 126.12 (s), 124.89 (s), 120.35 (s), 113.14 (s), 107.01 (s), 49.98 (s), 43.63 (s), 33.68 (s), 26.22 (s), 25.49 (s), 17.57 (s). Mass spectra (ESI) *m*/*z*: 460.3 (M – H)^−^. HRMS (ESI-TOF): *m*/*z* [M – H]^−^ calcd. for C_26_H_26_N_3_O_5_: 460.1872, found: 460.1879.

### Protein Expression

Expression and purification of His-tagged PTPN22 were performed as previously described.^71^ PTPN22 was expressed in BL21 *E. coli* cells. Protein expression was induced by 0.5 mM IPTG and harvested after overnight incubation at 18 °C. The cells were pelleted by centrifugation at 6000 rpm and 4 °C for 15 min, and then resuspended in 40 mL of 20 mM Tris buffer (pH 8.0) containing 500 mM NaCl and 5 mM imidazole. The suspension was lysed by sonication at 0 °C for 30 min in the presence of 1 mM PMSF. The bacterial lysate was centrifuged at 15,000 rpm at 4 °C for 30 min. The supernatant was collected and incubated with 1 mL of Ni-NTA beads washed with the same 20 mM Tris buffer by end-overend rotation for 1.5 h at 4 °C. The mixture was flowed through a gravity flow column, and the beads were washed with 20 mM Tris buffer (pH 8.0), 500 mM NaCl, and 20 mM imidazole. PTPN22 was eluted with 20 mM Tris buffer, pH 8.0, with 500 mM NaCl and 300 mM imidazole. PTPN22 was concentrated by centrifugation and further purified using a Sephadex G75 column. Purified PTPN22 was stored in 20 mM Tris buffer, pH 8.0, with 150 mM NaCl and 1 mM DTT. Other PTPs for selectivity, including SHP-2 PTP domain, PTP1B, PTP-MEG2, TCPTP, STEP, PTP–PEST, PTPα-D1D2, PTPε, PTPγ, PPK, Laforin, CDC14A, CD45-D1D2, and LWM-PTP, were expressed and purified using the same method.

### IC_50_ Determination and *K*_*i*_ Determination

The PTP kinetic assay was modified from previous reported protocols.^43^ PTP activity was assayed using *para*-nitrophenyl phosphate (*p*NPP, Thermo Scientific catalog#PI34045) as a substrate in 3,3-dimethylglutaric acid (DMG) buffer (50 mM DMG, pH 7.0, 1 mM EDTA, 18 mM NaCl) at 25 °C. To determine the IC_50_ values, the assays were performed in 96-well plates (Corning Costar 3915). The reaction was initiated by adding the enzyme (final concentration, 20 nM) to a reaction mixture containing pNPP (final concentration corresponding to *K*_m_) and various inhibitor concentrations. The final reaction volume is 300 μL. The reaction was allowed to proceed for 60 min with PTPN22 (and 3–60 min with the other PTPs). All reaction times were within the linear range with maximal signal responses, and then quenched by the addition of 50 μL of 5 N NaOH solution. The OD405 was measured using a CLARIOstar Plus Microplate Spectrophotometer (BMG Labtech). Data were fitted using Prism GraphPad 9.2.3.

To evaluate the selectivity, the IC_50_ of compounds against SHP-2 PTP domain, PTP1B, PTP-MEG2, TCPTP, STEP, PTP–PEST, PTPα-D1D2, PTPε, PTPγ, PPK, Laforin, CDC14A, CD45-D1D2, and LWM-PTP were tested under the same conditions as used for PTPN22, except that the final enzyme concentration was 20 nM and *p*NPP was at *K*_m_ concentrations.

All assays were performed with and without 0.01% Triton to eliminate aggregation effects. Each assay was independently repeated three times, and the results were averaged.

To determine the mode of inhibition, the reactions were initiated by adding the enzyme (final concentration, 20 nM) to reaction mixtures (final volume, 300 μL) containing concentrations ranging from 10 times above to 10 times below the *K*_m_ value, with varying concentrations of the inhibitor. The reaction was allowed to proceed for 60 min, then quenched by the addition of 50 μL of 5.0 M NaOH. The OD405 was measured using a CLARIOstar Plus Microplate Spectrophotometer. Data were fitted using Prism GraphPad 9.2.3.

### Molecular Docking Studies

Molecular docking studies were performed on a previously reported PTPN22 crystal structure (PDB 4J51) using the Maestro suite version 2021-3 (Schrödinger, LLC, New York, NY). Chain B was deleted, and Chain A was prepared using the Protein Preparation Workflow at pH 7.4 ± 1.0. Missing side chains and loops were filled using Prime. Hydrogen bond assignments were optimized using PROPKA, and the catalytic cysteine (C227) was manually ionized via the 3D Builder module. Lastly, a restrained minimization was performed to a 0.30 Å RMSD using the OPLS4 force field, and all waters were removed. The receptor grid was generated using the Receptor Grid Generation module and centered on the cognate ligand. Ligand preparation was conducted using the LigPrep module with the OPSL4 force field. Ionization states were generated using Epik at pH 7.4 ± 1.0. Docking was performed using Glide in the standard precision (SP) mode using default settings. The top 10 poses were retained for visual evaluation and assessed for their agreement with known SAR.

### IDO1 and Arginase Activity Assay

The relative enzymatic activity of IDO1 and Arginase was evaluated in MC38 murine colon carcinoma and HEK293 cell lysates following treatment with either DMSO or 5 μM of **L-32**. IDO1 activity was quantified using the Indoleamine 2,3-Dioxygenase 1 Activity Assay Kit (Abcam, Cat# ab235936), measuring the fluorometric conversion of tryptophan to *N*-formylkynurenine (NFK), which is subsequently hydrolyzed to kynurenine. Arginase activity was determined using the Arginase Activity Assay Kit (Sigma-Aldrich, Cat# MAK112), which utilizes a coupled enzymatic reaction in which Arginase catalyzes the conversion of Arginine to urea and ornithine. The urea produced is then measured through a chromogenic reaction. Results are expressed as relative activity (%) normalized by DMSO control and represent the mean ± SD of 3 independent replicates.

### *In Vitro* Kinase Assay

The relative kinase activity were performed using recombinant human SRC and CSK enzymes (20 nM final concentration). Reactions were initiated in kinase buffer (50 mM Tris-HCl, pH 7.5, 10 mM MgCl_2_) containing 10 μM ATP and the specific protein substrates: recombinant SHP2 (5 μM) for SRC and recombinant SRC (5 μM) for CSK. **L-32** was added at final concentrations of 1, 5, and 10 μM. Following a 30 min incubation at 30 °C, the reaction was stopped and the release of inorganic phosphate was quantified using BIOMOL Green reagent (Enzo Life Sciences, Cat# BML-AK111). Absorbance was measured at 620 nm using a microplate reader. Enzymatic activity is expressed relative to DMSO control and represent the mean ± SD of 3 independent replicates.

### Cytotoxicity Assay

The CCK-8 assay was adapted and used to determine the cell viability of cells exposed to corresponding compounds as previously reported.^72^ The number of cells in the prepared cell suspension was counted using the cell counting plate. Afterward, the cells were diluted and seeded in a 96-well culture plate at a concentration of 5000 cells (100 μL) per well. After 24 h, the cells were treated with various compounds (the control group was treated only with DMSO) for an additional 24 h. Then, CCK-8 reagents (Fisher Scientific, Cat#50-190-5564) were added to the well. The cells were incubated for 2–4 h at 37 °C in a humidified atmosphere of 5% CO_2_. The OD460 was measured using a CLARIOstar Plus Microplate Spectrophotometer. Data were fitted using Prism GraphPad 9.2.3.

### Extended Kinetic Solubility Assay

The extended kinetic solubility of compound **L-32** was determined by diluting 10 μL of a 10 mM DMSO stock solution into 990 μL of PBS buffer (pH 7.4) to afford a final concentration of 100 μM. The resulting suspension was incubated at 25 °C with stirring at 500 rpm for 24 h to allow equilibration. The samples were then centrifuged for 10 min to remove any undissolved material.

The concentration of dissolved compound in the supernatant was quantified by HPLC analysis, using calibration against a standard curve generated from known concentrations of **L-32**.

### Cell Permeability Studies

The membrane permeability of **L-32** was evaluated using a Corning BioCoat Precoated PAMPA Plate System according to the manufacturer’s instructions. Briefly, compound solutions were prepared by diluting 10 mM DMSO stock solutions in PBS to a final concentration of 200 μM. The compound solution (300 μL per well) was added to the donor (receiver) plate, while PBS (200 μL per well) was added to the acceptor wells of the precoated filter plate. The filter plate was then assembled with the donor plate and incubated at room temperature for 5 h without agitation.

At the end of the incubation, the plates were separated, and aliquots (150 μL) from both donor and acceptor wells were collected for analysis. Compound concentrations in both compartments were quantified by LC–MS.

The apparent permeability (*P*_e_, cm/s) was calculated using the following equations:

$$P_{e}=\left(A/t\right)\times \ln\left[1-\left(C_{A}\left(t\right)/C_{\mathrm{eq}}\right)\right]$$

$$C_{\mathrm{eq}}=\left[C_{D}\left(t\right)\times V_{D}+C_{A}\left(t\right)\times V_{A}\right]/\left(V_{D}+V_{A}\right)$$

where *A* is the filter area (0.3 cm^2^), *V*_D_ is the donor well volume (0.3 mL), *V*_A_ is the acceptor well volume (0.2 mL), *t* is the incubation time (s), and *C*_D_(*t*) and *C*_A_(*t*) are the compound concentrations in the donor and acceptor wells at time *t*, respectively.

### *In Vitro* Evaluation of **L-32** Metabolic Stability

The metabolic stability of **L-32** was evaluated in mouse liver microsomes (MLM), and the intrinsic clearance (Cl_int_) and *in vitro* half-life (*t*_1/2_) were determined based on the time-dependent depletion of the parent compound. Metabolic reactions were initiated in the presence of NADPH as a cofactor.

Briefly, **L-32** (final concentration: 300 μM) was preincubated with active MLM at 37 °C for 10 min in the absence of NADPH to allow equilibration. The metabolic reaction was then initiated by the addition of NADPH (final concentration: 10 mM). At designated time points (0, 5, 10, 15, 30, 45, and 60 min), aliquots were collected and the reactions were quenched by the addition of acetonitrile (ACN). Samples were subsequently centrifuged at 5500 rpm for 5 min to precipitate proteins, and the resulting supernatants were analyzed by HPLC–MS/MS.

The elimination rate constant (*k*_el_) was determined from the slope of the linear regression of ln­(AUC) versus time. The *in vitro* half-life (*t*_1/2_) and intrinsic clearance (Cl_int_) were calculated using the following equations:

$$t_{1/2}=0.693/k_{\mathrm{el}}$$

$${\mathrm{Cl}}_{\mathrm{int}}=\left(0.693/t_{1/2}\right)\times \left(\mathrm{\mu L}\;\mathrm{incubation}/\mathrm{mg}\;\mathrm{microsomes}\right)$$

### Cellular Assay

Jurkat cells were grown in RPMI-1640 medium supplemented with 10% FBS, penicillin (50 units/mL), and streptomycin (50 μg/mL) in a 37 °C incubator containing 5% CO_2_. To determine the cellular activity of selected compounds, Jurkat cells were treated with 10 μM of **L-26**, **L-29**, **L-32**, and **L-34** for 1 h, or various concentrations (1, 5, and 10 μM) of **L-32** and 10 μM of **L-34** for 1 h, then cells were stimulated with 2.5 μg/mL anti-CD3 antibody (OKT3) for 10 min. For Western blot analysis, protein samples were separated by SDS-PAGE, transferred to nitrocellulose membranes, and incubated overnight at 4 °C with corresponding primary antibodies diluted in 5% BSA in phosphate-buffered saline with Tween (PBST). SuperSignal West Pico PLUS (PI34580; Thermo Scientific) was used to visualize the antibodies in the bioanalytical imaging system (Azure Biosystems c500). *Anti*-pLCK (Y394) (70,926) and *anti-p*-ERK1/2 (T202/Y204) (4370) were purchased from Cell Signaling Technology. *Anti*-VCP (sc-57492) and anti-ACTIN (sc-8432) antibodies were purchased from Santa Cruz Biotechnology.

### Animal Study

All the *in vivo* studies were performed under an animal protocol (1511001324) approved by the Institutional Animal Care & Use Committee of Purdue University. Mice are typically housed in groups of five maximum per cage, in 12 h light/dark cycles, and receive food and water ad libitum. For the MC38 syngeneic tumor study, 12-week-old in-house bred C57BL/6J mice (both males and females) were subcutaneously injected with 10^6 MC38 cells in the right and left flanks to induce tumor growth. When tumors reached an average volume of 100 mm^3^, mice were tumor size-matched and randomly assigned to different experimental groups for experiments (groups were indicated in figure legend). Mice were intraperitoneally injected twice daily with saline or 10 mg/kg **L-1**/**L-32** for five consecutive days per week with or without PD-1 antibody (Bioxcell Catalog #BE0146) at a dose of 10 mg/kg twice weekly for 2 weeks. For the MC38 xenograft tumor study, 12-week-old NRG mice (females) were subcutaneously injected with 10^6 MC38 cells in the right and left flanks to induce tumor growth. When tumors reached an average volume of 50 mm^3^, mice were tumor size-matched and randomly assigned to different groups indicated in figure legend. Mice were intraperitoneally injected daily with saline or 10 mg/kg **L-32** for 2 weeks. Tumor volume was measured twice a week using the equation: *V* (mm^3^) = (length × width^2^)/2. At the end of the experiments, the mice were euthanized, and tumors were collected for IHC analysis using an anti-CD3 antibody (a T cell marker). For the pharmacokinetic investigation, the compound’s concentration in the blood over time was determined by collecting blood samples using the tail-clip method at 0.5, 1, 2, 3, 6, and 24 h postinjection of the compound. The study for each compound or each route was conducted on three inbred C57BL/6J mice (8–10 weeks old; ∼25 g each), including two males and one female. Each mouse was administered a single IP, IV or PO dose of 10 mg/kg, formulated as a solution with 10% (v/v) DMSO and 5% (v/v) Cremophor-EL in PBS. The volume of each injection was approximately 100 μL, based on the mouse’s weight. Blood samples (50 μL) were collected at each time point and centrifuged to get the serum. The serum (10 μL) was then mixed with acetonitrile (20 μL) and centrifuged. The supernatant was collected and analyzed by Liquid Chromatography/Mass Spectrometry. The Liquid Chromatography/Mass Spectrometry (LC/MS) analysis was carried out on an Agilent 1260 analytic HPLC system and an Agilent 6470 Triple Quadrupole MS detector, equipped with a Kinetex 2.6 μm C18 column (3 mm × 50 mm), eluted with 0–100% MeOH-H_2_O with 0.1% (w/v) formic acid at 0.7 mL/min flow-rate (gradient method: 1.2 min 0–10% MeOH linear-gradient, 1.5 min 10–90% MeOH linear-gradient, followed by 1.3 min 90–100% MeOH, followed by 2.5 min 100% MeOH), MS detector were set at single ion mode (SIM), monitoring the negative charge according to the compound molecular weight. The pharmacokinetic parameters were calculated in GraphPad Prism 6.

## Supplementary Material







## Data Availability

All data associated with this study are available in the published article and its Supporting Information.

## Notes

L-32_4J51 (PDB)
Detergent assay of compound **L-32**; selected kinase and protein activity of **L-32**; pharmacokinetic profile and metabolic stability of **L-32**; cytotoxicity assay of compound **L-1** and **L-32**; *in vivo* antitumor efficacy study of **L-1** and **L-32**; IHC staining for T cell infiltration into MC38 tumors; synthesis scheme of compound **L-34** and **L-35**; ^1^H and ^13^C NMR spectra for the final compounds (**L-1** to **L-36**); and LC/MS traces and HRMS data for compounds **L-26**, **L-29**, and L-32 (PDF)
Molecular formula strings (CSV)

## Abbreviations Used

AUC: area under concentration
DCM: dichloromethane
DIEA: *N,N*-disisopropylethylamine
DMF: *N,N*-dimethylformamide
DMSO: dimethyl sulfoxide
dr: diastereomeric ratio
HBTU: Hexafluorophosphate Benzotriazole Tetramethyl Uronium
HOBt: Hydroxybenzotriazole
*p*NPP: *p*-nitrophenyl phosphate
PTP: protein tyrosine phosphatase
QCA: quinoline-3-carboxylic acid
