# Supplementary material for: A Potent and Selective Quinolone-Based PTPN22 Inhibitor with Improved Immunotherapeutic Activity
Source: J Med Chem. 2026 Jul 2;69(14):16401–27. doi: 10.1021/acs.jmedchem.5c03467 (PMC13403317; doi:10.1021/acs.jmedchem.5c03467)

Supporting Information

A Potent and Selective Quinolone-Based PTPN22 Inhibitor with Improved Immunotherapeutic Activity

Jianping Lin,[†a] Brenson A. Jassim,[†a] Yunpeng Bai,[†a] Zihan Qu[b], Frederick Nguele Meke,[a] Jiajun Dong,[a] Li Wu,[c] Benjamin Babalola,[b] Jingmei Yu,[a] Haoran Zhang,[a] and Zhong-Yin Zhang[a,b,c,d,*]

[a]Borch Department of Medicinal Chemistry and Molecular Pharmacology, Purdue University, West Lafayette, IN 47907, USA, [b]The James Tarpo Jr. and Margaret Tarpo Department of Chemistry, Purdue University, West Lafayette, IN 47907, USA, [c]Institute for Drug Discovery, Purdue University, West Lafayette, IN 47907, USA, and [d]Institute for Cancer Research, Purdue University, West Lafayette, IN 47907, USA.

[†]Authors contributed equally

Correspondence:

Zhong-Yin Zhang, Email: zhang-zy@purdue.edu; Phone: (765) 494-1403; Fax: (765) 494-1414.

Table of Contents

Figure S1. Detergent assay of compound L-32……………………………………………………S3

Figure S2. Selected kinase and protein activity of L-32……………………………………...S4

Figure S3. Pharmacokinetic profile and metabolic stability of L-32…………………………S5

Figure S4. Cytotoxicity assay of compound L-1 and L-32……………………………….…....S6

Figure S5. *In vivo* antitumor efficacy study of L-1 and L-32……………………………….....S7

Figure S6. IHC staining for T cell infiltration into MC38 tumors…………………………….S8

Scheme S1. Synthesis scheme of compound L-34……………..……………………..……..…....S9

Scheme S2. Synthesis scheme of compound L-35…………………….………………..……..…....S9

^{1}H, ^{13}C NMR Spectra for the final compounds……………..…………..…..………........................S10

LC/MS Traces and HRMS data for Compounds L-26, L-29, and L-32 …………….........................S43

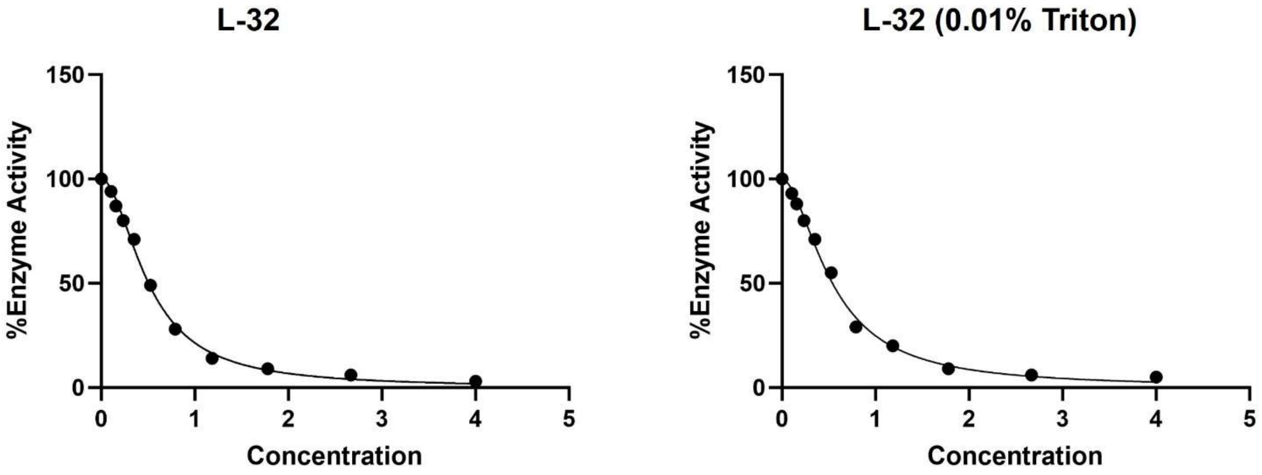

Figure S1: The potency of compound **L-32** is not affected by the presence of detergent. The IC_{50} value remained consistent at 0.53 ± 0.03 μM in the presence of the detergent TritonTM X-100, indicating that compound **L-32** does not inhibit PTPN22 activity through nonspecific aggregation.

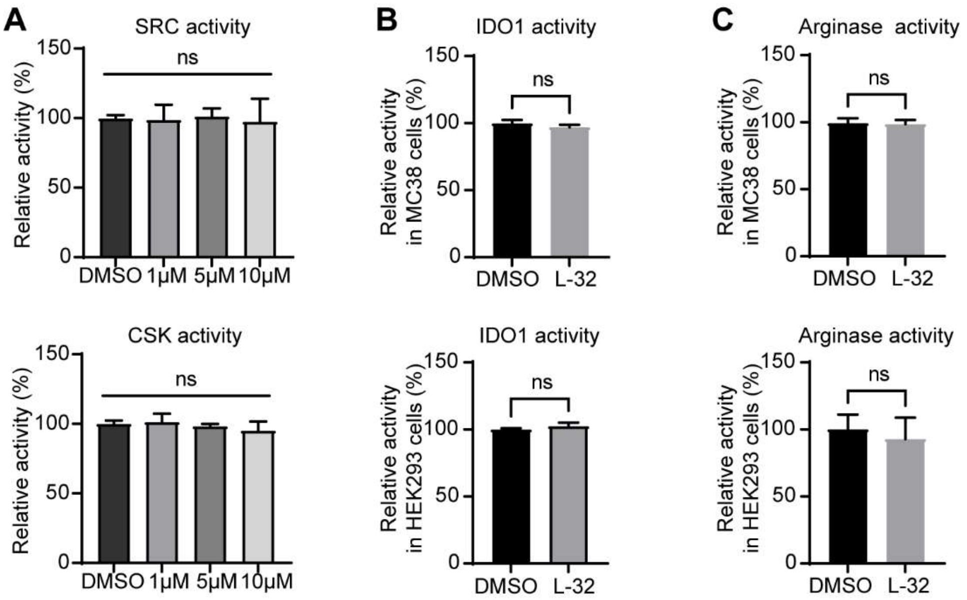

Figure S2: (**A**) The relative SRC kinase (top) and CSK kinase (bottom) activity was evaluated using recombinant SRC and CSK enzymes following treatment of **L-32** at 1, 5, and 10 μM in the presence of 10 μM ATP and the specific protein substrates: recombinant SHP2 (5 μM) for SRC and recombinant SRC (5 μM) for CSK. After 30-minute incubation at 30°C, the reaction was stopped and the release of inorganic phosphate was quantified using BIOMOL Green reagent. (**B-C**) The relative enzymatic activity of IDO1 (**B**) and Arginase (**C**) was evaluated in MC38 murine colon carcinoma (top) and HEK293 (bottom) cell lysates following treatment with either DMSO or 5 μM of **L-32**. IDO1 activity was quantified using the Abcam Indoleamine 2,3-Dioxygenase 1 Activity Assay Kit, measuring the fluorometric conversion of tryptophan to N-formylkynurenine (NFK). Arginase activity was determined using the Sigma-Aldrich Arginase Activity Assay Kit. Results are expressed as relative activity (%) normalized by DMSO control and represent the mean ± SD of 3 independent replicates.

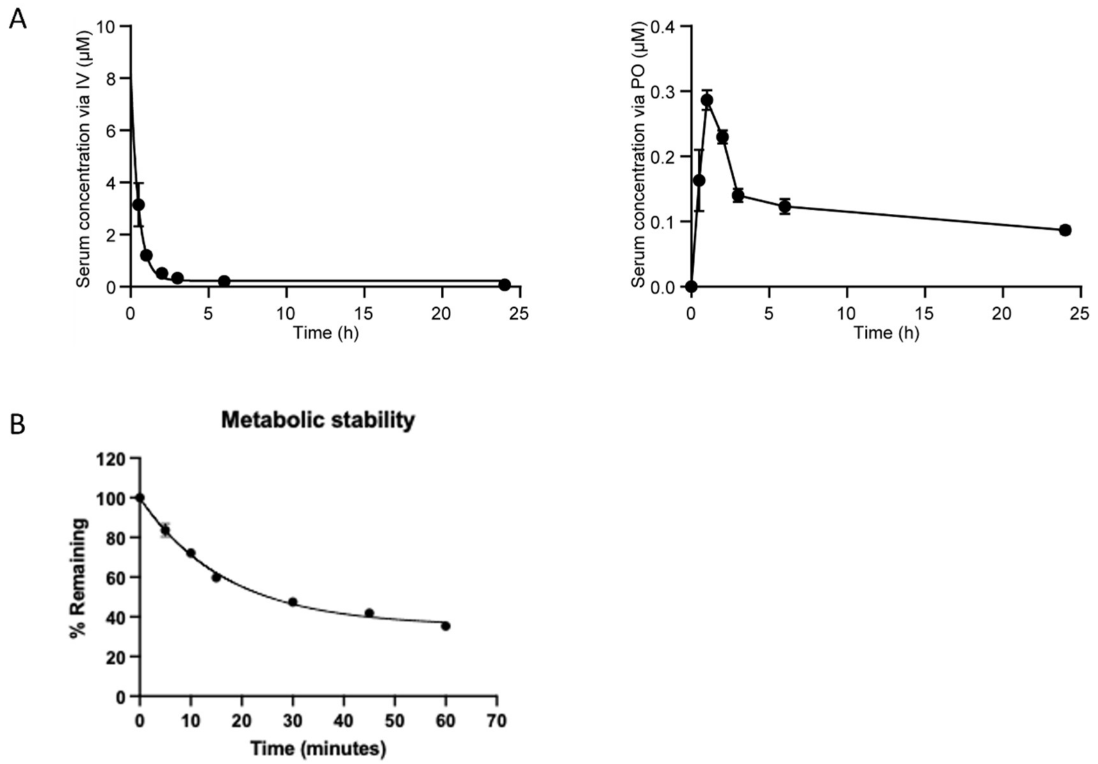

Figure S3. (A) Pharmacokinetic profile of **L-32** via IV (left) and PO (right). *In vivo* pharmacokinetic data based on mass spectrometric quantification at 0.5, 1, 2, 4, 6, and 24-hour time points after a single 10 mg/kg IV (left) dose or PO (right) for three mice. (B) The **L-32** metabolic stability curve in mouse liver microsomes.

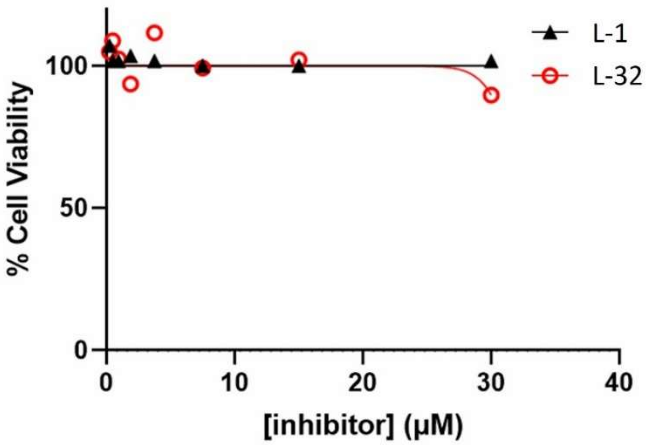

Figure S4. % cell viability of HEK293 cells upon 24h treatment of up to 30μM of **L-1** and **L-32**.

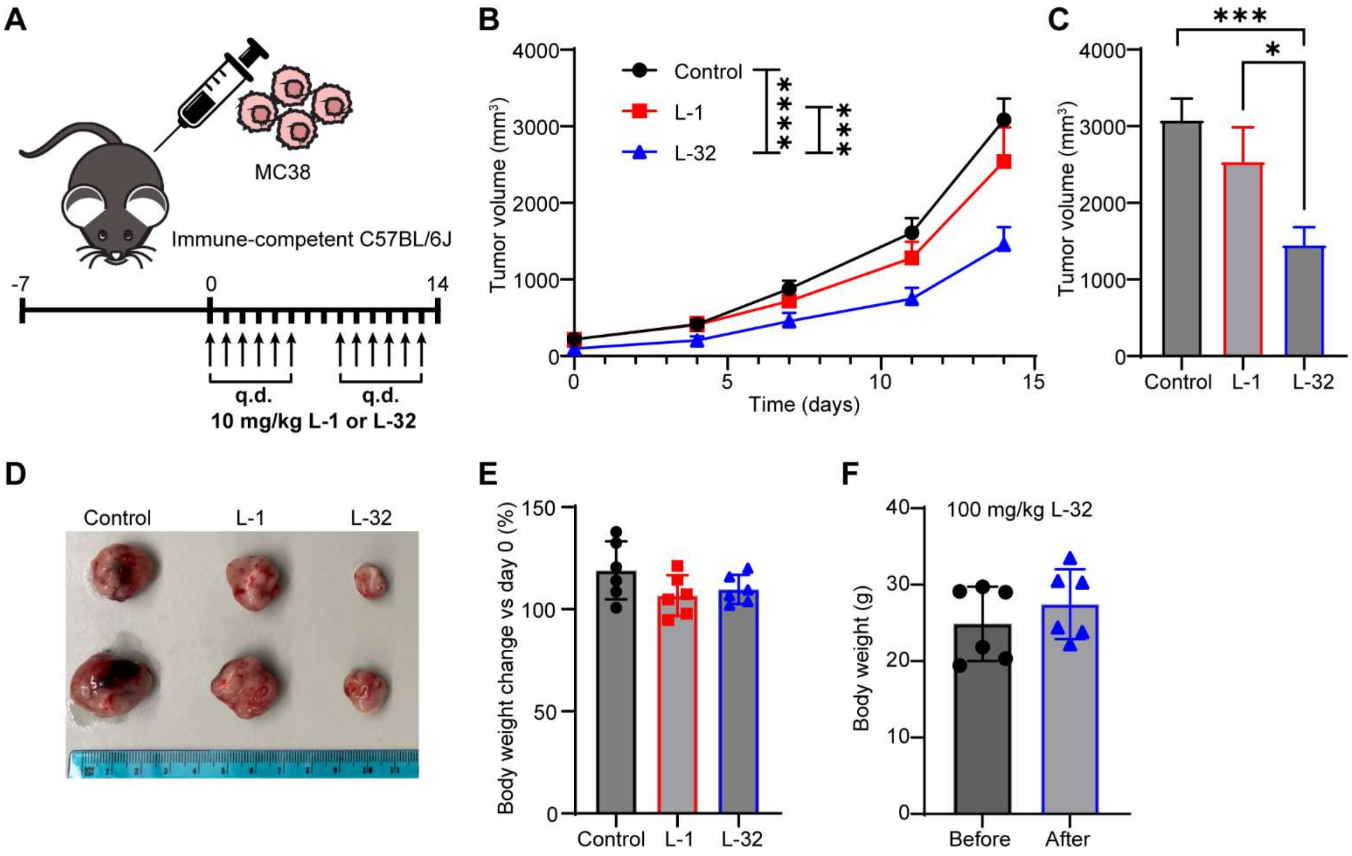

Figure S5. (**A**) Schematic of the **L-1**/**L-32** treatment of the MC38 tumor model. Mice were injected with 10^6 MC38 cells at both sides to induce tumor growth, and divided into three groups: Control group (n=6), **L-1** treatment group (n=6) and **L-32** treatment group (n=6). 7 days after injection, **L-32** is administered at 10 mg/kg for two cycles of daily intraperitoneal injections for six consecutive days, separated by a two-day treatment-free interval. (**B**)&(**C**) Changes in tumor volume and final tumor volume for control, **L-1** and **L-32** treatment groups. *p<0.05, ***p<0.001 and ****p<0.0001. (**D**) Images of representative dissected tumors. (**E**) Body weight change compared to day 0 upon treatment of control, **L-1** and **L-32** at 10 mg/kg. (**F**) Body weight before or after treatment of **L-32** at 100 mg/kg for 5 days.

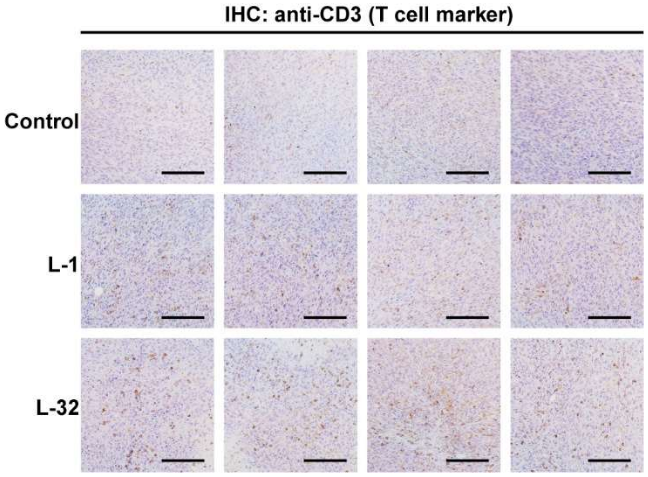

Figure S6. IHC staining for T cell infiltration into MC38 tumors. Scale bar = 100 μm.

Scheme S1. Synthesis of Compound **L-34**[a]

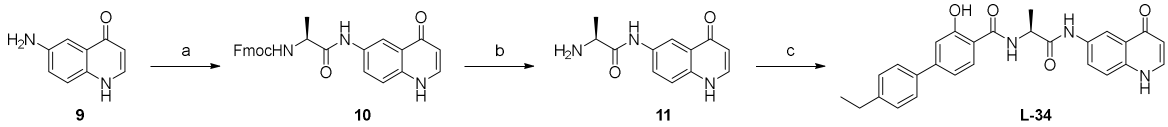

[a]Reagents and conditions: (a) Fmoc-L-Ala-OH, HOBt, HBTU, DIPEA, DMF, r.t., overnight, 87%; (b) Piperidine, DMF, r.t., 1 h, 76%; (c) 4'-ethyl-3-hydroxy-[1,1'-biphenyl]-4-carboxylic acid, HOBt, HBTU, DIPEA, DMF, r.t., overnight, 71%.

Scheme S2. Synthesis of **L-35**[a]

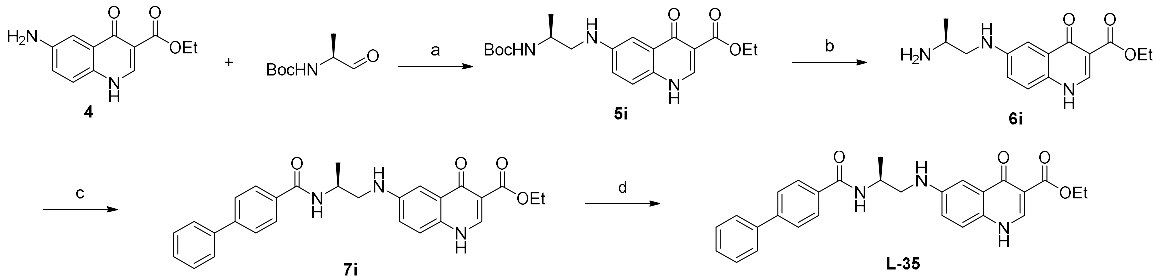

[a]Reagents and conditions: (a) $NaBH_3CN$, DMF, r.t., overnight, 81%; (b) CF_3COOH, DMF, r.t., 6 h, 86%; (c) Biphenyl-4-carboxylic acid, HOBt, HBTU, DIPEA, DMF, r.t., overnight, 78%; (d) KOH, $MeOH/H_2O$, 60 °C, 16 h, 75%.

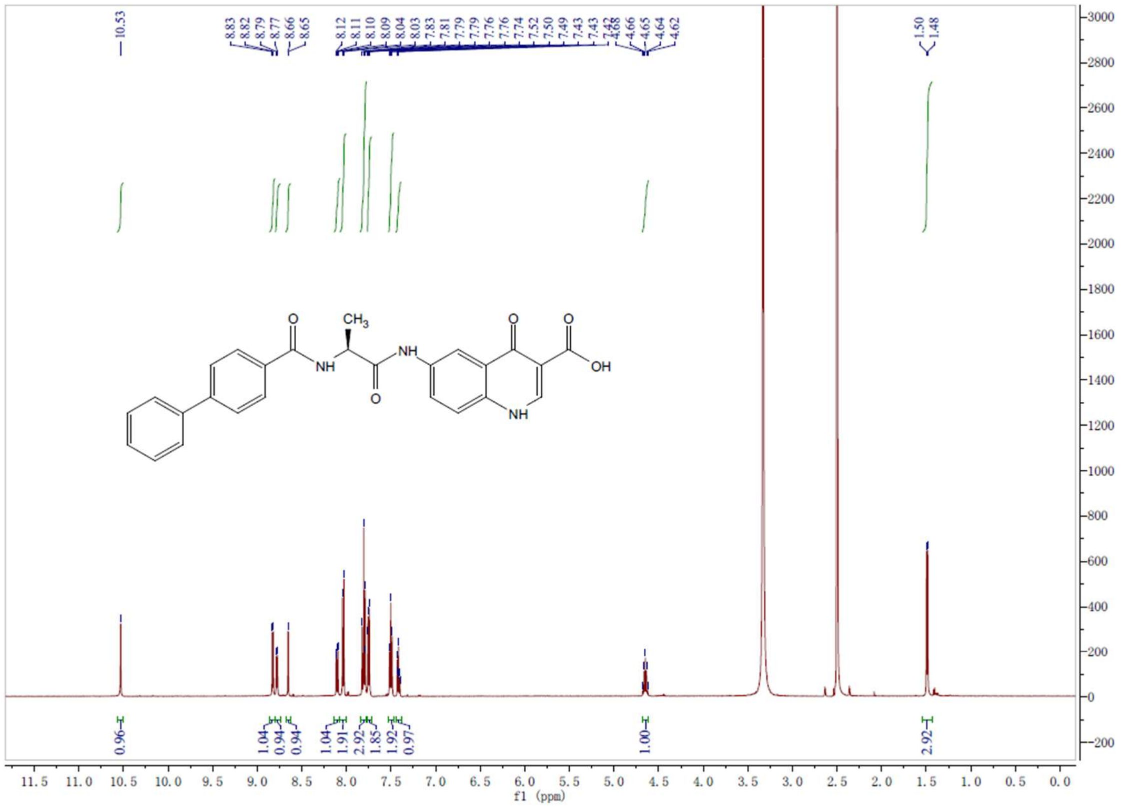

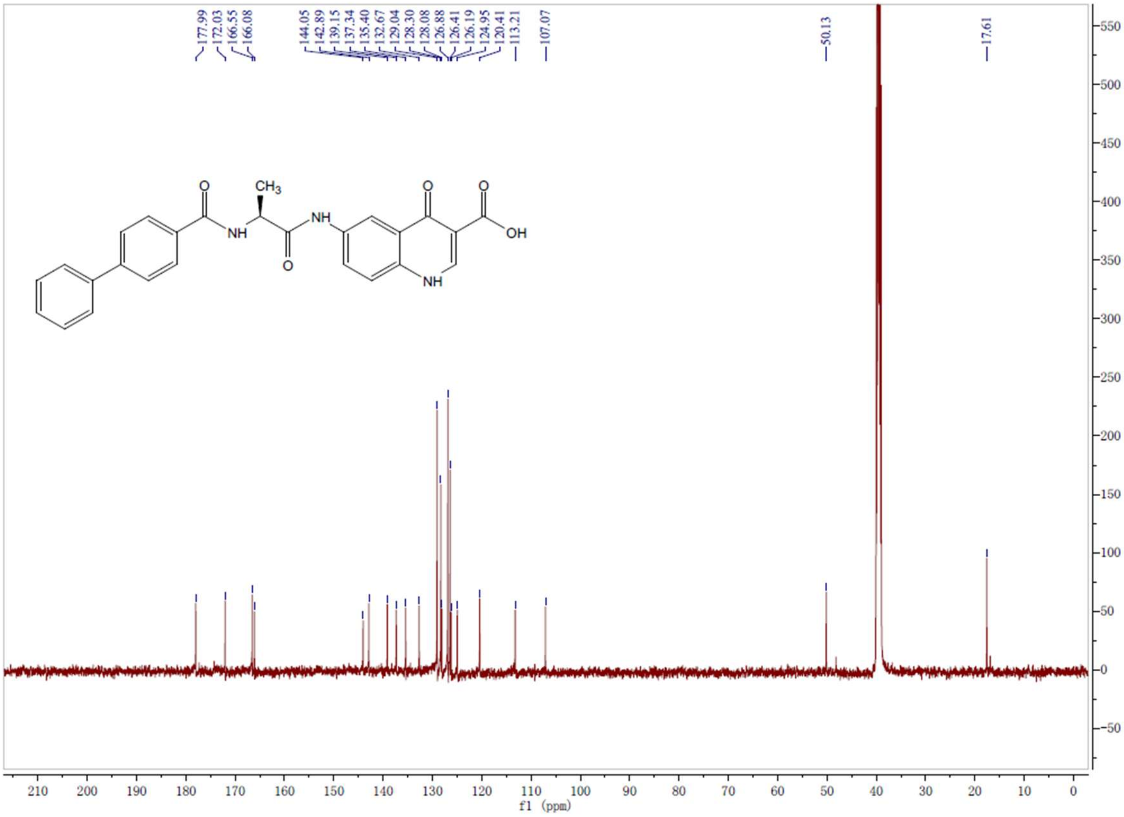

^{1}H NMR and ^{13}C NMR Spectra for L-1

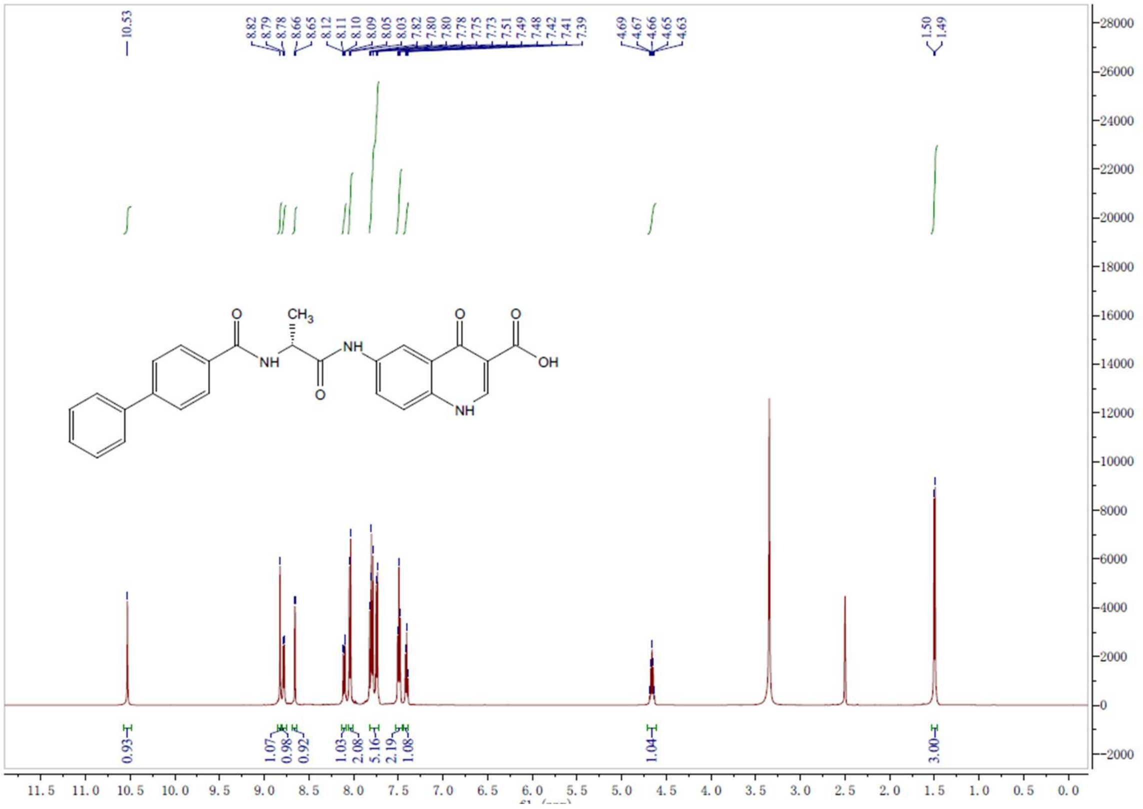

10.53
8.82 8.79 8.78 8.66 8.65 8.12 8.11 8.10 8.09 8.05 8.03 7.82 7.80 7.78 7.75 7.73 7.51 7.49 7.48 7.42 7.41 7.39
4.69 4.67 4.66 4.65 4.63
1.50 1.49
CH3
O
NH
OH
0.93 1.07 0.98 0.92 1.03 2.08 5.16 2.19 1.08 1.04 3.00
f1 (ppm)

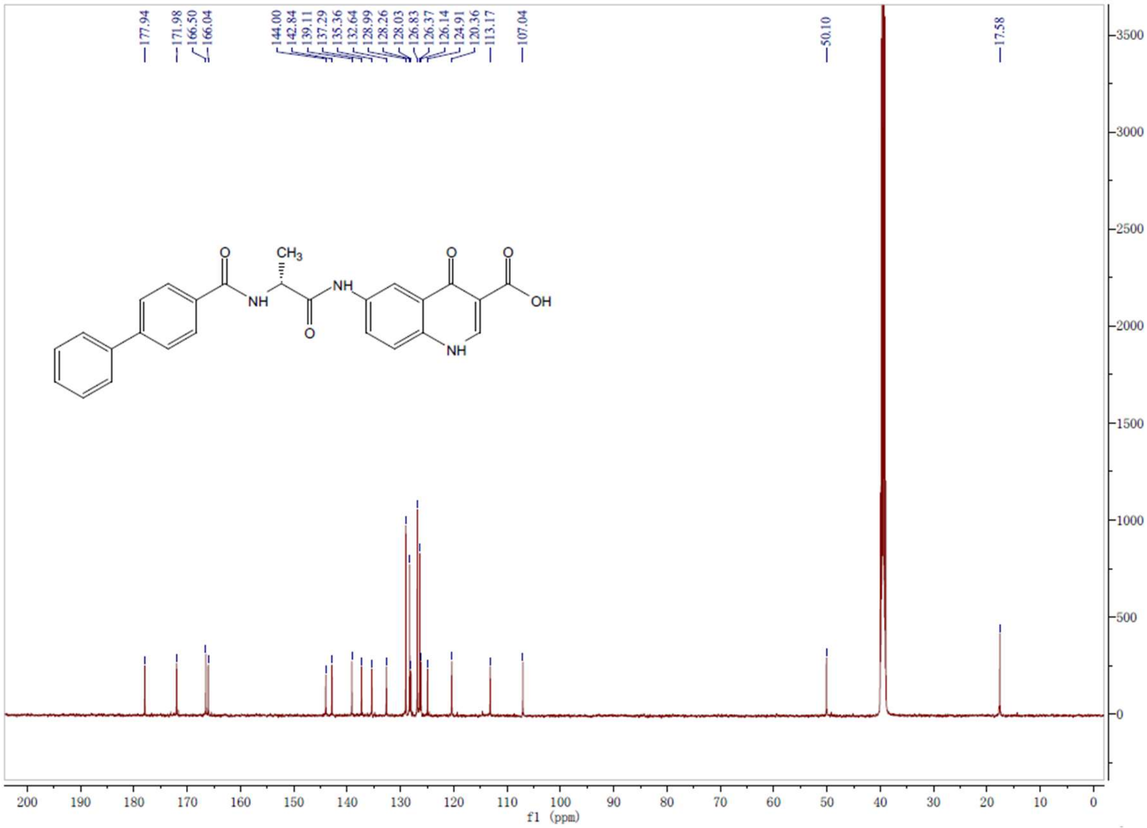

177.94 171.98 166.50 166.04
144.00 142.84 139.11 137.29 135.36 132.64 128.99 128.26 128.03 126.83 126.37 126.14 124.91 120.36 113.17 107.04
50.10
17.58
CH3
O
NH
OH
f1 (ppm)

^{1}H NMR and ^{13}C NMR Spectra for L-2

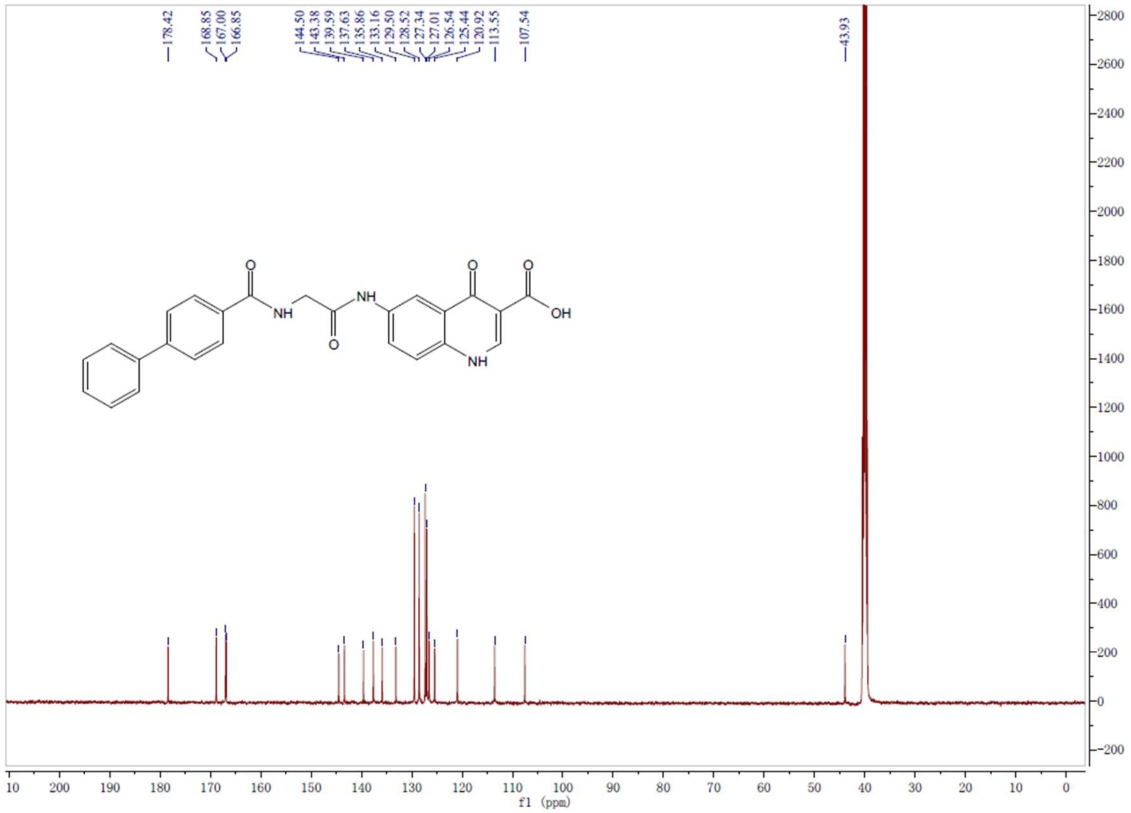

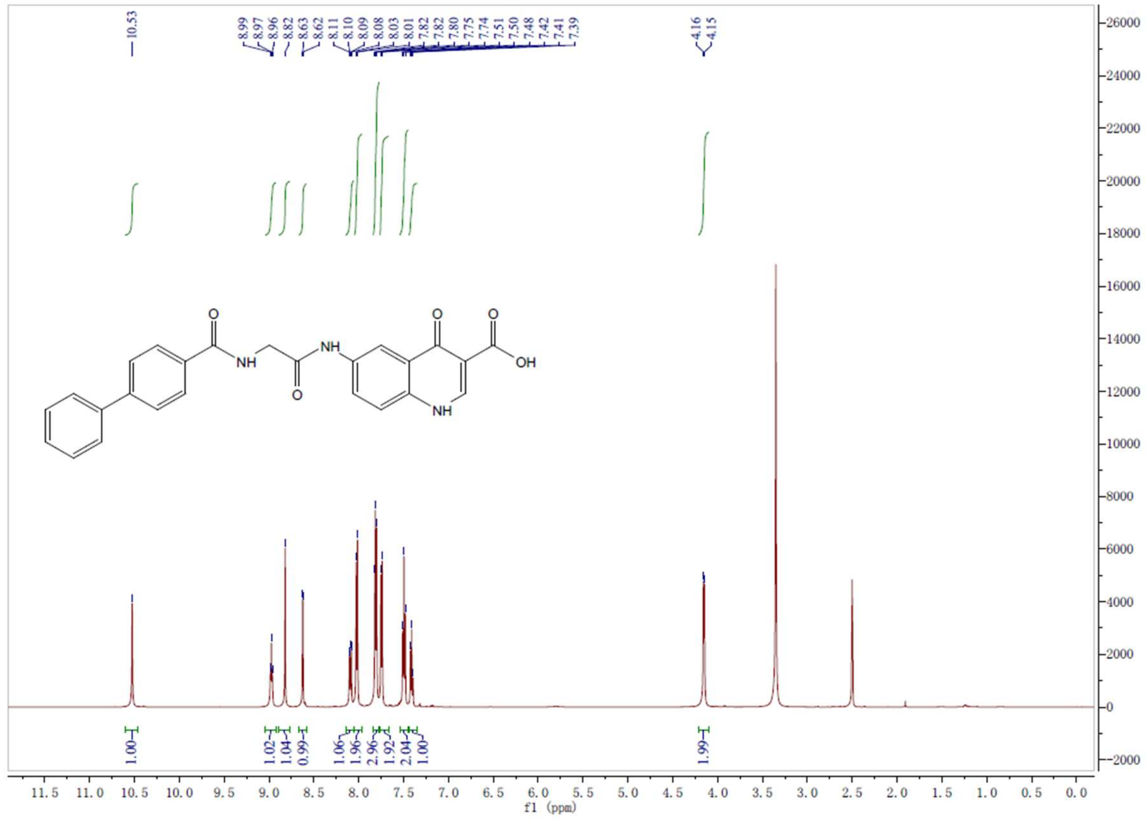

^{1}H NMR and ^{13}C NMR Spectra for L-3

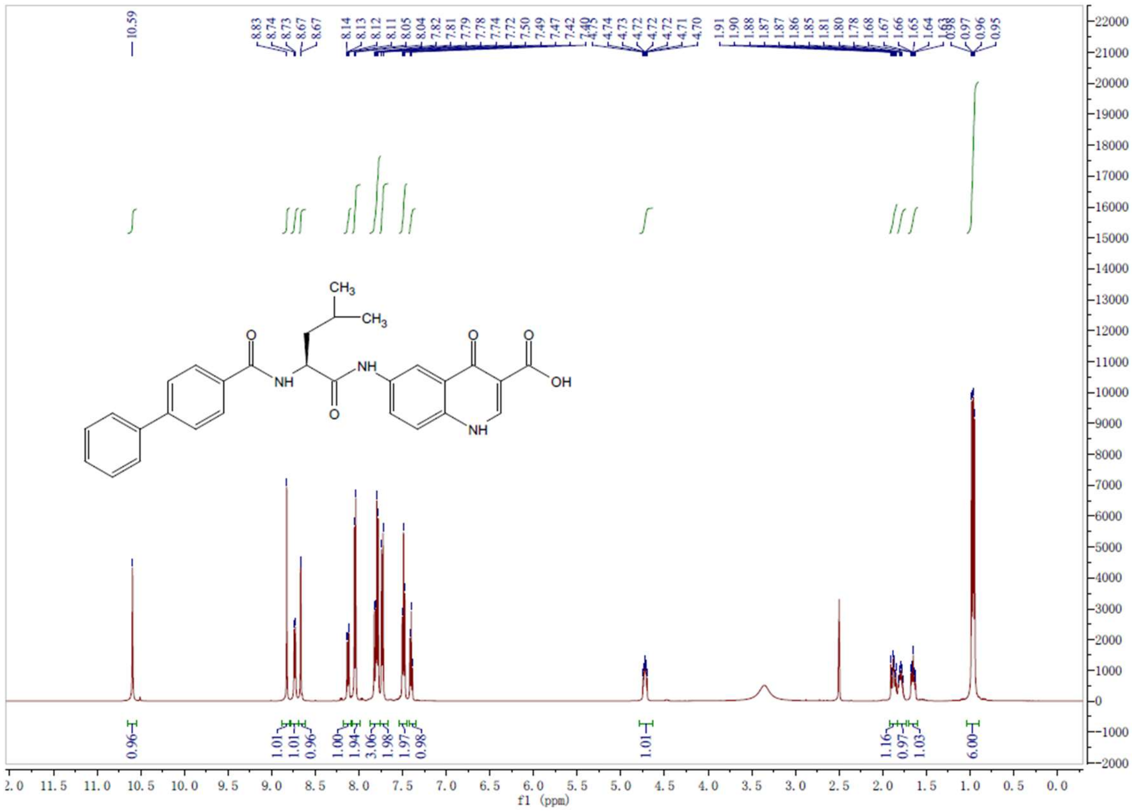

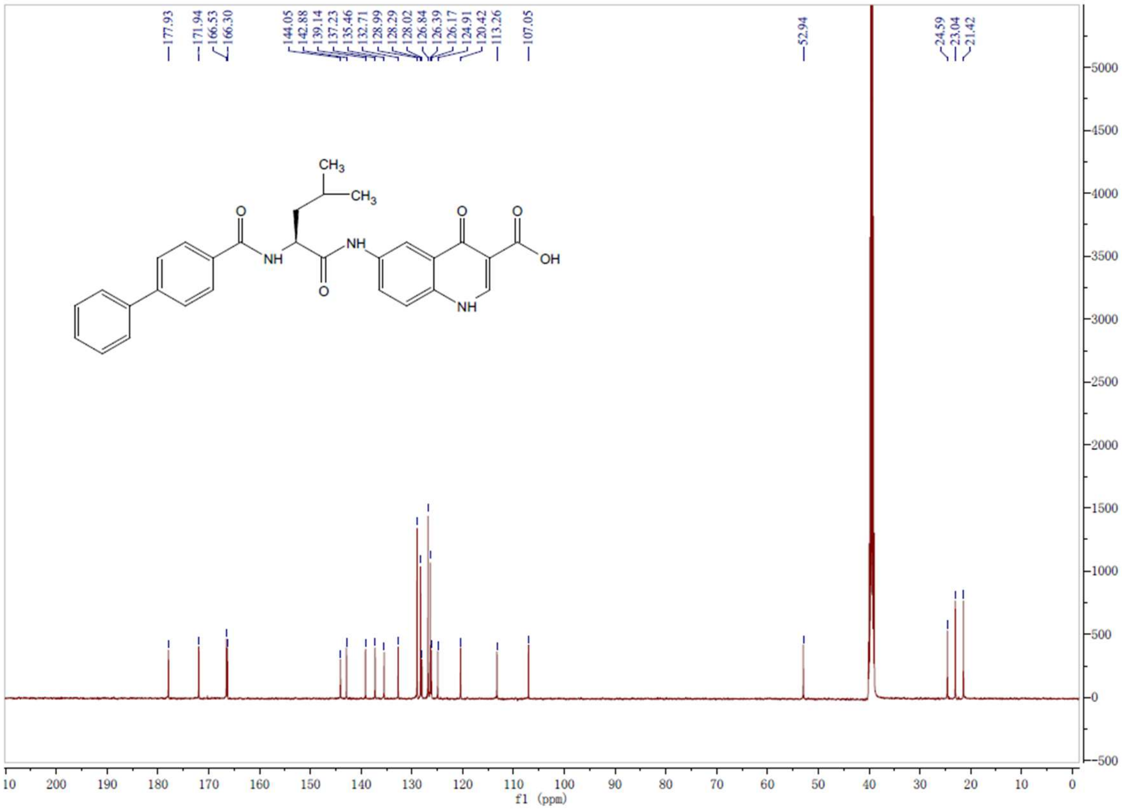

^{1}H NMR and ^{13}C NMR Spectra for L-4

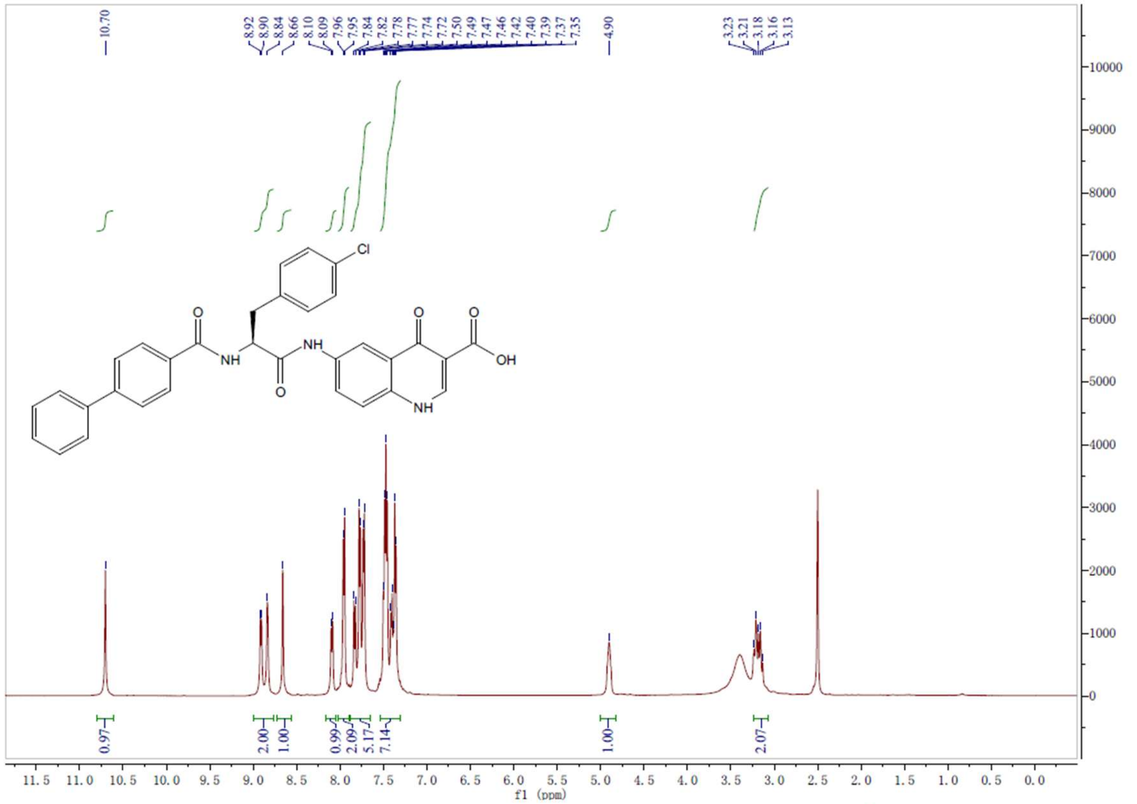

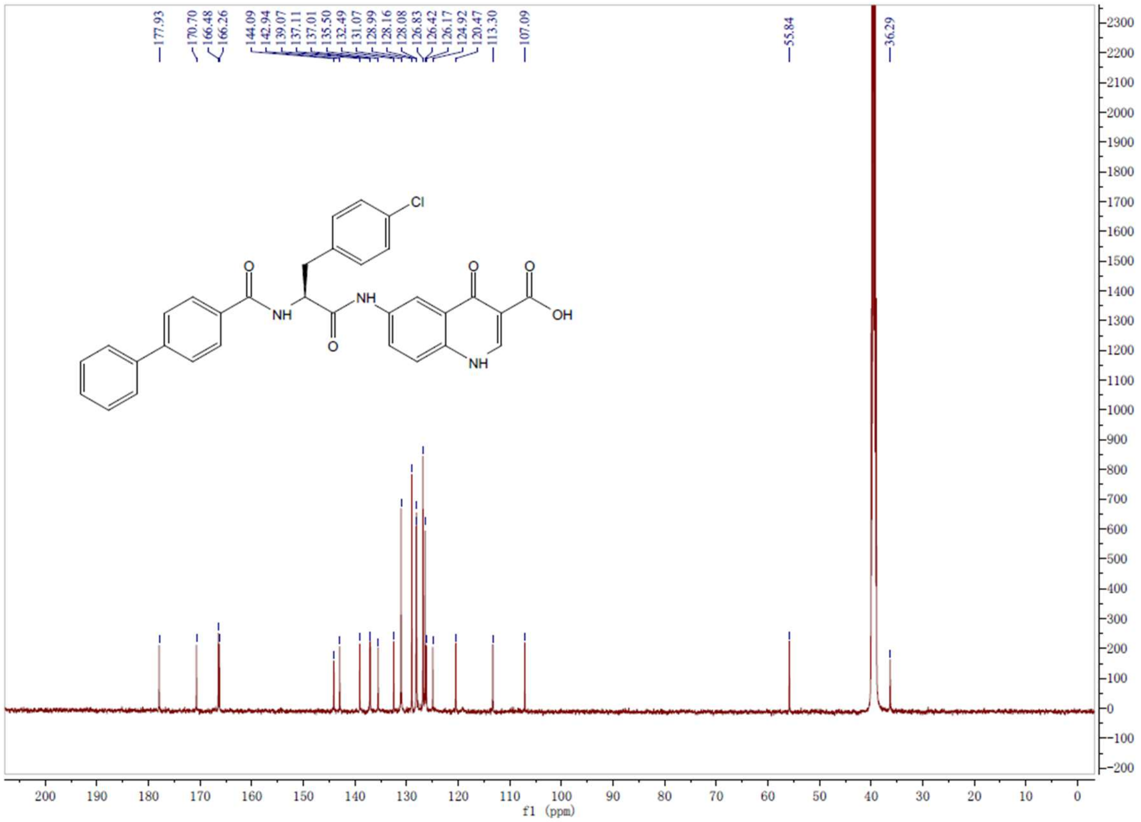

^1H NMR and ^{13}C NMR Spectra for L-5

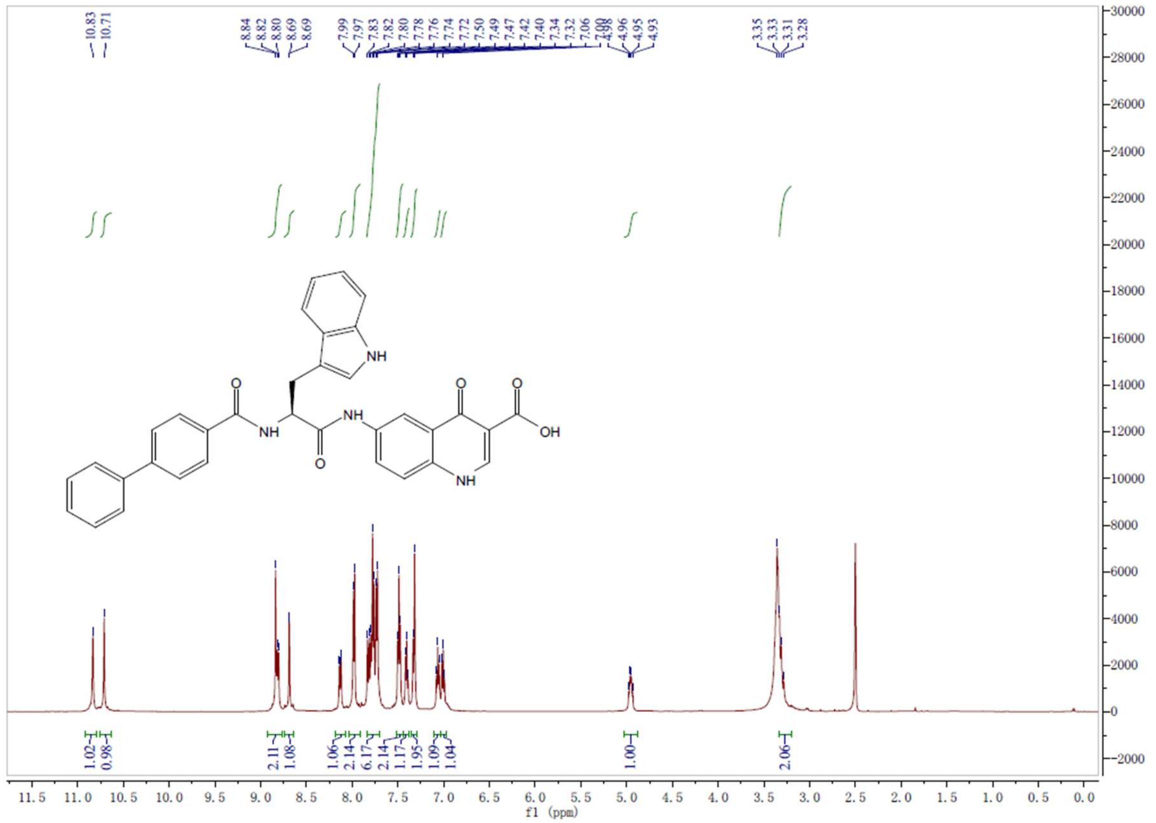

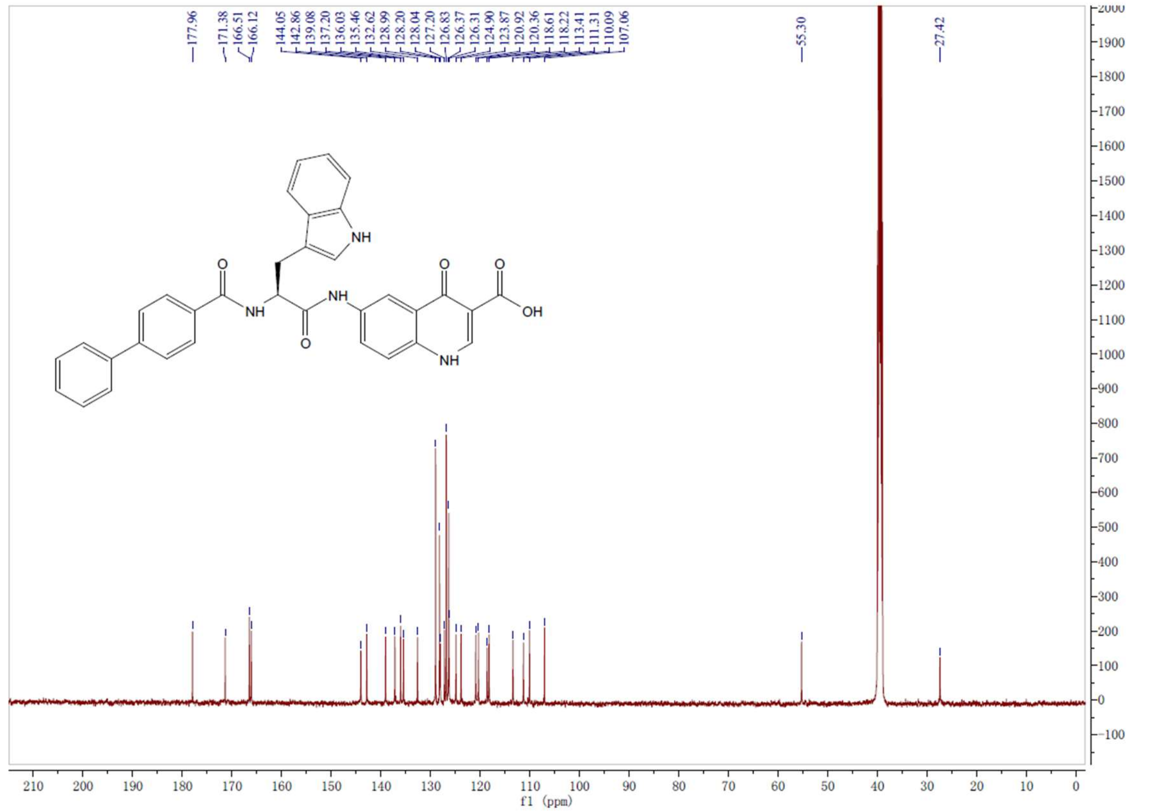

^{1}H NMR and ^{13}C NMR Spectra for L-6

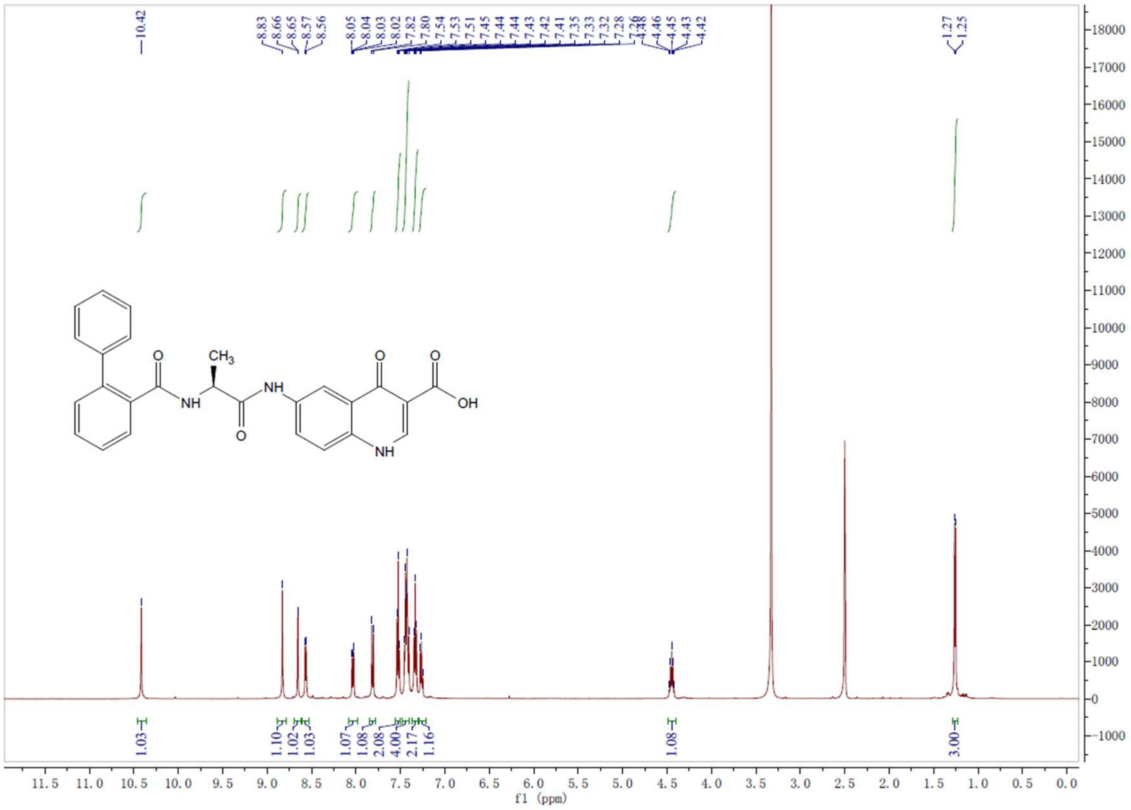

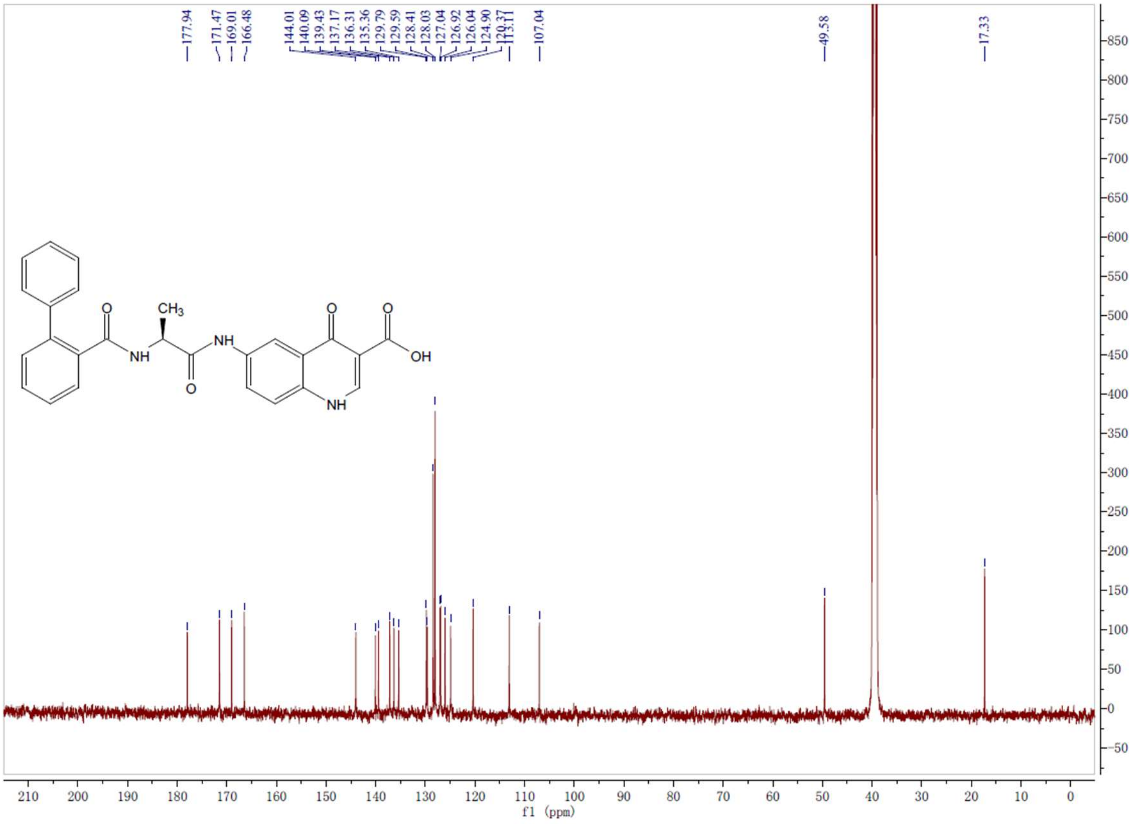

^{1}H NMR and ^{13}C NMR Spectra for L-9

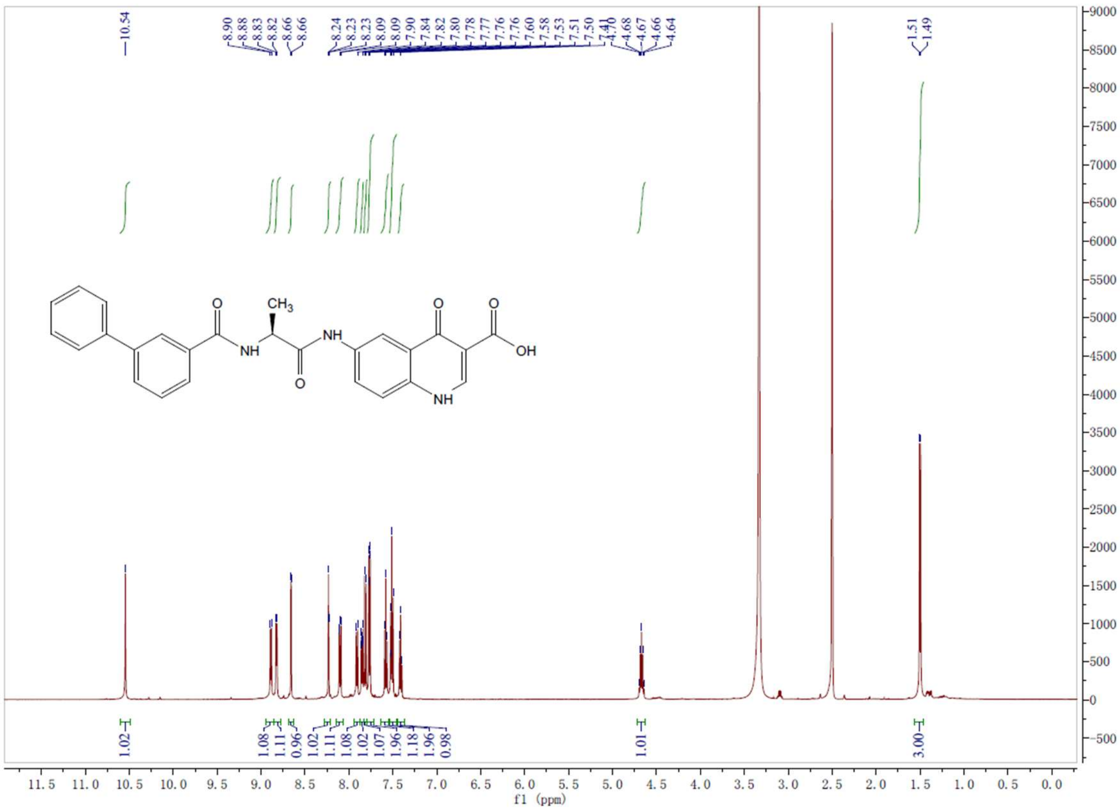

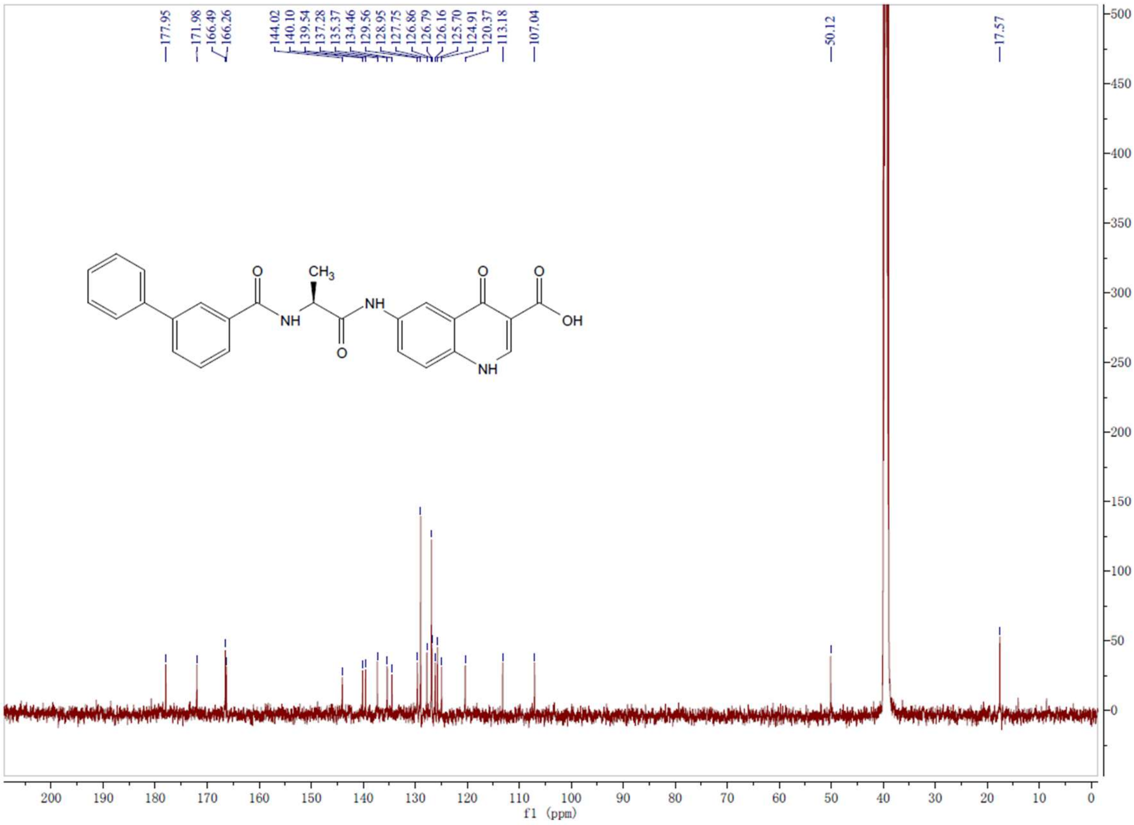

^{1}H NMR and ^{13}C NMR Spectra for L-10

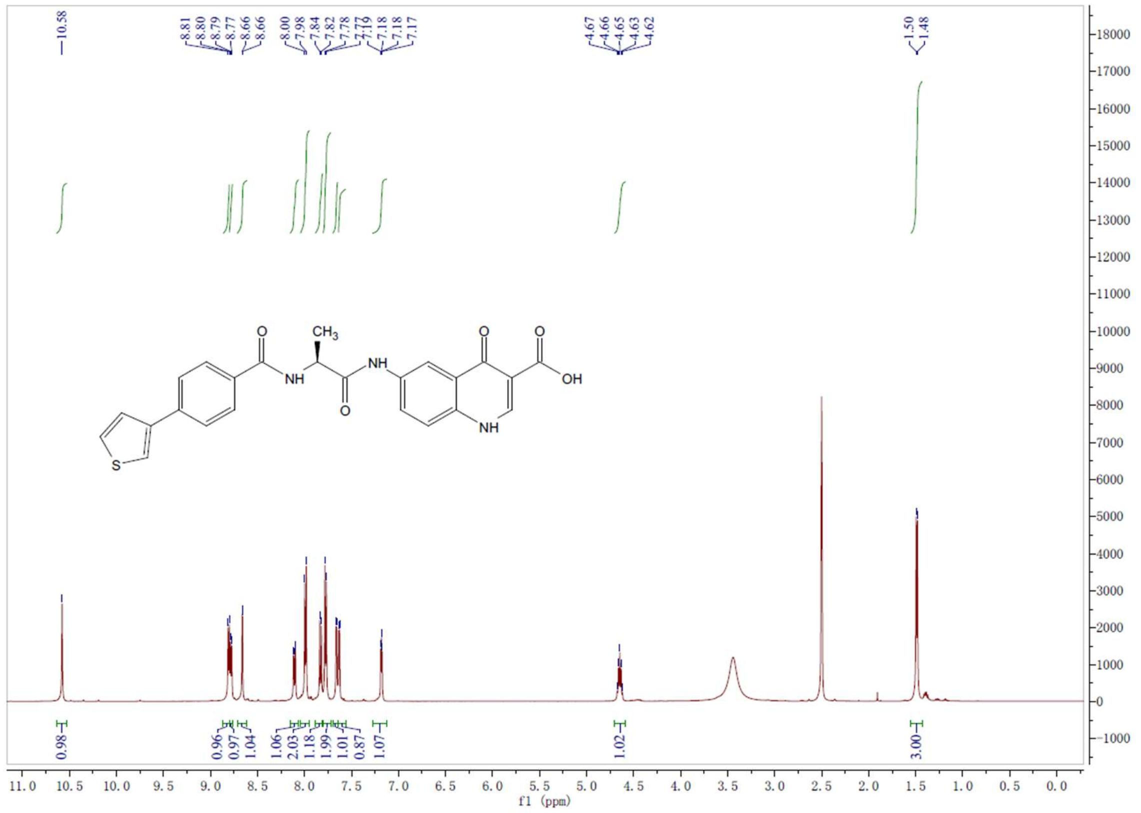

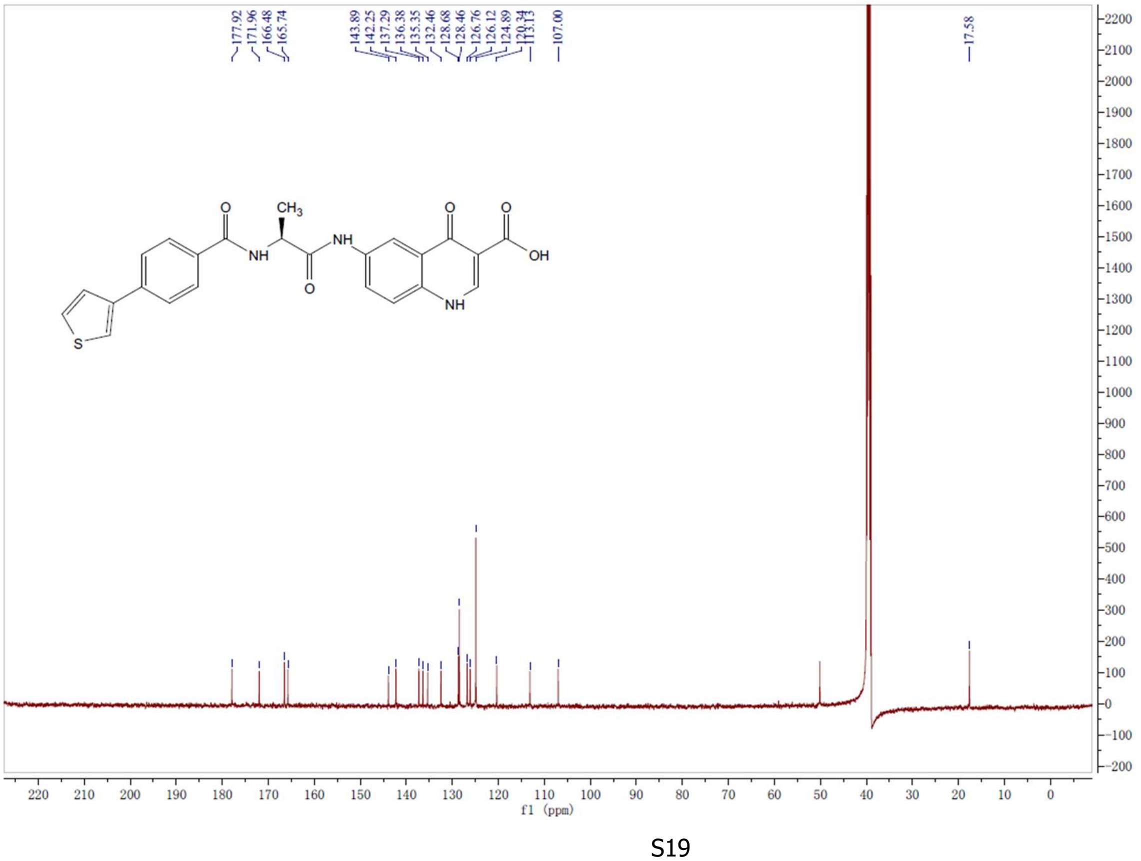

^{1}H NMR and ^{13}C NMR Spectra for L-11

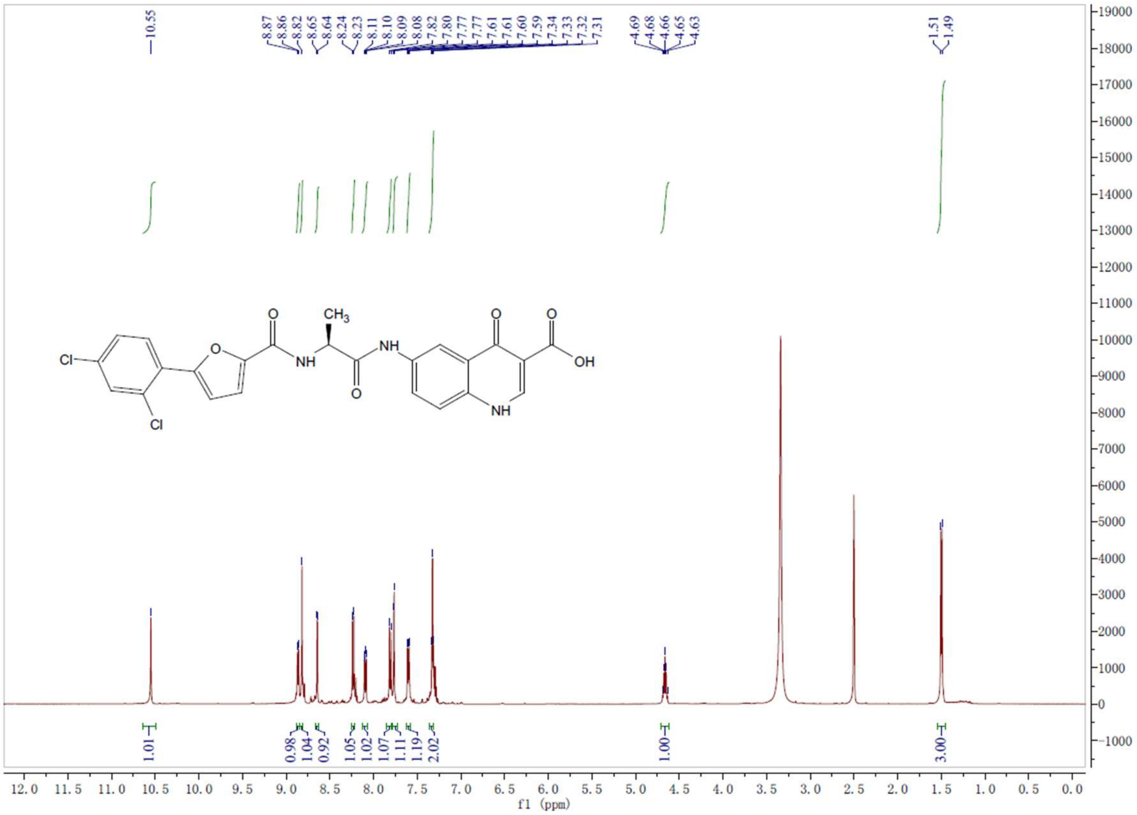

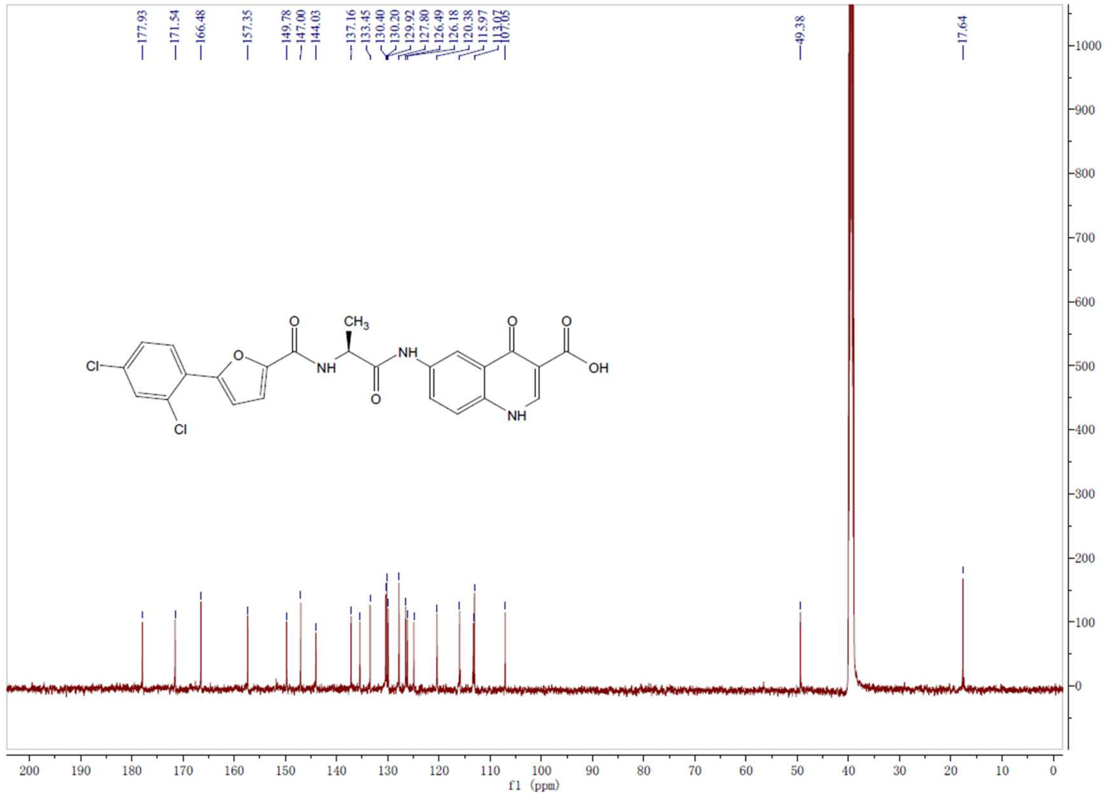

^{1}H NMR and ^{13}C NMR Spectra for L-12

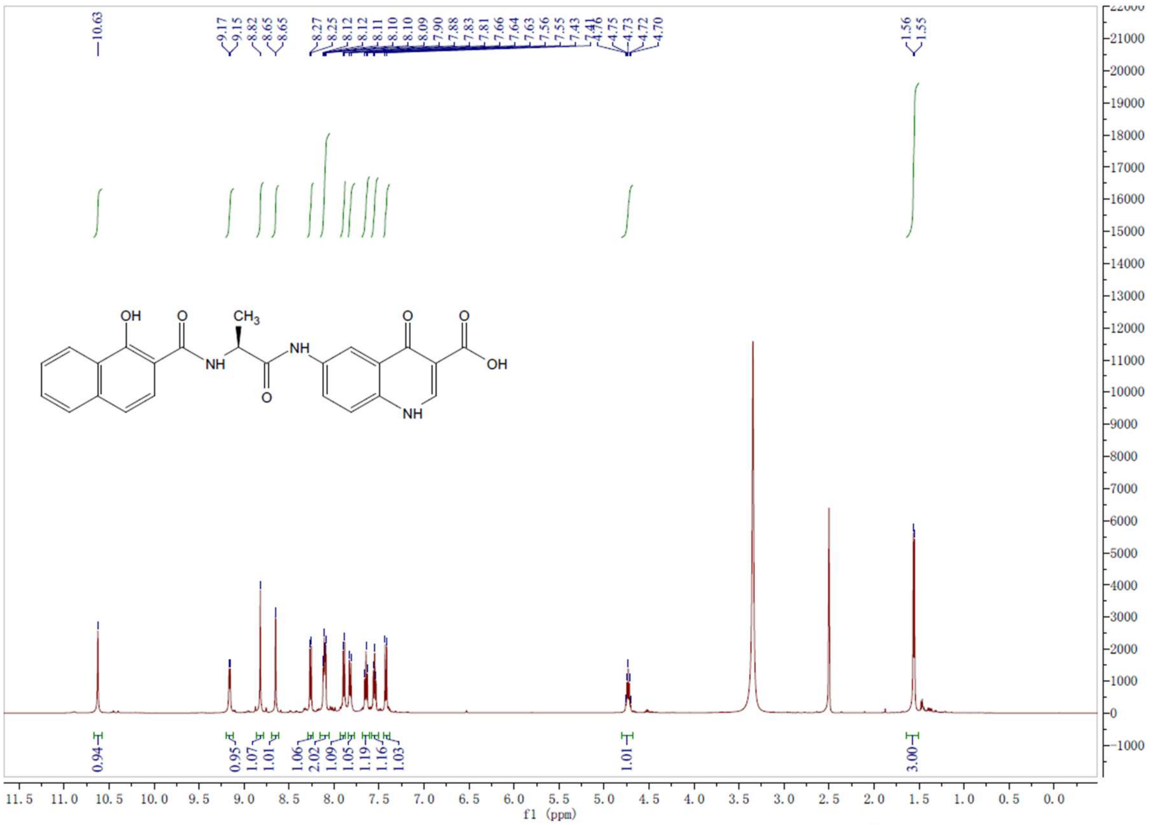

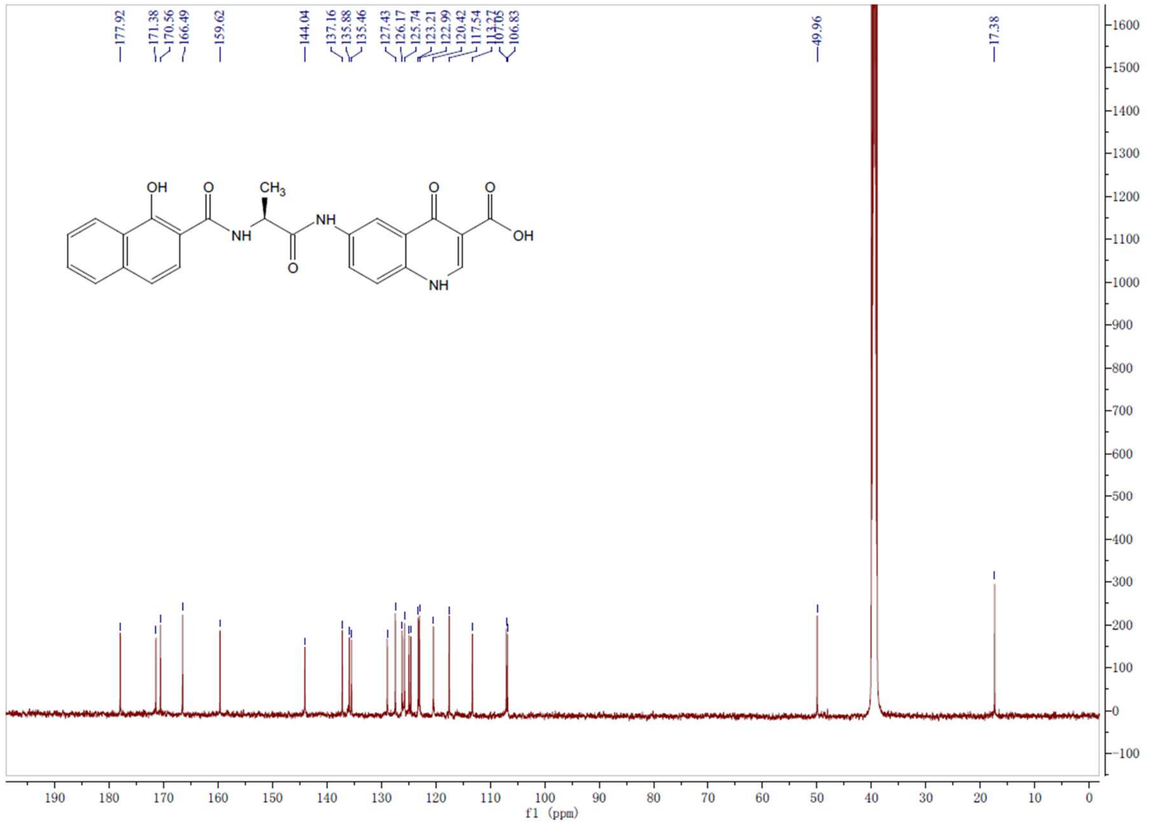

^{1}H NMR and ^{13}C NMR Spectra for L-13

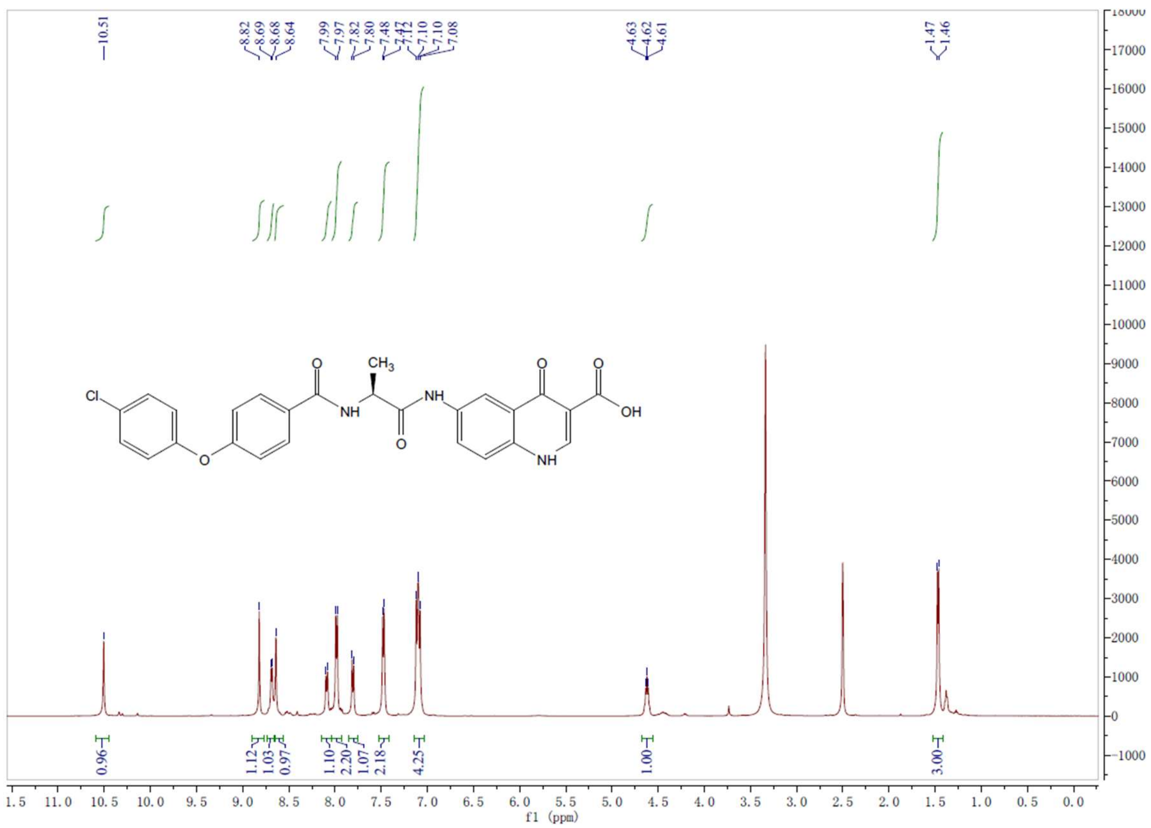

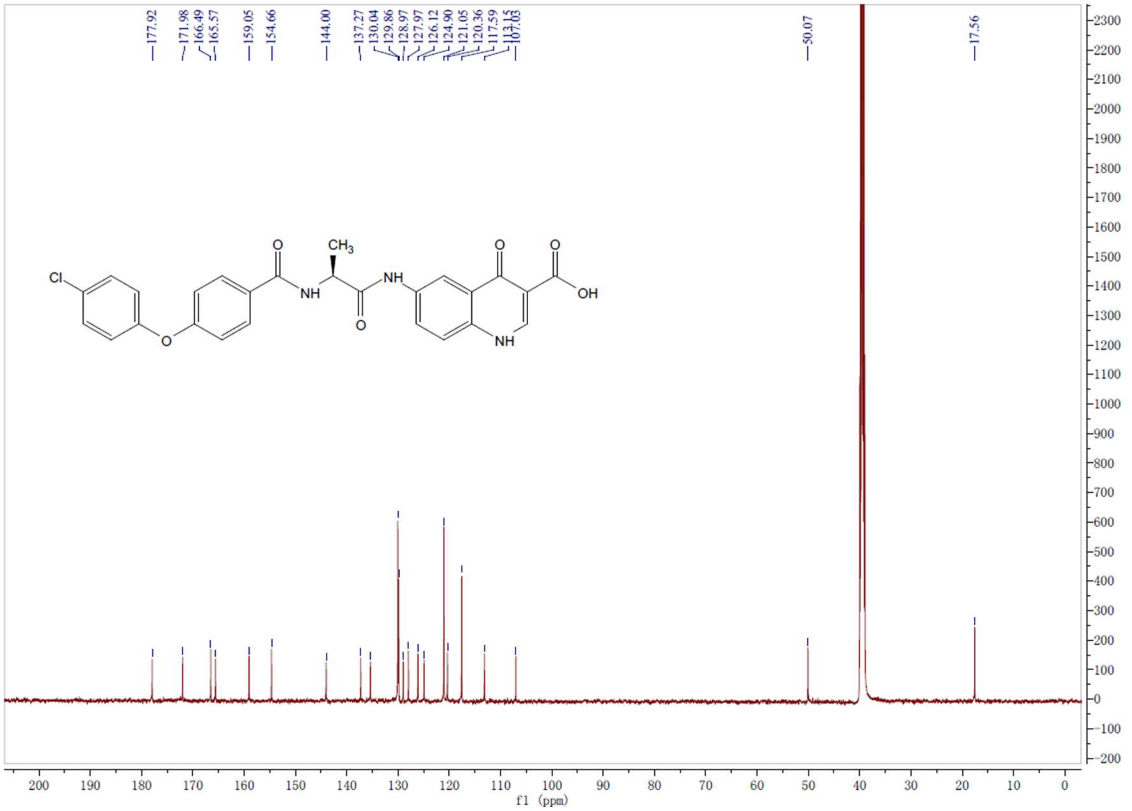

^{1}H NMR and ^{13}C NMR Spectra for L-14

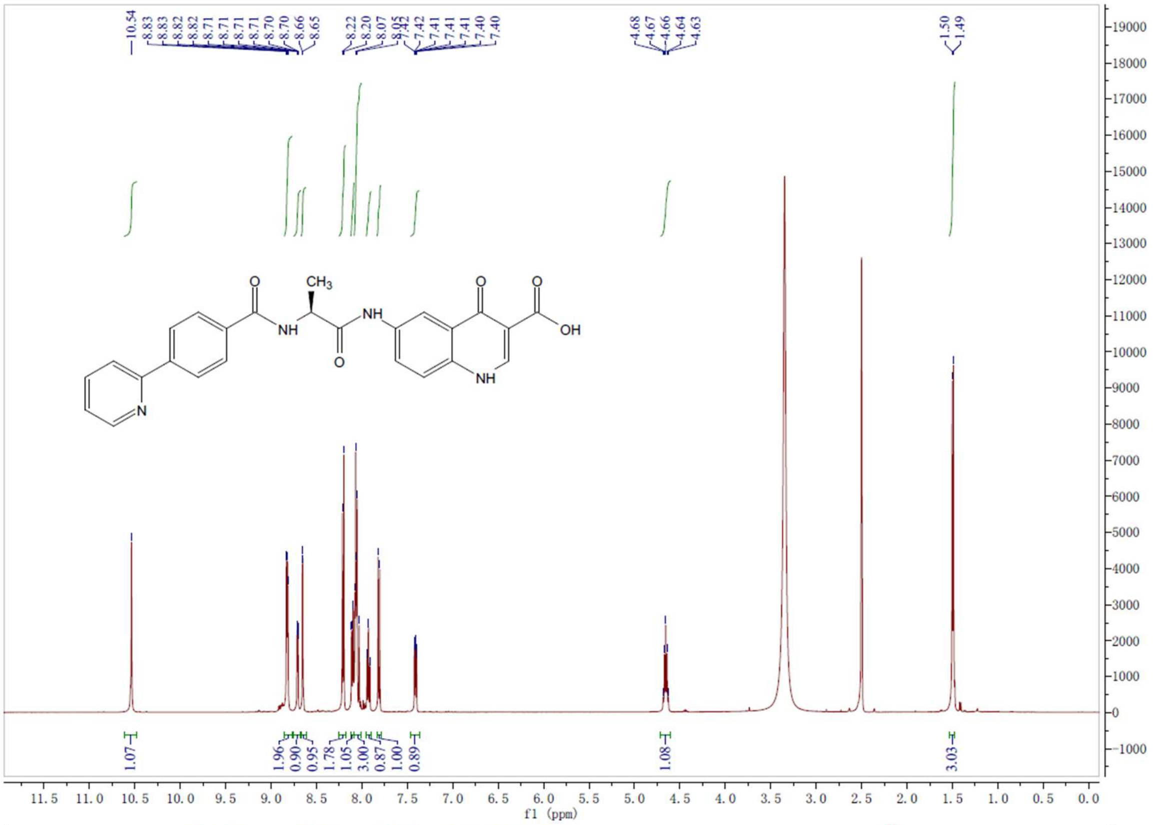

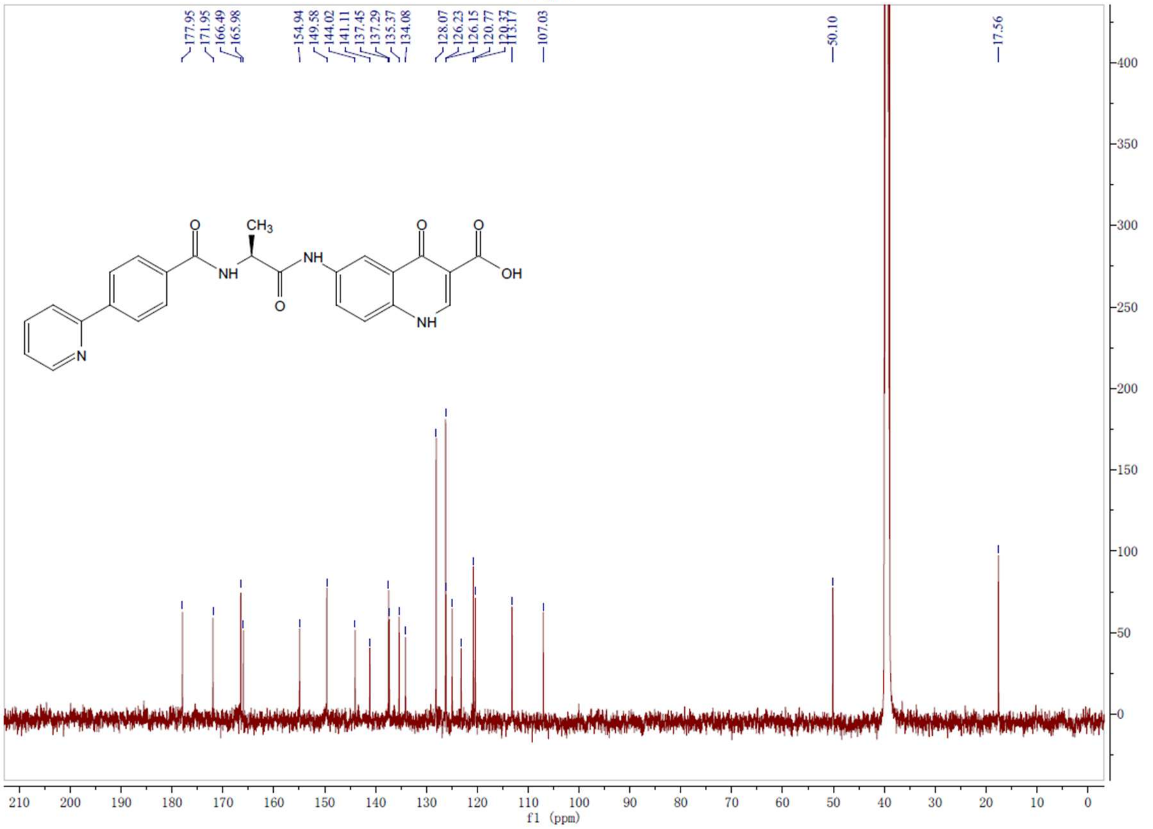

^{1}H NMR and ^{13}C NMR Spectra for L-15

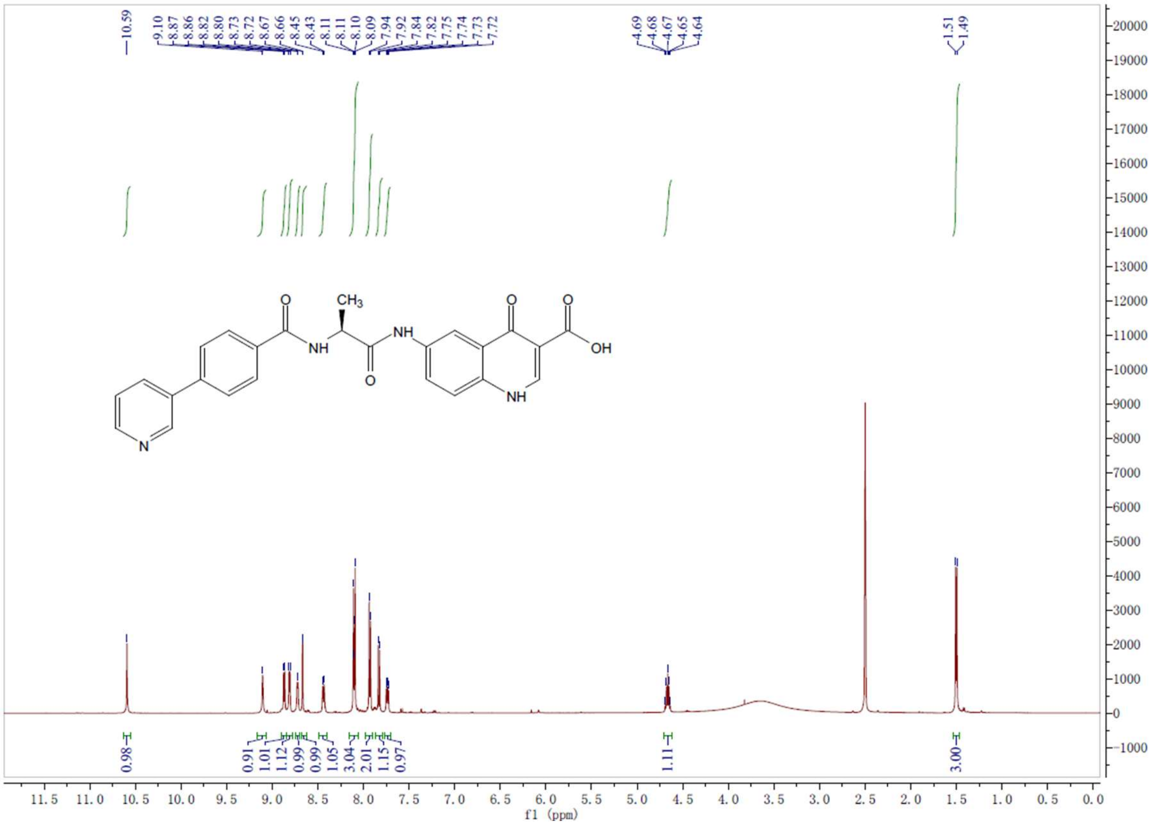

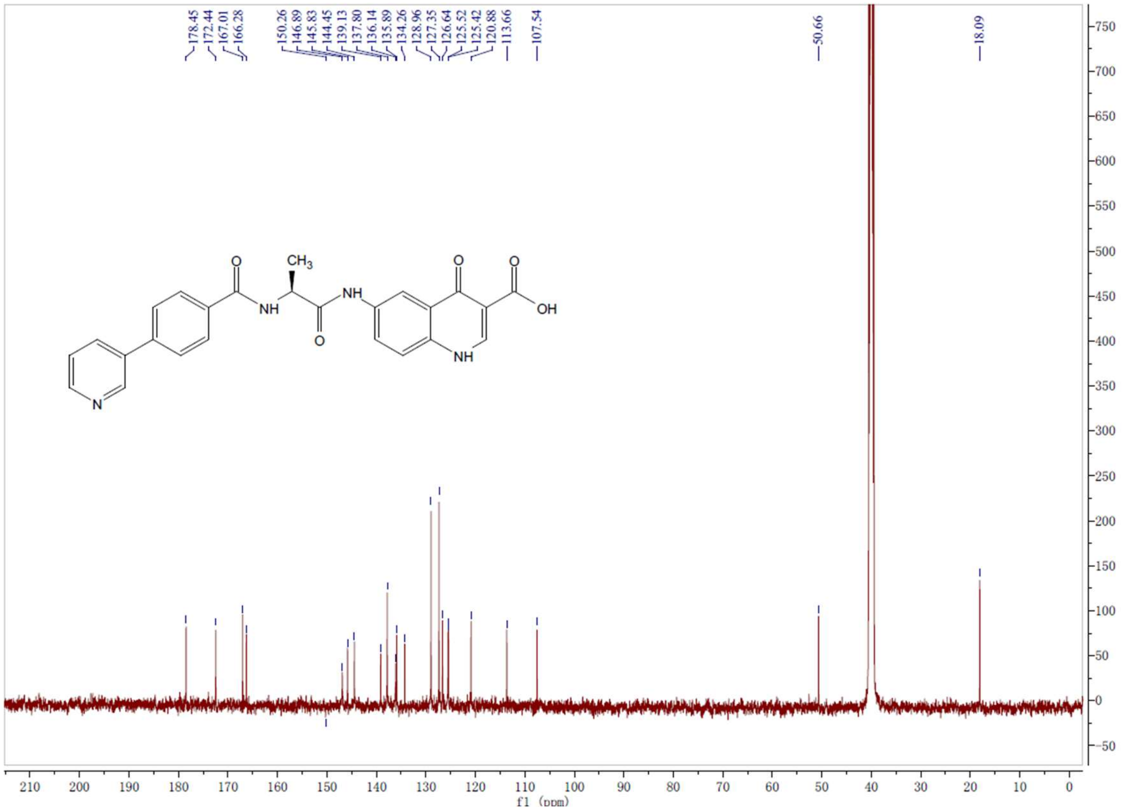

^{1}H NMR and ^{13}C NMR Spectra for L-16

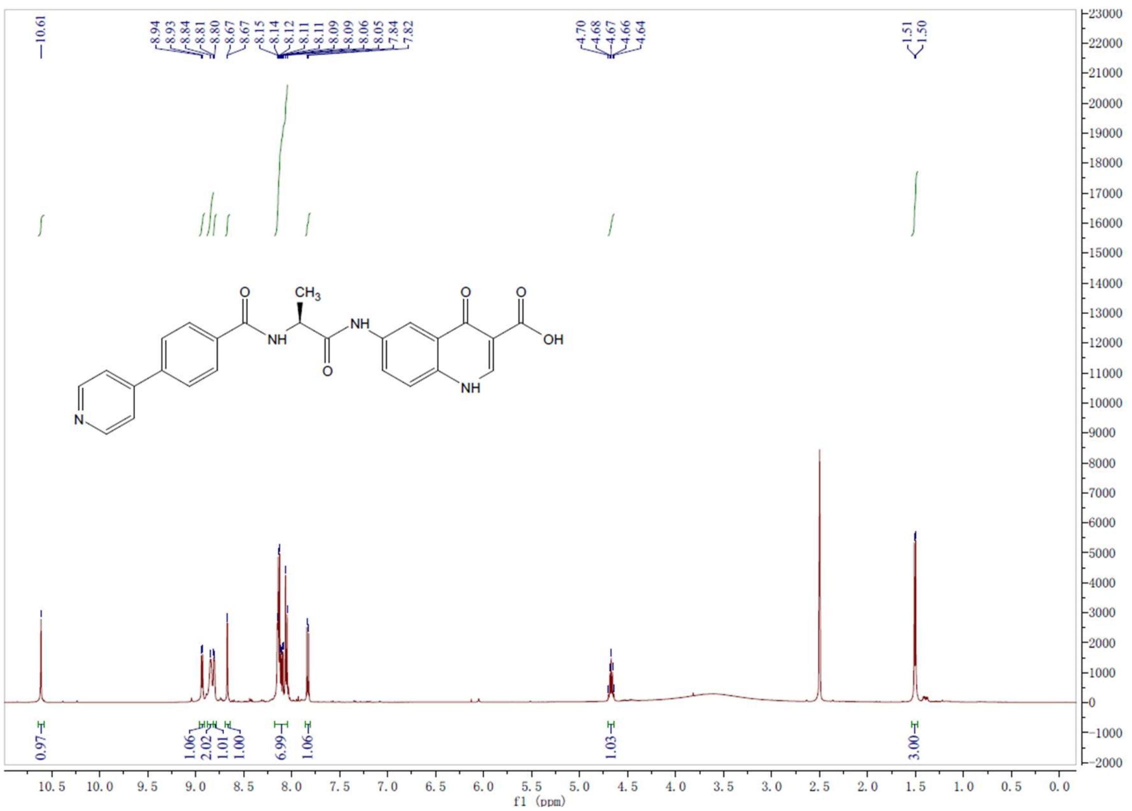

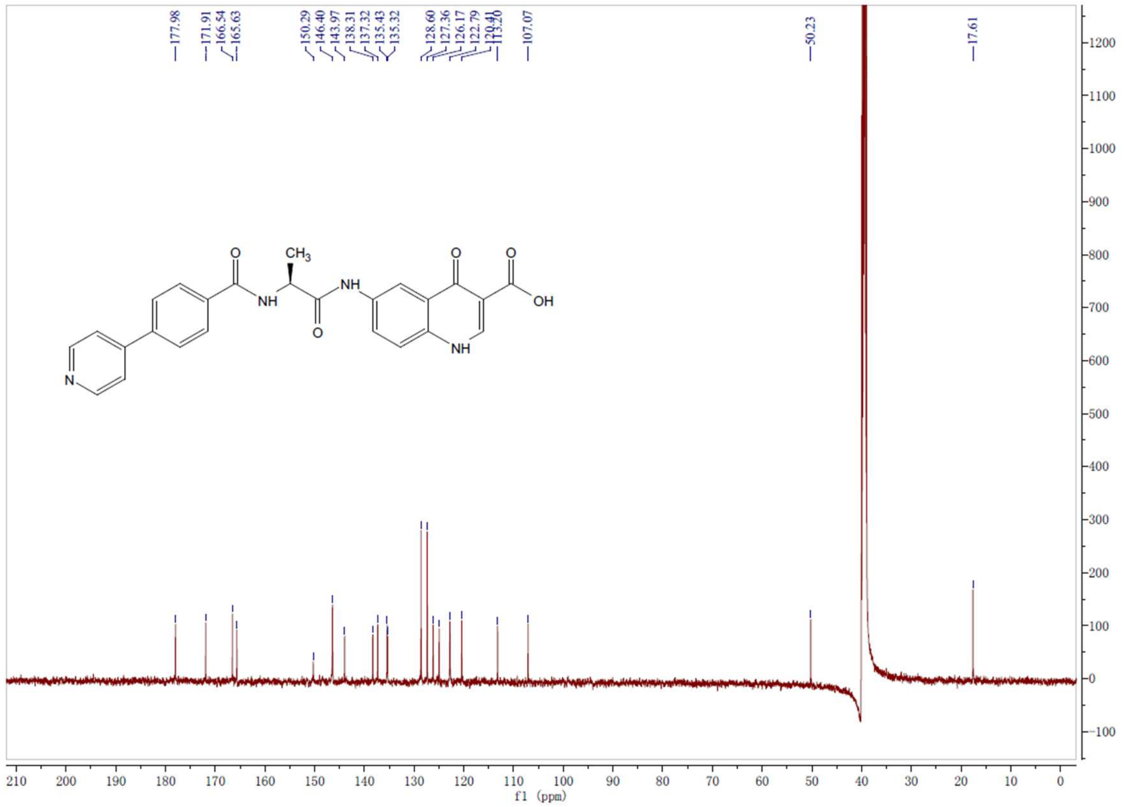

^{1}H NMR and ^{13}C NMR Spectra for L-17

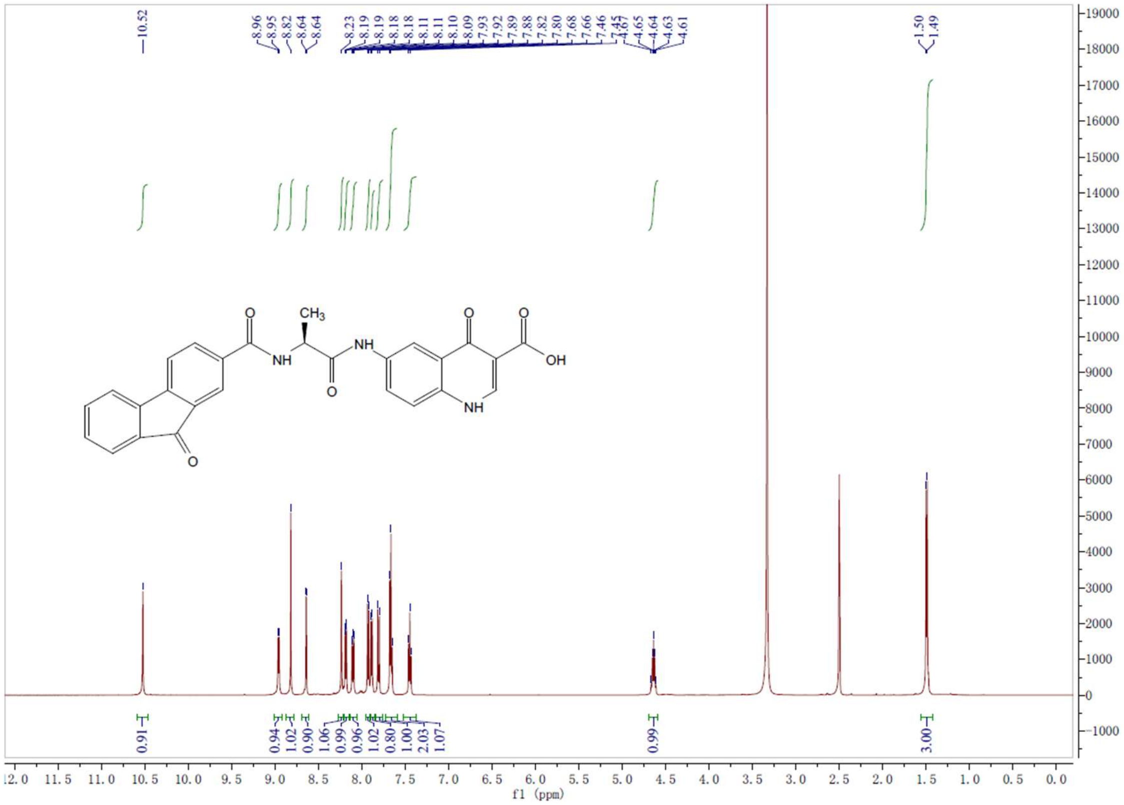

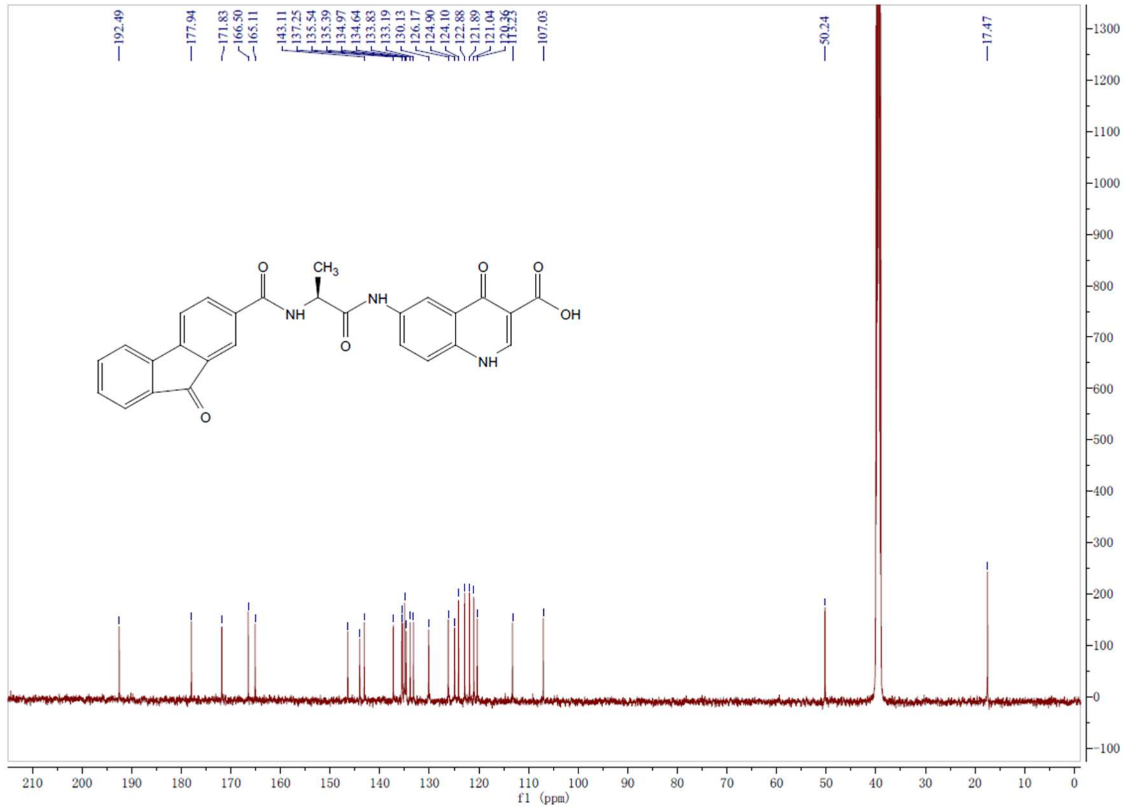

^{1}H NMR and ^{13}C NMR Spectra for L-18

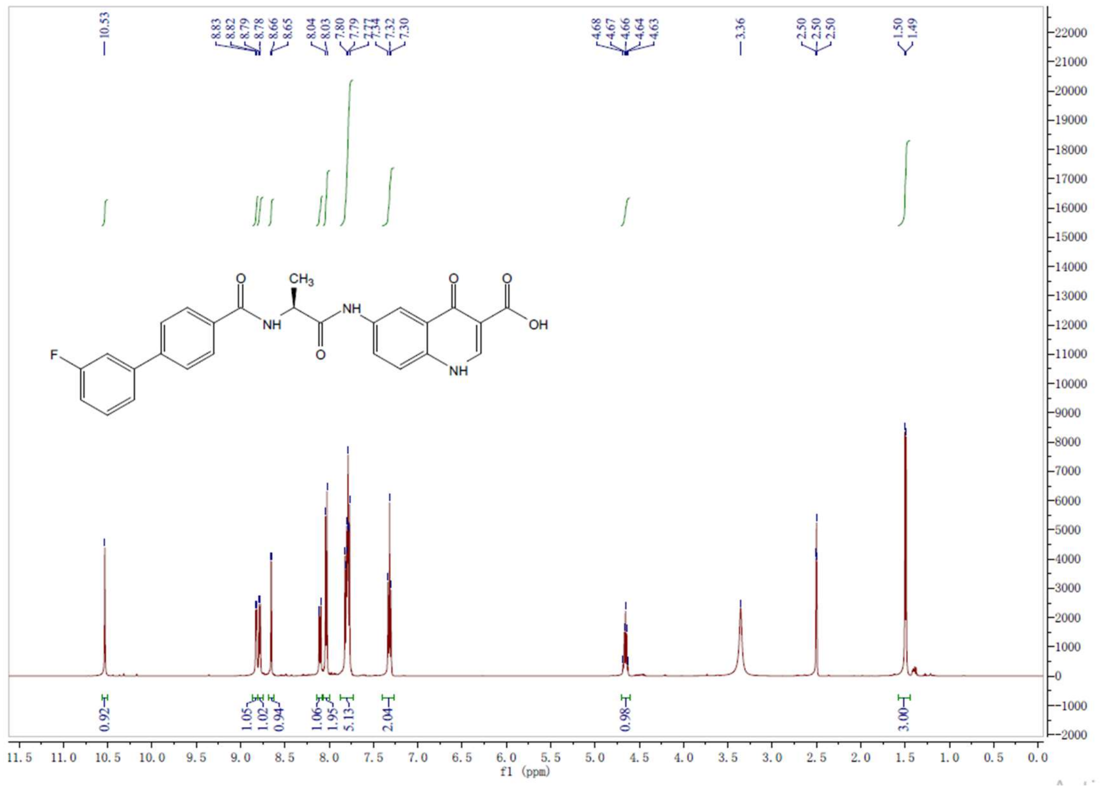

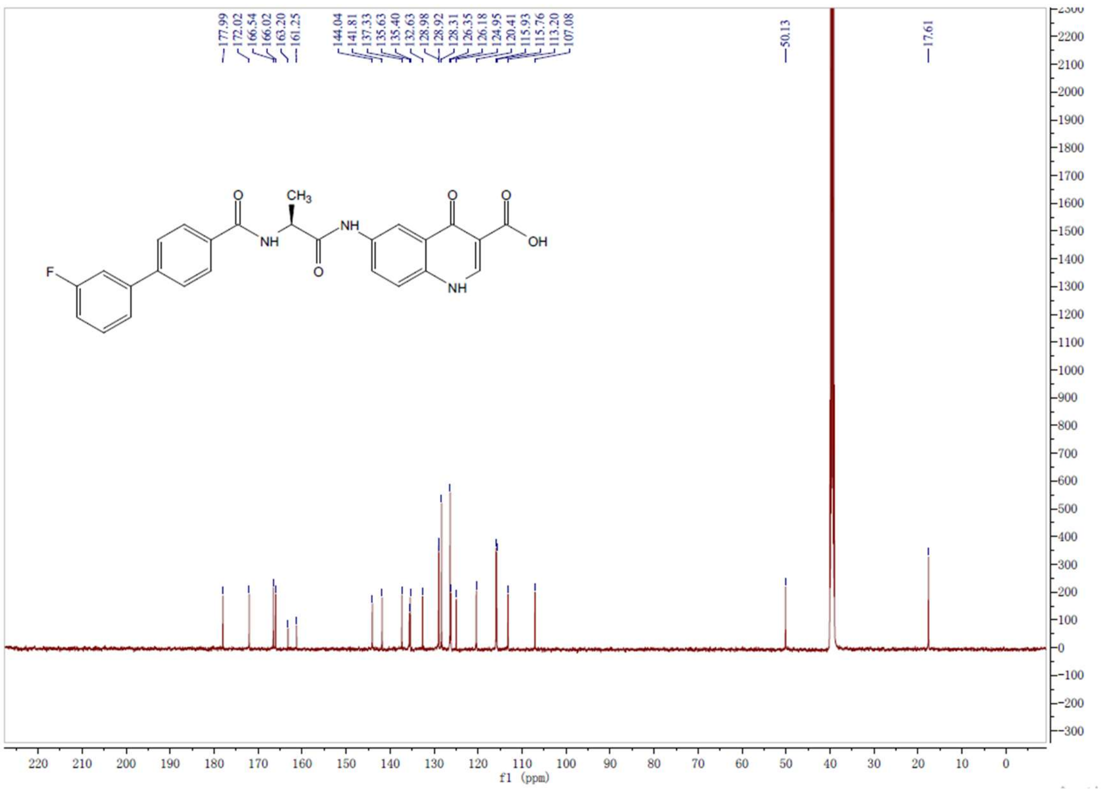

^{1}H NMR and ^{13}C NMR Spectra for L-19

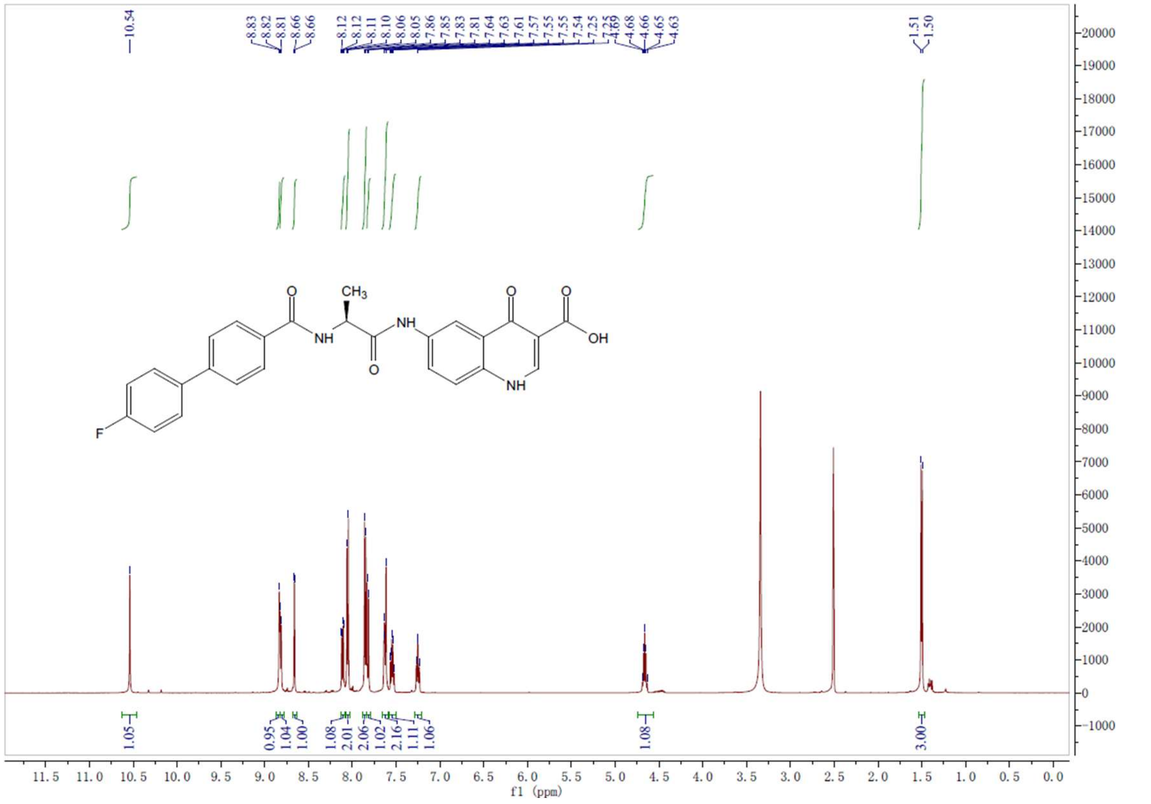

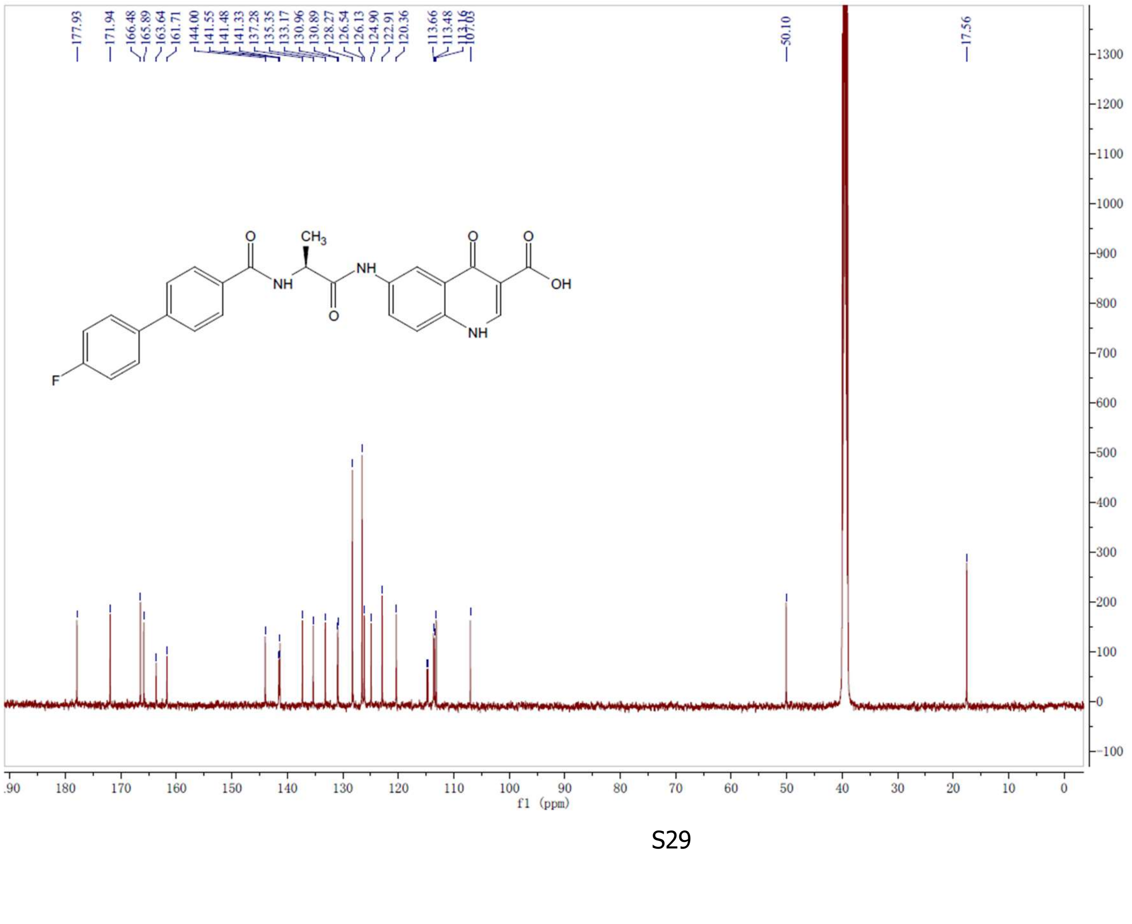

^{1}H NMR and ^{13}C NMR Spectra for L-20

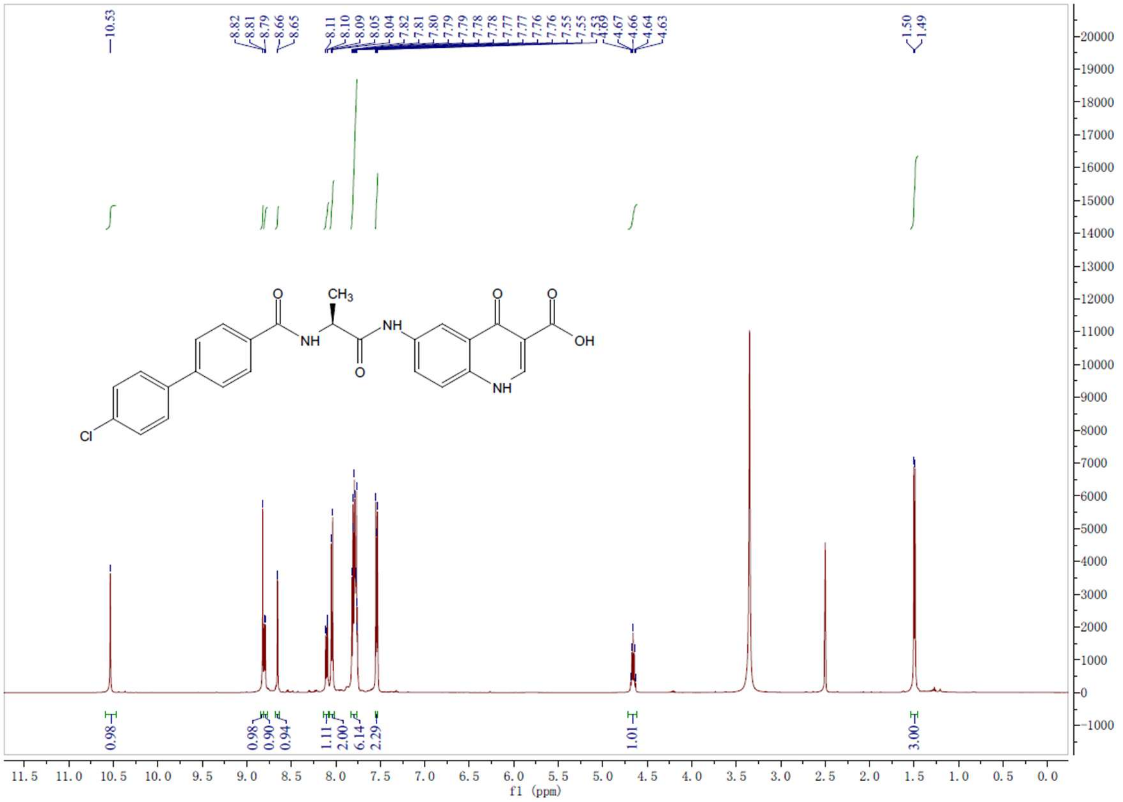

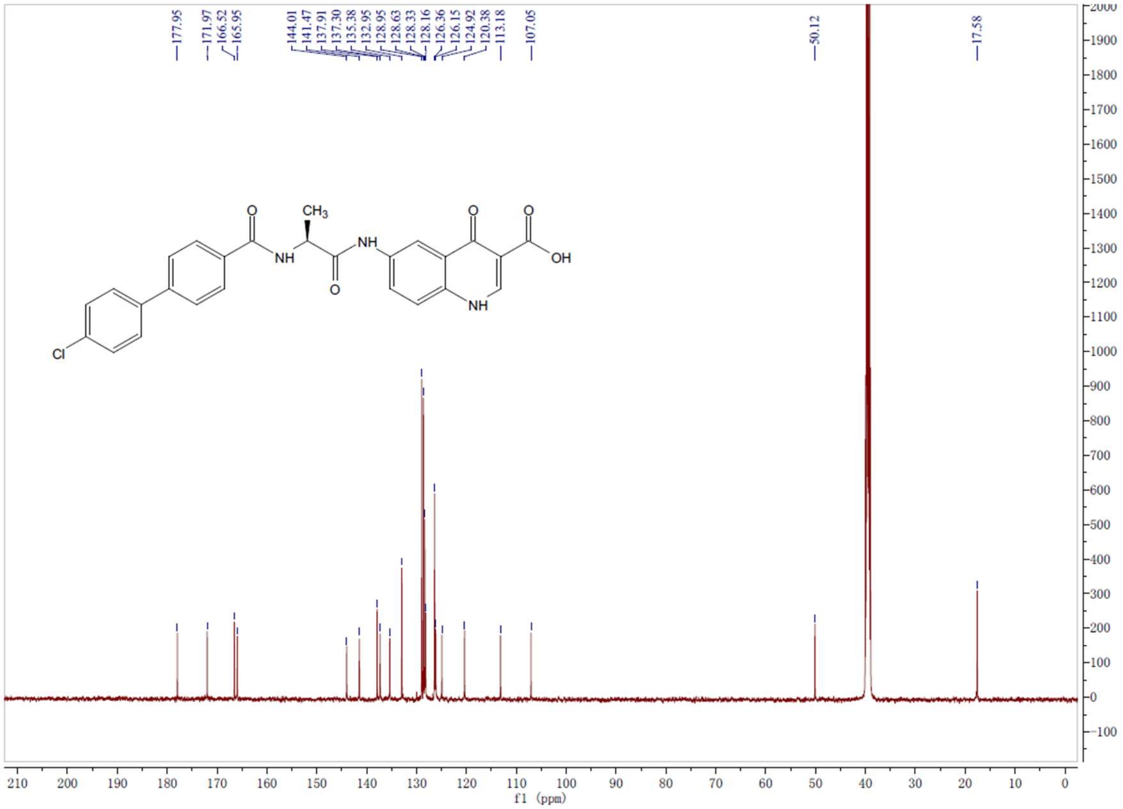

^{1}H NMR and ^{13}C NMR Spectra for L-21

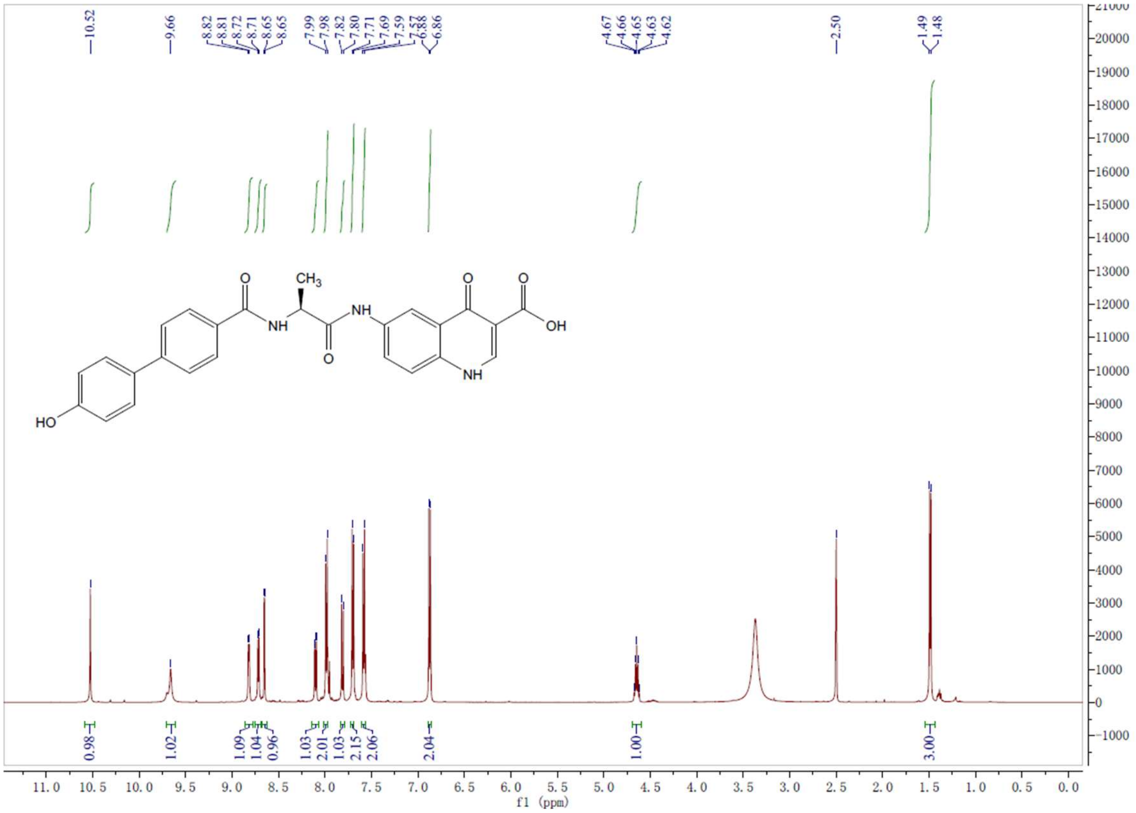

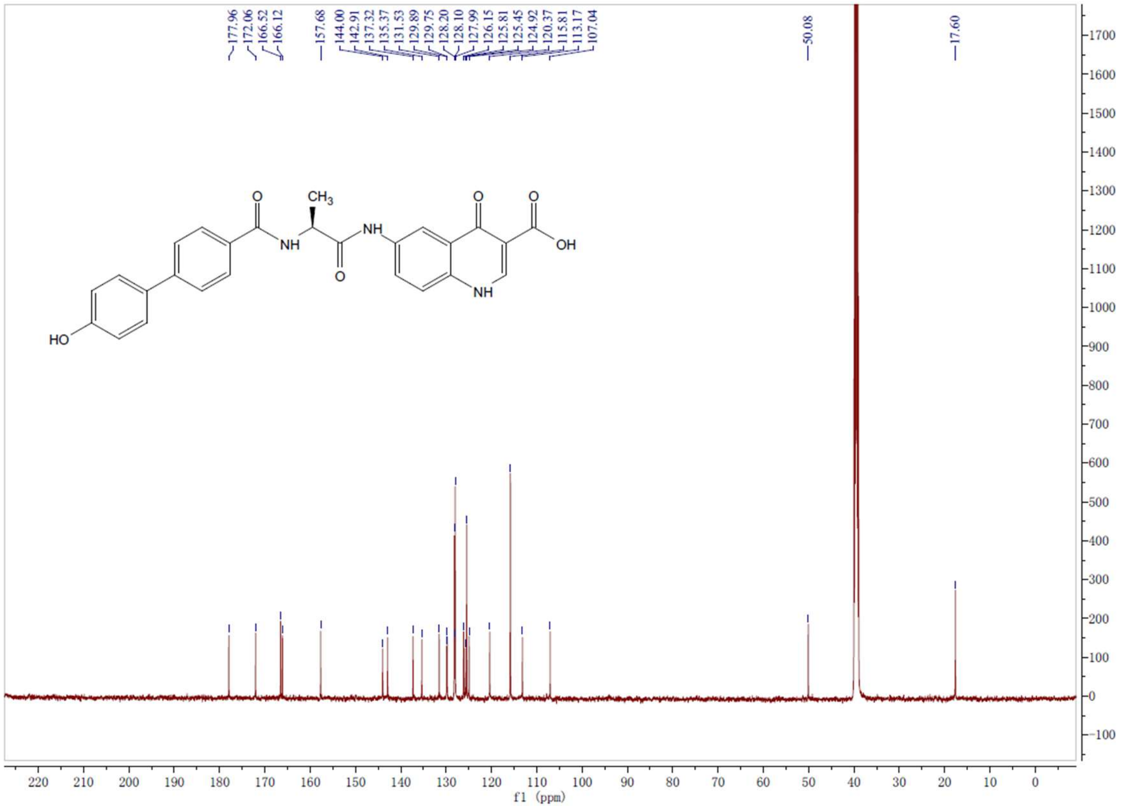

^{1}H NMR and ^{13}C NMR Spectra for L-23

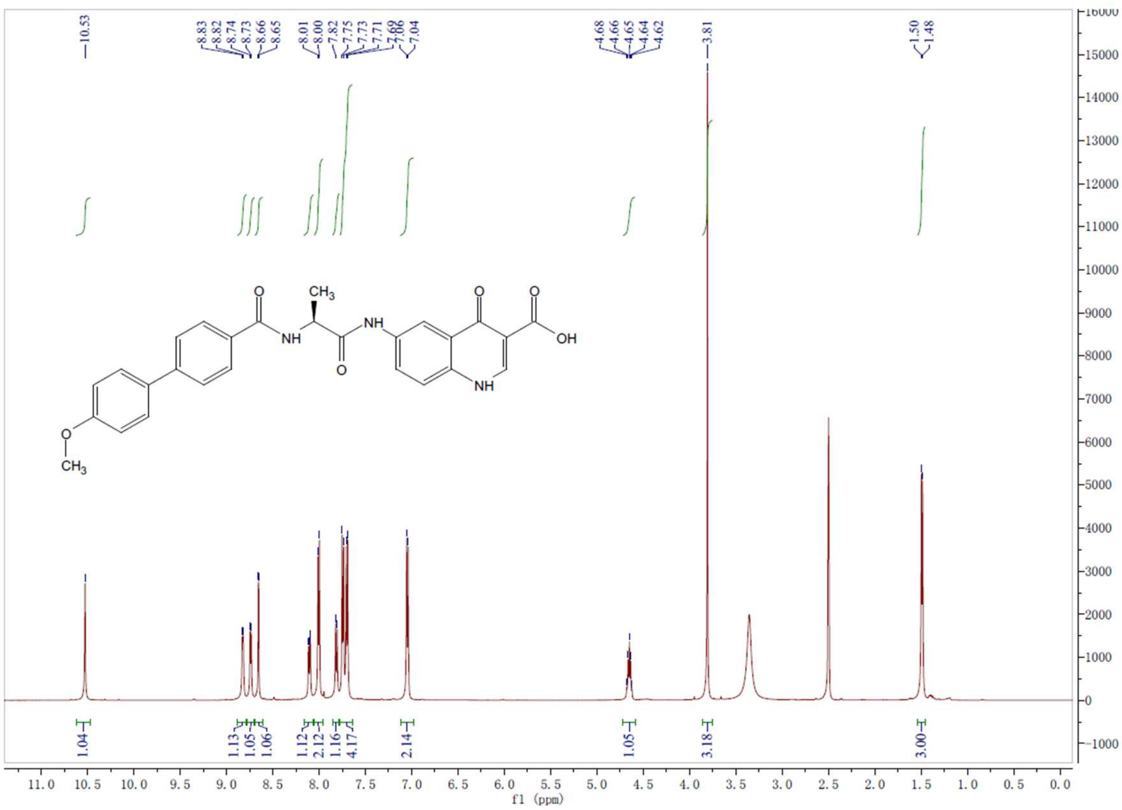

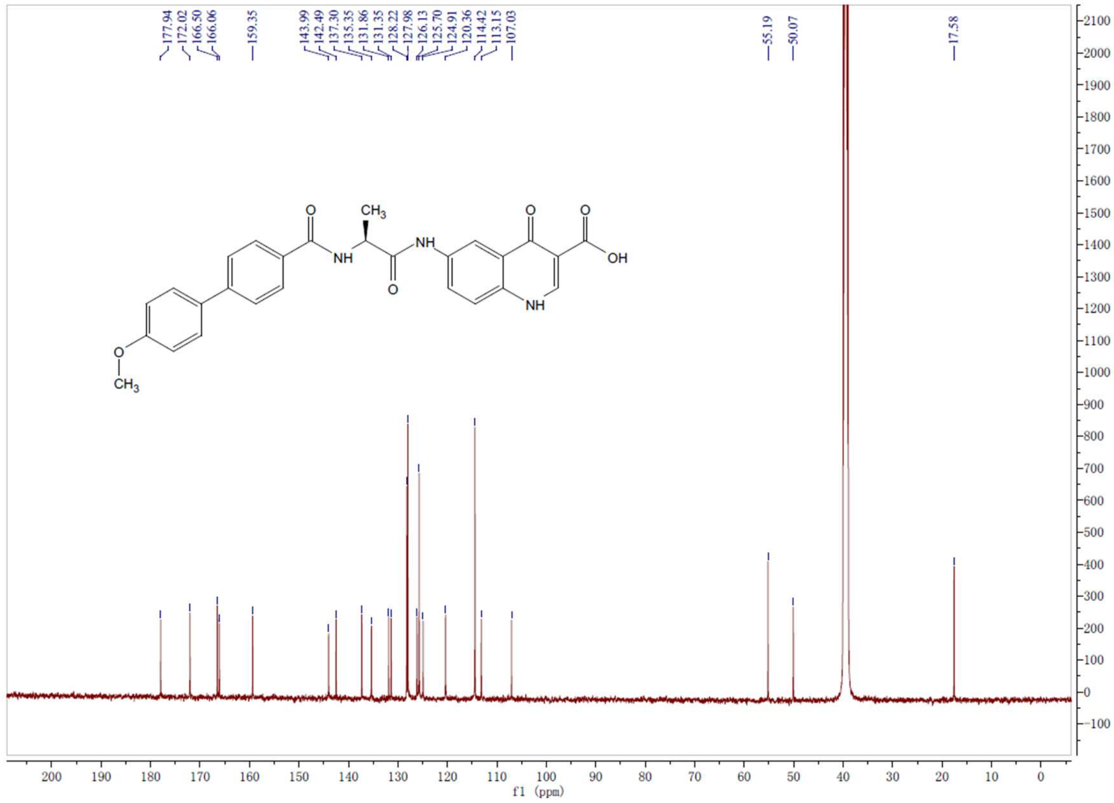

^{1}H NMR and ^{13}C NMR Spectra for L-24

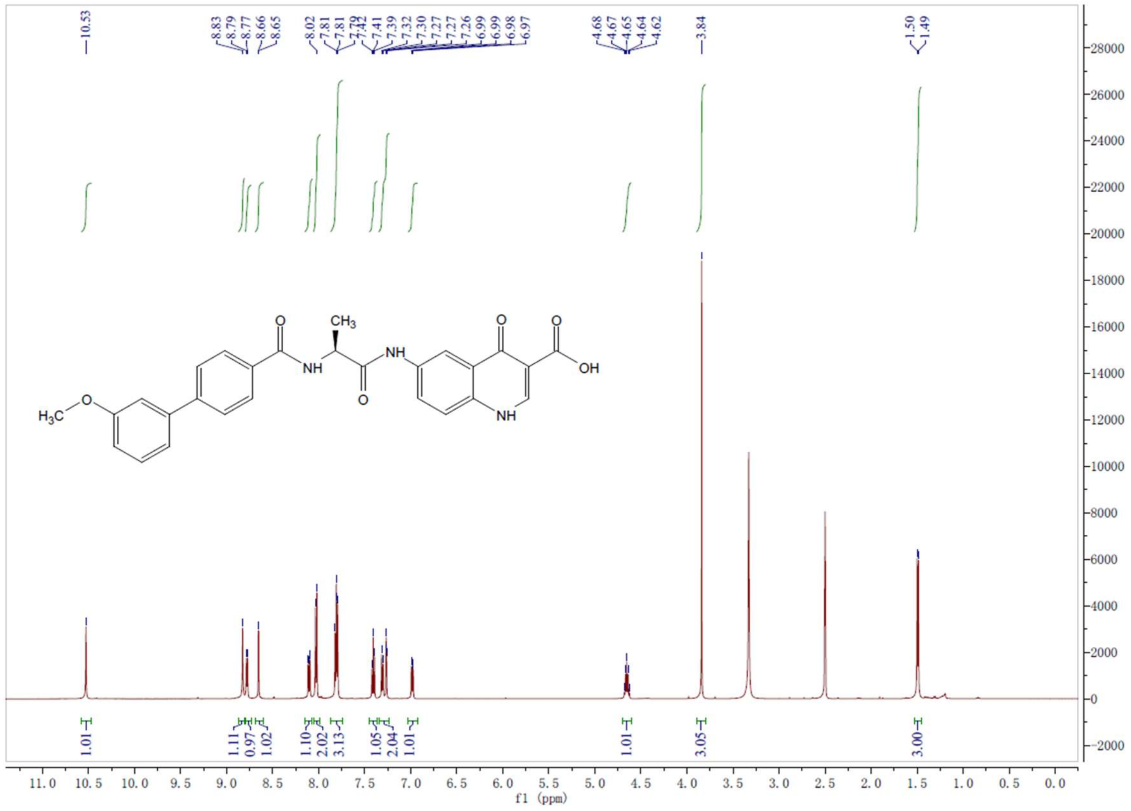

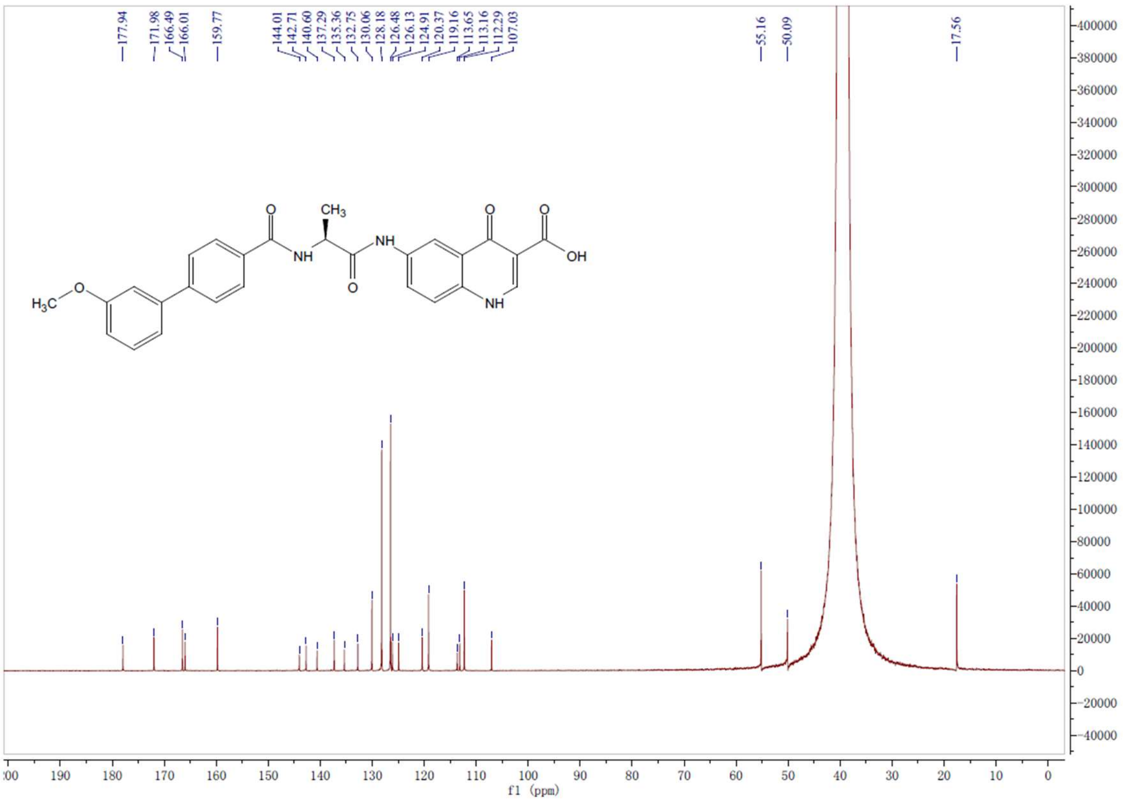

^{1}H NMR and ^{13}C NMR Spectra for L-25

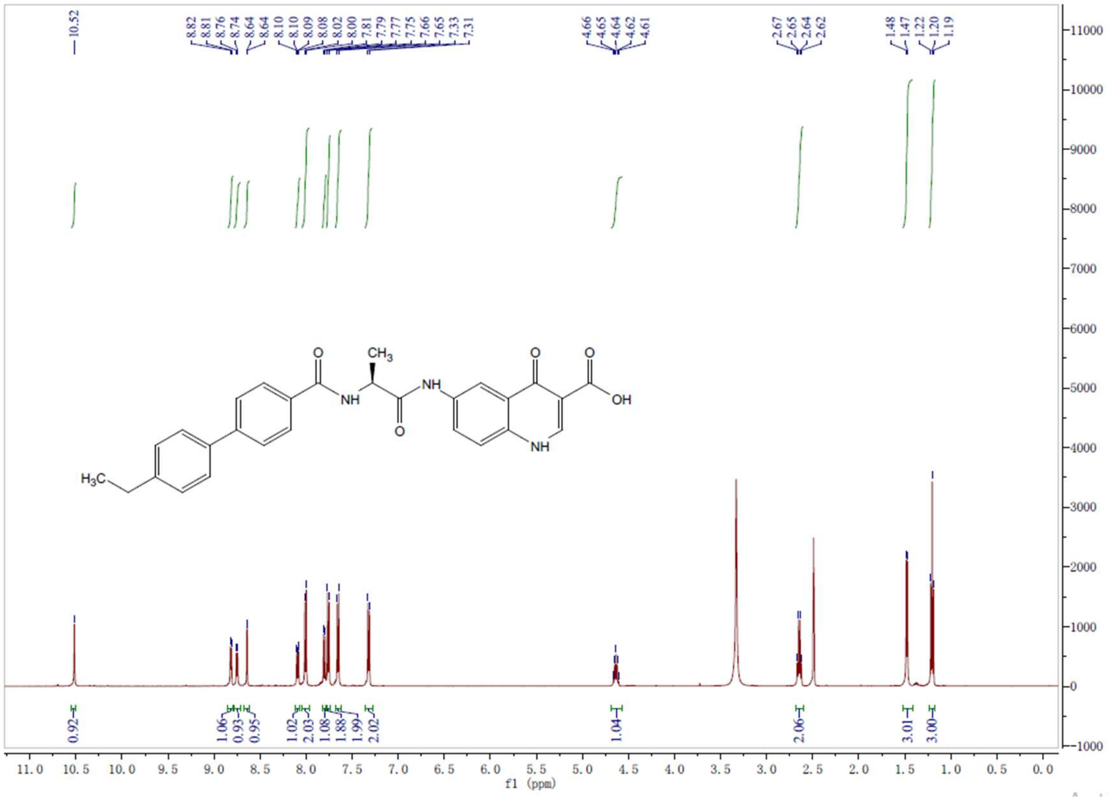

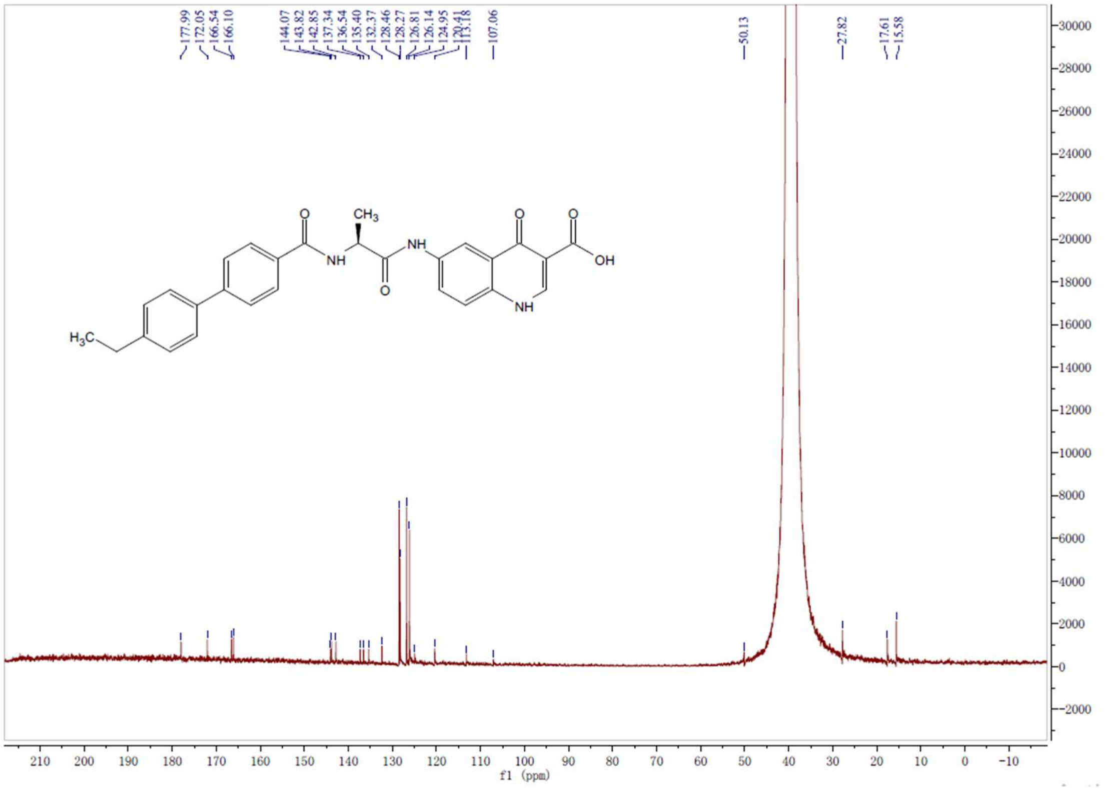

^{1}H NMR and ^{13}C NMR Spectra for L-26

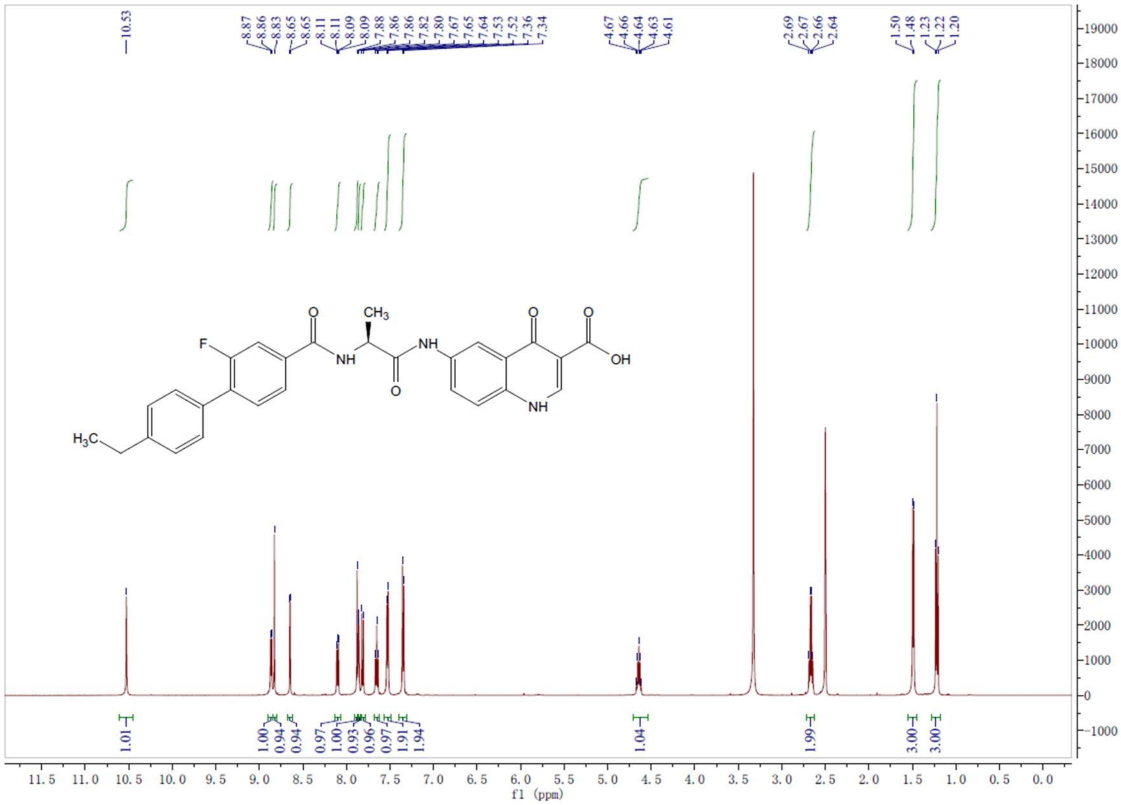

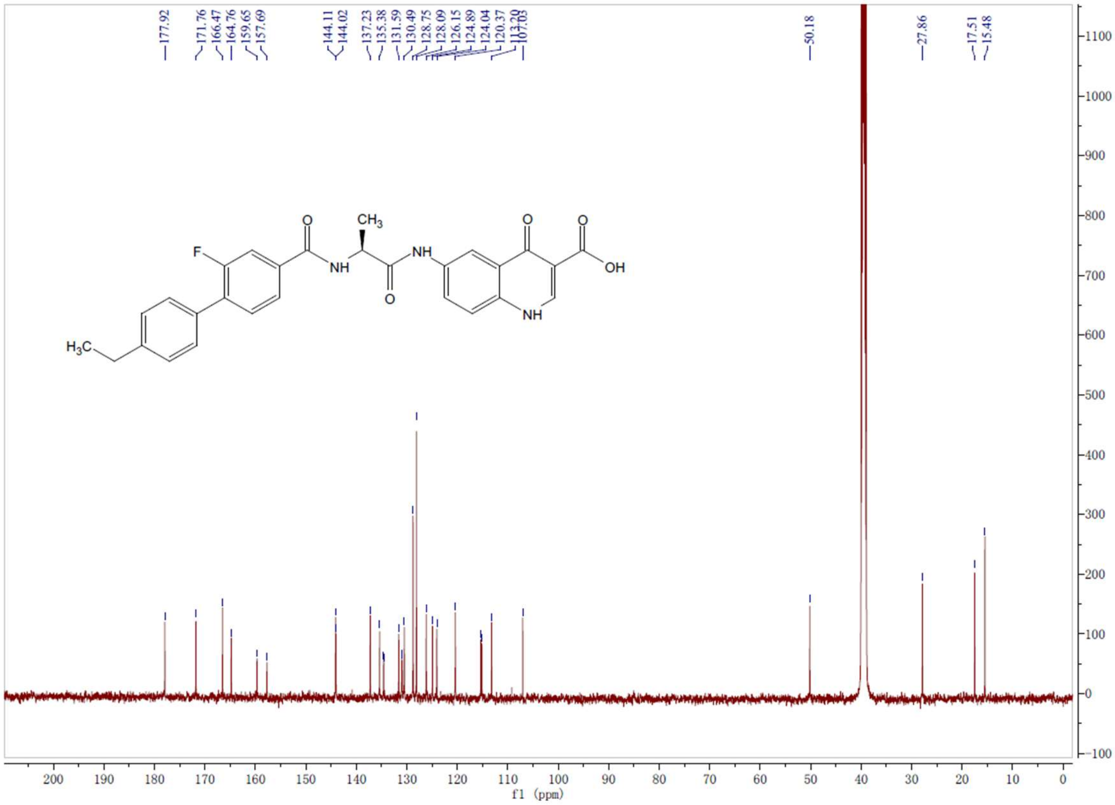

^{1}H NMR and ^{13}C NMR Spectra for L-28

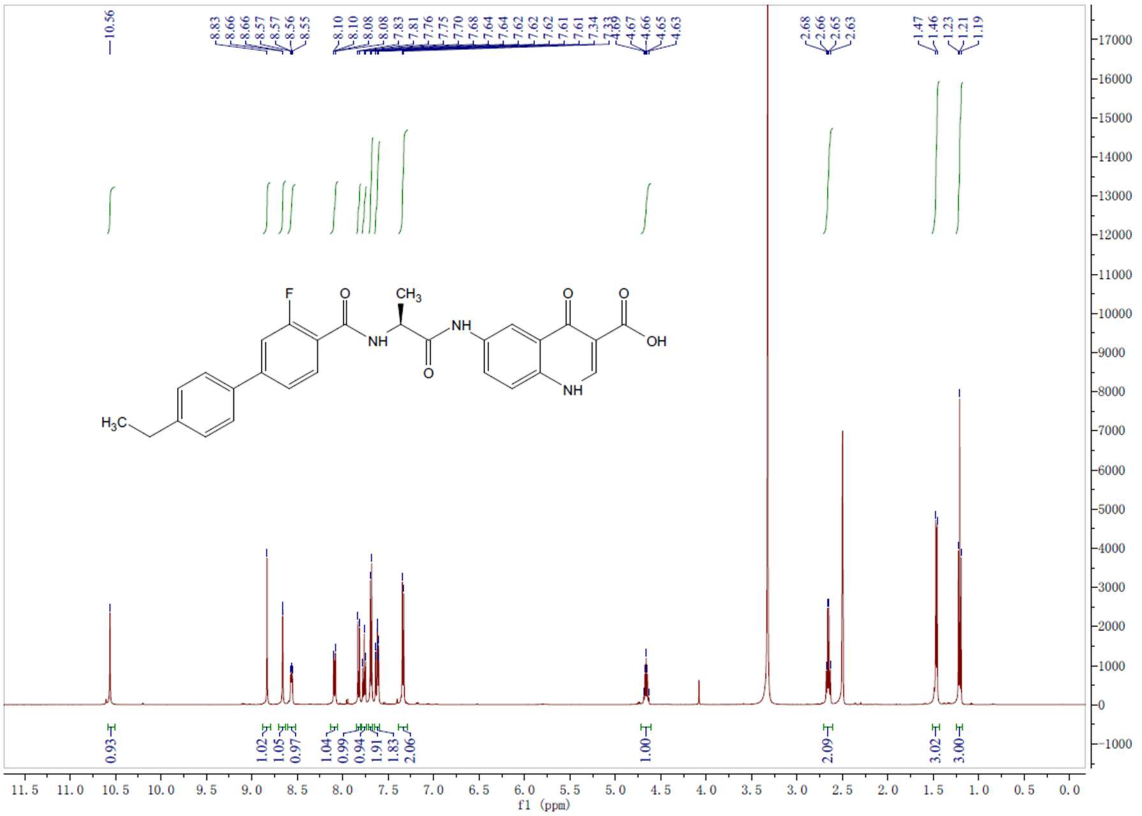

10.56
8.83 8.66 8.66 8.57 8.57 8.56 8.55
8.10 8.10 8.08 8.08 7.83 7.81 7.76 7.75 7.70 7.68 7.64 7.64 7.62 7.62 7.62 7.61 7.61 7.34 7.33
4.69 4.67 4.66 4.65 4.63
2.68 2.66 2.65 2.63
1.47 1.46 1.23 1.21 1.19
F
O
CH3
O
O
NH
NH
OH
O
NH
H3C
0.93
1.02
1.05
0.97
1.04
0.99
0.94
1.91
1.83
2.06
1.00
2.09
3.02
3.00
f1 (ppm)

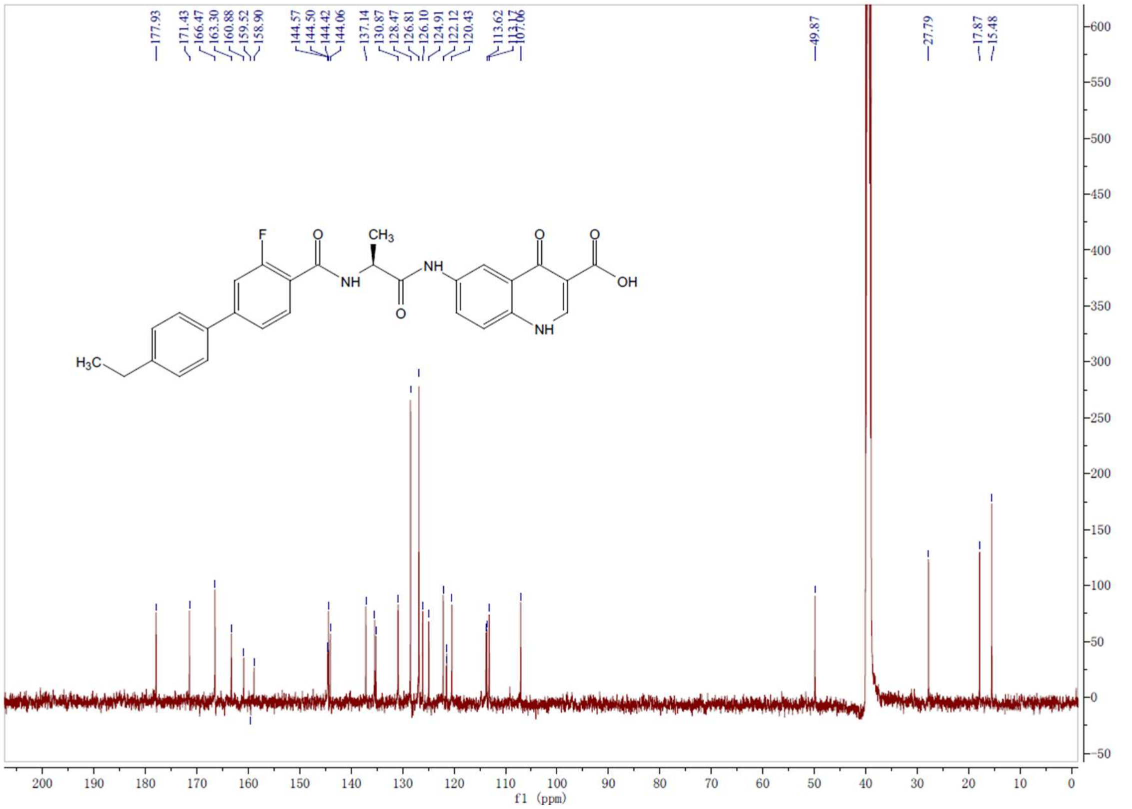

177.93 171.43 166.47 163.30 160.88 159.52 158.90
144.57 144.50 144.42 144.06
137.14 130.87 128.47 126.81 126.10 124.91 122.12 120.43
113.62 113.17 107.06
49.87
27.79
17.87 15.48
F
O
CH3
O
O
NH
NH
OH
O
NH
H3C
f1 (ppm)

^{1}H NMR and ^{13}C NMR Spectra for L-29

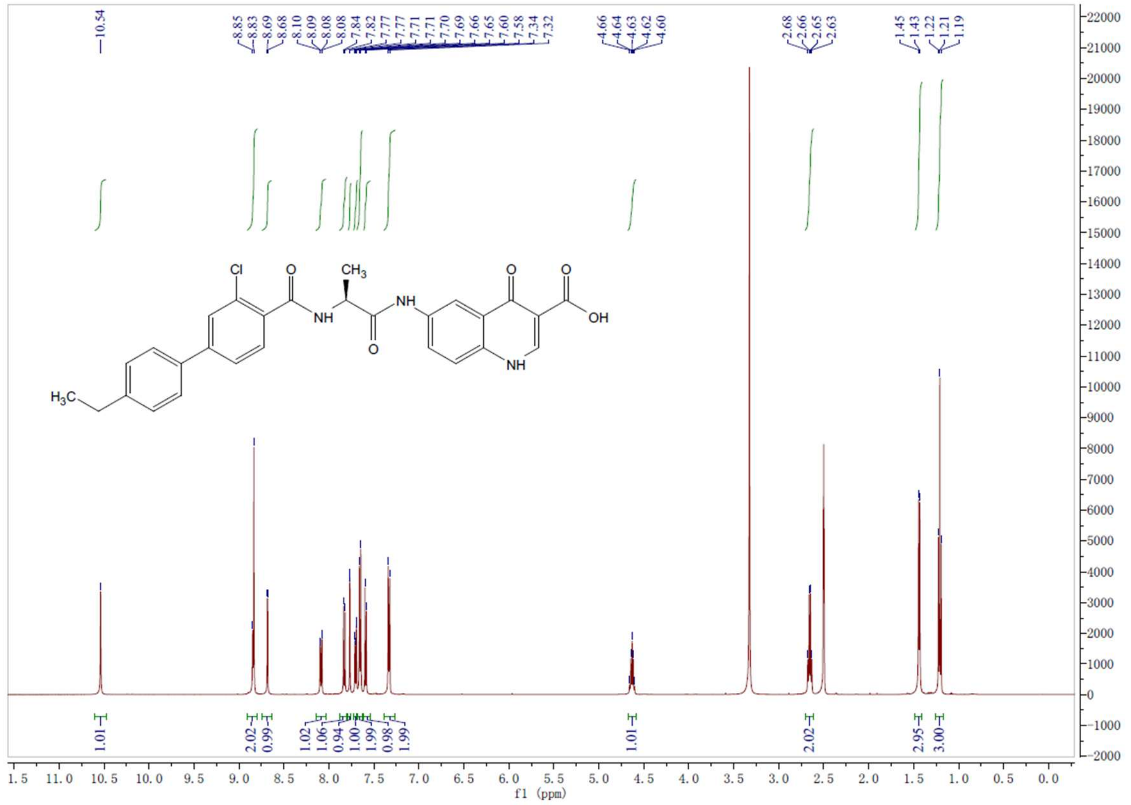

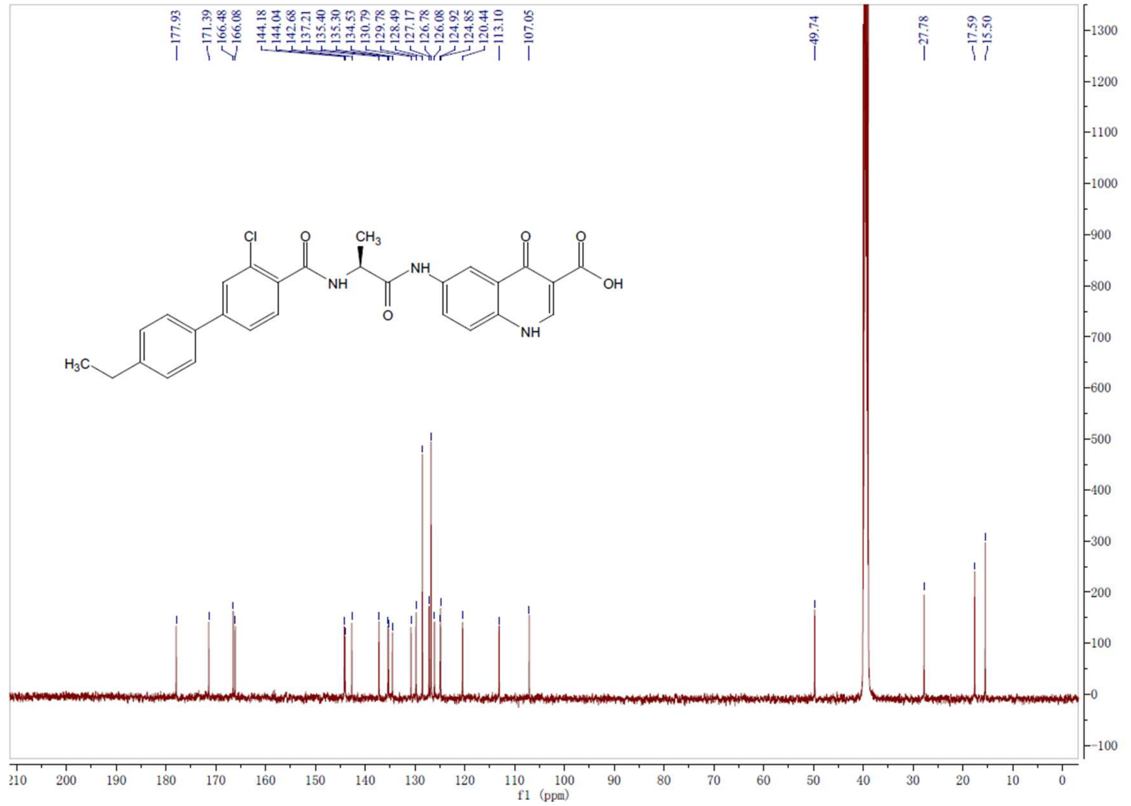

^{1}H NMR and ^{13}C NMR Spectra for L-30

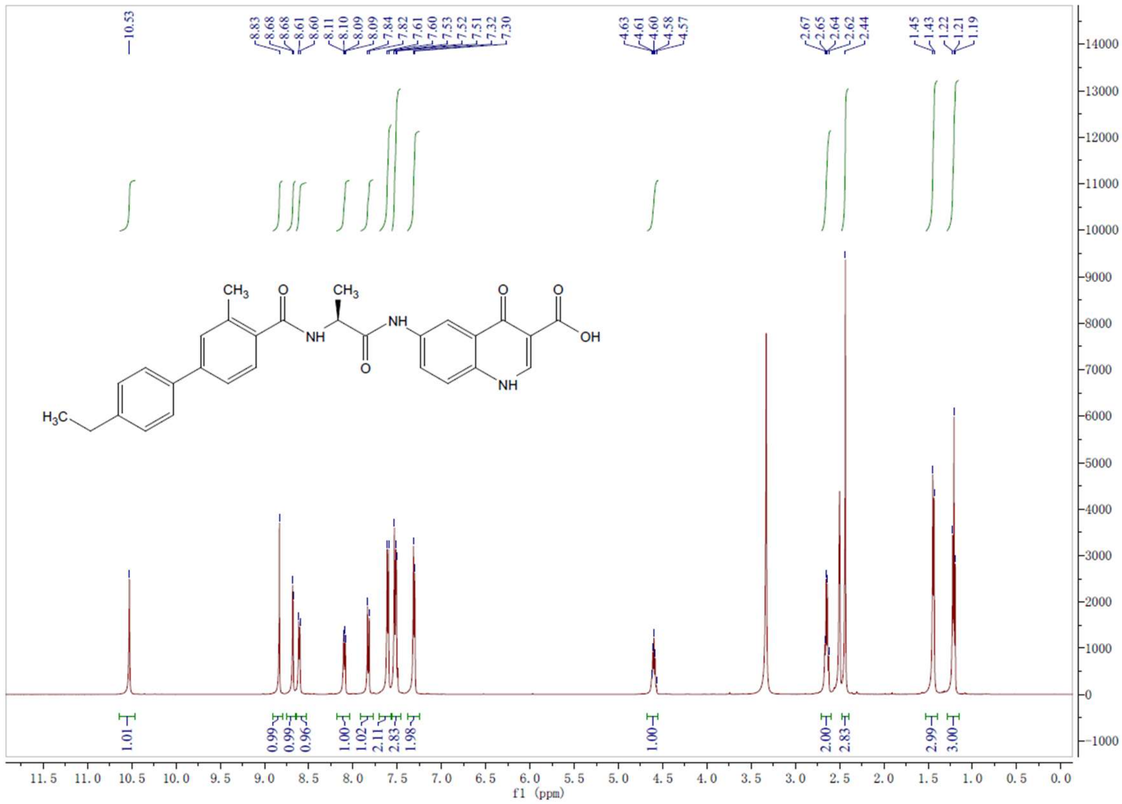

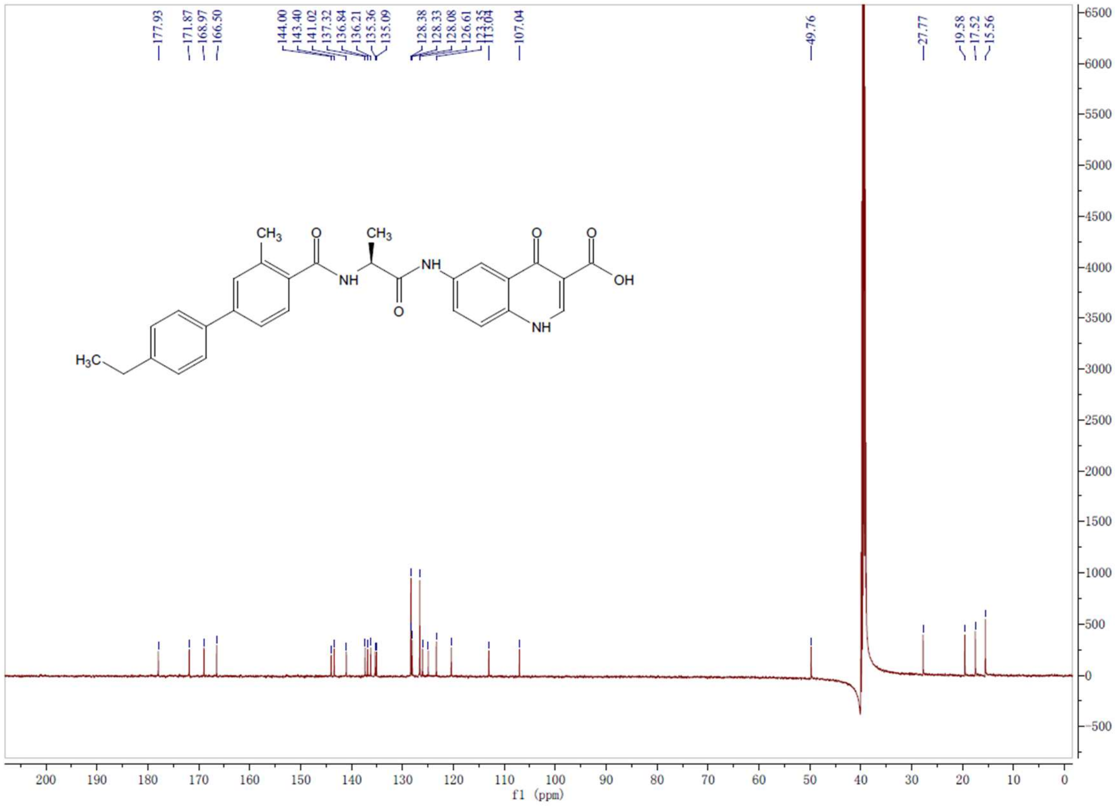

^{1}H NMR and ^{13}C NMR Spectra for L-31

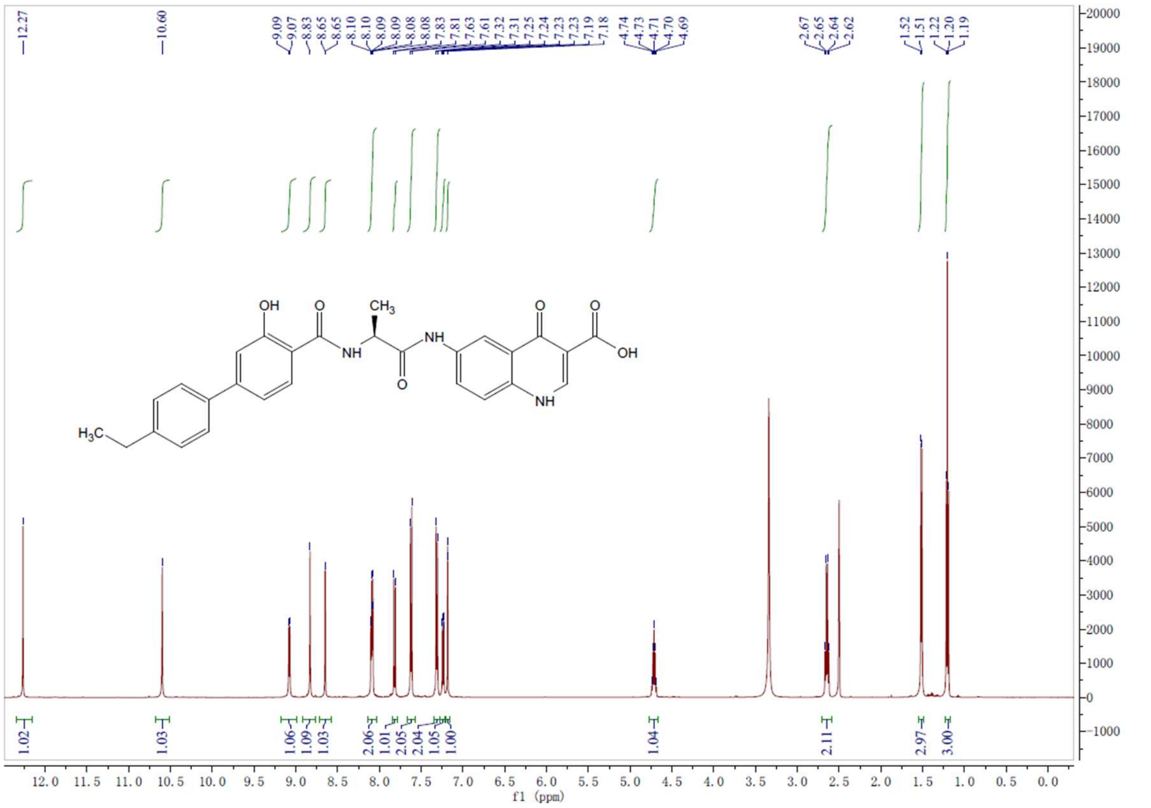

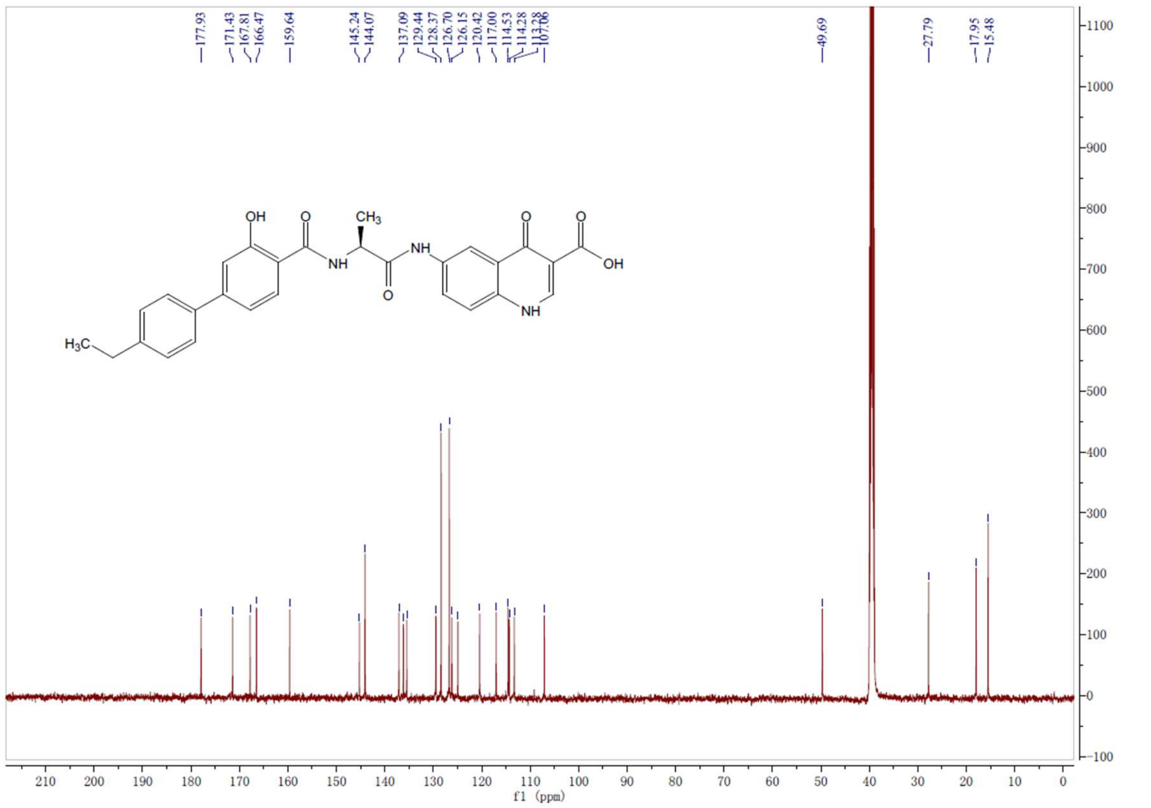

^{1}H NMR and ^{13}C NMR Spectra for L-32

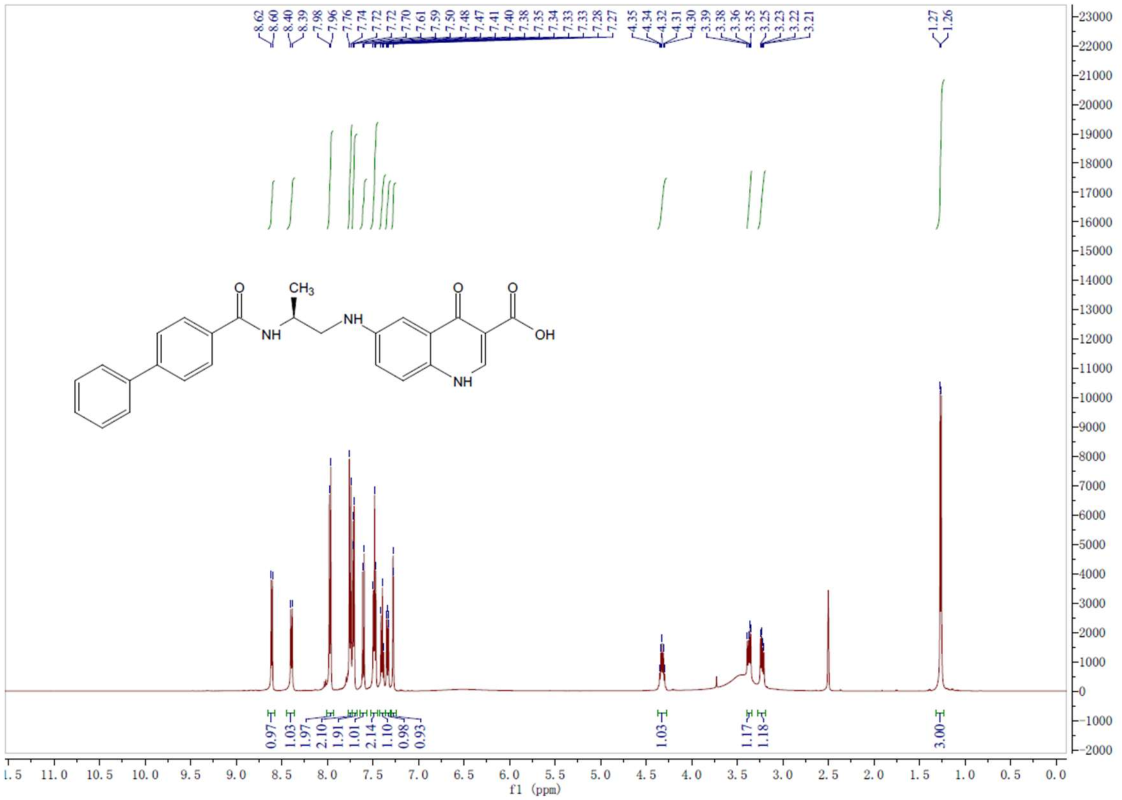

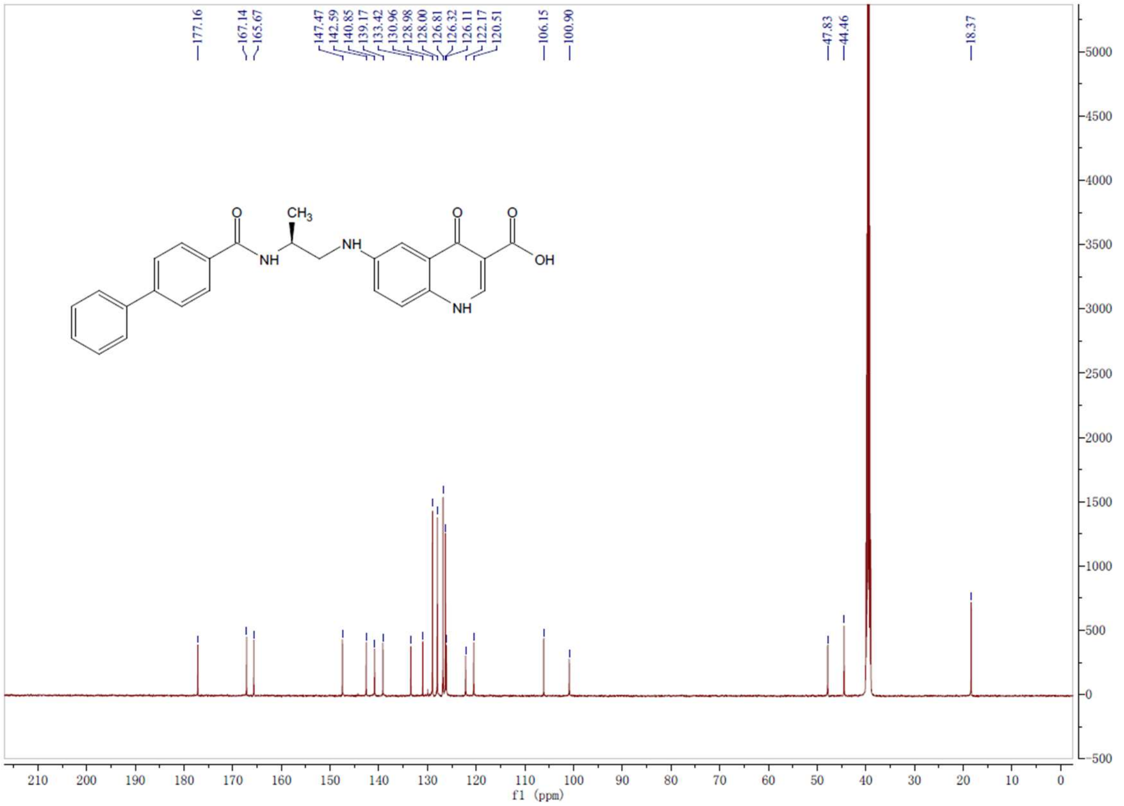

^{1}H NMR and ^{13}C NMR Spectra for L-35

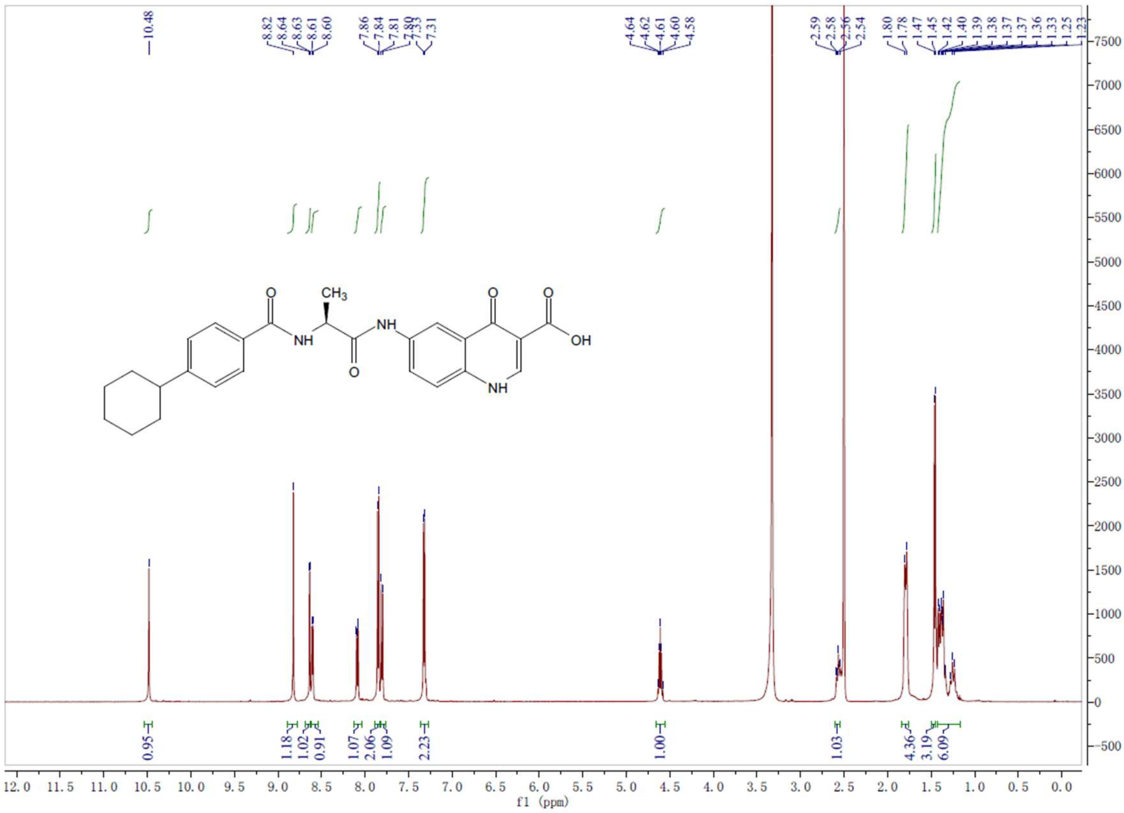

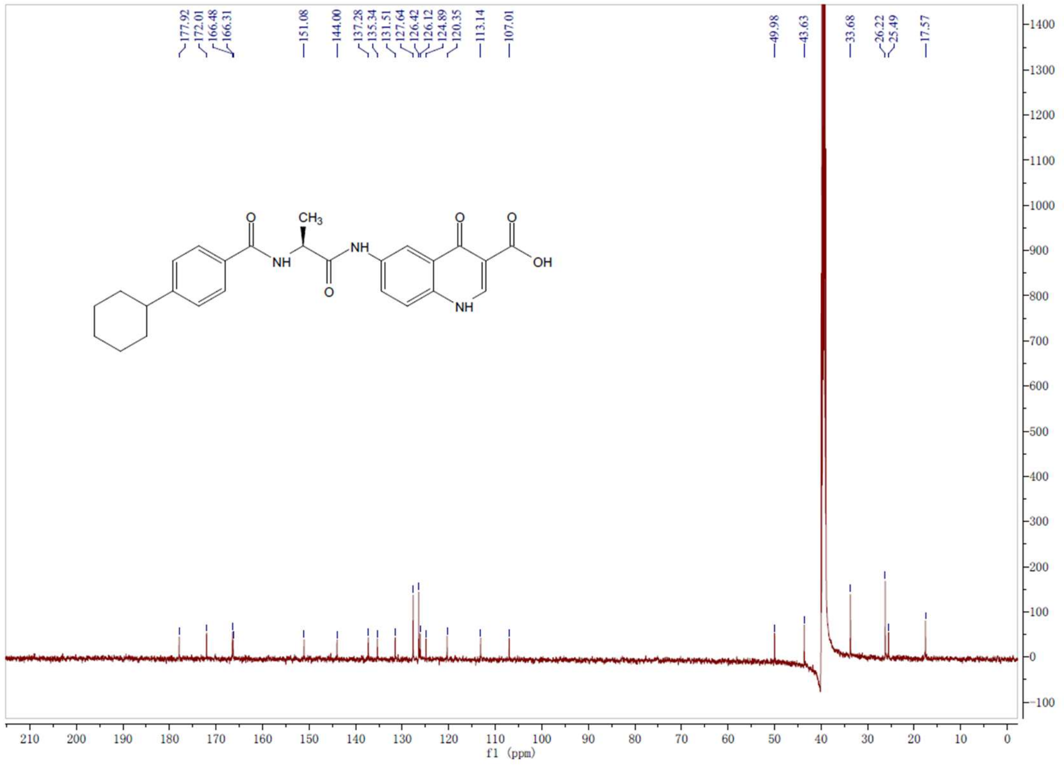

^{1}H NMR and ^{13}C NMR Spectra for L-36

LC/MS Traces and HRMS data for Compounds L-26, L-29, and L-32

LC/MS for Compound **L-26**

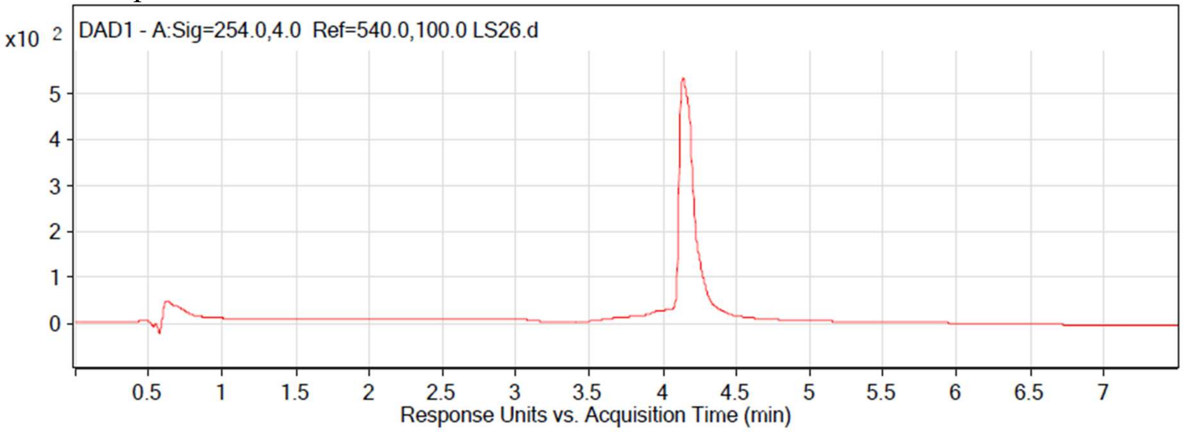

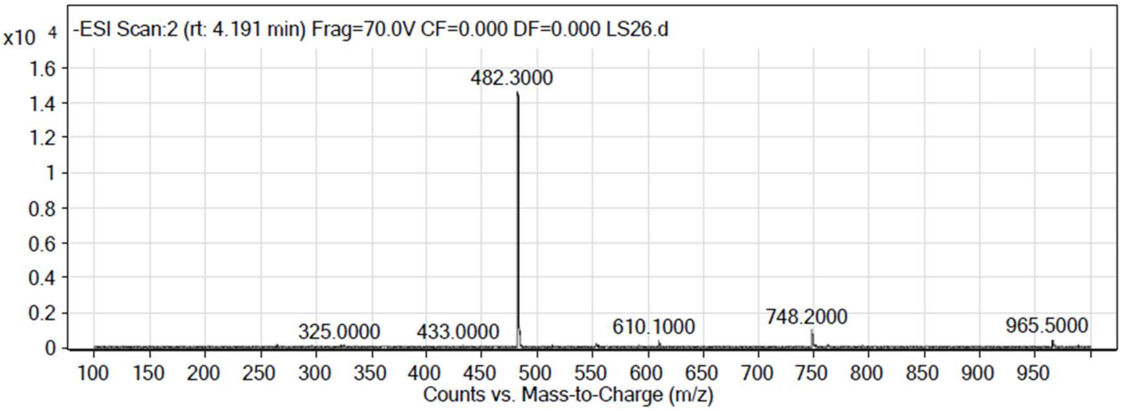

HRMS for Compound **L-26**

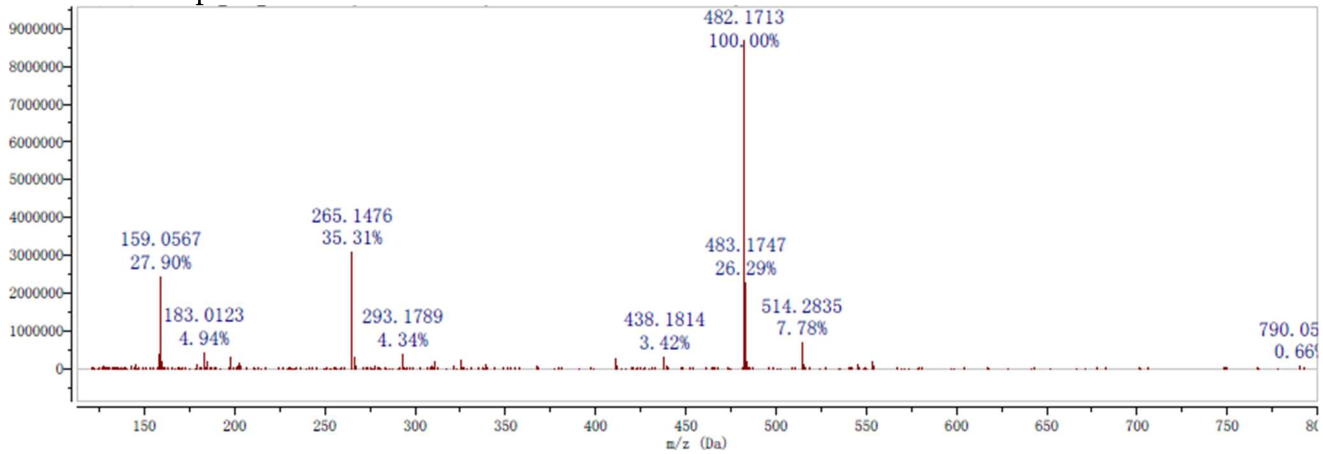

LC/MS for Compound **L-29**

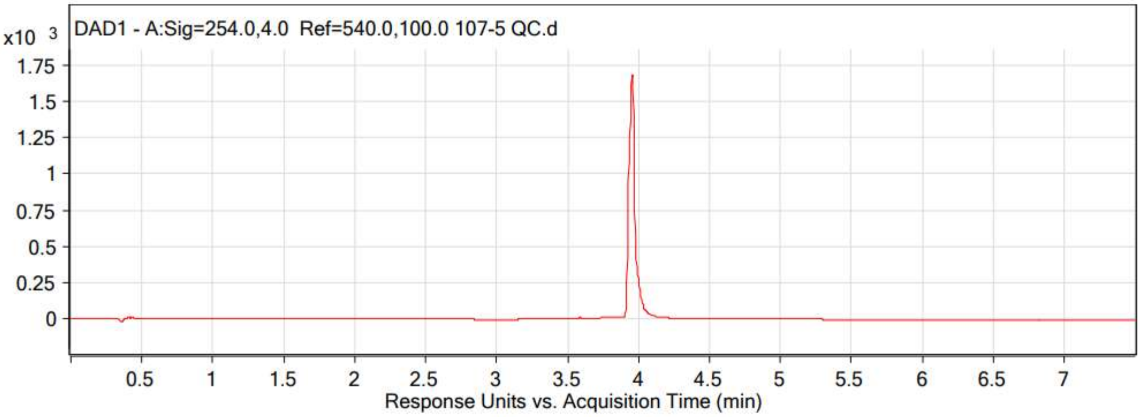

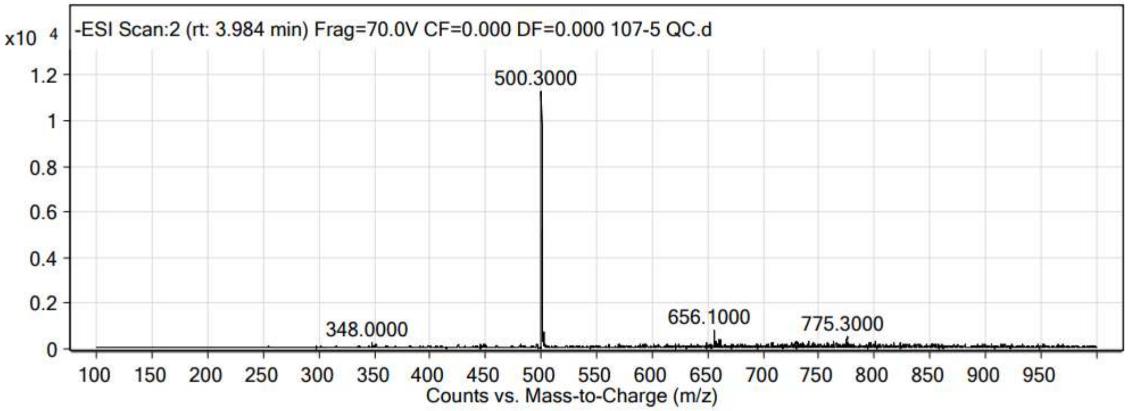

HRMS for Compound **L-29**

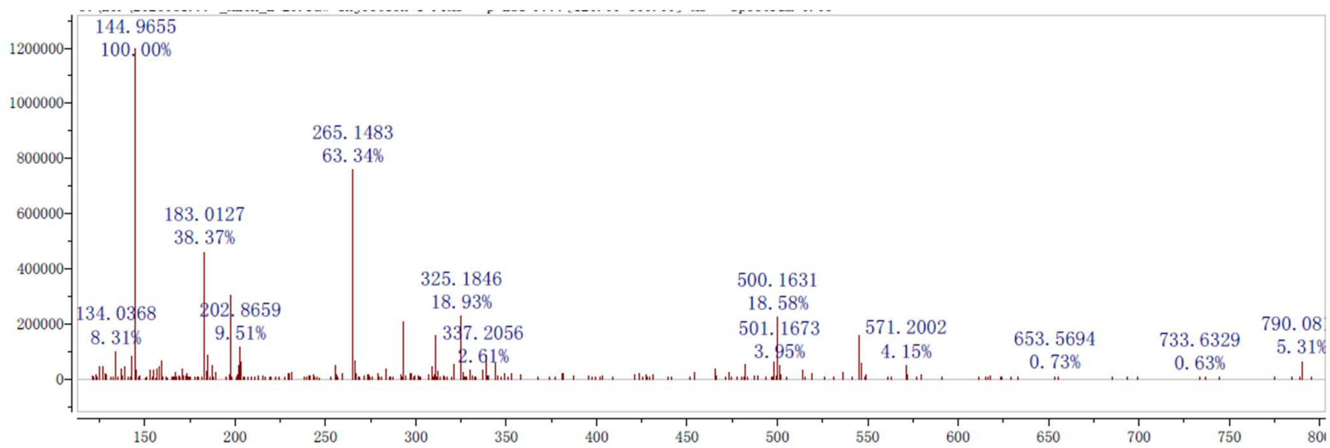

LC/MS for Compound **L-32**

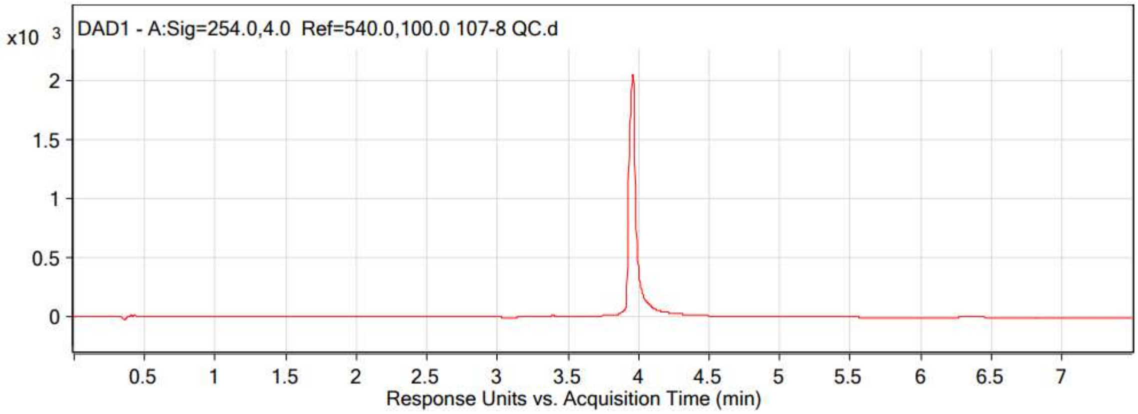

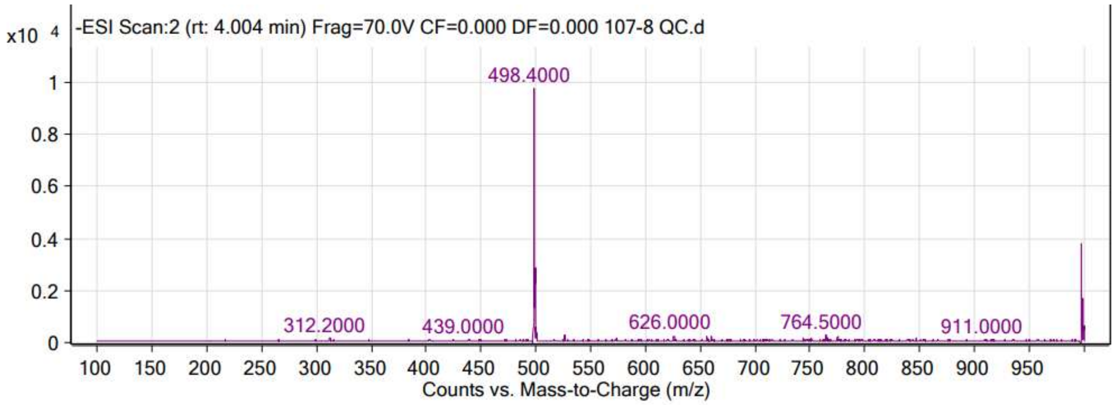

HRMS for Compound **L-32**

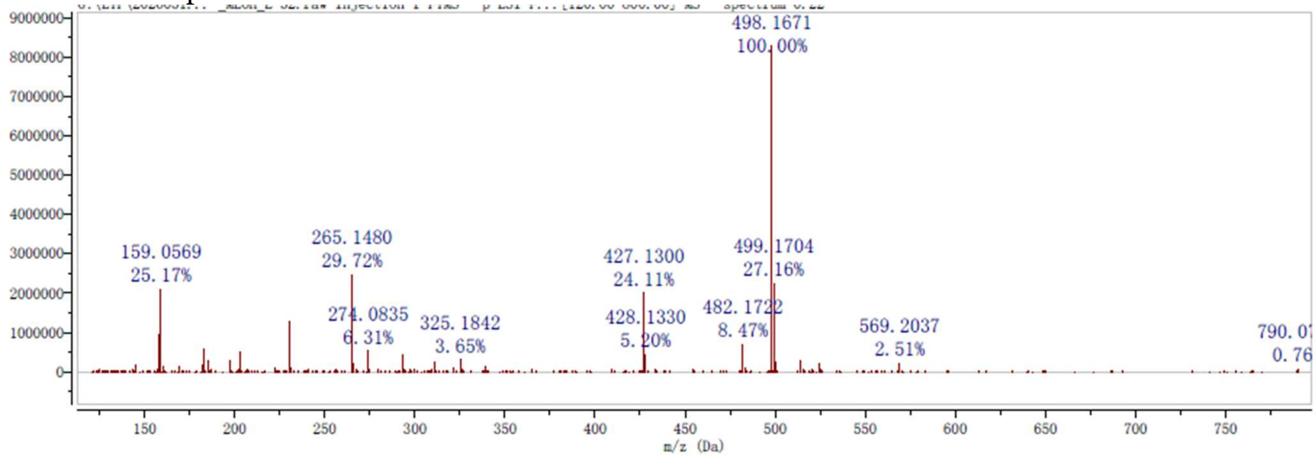

Supplement: Supplementary file 2 [file jm5c03467_si_002.pdf]
